# Query-guided protein–protein interaction inhibitor discovery†

**DOI:** 10.1039/d1sc00023c

**Published:** 2021-03-02

**Authors:** Sergio Celis, Fruzsina Hobor, Thomas James, Gail J. Bartlett, Amaurys A. Ibarra, Deborah K. Shoemark, Zsófia Hegedüs, Kristina Hetherington, Derek N. Woolfson, Richard B. Sessions, Thomas A. Edwards, David M. Andrews, Adam Nelson, Andrew J. Wilson

**Affiliations:** Astbury Centre for Structural Molecular Biology, University of Leeds Woodhouse Lane Leeds LS2 9JT UK a.j.wilson@leeds.ac.uk a.s.nelson@leeds.ac.uk; School of Chemistry, University of Leeds Woodhouse Lane Leeds LS2 9JT UK; School of Molecular and Cellular Biology, University of Leeds Woodhouse Lane Leeds LS2 9JT UK; School of Chemistry, University of Bristol Cantock's Close Bristol BS8 1TS UK; School of Biochemistry, University of Bristol Medical Sciences Building, University Walk Bristol BS8 1TD UK; BrisSynBio, University of Bristol Life Sciences Building, Tyndall Avenue Bristol BS8 1TQ UK; Early Oncology, AstraZeneca Hodgkin Building, Chesterford Research Campus, Saffron Walden Cambridge CB10 1XL UK david.andrews@astrazeneca.com

## Abstract

Protein–protein interactions (PPIs) are central to biological mechanisms, and can serve as compelling targets for drug discovery. Yet, the discovery of small molecule inhibitors of PPIs remains challenging given the large and typically shallow topography of the interacting protein surfaces. Here, we describe a general approach to the discovery of orthosteric PPI inhibitors that mimic specific secondary protein structures. Initially, hot residues at protein–protein interfaces are identified *in silico* or from experimental data, and incorporated into secondary structure-based queries. Virtual libraries of small molecules are then shape-matched against the queries, and promising ligands docked to target proteins. The approach is exemplified experimentally using two unrelated PPIs that are mediated by an α-helix (p53/*h*DM2) and a β-strand (GKAP/SHANK1-PDZ). In each case, selective PPI inhibitors are discovered with low μM activity as determined by a combination of fluorescence anisotropy and ^1^H–^15^N HSQC experiments. In addition, hit expansion yields a series of PPI inhibitors with defined structure–activity relationships. It is envisaged that the generality of the approach will enable discovery of inhibitors of a wide range of unrelated secondary structure-mediated PPIs.

## Abstract 2

Small-molecule protein–protein interaction inhibitors were prioritised on the basis of shape similarity to secondary structure-based queries incorporating hot-spot residues.

## Graphical abstract

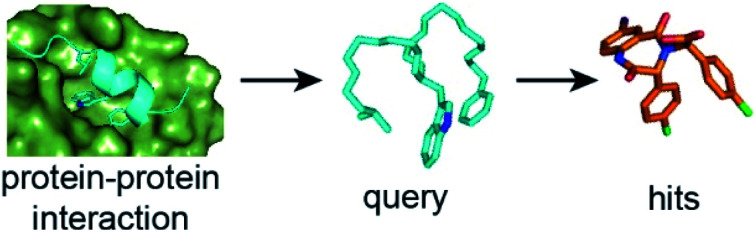

## Introduction

The discovery of small-molecule modulators of protein–protein interactions (PPIs) is a central challenge in both chemical biology and medicinal chemistry.^1–6^ For a number of PPI targets, potent PPI inhibitors and stabilizers have now been successfully discovered, for example by optimisation of high-throughput screening hits, fragment-based discovery approaches or virtual methods targeting p53/hDM2,^7–10^ the BCL-2 family^11–15^ and other interactions.^16–21^ However, the paucity of small-molecule PPI inhibitors that have progressed as clinical candidates,^3^ within the context of an enormous protein–protein interactome,^22^ provides continued motivation for development of novel and general small-molecule discovery approaches.

Although PPIs are known to involve shallow, relatively large interfaces of varied topography, the identification of hot-spots^23,24^ – *i.e.*, amino-acid residues that contribute significantly to binding – can provide focus for ligand design efforts. Moreover, a significant proportion of the protein–protein interactome involves short linear peptide sequences that adopt defined secondary structures, providing promising templates for design of orthosteric peptidomimetic inhibitors.^2,5^

Design approaches can be categorized into four groups.^5^ Class A mimetics are peptides with a limited number of modified amino acids introduced to optimize properties (*e.g.* stapled peptides). Class B mimetics are peptidic in nature but with more dramatic changes to structure; they include foldamers and mimic secondary structure topology. Class C mimetics are topographical mimics and are more small-molecule like; a core scaffold projects groups to mimic the orientation and composition of hot-spot residues. Class D mimetics are functional mimics and do not necessarily have a connection to structure.^25–29^ Class C mimetics – also termed proteomimetics – have successfully been developed to inhibit a range of α-helix-mediated PPIs, in some cases with selectivity and cellular activity.^25–29^ However, the proteomimetic approach has not been widely demonstrated for other classes of PPIs and generates simplified ligands (more likely to have off-target effects) with undesirable molecular properties (*e.g.* solubility, permeability) and hence limited scope for optimisation.^26^ Extension of the proteomimetic concept to small molecules with potential for rational medicinal chemistry optimisation into drug-like PPI inhibitors is therefore desirable.

**Fig. 1:**
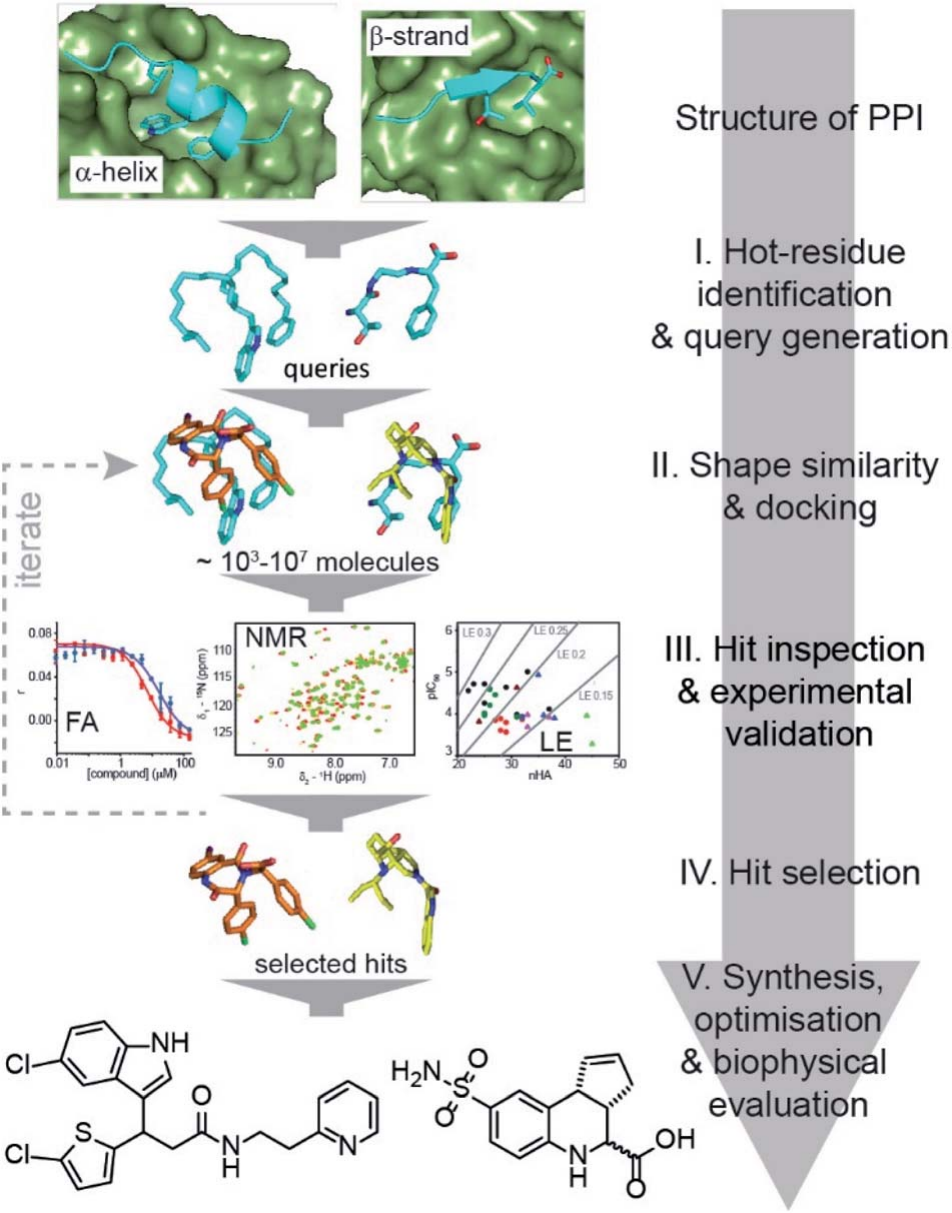
Schematic illustrating query-guided PPI inhibitor discovery in which small-molecules can potentially mimic any secondary structure. The discovery workflow is implemented through several stages that combine established computational tools and experimental validation. Initially a query is built that incorporates the key secondary structural motif and hot residues from the PPI. A virtual library of small molecules is then shape-matched against the query, and promising compounds docked against the target protein. Candidate inhibitors are then subjected to experimental screening and characterisation, enabling selection of hits for: (i) clustering, near neighbour expansion and further screening by reiteration of step II and III of the workflow; or (ii) further development.

Here, we exemplify a general approach for the discovery of small-molecule proteomimetic inhibitors of PPIs (Fig. 1) which we term: *Query-Guided PPI Inhibitor Discovery*. Hot-spot residues from a protein–protein interface are built into secondary structure-based queries. Assessment of the shape similarity^30–36^ of ligands to the query, in this case using FastROCS,^30,31,33–36^ enables small-molecule prioritisation. To provide additional confidence prior to experimental studies, further docking is performed. We demonstrate the power of the approach using two unrelated exemplar PPI targets: (i) p53/*h*DM2, an α-helix-mediated PPI^37^ that is a clinically relevant oncology target;^38^ and (ii) GKAP/SHANK1-PDZ,^39^ a β-strand-mediated PPI which plays a key role at the synaptic junction^40^ and is representative of PDZ-mediated interactions, which remain challenging for small-molecule inhibitor discovery.^41,42^ For both targets, we used this approach to virtually screen ∼4 million compounds for which physical samples were available in AstraZeneca's compound collection and then validated the approach experimentally using fluorescence anisotropy and ^1^H–^15^N HSQC biophysical screens. Hit-expansion for the p53/*h*DM2 inhibitor identified a series of PPI inhibitors with defined structure–activity relationships demonstrating the approach identifies developable compounds.

## Results and discussion

### Discovery of small-molecule p53/*h*DM2 inhibitors

**Fig. 2:**
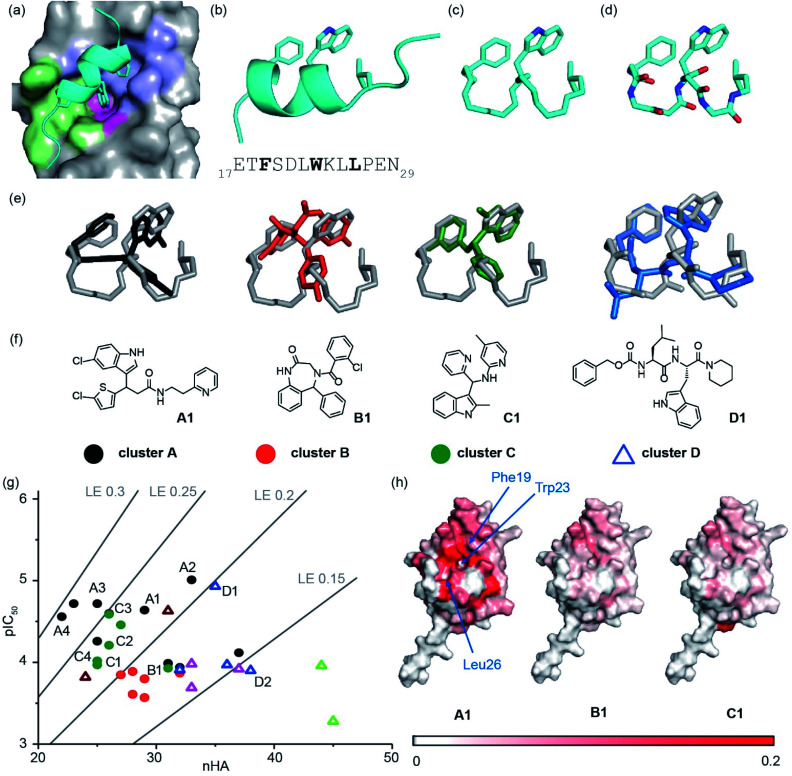
Discovery of small-molecule inhibitors of the p53/*h*DM2 interaction: (a) close-up of the p53/*h*DM2 interaction structure (PDB ID: 1YCR),^41^ p53 (cyan), with key side chains Phe19, Trp23 and Leu26 highlighted, docks into the *h*DM2 cleft with the *h*DM2 surface defined by the Phe19 (violet), Trp23 (magenta) and Leu 26 (green) pockets, into which each corresponding hydrophobic amino acid projects; (b) ribbon representation of the p53_17–29_ transactivation domain with key side chains Phe19, Trp23 and Leu 26 highlighted together with the primary sequence (below); (c) query that incorporates the hot residues and a “hydrocarbon” backbone; (d) query that incorporates the hot residues and a peptidic backbone. (e) Overlay of exemplar shape-matched hit compounds and queries; (f) structures of the exemplar hit compounds as representatives of the most populated clusters; (g) ligand efficiency plot for the 37 hits obtained from the computational workflow (LE = 1.4 × pIC_50_/nHA, nHA: number of non-hydrogen atoms, IC_50_ obtained by fluorescence anisotropy competition: 150 nM *h*DM2 and p53_15–31_Flu, 40 mM phosphate, pH 7.4, 200 mM NaCl and 0.02 mg ml^−1^ BSA); (h) mapping of the chemical shift perturbations in *h*DM2 for hit compounds from cluster A, cluster B and cluster C. Colour variation is associated with a chemical shift perturbation that goes from 0 ppm (white) to 0.2 ppm (red).

We used the p53/*h*DM2 interaction as an exemplar PPI to benchmark our discovery approach. The p53/*h*DM2 interaction involves thirteen residues of the disordered N-terminal transactivation domain of p53 (p53_17–29_ henceforth referred to as p53), which folds into a helix and docks into a hydrophobic cleft on *h*DM2 (Fig. 2a).^37^ Three key side chains – Phe19, Trp23 and Leu26 – located on one face of the helix (at the *i*, *i* + 4 and *i* + 7 positions), have been identified as making a dominant contribution to the binding free energy of association (Fig. 2a and b).^43,44^ These hot residues were used to construct queries for structure similarity searching. First, the p53 peptide was excised from a structure of p53/*h*DM2 (PDB: 1YCR), and a query was generated that contained the three hot-spot residues linked by a hydrocarbon backbone (Fig. 2c). A second query was generated in which a peptide backbone was retained (Fig. 2d).

A virtual library of 42 million conformers based on the AstraZeneca screening collection was shape-matched against both queries using FastROCS.^30,31,33–36^ For each query, the top 1000 hit conformers, based on the sum of the scoring functions for 3D shape match and surface complementarity, were selected. These conformers were then docked rigidly to *h*DM2 (OEDocking; ©2019, OpenEye Scientific Software, Inc.),^45–49^ and scored with a hybrid function that captured both shape similarity to the bound p53 peptide and shape complementarity to the *h*DM2 binding site (Fig. 1, stage II, see Fig. S1† for more detailed workflow including clustering,^50^ near neighbour expansion and further screening which can be reiterated as appropriate, and, ESI† for methodology). Next, the virtual hits were triaged ahead of experimental evaluation (Fig. 1, stage III). First, we removed fragment-like hits, a decision informed by observations made in the deconstruction of the known inhibitor RG7112: this retrospective analysis suggested a fragment-based discovery approach would only have been possible by starting with fragments that retained at least two hot-spot binding groups.^51^ Second, a significant number of compounds were removed because they had flipped during docking (*i.e.*, the docked pose no longer adopted a p53 mimicking orientation), and therefore did not fit with the design hypothesis. Third, the remaining hits were clustered (Tanimoto similarity >0.7), and one or two representatives were retained from each cluster (see ESI†); clusters with singleton hits were only retained if there was at least one near-neighbour in the main AstraZeneca screening collection. For each query, ∼100 compounds were prioritised for experimental evaluation, including representatives of the top 30 clusters that were predicted to interact with at least two hot residue binding pockets. In addition, 100 randomly selected compounds were also selected for analyses (see ESI†).

The ability of compounds to displace a fluorescently labelled p53 peptide (p53_15–31_Flu (L31C), Flu = fluorescein-5-maleimide) was determined using a competition fluorescence anisotropy assay (see ESI Fig. S2† for assay and Fig. S3–S6† for % inhibition at 10 and 100 μM). For compounds that displayed significant activity at 100 μM, structurally similar near-neighbours were selected from the main AstraZeneca screening collection for hit expansion. For the query with a hydrocarbon backbone, 21 hits from 15 clusters were identified, and were complemented with 100 near-neighbours. For the query with the peptide backbone, 3 hits from 3 clusters were identified, and were complemented with 11 near-neighbours. These 135 compounds were evaluated at 10 μM and 100 μM in the competition fluorescence anisotropy assay (not shown). Subsequently, based on visual inspection and team discussion of how well they matched the initial FastROCS shape-match and docking, alongside medicinal chemistry and synthetic assessment, 63 compounds from 9 clusters (*e.g.*Fig. 2e and f) were selected for IC_50_ determination (Fig. S6†). A number of these compounds had mediocre inhibitory potency that fell outside the assay window leaving 37 compounds for which IC_50_ values could be determined, allowing an assessment of their ligand efficiency (Fig. 2g), *i.e.* the binding energy per non-hydrogen atom of a ligand to its binding partner which is commonly used in drug discovery programmes to assist in identifying compounds with optimal combinations of properties.^52,53^

Mapping of chemical shift perturbations by ^1^H–^15^N HSQC for representatives from each promising cluster confirmed a *bona fide* interaction with *h*DM2 (Fig. 2h and S7–S9†). We used ligand efficiency scores and chemical accessibility to eventually select compound **A1** (Fig. 2) as a hit upon which to carry out structure activity relationship (SAR) analysis (see later and Fig. 4). At IC_50_ = 7.9 (±0.9) μM, hit compound **A1** with MW = 444 is only one order of magnitude lower in inhibitory potency than Nutlin-3 (IC_50_ = 0.6 ± 0.04 μM; MW = 581, see ESI Fig. S8†). However, it has a comparable ligand efficiency of 0.24 (*versus* 0.22 for Nutlin-3) and significant potential for further derivatization and optimization. Compound **A1** was also shown to induce shifts in the ^1^H–^15^N HSQC spectrum and when mapped onto the residue assignments, the affected resonances corresponded well to those affected upon binding of Nutlin-3, lending support to the design hypothesis. Compound **A1** was also tested in a FA competition assay for BID/MCL-1 (Fig. S10†), a further α-helix mediated PPI: no inhibition was observed, indicating the compound **A1** to be selective and confirming it as a good choice for further development.

**Fig. 3:**
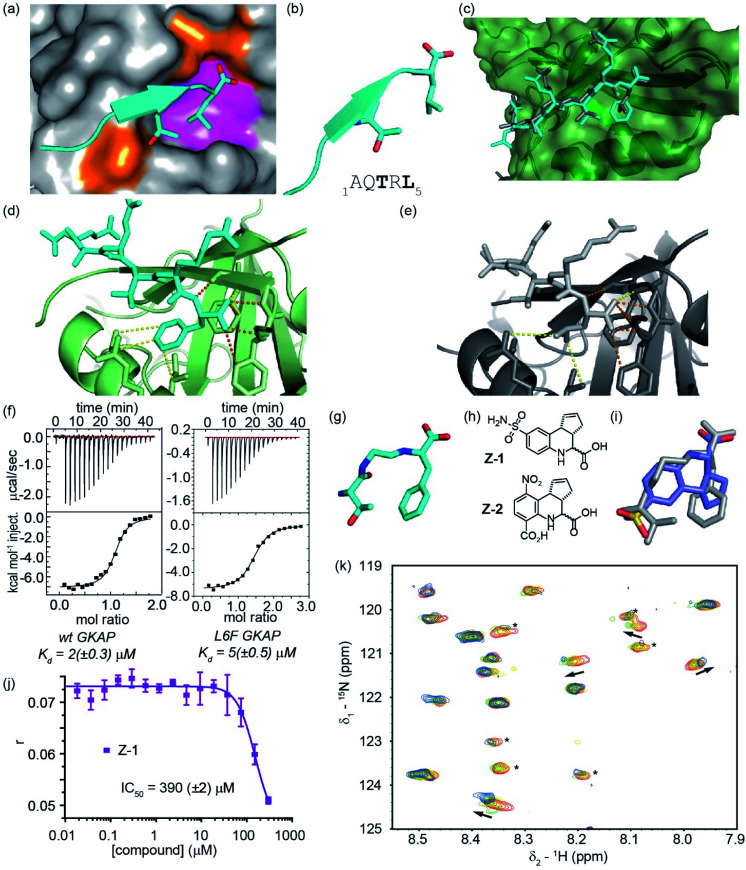
Development of a GKAP query for inhibitor screening against the GKAP/SHANK1-PDZ interaction; (a) close-up of the GKAP/SHANK1 PDZ interaction structure (PDB ID: 1Q3P),^43^ GKAP (cyan), with key side chains Thr3 and Leu5 highlighted, interacts with SHANK1 PDZ domain mainly through polar contacts (orange area); however, hydrophobic effects play a significant role in binding (magenta area); (b) cartoon representation of the GKAP_1–5_ peptide with key side chains Thr3 and Leu5 highlighted together with the primary sequence (below); (c) X-ray crystal structure of Ac-Glu-Ala-Gln-Thr-Arg-Phe peptide (L6F) bound to SHANK1-PDZ (PDB ID: 7A00) illustrating good correspondence with the position of key recognition groups observed for the wild-type sequence (PDB ID: 1Q3P); (d) close up of the interactions between the C-terminus of the L6F GKAP peptide and SHANK1 PDZ (H-bonds orange, other contacts yellow dashed lines); (e) close up of the interactions between the C-terminus of the wt GKAP peptide and SHANK1 PDZ; (f) binding of wild type (left) and L6F (right) GKAP peptide to SHANK1, monitored by ITC (25 °C in 20 mM Tris, 150 mM NaCl, pH 7.5, heats of peptide dilution were subtracted from each measurement raw data) with data analysed using Microcal Origin 8 and fitted to a one-binding site model; (g) GKAP query whereby the Thr3 side chain is retained together with a Phe *in lieu* of a Leu side chain alongside key backbone donor (NH) and acceptor (CO) groups; (h) structure of the hit compounds **Z-1** and **Z-2** identified from single point screening workflow using the query in panel (g); (i) overlay of the compound **Z-1** and the query (grey); (j) fluorescence anisotropy competition assay for compound **Z-1** (FITC-GKAP 50 nM, SHANK1-PDZ 1 μM, pH 7.4, 20 mM Tris, 150 mM NaCl, 0.01% Triton-X-100 buffer); (k) expansion of the ^1^H–^15^N HSQC spectra of ^15^N labelled SHANK1 in the absence (red) and presence of compound **Z-1** (compound : protein molar ratio 1 : 1 yellow, 2 : 1 green, 4 : 1 blue). Peaks indicated with asterisk undergo significant changes in intensity upon binding (SHANK1-PDZ 50 μM, pH 7.4, 5 mM Tris, 100 mM NaCl buffer).

**Fig. 4:**
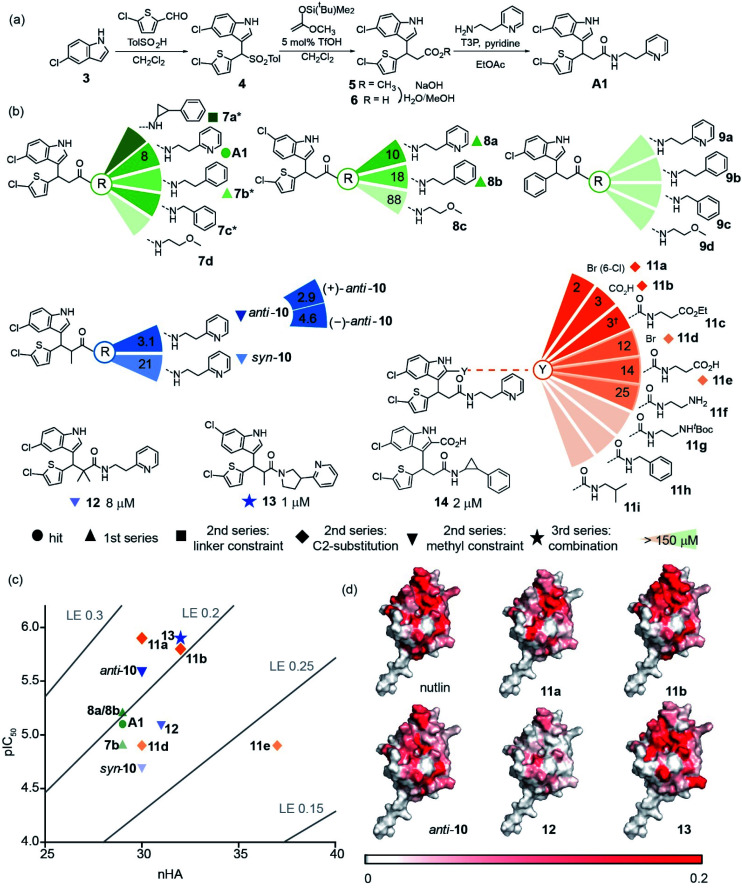
Development of p53/*h*DM2 inhibitors, (a) synthetic route to hit **A1**; (b) selected chemical structures and associated IC_50_ values (fluorescence anisotropy competition: 150 nM *h*DM2 and p53_15–31_Flu, 40 mM phosphate, pH 7.4, 200 mM NaCl and 0.02 mg ml^−1^ BSA) of developed inhibitors (increasing colour intensity of the wedges denotes increased potency, *reduced anisotropy in the assay attributed to solubility †non-specific interactions observed); (c) ligand efficiency plot for selected inhibitors shows exploration of the chemical space starting from selected hit **A1** (green sphere). Coloured shapes are assigned to describe inhibitors from the first (triangle), second (square, diamond and inverse triangle) and third series (star). (d) Mapping of chemical shift perturbations in *h*DM2 for selected inhibitors (750 MHz, 100 mM phosphate, pH 7.4, 2.5% glycerol, 1 mM DTT, increasing concentration of compound was titrated into 50 μM *h*DM2 and CSP wasdetermined at 1 : 2 protein : compound ratio). Colour variation is associated with a chemical shift perturbation from 0 ppm (white) to 0.2 ppm (red).

### Application of the discovery workflow to GKAP/SHANK1-PDZ

Subsequently, we applied the same workflow to the GKAP/SHANK1-PDZ interaction (Fig. 3). The GKAP PDZ-binding motif (Ac-Glu-Ala-Gln-Thr-Arg-Leu-CO_2_H), henceforth referred to as GKAP contains the known Type-I PDZ recognition motif:^54^ Thr-Xxx-Leu, with the C-terminal carboxylate making three essential hydrogen bonds with backbone amide hydrogen atoms in a loop on SHANK1 (Fig. 3a, orange area, PDB ID: 1Q3P).^39^ In our prior studies, computational modelling using BudeAlaScan^55,56^ and experimental analyses confirmed the Thr and Leu amino acids together with the terminal carboxylic acid as crucial for binding (Fig. 3b).^55,56^ Although SHANK1 has a defined hydrophobic cavity in which the Leu side chain of GKAP is accommodated (Fig. 3a, magenta area), it represents a significantly more challenging target for small-molecule mimicry than p53/*h*DM2. As a β-strand mediated PPI, the GKAP/SHANK1 interface relies on both side-chain contacts and an extensive hydrogen bond network that plays an active role in molecular recognition (Fig. 3a, d and e, H-bonds are shown as orange dashed lines); these features are not readily reproduced by typical (often hydrophobic) small molecule scaffolds. Thus, for query generation, the key hot residues of GKAP together with backbone heteroatoms were used. However, when our workflow was implemented with a query bearing these features, we identified only flexible peptide-based inhibitor candidates (not shown).

We reasoned that the C-terminal leucine, with more side-chain degrees of freedom, might have contributed to the moderate results in our *in silico* structure similarity analyses. The plasticity of PDZ domains allows the accommodation of various hydrophobic side chains at the C-terminus of the peptide; for SHANK1, Leu dominates for C-terminal carboxylates, however Phe has been observed to dominate for non-C-terminal sequences.^57,58^ Therefore, we considered Phe, with its more rigid hydrophobic amino acid sidechain, as a suitable surrogate in this position of the query. Gratifyingly, preparation of the Ac-Glu-Ala-Gln-Thr-Arg-Phe-CO_2_H peptide and assessment by isothermal titration calorimetry confirmed the Phe variant peptide to be tolerated (*K*_d_ = 5 (±0.5) μM compared with *K*_d_ = 2 (±0.3) μM for the GKAP sequence) and to act as an effective inhibitor (Fig. 3f and S11† for fluorescence competition anisotropy assay data using FAM-Ahx-Glu-Ala-Gln-Thr-Arg-Leu-CO_2_H as tracer). We obtained a peptide/protein co-crystal structure confirming the Phe to be a viable substitution for the C-terminal Leu (PDB ID: 7A00). The structure (Table S1† for Data collection and refinement statistics) reveals that the L6F variant peptide binds in the PDZ-binding site and reproduces many of the key recognition features and similar orientation of side chains, albeit with subtle differences (Fig. 3c–e).

Application of the *in silico* screening workflow to a modified query with the Phe substitution (Fig. 3g) identified a number of small-molecule candidate inhibitors (*e.g.*Fig. 3h, i and S12†). These candidates were screened in a fluorescence anisotropy competition assay at 30 and 300 μM following a similar approach to that pursued for the p53/*h*DM2 interaction, although a smaller number of less potent hits were obtained. Compound **Z-1** had an IC_50_ ∼ 300 μM in a fluorescence anisotropy competition assay (Fig. 3j). The moderate potency is deceiving; the low molecular weight (294 Da) and promising ligand efficiency (LE: 0.24) render it a promising starting point for subsequent optimization. ^1^H–^15^N HSQC perturbation shifts indicated interaction of the ligand with the SHANK1-PDZ domain (Fig. 3k and S13†), however in these analyses the absence of assignments prevented mapping of a putative binding site. Compound **Z-1** has significant similarity to a recently-reported small-molecule ligand for SHANK3: compound **Z-2**.^59^ We synthesized and tested compound **Z-2** in the fluorescence anisotropy competition and ^1^H–^15^N HSQC assays (see ESI†); these experiments confirmed that compound **Z-2** also acts as a GKAP/SHANK1-PDZ inhibitor (IC_50_ ∼ 110 μM in competition assay, see ESI Fig. S14†).

### Query-guided hit identification provides useful starting points for PPI inhibitor elaboration

**Fig. 5:**
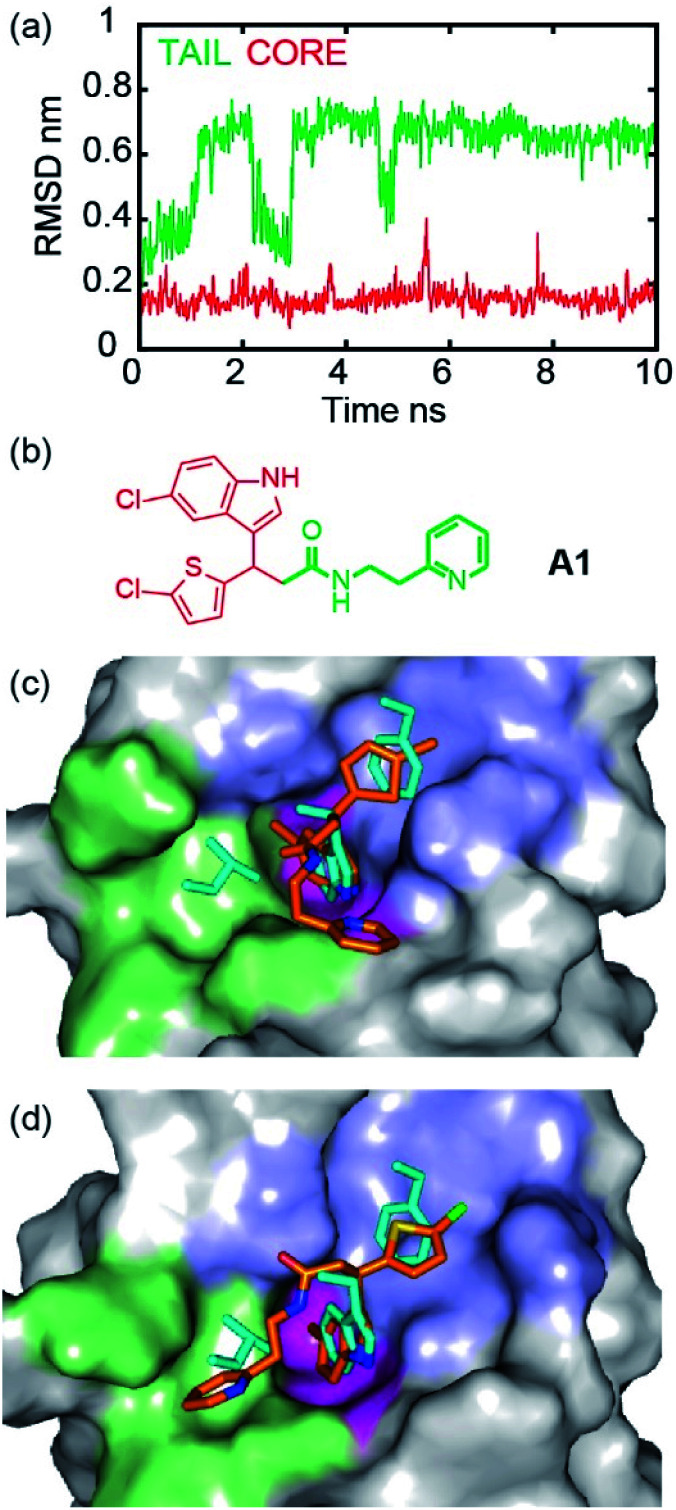
Molecular dynamics simulation of hit compound **A1** indicates coexistence of two conformations at the *h*DM2 binding site. (a) Root mean square deviations during a 10 nanoseconds molecular dynamics simulation of **A1**/*h*DM2. Red represents the core and green represents the tail. (b) Chemical structure of **A1** highlighting core and tail. (c) MD snapshot at 0 ns shows the folded conformation, which occupies only two of the three hot-spots, (d) MD snapshot at 8 ns shows the extended conformation which occupies the three hot-spots. p53 hot residue side chains are shown in cyan, Phe19, Trp23 and Leu26 pockets of *h*DM2 are highlighted in violet, magenta and green, respectively.

To illustrate the application of our approach to the identification of useful starting ligands for further optimization, we carried out structure–activity relationship analysis on one of the p53/hDM2 inhibitor series. After a synthetic route was established (Fig. 4a and Schemes S1–S3†), the original hit **A1** was resynthesized together with a series of derivatives (Fig. 4b for selected compounds). For the first series, full competition anisotropy experiments and NMR titrations (Fig. S15 and Table S2† for competition data and Fig. S16† for representative HSQC spectra) were performed for these (racemic) compounds, revealing compounds **A1**, **8a** and **8b** to be the most potent in the series (Fig. 4b). The loss of activity upon exchange of the 5-chloro-2-thiophene ring for a benzene ring (compounds **9a–9d**) and the hydrophobic amide linker length (compound **7c**) point to a key role of these groups as determinants of inhibition. Indole ring modifications and pyridine-to-benzene substitution were tolerated; however, an aromatic group in this position was necessary (analogues **7b** and **8b***versus* analogues **7d** and **8c**). Although we screened multiple crystallisation conditions, crystals of the **A1**/*h*DM2 complex were not obtained. Therefore, to rationalise the initial SAR and further investigate the binding mode of **A1**, we carried out molecular dynamics (MD) simulations (Fig. 5). The root mean square deviations (RMSD) of the atomic positions of the ligand over a 10 nanosecond simulation of the *R*-**A1**/*h*DM2 complex revealed important features. The indole and thiophene rings of *R*-**A1** formed a fixed core occupying the Trp23 and Phe19 pockets, respectively. In contrast, the (pyridin-2-yl)ethyl moiety appeared to flip between two different positions, as a consequence of rotation about the CO–Cα carbon bond and the inherent flexibility of the ethyl linker (Fig. 5c and d). Interestingly, only one of those positions occupied the shallower Leu26 pocket, which corresponds to an extended conformation of the inhibitor. This behaviour was replicated in a longer 200 ns MD simulation (Fig. S17a and Movie R-A1†). For the *S*-enantiomer the initial docked poses fit less well to the design hypothesis; for one pose the thiophene was placed in the W23 site whilst the other pose placed the indole in the W23 site, however, in both simulations the remaining arms were more dynamic indicating a poor correlation with the design hypothesis (Fig. S17b, c, Movies S-A1-I and II†). Given the extended conformation of *R*-**A1** was shown to best overlay with the three p53 hot-spot residues, we assumed it to be the most active conformation. Therefore, we developed a second series of inhibitors focusing on stabilisation of the extended conformation.

For the second series, we introduced modifications at three different positions of the hit structure **A1**: methyl substituents on the α carbon of the CO–Cα bond, cyclic constraints at the ethyl linker moiety and halogen, and, carboxylic acid or amide substituents at the C2-indole position (Fig. 4b, S15, S16 and Table S2†). These modifications were anticipated to increase steric congestion adjacent to the CO–Cα bond, restricting the accessible conformations in favour of the extended conformation with concomitant improvement in inhibitory potency (hypotheses supported by MD simulations Fig. S18–S21,† see below). Compounds *anti*-**10**, **11a** and **11b** were identified as the most potent inhibitors from this second series (see Fig. S22 and S23† for *syn*/*anti* assignment). Although there is still a two- to five-fold difference in potency in comparison to Nutlin-3, the improved ligand efficiency (0.28 **11a** and 0.26 for *anti*-**10** and **11b***versus* 0.22 for Nutlin-3) corroborates their potential as hits for development of drug-like p53/*h*DM2 inhibitors. The potential for further development of this series is underscored by the fact that it is possible to increase potency without marked increase in heavy atom count. Restriction of the CO–Cα bond with a gem-dimethyl group in **12** had no effect upon inhibition, (IC_50_ ∼ 8 μM), whereas single methyl analogues resulted in a significant variation in inhibitory activity. Diastereomer *anti*-**10** inhibits the p53/*h*DM2 interaction with an IC_50_ five times smaller than *syn*-**10**. Resolution of the *anti*-**10** enantiomers by chiral HPLC (>99.0% ee, see Fig S24 and 25†) allowed us to test them separately, with both showing comparable potency (3.1 μM for *anti*-10, 4.6 μM for (−)-*anti*-**10** and 2.9 μM for (+)-*anti*-**10**). MD simulations were performed to explore behaviour of the 4 stereoisomers of **10** to determine which best fits the p53 binding site of *h*DM2. Although more complex behaviour was observed, the simulations validate the hypothesis that restriction of the conformational mobility improves inhibitor potency (Fig. S18 and S19, Movies RR-10, SS-10, RS-10 and SR-10†). Compound **7a** with a constrained ethyl linker appeared to have good inhibitory potency, however, although HSQC analyses were consistent with effective binding, a lower anisotropy was observed in the assay which we attribute to poor solubility. Nonetheless, one of the 8 stereoisomers was subjected to MD simulation; this was generated by adding the cyclopropyl ring to *R*-**A1** in the only position compatible with the bound conformation and the constraints of the binding site. Bound **7a** shows particularly low mobility (Fig. S20 and Movie 7a†) consistent with the design hypothesis.

Modifications at the C2-indole position also induced a notable increase in potency; derivatisation with a bromine in **11a** and a carboxylic acid in **11b**. For derivatives **11a** and **11d**, the significant difference in activity (1 μM *versus* 13 μM) was attributed to a regioisomer effect arising from the presence of both the bromine and chlorine substituents. In the absence of the bromine, the regioisomers **A1** and **8a** (8 μM *versus* 6 μM) elicited similar potencies, pointing to a potential synergy between the orientation of the indole in the Trp23 pocket and the orientation of the other two hot-spot mimicking groups. Inhibitor **11b** was also observed to have increased potency; MD simulations place the 5-chloroindole ring deep in the Trp23 pocket, projecting the C2-indole carboxylate toward the edge of the α2 helix of *h*DM2 (see Fig. S21 Movies R-11b and S-11b†).^27^ This would permit additional hydrogen bonds, although a water mediated interaction has been observed for C2-carboxylate indole substituted p53/hDM2 inhibitors.^60–62^ More significantly, the simulation on both *R*-**11b** and *S*-**11b** supports the notion that the C2-indole substitution impedes back folding of the pyridyl group to enforce an extended conformation (Fig. S21, Movies R-11b and S-11b†). Amides **11e** and **11f**, bearing terminal polar ionic groups, had moderate potency (13 μM and 25 μM, respectively), whereas amides **11g–11i**, with apolar substituents, were poorer inhibitors. These observations are consistent with the hypothesis^51^ that C-2 indole substituents promote a bioactive conformation, while directing one substituent into the Phe19 pocket the C2-indole substituent acts as a cap shielding the hydrophobic molecule from solvent. Finally, the combination of methyl substituents on the α carbon of the CO–Cα bond or C2-indole substitution and a constraint on the ethyl linker also resulted in potent compounds (**13**, IC_50_ = 1.2 (±0.4) μM and **14**, IC_50_ = 2.2 (±0.5) μM, Fig. 4b). We then performed ^1^H–^15^N HSQC experiments on these most potent inhibitors *anti*-**10**, **11a**, **11b** and **13** and mapped their chemical shift variations onto the *h*DM2 protein (Fig. 4d and Fig. S16†). In all cases, the major chemical shift variations included amino acids Leu54, Gly58 and Val93, while less pronounced shifts were observed for less potent compounds (*e.g.***12** see Fig. 4d). These amino acids are located around the three hot-spot pockets, thus the perturbations arising from binding of the small-molecules can be attributed to recognition of the hydrophobic p53 binding cleft of *h*DM2.

## Conclusions

In summary, we have used query-guided inhibitor discovery to identify small-molecule inhibitors of the p53/*h*DM2 and GKAP/SHANK1 interactions. For each interaction, a computational workflow that involved FastROCS matching to secondary structure queries identified candidate inhibitors. Experimental screening of subsets of these candidates identified multiple genuine inhibitors demonstrating the approach to be valid for improving hit rate. Characterization of the most-promising compounds using two orthogonal assays identified inhibitors for both targets with promising ligand efficiency, which bound at the anticipated binding site and could be further developed. For instance starting with 200 compounds for experimental screening against p53/*h*DM2 we obtained IC_50_ values for 37 compounds. This demonstrates the strong performance of the computational workflow considering that high-throughput or fragment screening typically require >1 million or 10^3^ compounds respectively to be screened to obtain similar numbers of hits to take forward. Although the approach is less likely to identify inhibitors that rely on induced conformational changes – indeed Nutlin-like compounds (which are known to have subtly different interactions with the dynamic hDM2 surface when compared to p53)^7^ are in the AstraZeneca compound collection, but were not scored in the top 1000 hits identified for experimental screening – this represents an excellent hit rate. Whilst proteomimetics have been used to inhibit a range of intracellular α-helix-mediated PPIs, there remains a need to broaden the methodology to other targets and use the conceptual framework to identify small molecules that can be developed further.^63–66^ This study also emphasizes that identification of non-α-helix mediated PPI inhibitors *e.g.* β-strand-mediated PPIs is more difficult, although the identification of inhibitors for a strand-mediated interaction with a PDZ domain, albeit with a significantly lower hit-rate represents the first steps towards such a goal. Similarly, pharmacophore-based virtual screening approaches whereby key hot-spot residues have been used as an anchor^67^ have also recently been introduced, but to date brought to bear only on targets with a dominant hot residue. Taken together, our new approach harnesses the best of both worlds to identify and prioritise small-molecules that mimic diverse secondary structures and inhibit PPIs broadening the proteomimetic concept beyond α-helix mediated PPIs and extending it to genuine small-molecule ligands.

## Author contributions

S. C., F. H. and T. J. contributed equally to this work. D. N. W., R. B. S., T. A. E., D. M. A., A. N. and A. J. W., conceived and designed the research program, S. C., F. H., T. J., A. A. I., G. J. B., D. K. S., Z. H. and K. H. designed studies and performed research. The manuscript was written by S. C., A. N and A. J. W. with contributions from all authors.

## Conflicts of interest

There are no conflicts to declare.

## Supplementary Material

SC-012-D1SC00023C-s001

SC-012-D1SC00023C-s002

SC-012-D1SC00023C-s003

SC-012-D1SC00023C-s004

SC-012-D1SC00023C-s005

SC-012-D1SC00023C-s006

SC-012-D1SC00023C-s007

SC-012-D1SC00023C-s008

SC-012-D1SC00023C-s009

SC-012-D1SC00023C-s010

SC-012-D1SC00023C-s011

SC-012-D1SC00023C-s012
