# Supplementary material for: Query-guided protein–protein interaction inhibitor discovery
Source: Chem Sci. 2021 Mar 2;12(13):4753–62. doi: 10.1039/d1sc00023c (PMC8179539; doi:10.1039/d1sc00023c)
Supplement: SC-012-D1SC00023C-s006 [file SC-012-D1SC00023C-s006.pdf]

Electronic Supplementary Material (ESI) for Chemical Science.
This journal is © The Royal Society of Chemistry 2021

Query-guided Protein-Protein Interaction Inhibitor Discovery

Supporting information

Sergio Celis,[a,b,†] Fruzsina Hobor,[a,c,†] Thomas James,[a,b,†] Gail J. Bartlett,[d] Amaurys A. Ibarra,[e] Deborah K. Shoemark,[e,f] Zsofia Hegedus,[a,b] Kristina Hetherington,[a,b] Derek N. Woolfson,[d,e,f] Richard B. Sessions,[e,f] Thomas A. Edwards,[a,c] David M. Andrews,*[g] Adam Nelson,*[a,b] Andrew J. Wilson* [a,b]

[a] Astbury Centre for Structural Molecular Biology, University of Leeds, Woodhouse Lane, Leeds LS2 9JT, UK
[b] School of Chemistry, University of Leeds, Woodhouse Lane, Leeds LS2 9JT, UK
[c] School of Molecular and Cellular Biology, University of Leeds, Woodhouse Lane, Leeds LS2 9JT, UK
[d] School of Chemistry, University of Bristol, Cantock's Close, Bristol BS8 1TS, UK
[e] School of Biochemistry, University of Bristol, Medical Sciences Building, University Walk, Bristol BS8 1TD, UK
[f] BrisSynBio, University of Bristol, Life Sciences Building, Tyndall Avenue, Bristol BS8 1TQ, UK
[g].Early Oncology, AstraZeneca, Hodgkin Building, Chesterford Research Campus, Saffron Walden, Cambridge, CB10 1XL, UK

1. Supplementary figures and tables 2
2. Further Discussion of molecular dynamics simulations 19
3. Determination of the relative configuration of diastereomers 27
4. Chiral separation of enantiomers (+)-*anti*-10 and (–)-*anti*-10 29
5. Supplementary methods 32
5.1. Biophysical methods 32
5.1.1. Screening of AZ compounds 32
5.1.2. IC_{50} determination – fluorescence anisotropy competition assays 33
5.1.3. NMR spectroscopy 33
5.1.4. Crystallography 34
5.1.5. Protein expression and purification 34
5.1.6. Isothermal titration calorimetry experiments 34
5.2. Computational methods 35
5.3. Molecular Dynamics Methods 38
5.4. Synthetic methods 39
5.1. Peptides 40
5.2. General synthetic procedures 40
5.2.1. Procedure A: multicomponent reactions 40
5.2.2. Procedure B: methyl carboxylate ester formation 41
5.2.3. Procedure C: ester hydrolysis 42
5.2.4. Procedure D: bromination of the C2-H position of an indole 42
5.2.5. Procedure E: amide bond formation 42
5.2.6. Procedure F: aza-Diels-Alder cycloaddition 43
5.3. Synthetic schemes 44
6. Synthesis and characterisation 46
7. Peptide Characterization data 93
8. Protein Characterization 96
9. Small Molecule Spectra 97
10. Bibliography 162

1. Supplementary figures and tables

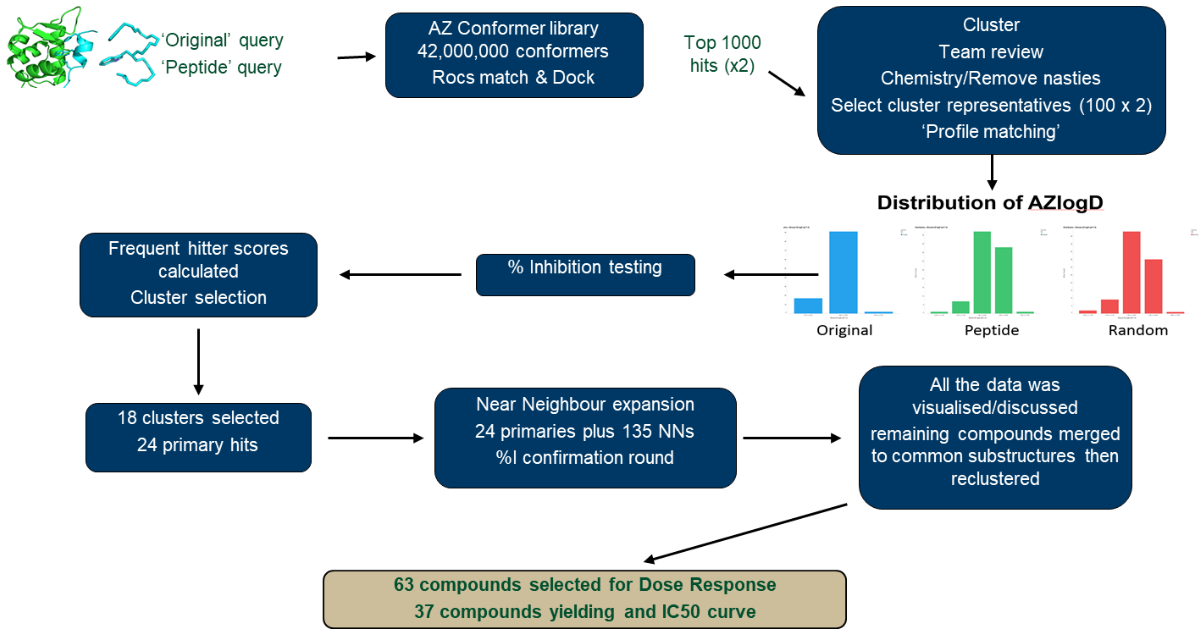

Figure S1. *Detailed summary of steps in workflow (shown in Figure 1 of the manuscript) used to identify inhibitors with a query guided approach (numbers are specific to p53/hDM2, with similar approach used for GKAP/SHANK 1 PDZ): Following hot-spot identification and query generation, a virtual library of small molecules is shape-matched against the query, and promising compounds docked against the target protein. Candidate inhibitors are then subjected to experimental screening and characterisation, enabling selection of hits for: (i) clustering, near neighbour expansion and further screening with reiteration of shape-matching/docking, % inhibition as necessary or (ii) further development/optimization. Clustering was based on Daylight Fingerprint and Tanimoto Similarity. Near neighbours were selected using Tanimoto Similarity from the larger (scored and unscored) AstraZeneca screening database*

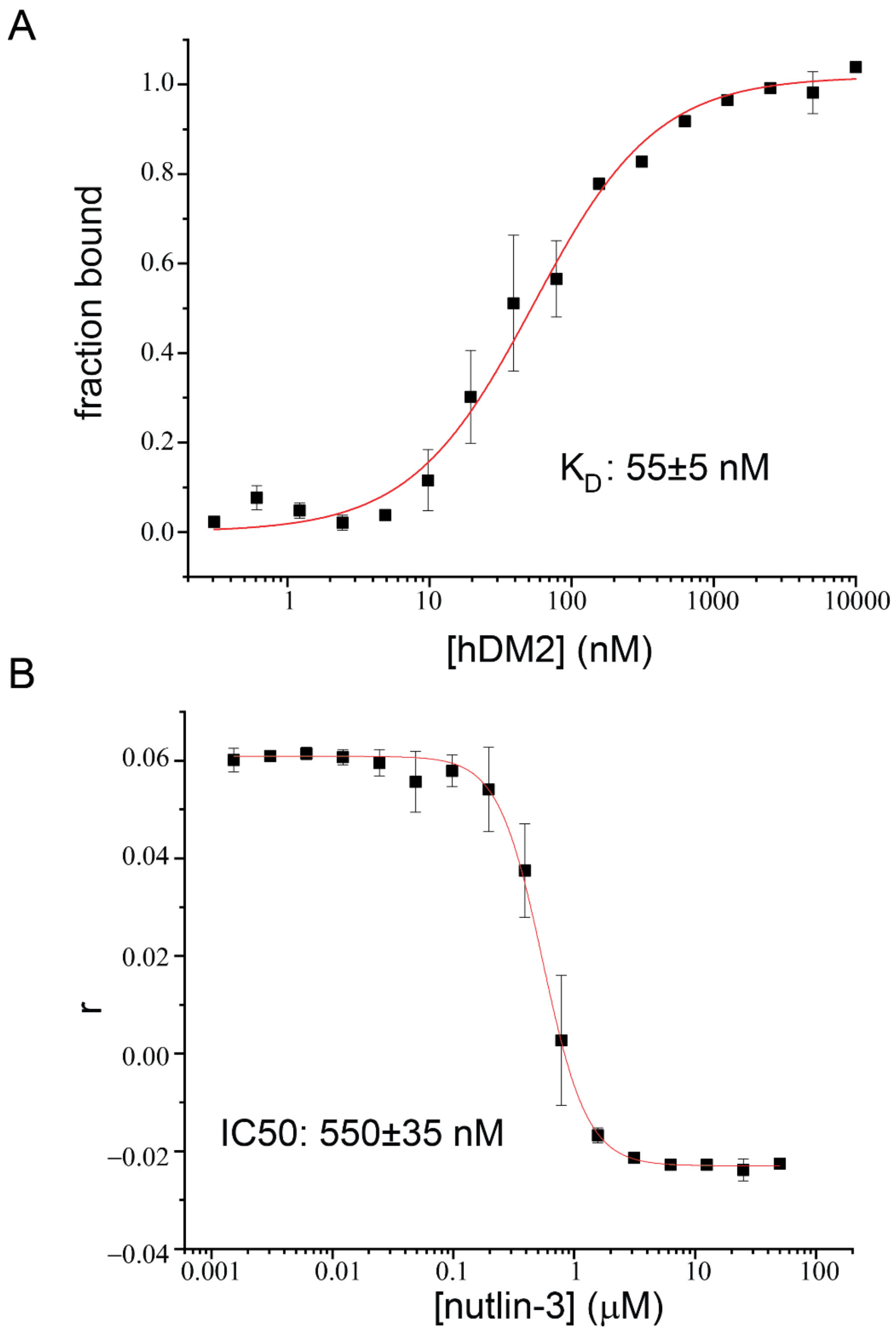

Figure S2. *(A) Binding of hDM2 17-125 to p53$_{15\text{-}31}$Flu (40 mM Phosphate, 200 mM NaCl, 0.02 mg/ml BSA, pH 7.4, Plate Reader II) monitored by fluorescence anisotropy, after 4 hours of incubation at room temperature; (B) Competition fluorescence anisotropy binding assay (40 mM Phosphate, 200 mM NaCl, 0.02mg/ml BSA, pH 7.4, Plate Reader II) to monitor the replacement of 25 nM p53$_{15\text{-}31}$Flu by Nutlin-3 in the presence of 150nM hDM2, after 4 hours of incubation at room temperature (bottom). Data was analysed using Origin.*

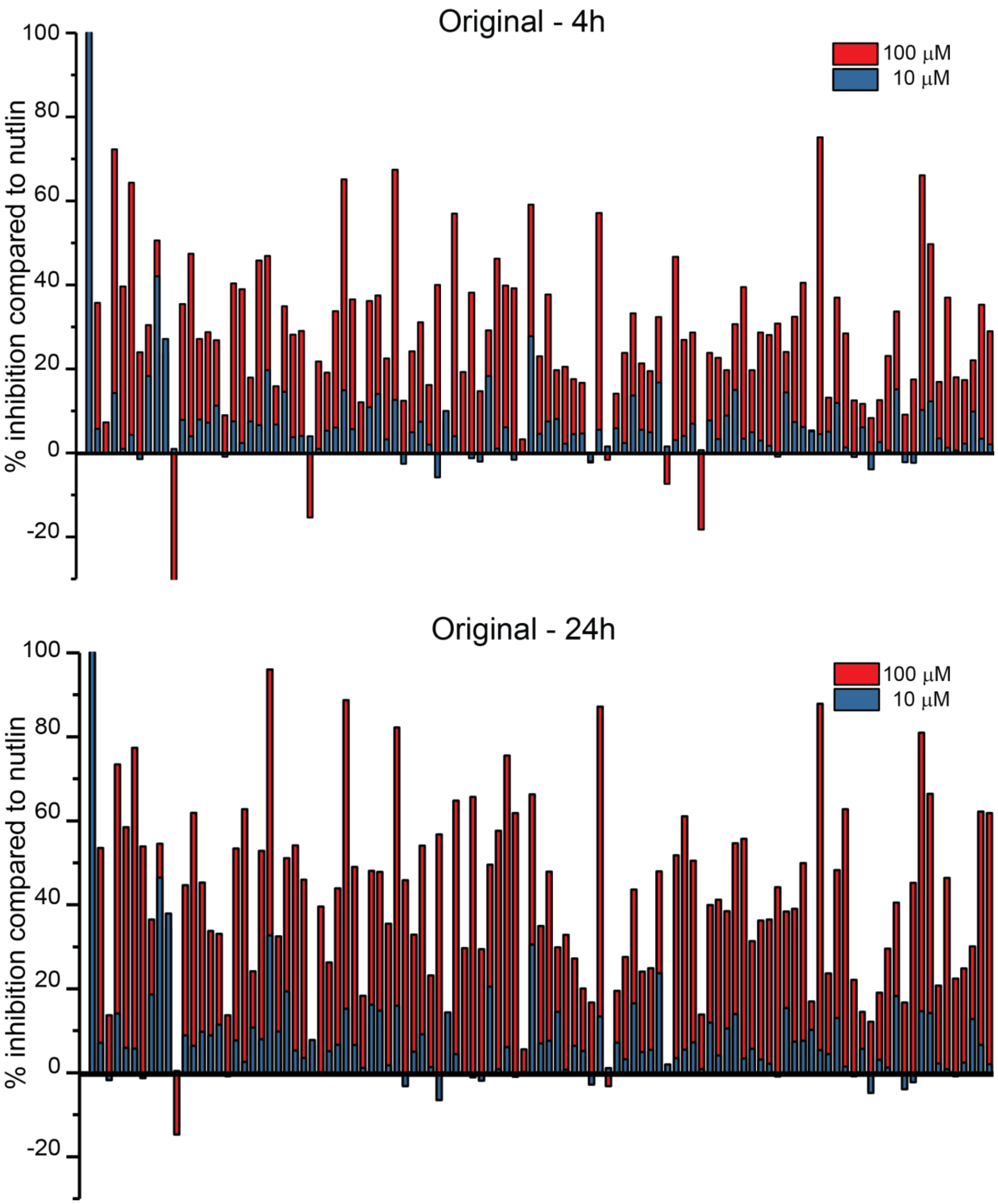

***Figure S3**. Percentage inhibition of compounds from the hydrocarbon query at 10 and 100μM, compared to Nutlin-3 determined after 4 and 24 hours of incubation (150nM hDM2, 25nM $p53_{15\text{-}31}$Flu, 40mM phosphate, pH 7.4, 200mM NaCl and 0.02 mg/ml BSA, Plate Reader I). Data was analysed in Excel and plotted in Origin.*

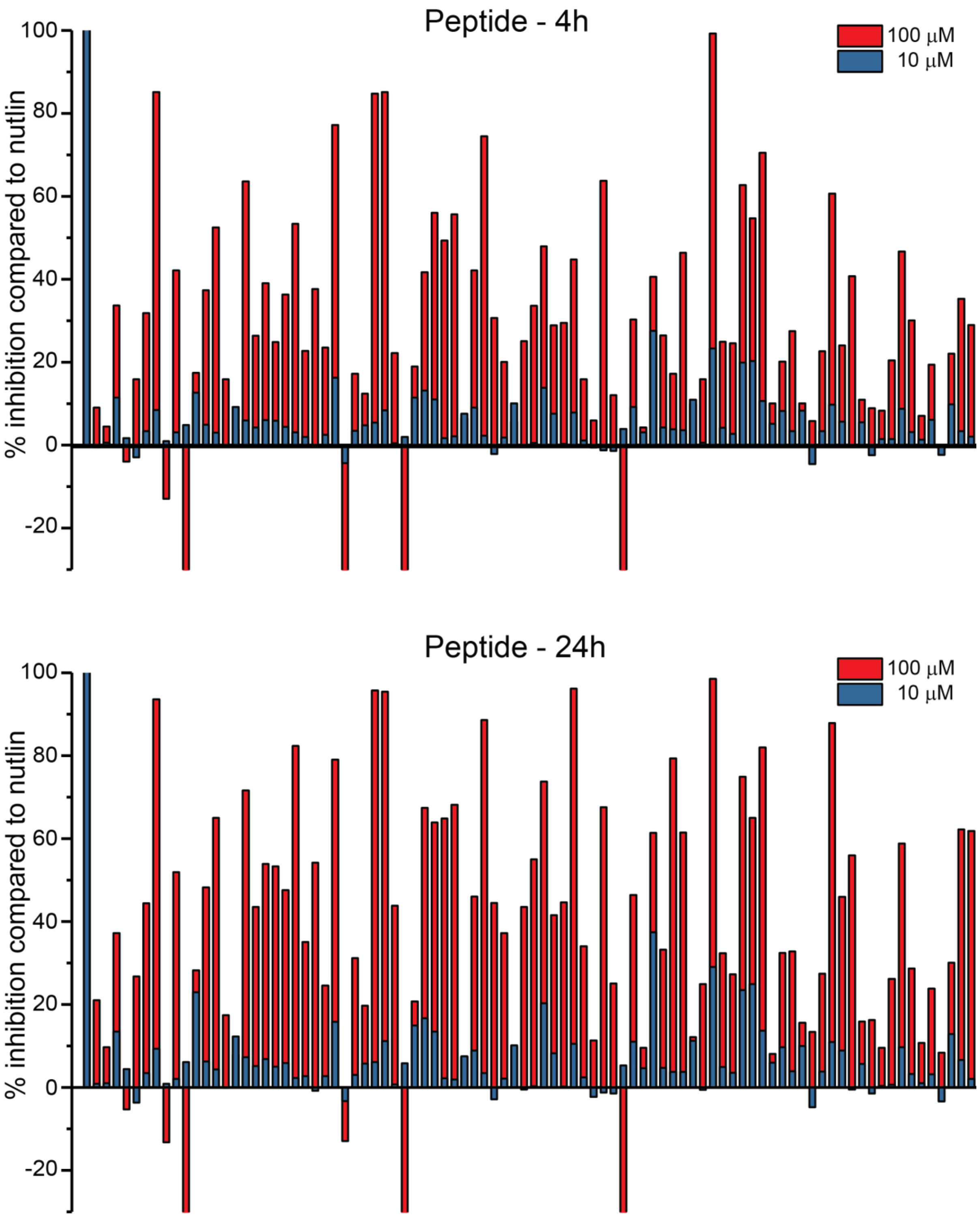

Figure S4. *Percentage inhibition of compounds from the peptide query at 10 and 100µM, compared to Nutlin-3 determined after 4 and 24 hours of incubation (150nM hDM2, 25nM p53*$_{15\text{-}31}$*Flu, 40mM phosphate, pH 7.4, 200mM NaCl and 0.02 mg/ml BSA, Plate Reader I). Data was analysed in Excel and plotted in Origin.*

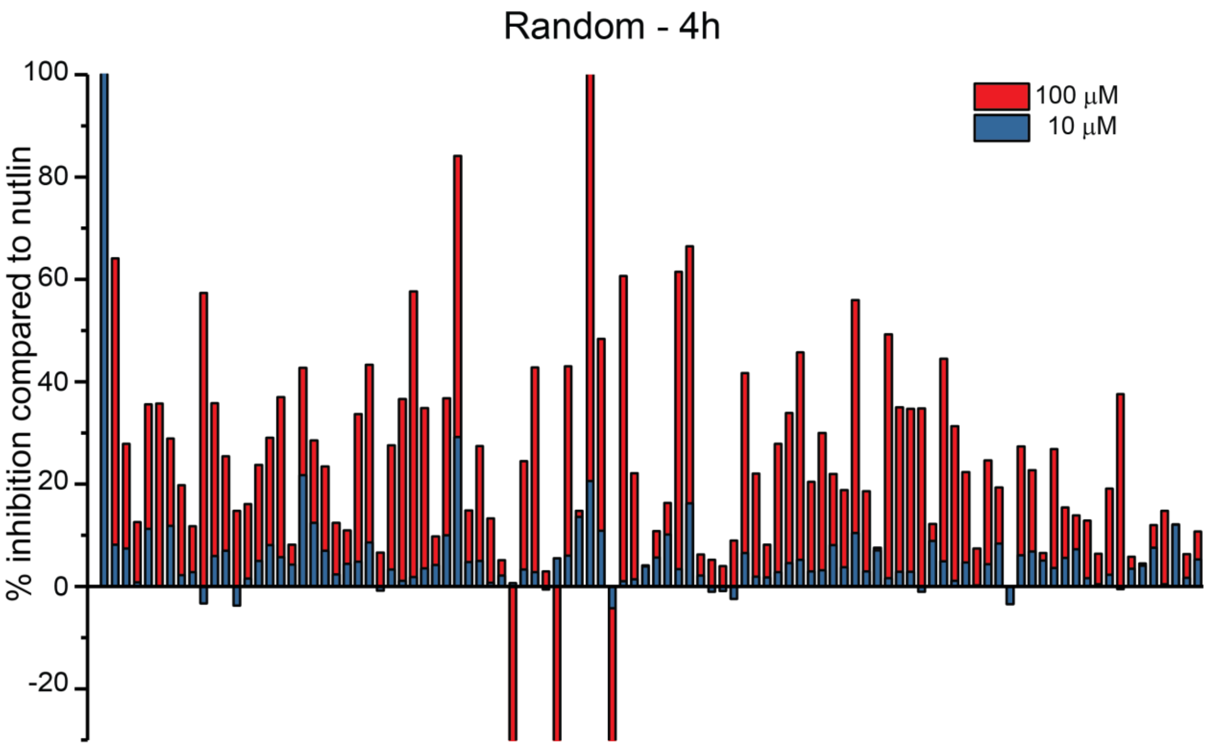

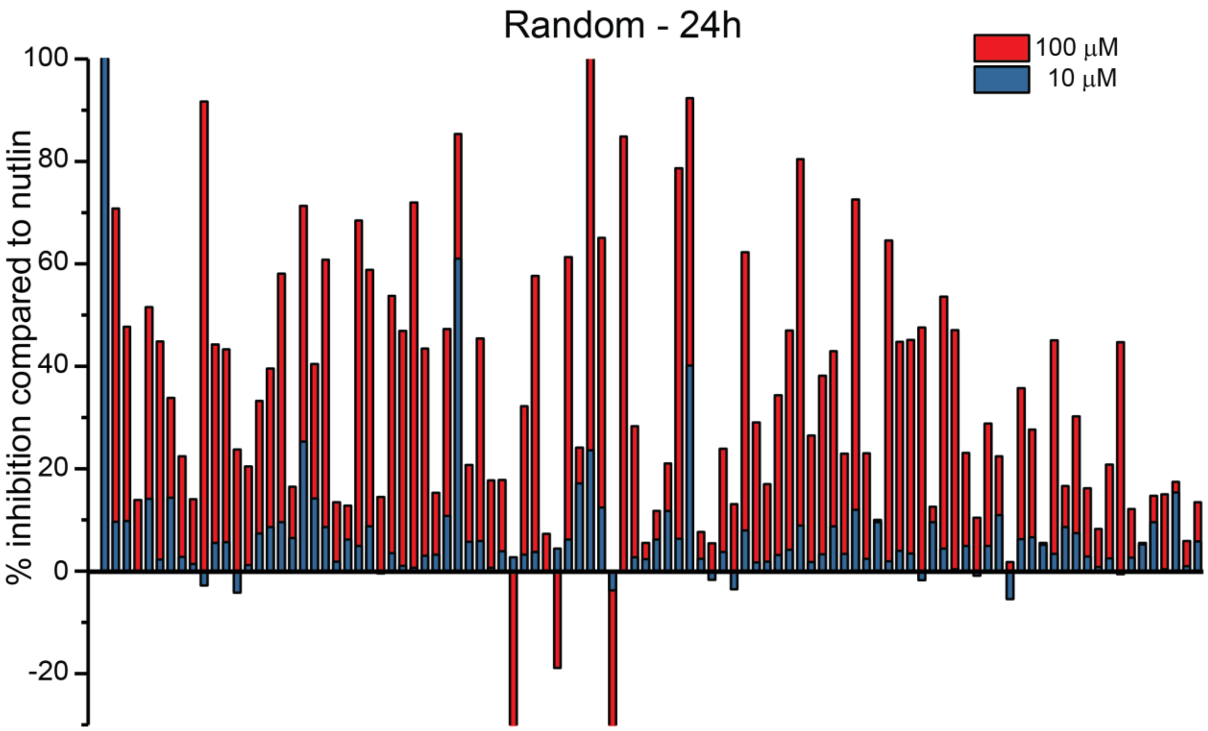

Figure S5. *Percentage inhibition of random compounds at 10 and 100μM, compared to Nutlin-3 determined after 4 and 24 hours of incubation (150nM hDM2, 25nM p53$_{15\text{-}31}$Flu, 40mM phosphate, pH 7.4, 200mM NaCl and 0.02 mg/ml BSA, Plate Reader I). Data was analysed in Excel and plotted in Origin.*

Of the one hundred randomly selected compounds selected for analyses a number inhibited the p53/hDM2 interaction (Fig. S5); hit-rates for the randomly selected compounds were comparable to those for the hydrocarbon and amide queries (Fig. S3-42). However, whilst a number were active at 100 μM, a greater proportion exhibited significantly reduced activity at 10 μM. Team discussions accounting for frequenter hitter status, synthetic accessibility concluded these would be undesirable for further development (not shown) and they were pursued no further.

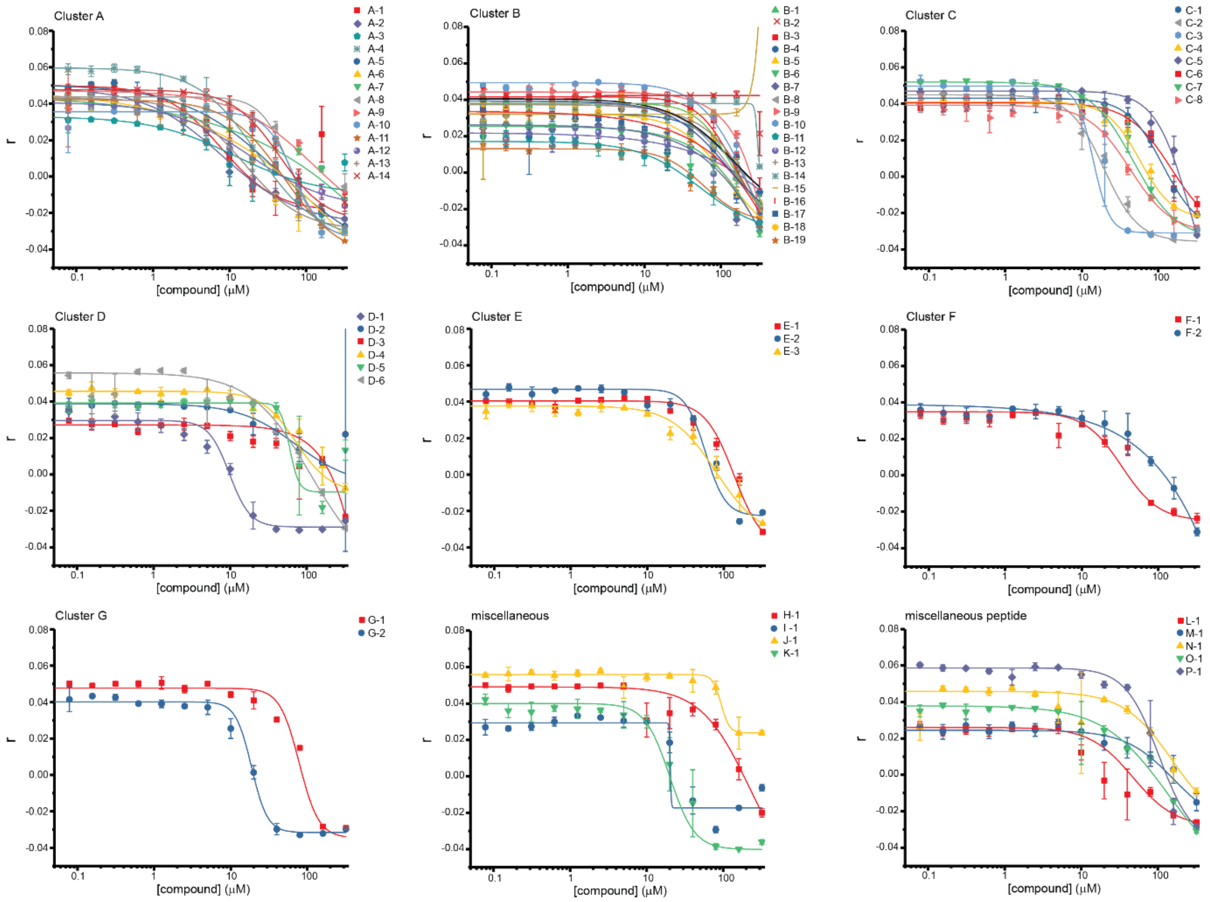

Figure S6. *Fluorescence anisotropy competition assays to determine IC_{50} values for selected compounds (150nM hDM2, 25nM $p53_{15\text{-}31}$Flu, 40mM phosphate, pH 7.4, 200mM NaCl and 0.02 mg/ml BSA, Plate Reader I). The data was analysed using Origin, the curves are grouped based on the clusters.*

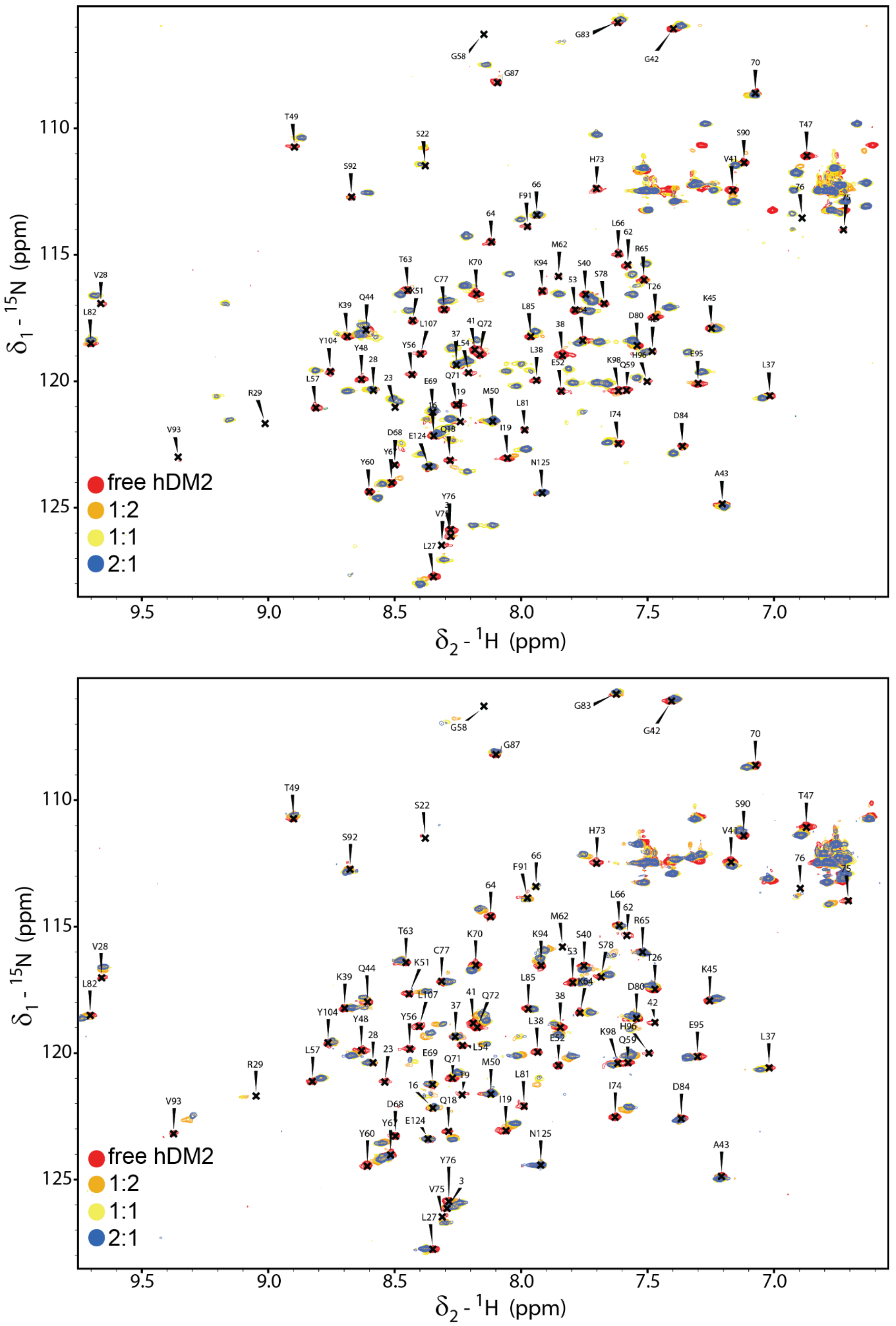

***Figure S7**. Chemical shift perturbation of hDM2 17-125 upon binding to Nutlin-3 (top) and A-1 (bottom) compounds. For NMR titrations (750 MHz, 100mM phosphate. pH 7.4, 2.5% glycerol, 1mM DTT) increasing amount of compound was titrated into 50μM hDM2. The NMR data was analysed using Sparky.*

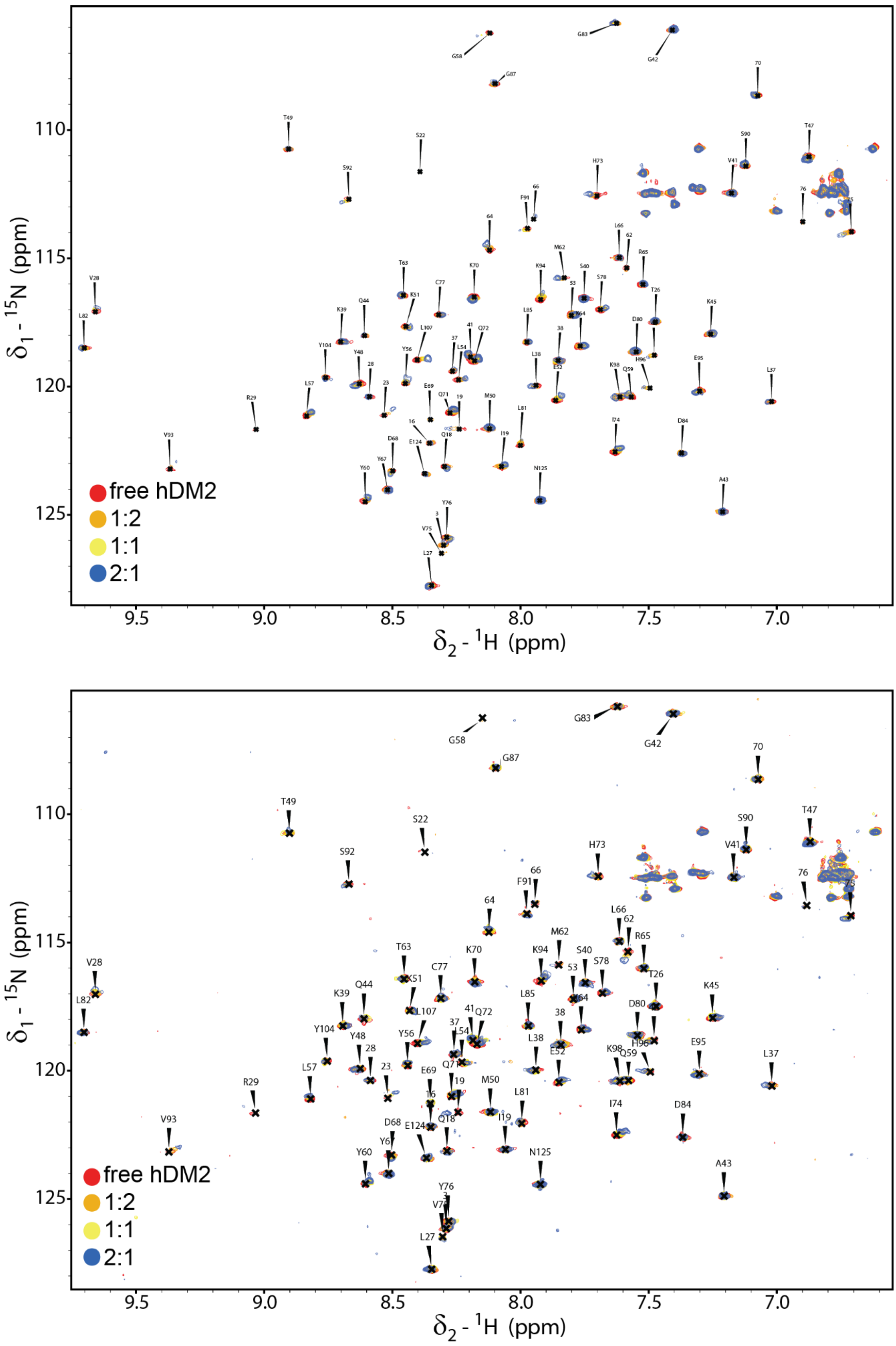

Figure S8. *Chemical shift perturbation of hDM2 17-125 upon binding to B-1 (top) and C-1 (bottom) compounds. For NMR titrations (750 MHz, 100mM phosphate, pH 7.4, 2.5% glycerol, 1mM DTT), increasing amount of compound was titrated into 50μM hDM2. The NMR data was analysed using Sparky.*

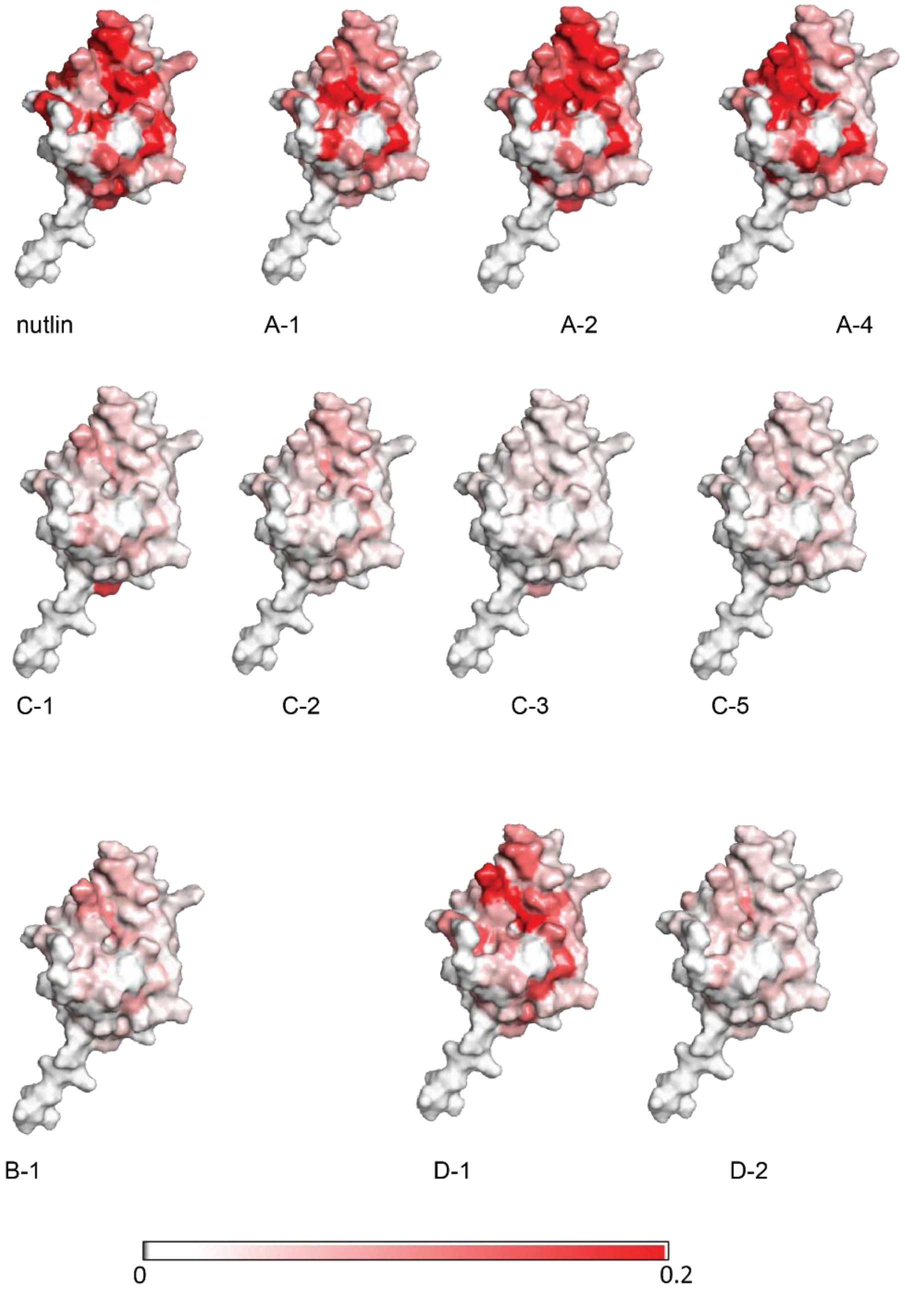

Figure S9. *Chemical shift perturbation upon binding to selected compounds mapped on the structure of hDM2 17-125. For NMR titrations (750 MHz, 100mM phosphate, pH 7.4, 2.5% glycerol, 1mM DTT), increasing amount of compound was titrated into 50μM hDM2. CSP was determined at 1:2 protein:compound ratio. The NMR data was analysed using Sparky, the heatmaps were generated in PyMol.*

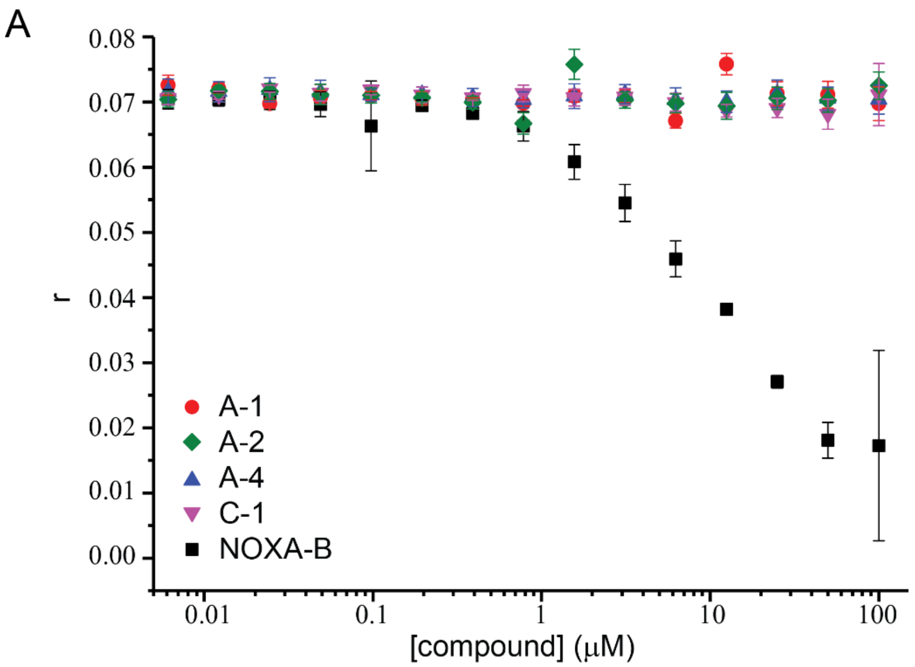

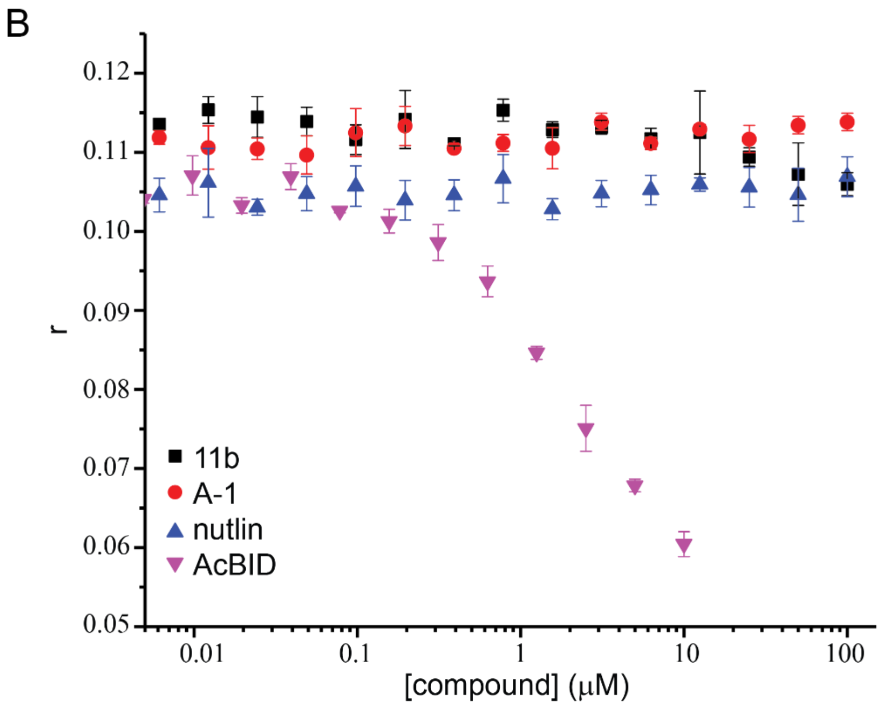

Figure S10. *Fluorescence anisotropy competition assays to show the selectivity of selected compounds from clusters A and C (A) compound 11b (B) in the presence of 200nM MCL-1 and 50nM FITC-Ahx-NOXA-B, (25mM Tris, pH 7.5, 150 NaCl, 10mg/ml BSA and 0.01% Triton-X, Plate Reader II). The data was analysed using Origin.*

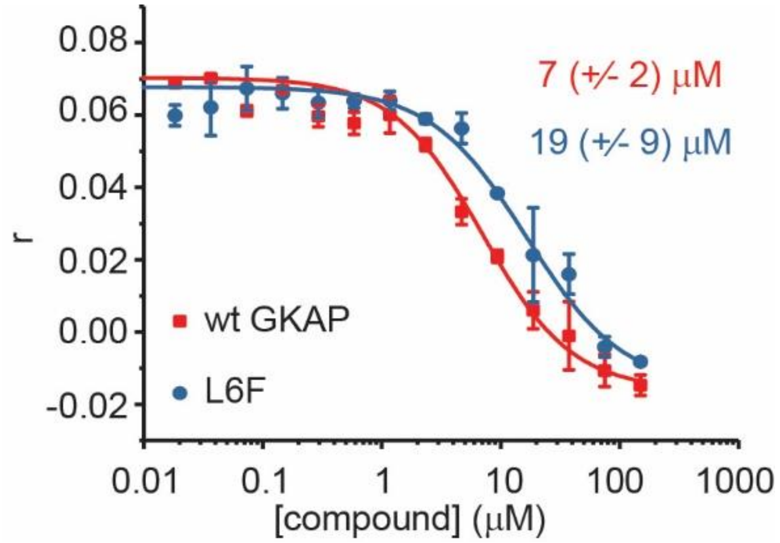

Figure S11. *Fluorescence anisotropy competition assay of variant Ac-Glu-Ala-Gln-Thr-Arg-Phe peptide (L6F) and wild-type GKAP (FITC-Ahx-GKAP 50 nM, SHANK1-PDZ 1 μM, pH 7.4, 20mM Tris, 150mM NaCl, 0.01% Triton-X-100 buffer, Plate Reader I);*

Supplementary Table S1

L6F-GKAP/Shank	
**Reservoir conditions**	0.1 M HEPES 7 PEG 400 40%
**Data Collection**	
X-ray source	DLS beamline i04
Data collected on	17th February 2019
Processed using	xia2 3dii (XDS, XSCALE)
oscillations	0.5
images collected	720
Space group	P 21 21 21
a, *b*, *c*, (Å)	44.18, 66.47, 88.58
α,β,γ (°)	90.00, 90.00, 90.00
Resolution	1.78 - 53.17 (1.78 - 1.81)
Observations	338235 (17068)
Unique reflections	25591 (1253)
R_{merge} (I)	0.074 (1.993)
R_{meas} (I)	0.077 (2.071)
R_{pim} (I)	0.021 (0.559)
CC 1/2	0.999 (0.595)
I/σ	16.6 (1.1)
Completeness	99.6 (99.7)
Redundancy	13.2 (13.6)
Protein molecules in au	2
**Rwork/Rfree**	0.2133/0.2486
No atoms	1944
Protein	1769
Ligand	6
Water	169
**Mean B factors (A)**	45.44
Protein	45.01
Ligand	67.94
Water	49.19
**R.m.s. deviations**	
Bond length (A)	0.006
Bond angles	0.73
**Ramachandran statistics**	
% favoured	100
% allowed	0
% outliers	0
Clashscore	3.11

Data collection and refinement statistics

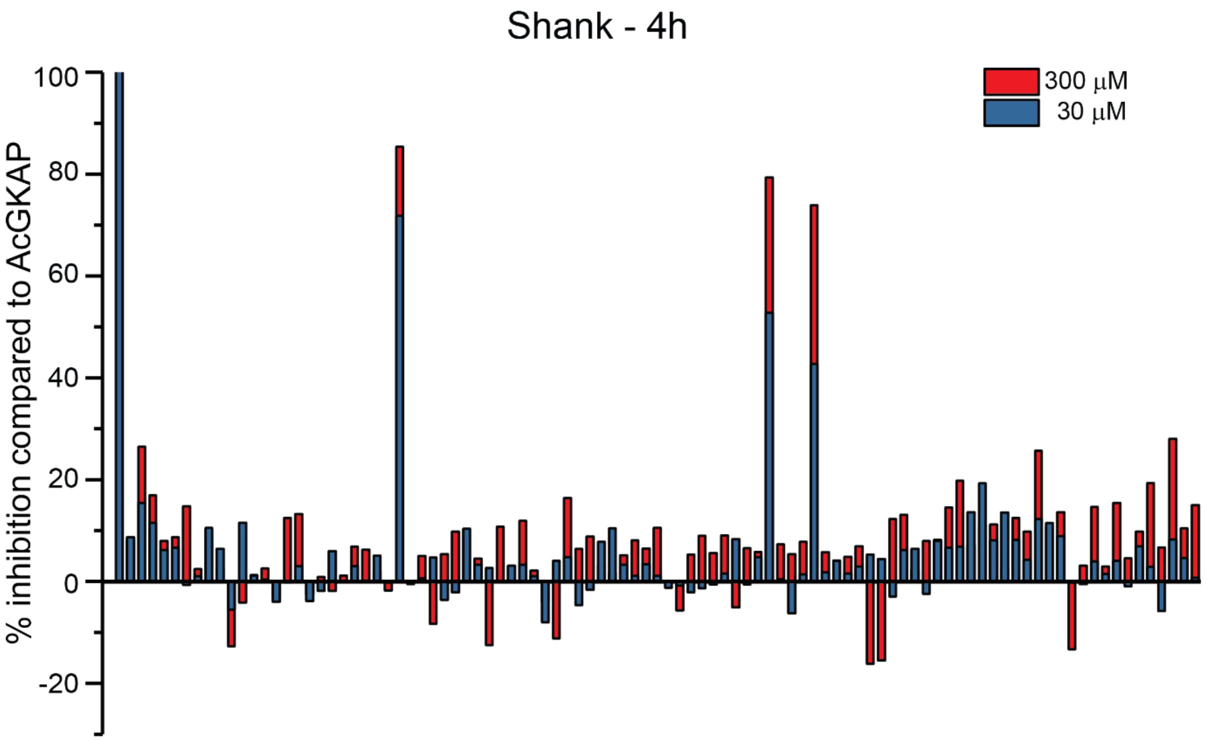

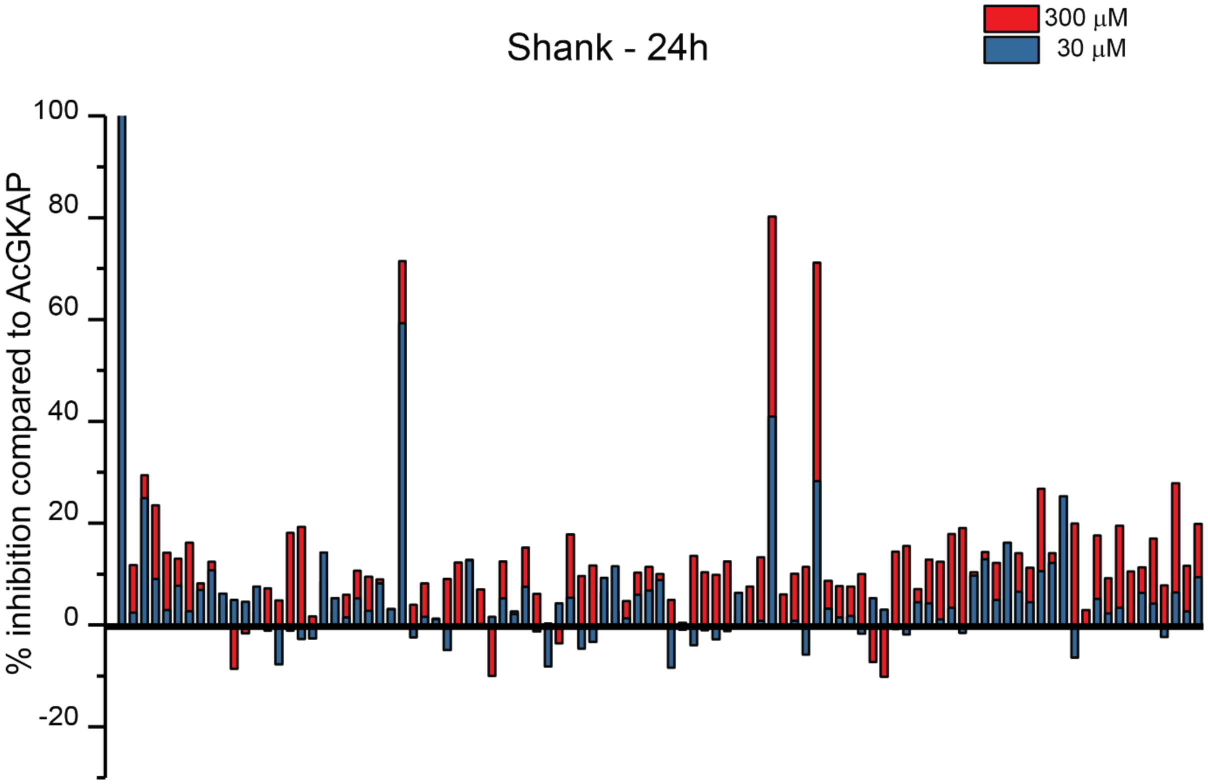

***Figure S12**. Percentage inhibition of compounds from the F-GKAP query at 30 and 300μM, compared to acetylated GKAP determined after 4 and 24 hours of incubation (FITC-Ahx-GKAP 50 nM, SHANK1-PDZ 1 μM, pH 7.4, 20mM Tris, 150mM NaCl, 0.01% Triton-X-100 buffer, Plate Reader I). Data was analysed in Excel and plotted in Origin.*

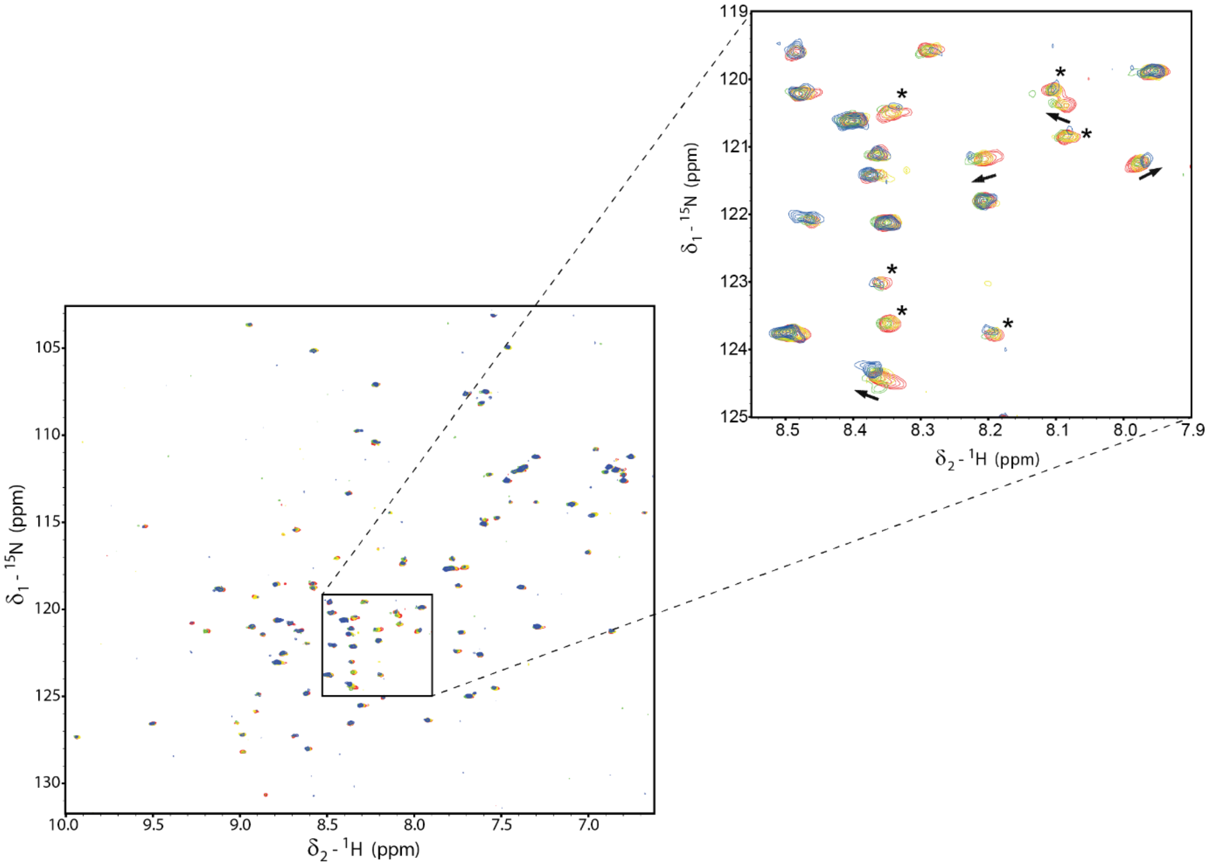

***Figure S13**. Chemical shift perturbation of SHANK-1 PDZ 656-762 upon binding to **Z-1**. For NMR titrations (750 MHz, 5 mM Tris, 100 mM NaCl, pH 7.4), increasing amount of compound was titrated into 50μM protein. The NMR data was analysed using Sparky.*

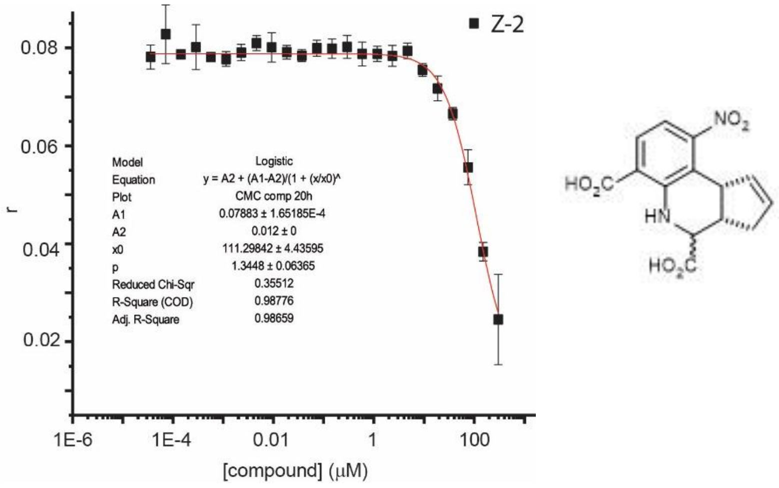

***Figure S14**. Fluorescence anisotropy competition assay for compound Z-**2** (FITC-GKAP 50 nM, SHANK1-PDZ 1 μM, pH 7.4, 20mM Tris, 150mM NaCl, 0.01% Triton-X-100 buffer, Plate Reader I);*

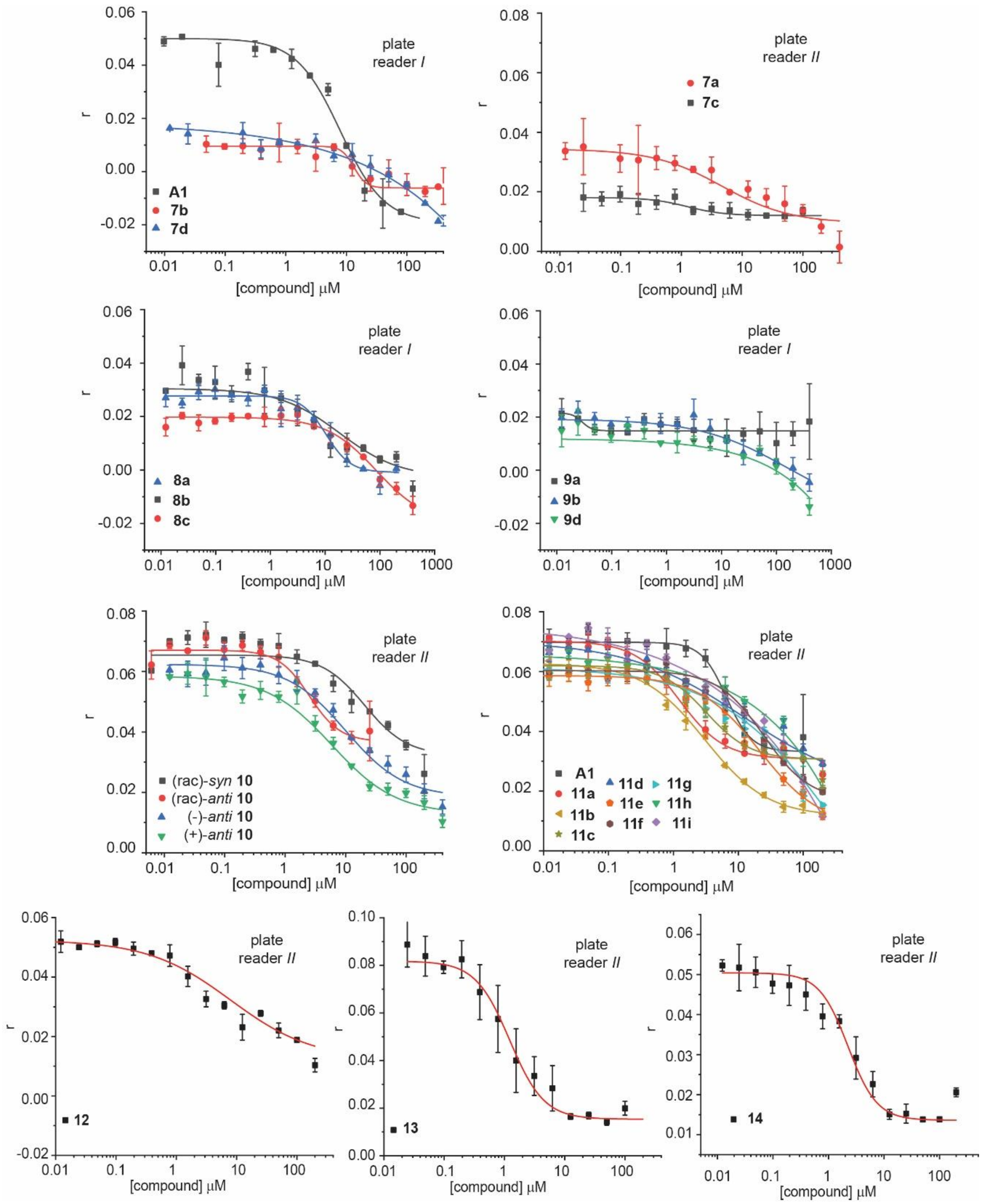

***Figure S15**. Fluorescence anisotropy competition assays to determine the IC_{50} values of selected compounds from SAR (150nM hDM2 and $p53_{15\text{-}31}$Flu, 40mM phosphate, pH 7.4, 200mM NaCl and 0.02 mg/ml BSA, Plate Reader II). The data was analysed using Origin.*

Supplementary Table S2. Summary of IC_{50} values for fluorescence anisotropy SAR competition assays

compound	IC_{50} (µM)	compound	IC_{50} (mM)
**A1**	7.9 ± 0.9	(rac)-*syn* **10**	21 ± 14
**7a***	1.2 ± 1*	(rac)-*anti* **10**	3.1 ± 0.4
**7b***	13 ± 2*	(-)-*anti* **10**	4.6 ± 0.6
**7c***	5 ± 2*	(+)-*anti* **10**	2.9 ± 0.4
**7d**	>200	**11a**	1.7 ± 0.2
**8a**	10 ± 2	**11b**	2.9 ± 0.2
**8b**	18 ± 11	**11c**	3.2 ± 0.7
**8c**	86 ± 29	**11d**	12 ± 9
**9a**	>200	**11e**	14 ± 2
**9b**	>200	**11f**	25 ± 4
**9c**	n.d.	**11g**	>200
**9d**	>200	**11h**	>200
		**11i**	>200
		**12**	8 ± 4
		**13**	1.2 ± 0.4
		**14**	2.2 ± 0.5

*a lower anisotropy was observed in these assays, which we attribute to solubility causing assay interference.

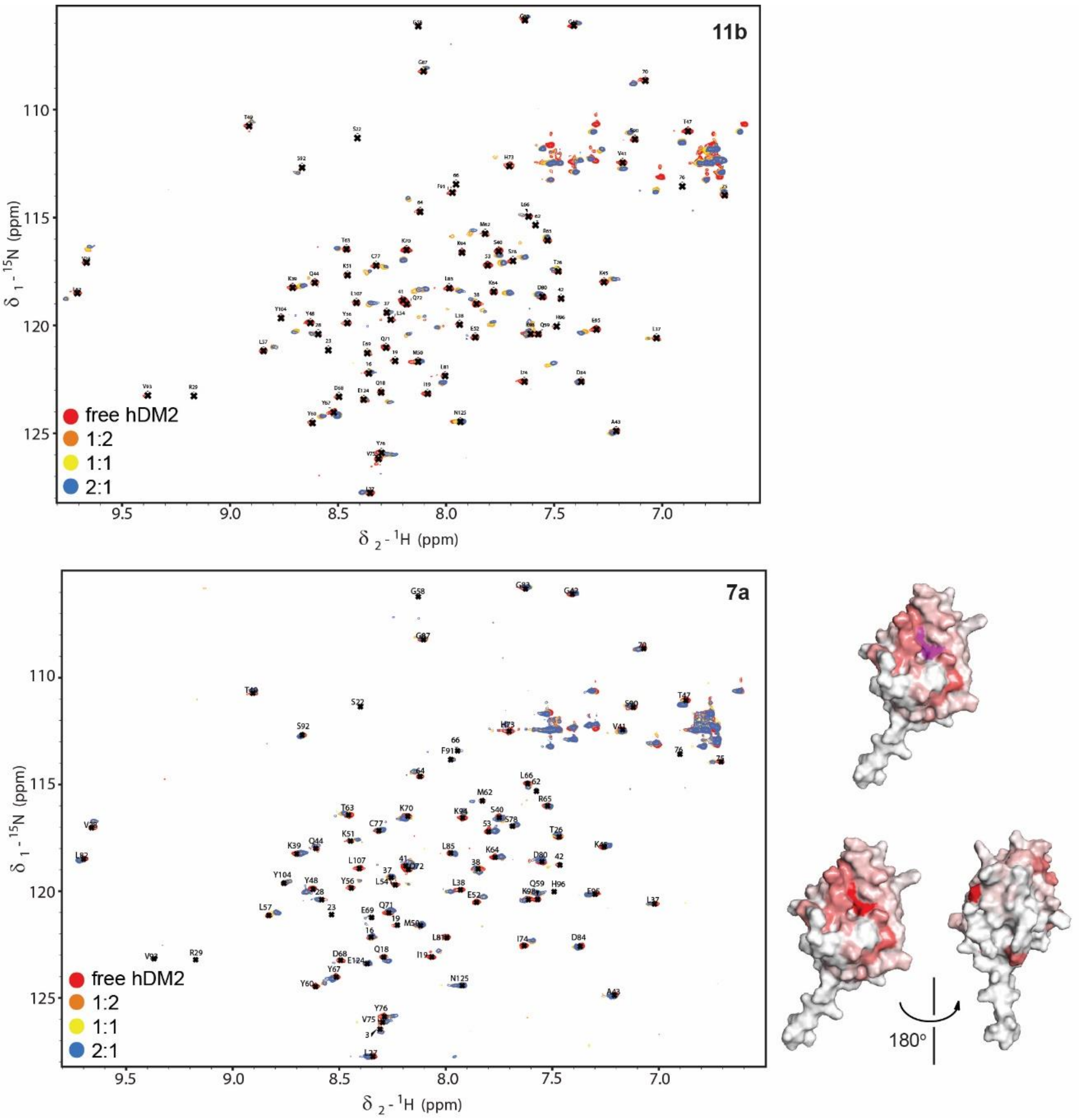

Figure S16. *Representative chemical shift perturbation of hDM2 17-125 observed upon binding to second series of ligands. For NMR titrations (750 MHz, 100mM phosphate, pH 7.4, 2.5% glycerol, 1mM DTT), increasing amount of compound was titrated into 50μM hDM2. The NMR data was analysed using Sparky. Top spectra* ***11b*** *– similar data were used to produce the chemical shift mapping diagrams for anti-**10**, **11a**, **11b**, **12** and **13** shown in Fig. 4d). Bottom spectra* ***7a*** *– here a number of resonances were lost upon titration which is consistent with slow exchange and tight binding (top shift map shows the regions where resonances disappear in pink with bottom map showing all affected regions)*

2. Further Discussion of molecular dynamics simulations

The pipeline process that selected **A1** matched the *3R* stereoisomer to the query. The pose matches the pyridyl group (P) to the F23 (green) site, the indole group (I) to the W21 (magenta) site and the thiophene (T) to the L19 (cyan) site which we can abbreviate to [PIT] (see movie R-A1 and Fig. S16a). The best docked pose of *3S*-**A1** swaps the position of the thiophene and indole groups and remains in this pose [ITP] throughout (see movie S-A1-I and Fig. S16b). A second, less favourable, *3S*-**A1** docked pose was chosen [PIT] and simulated. After 45 ns a large change occurs where the pyridyl group joins the thiophene group at the F23 site, giving non-canonical binding with an empty F23 site, indole in the W21 site and both other groups in the L19 site (see S-A1-II and Fig. S16c). Plots of ligand RMSD with time over these trajectories also show that the *S*-**A1** stereoisomer is more mobile and less tightly associated with the protein than *R*-**A1**. We interpret these results to indicate the R stereoisomer both fits the design remit and binds more tightly than S-A1.

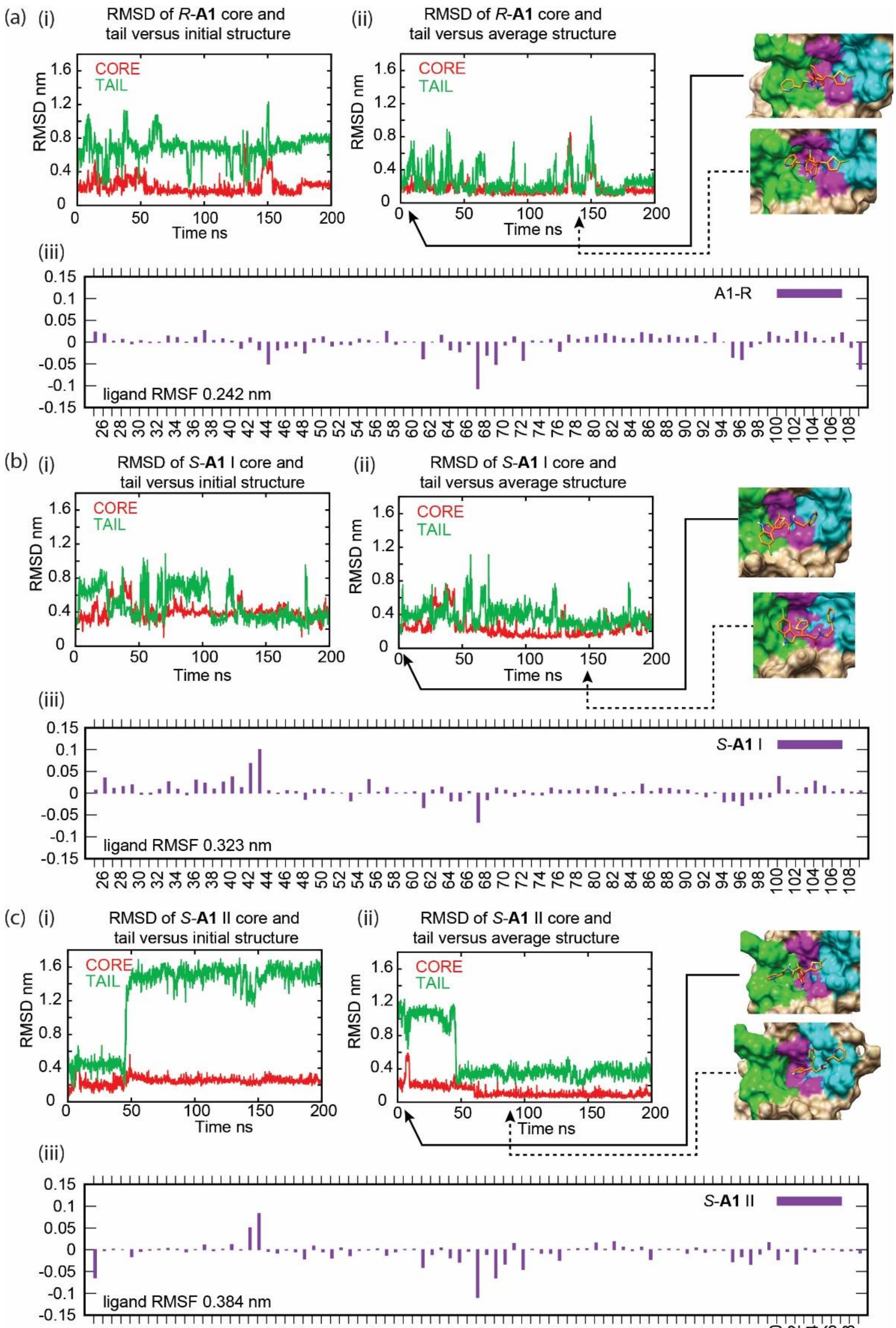

***Figure S17**. Molecular dynamics data for **A1**/hDM2 interaction (200 ns) for R-**A1** (a), for S-**A1** pose I (b) and for S-**A1** pose II (c); plots for the root mean square deviation for the core and tail of the ligand (i) with respect to the initial structure and (ii) with respect to the average structure, (iii) plots of the relative fluctuations per residue over the trajectories. Δ-RMSF = $RMSF_{complex}$ - $RMSF_{apo}$. Y ordinates are RMSD (nm) X ordinates are residue number. Left-*

hand column: RMSD versus structure at t = 0 ns; right-hand column: RMSD versus average coordinates over trajectory. Bars above zero indicate more flexibility of these residues in the complex than the in the apo. Likewise, bars below zero indicate a loss of mobility of these residues in the complex compared with the apo. The RMSF of the ligand in each complex is also shown.

Development of **A1** focussed on introducing substituents to reduce the conformational flexibility of the molecule. Firstly a methyl group was added to the 2 position to increase steric congestion adjacent to the CO-Cα bond giving a four stereoisomers. Each of these was simulated for 200 ns and the RMSD to the initial and average coordinates recorded (see movies and Fig. S17). The starting poses were based on the **A1** simulations with the 3R pair docked [PIT] and the 3S pair [ITP]. Both *2R,3R*-**10** and *2S,3R*-**10** remain close to the initial and average positions in the binding site (Fig. S18, and movies RR-10 and SR-10) and induce moderate dampening of fluctuations for residues around the recognition site (Fig. S18 RMSF per residue plots). For both *2R,3S*- and *2S,3S*-**10** the thiophene remains bound to the W21 site (Fig. S20 and movies RS-10 and SS-10). Even with non-canonical binding, *2S,3S*-**10** is mobile in its binding site, with high values evident in the RMSD plots and shows little perturbation of the protein dynamics beyond an increase in fluctuations around the C-terminus (see RMSF per residue). For *2R,3S* in the RMSD and RMSF plots its behaviour is similar to the 3R stereoisomers.

In terms of the correlation with experiment, it is worth reiterating that these simulations were based on the original docked poses of *R*-**A1** and *S*-**A1** and that alternative binding poses for each of the four diasteromers of **10** might be accessible or the ligands may adopt a combination of binding poses. For the more potent *anti* pair (*2R,3R*-**10** and *2S,3S*-**10**), the simulations indicate that introduction of the 3-methyl group indeed restricts the conformational dynamics of the pyridyl group as intended – the small difference in potency (2.9 μM vs 4.6 μM) may arise from a difference in preferred binding pose, the reduced dynamics of *2R,3R*-**10** in comparison to *2S,3S*-**10** or a combination of both. *syn*-**10** was shown to be less potent that *anti*-**10** and we did not separate the enantiomers (*2S,3R*-**10** and *2R,3S*-**10**), thus a more guarded interpretation of the simulations for this pair is warranted. However, similar qualitative conclusions may be drawn i.e. the difference in protein affinity for different poses may be subtle and introduction of a 3-methyl group restricts the conformational dynamics of the ligand.

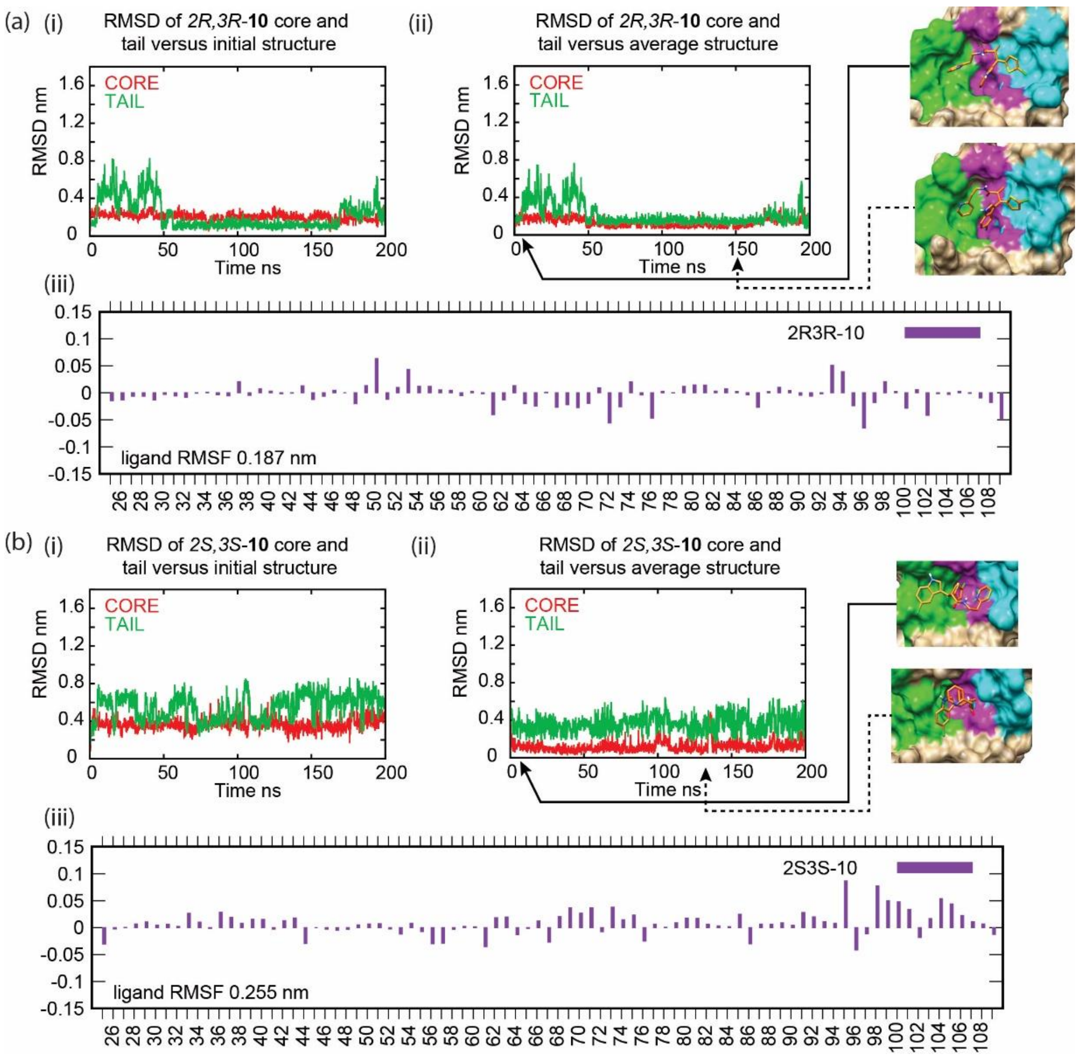

Figure S18. *Molecular dynamics data for anti-**10**/hDM2 interaction (200 ns) for 2R,3R-**10** (a) and 2S,3S-**10** (b); plots for the root mean square deviation for the core and tail of the ligand (i) with respect to the initial structure and (ii) with respect to the average structure, (iii) plots of the relative fluctuations per residue over the trajectories. Δ-RMSF = $RMSF_{complex}$ - $RMSF_{apo}$. Y ordinates are RMSD (nm) X ordinates are residue number. Left-hand column: RMSD versus structure at t = 0 ns; right-hand column: RMSD versus average coordinates over trajectory. Bars above zero indicate more flexibility of these residues in the complex than the in the apo. Likewise, bars below zero indicate a loss of mobility of these residues in the complex compared with the apo. The RMSF of the ligand in each complex is also shown.*

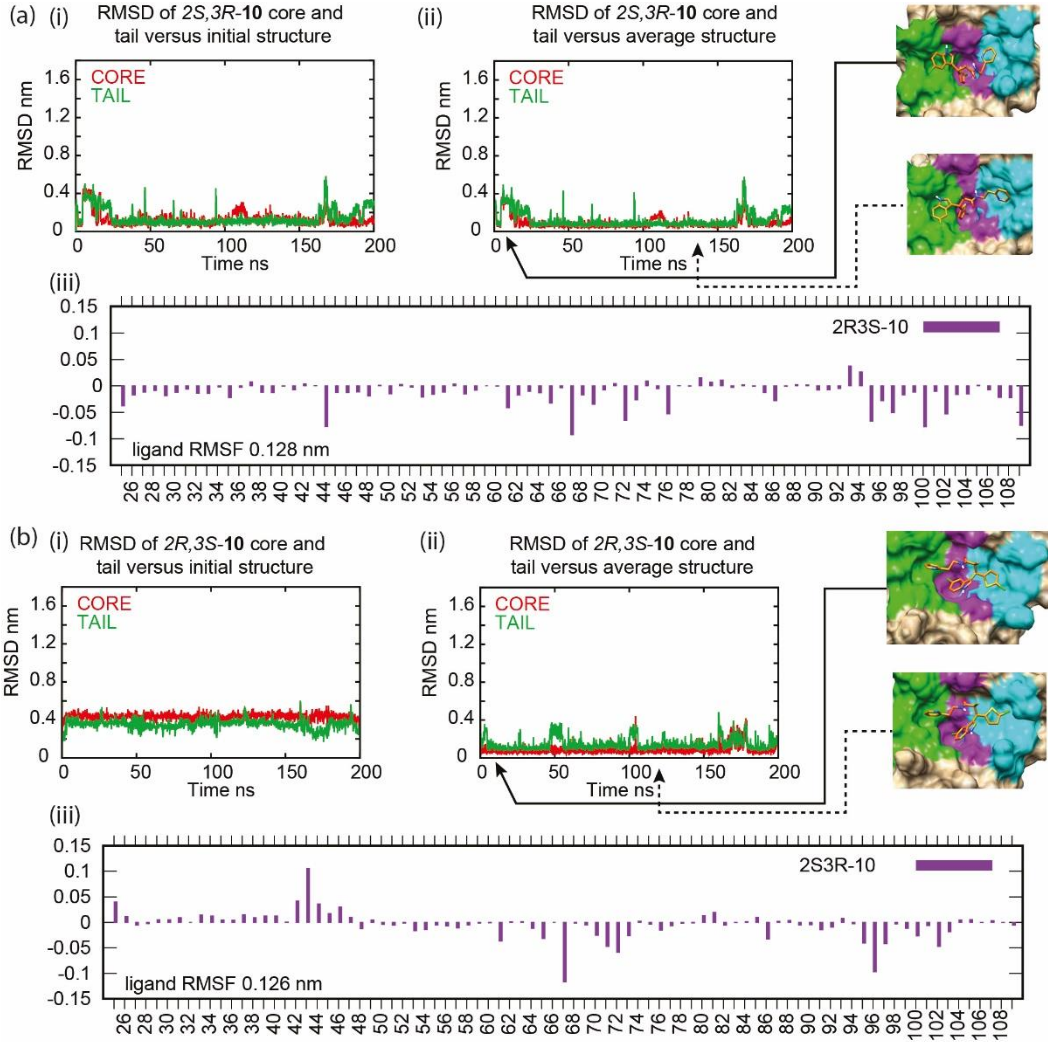

Figure S19*. Molecular dynamics data for syn-**10**/hDM2 interaction (200 ns) for 2S,3R-**10** (a) and 2R,3S-**10** (b); plots for the root mean square deviation for the core and tail of the ligand (i) with respect to the initial structure and (ii) with respect to the average structure, (iii) plots of the relative fluctuations per residue over the trajectories. Δ-RMSF = $RMSF_{complex}$ - $RMSF_{apo}$. Y ordinates are RMSD (nm) X ordinates are residue number. Left-hand column: RMSD versus structure at t = 0 ns; right-hand column: RMSD versus average coordinates over trajectory. Bars above zero indicate more flexibility of these residues in the complex than the in the apo. Likewise, bars below zero indicate a loss of mobility of these residues in the complex compared with the apo. The RMSF of the ligand in each complex is also shown.*

A similar observation was made for **7a**. Only one stereoisomer of **7a** (cyclopropyl in the pyridyl group) could be built based on the preferred pose of *R*-**A1** (*3R*,*X*,*Y*)-**7a**. Simulation of this complex shows notably low mobility of the ligand (see movie 7a and Fig. S19)

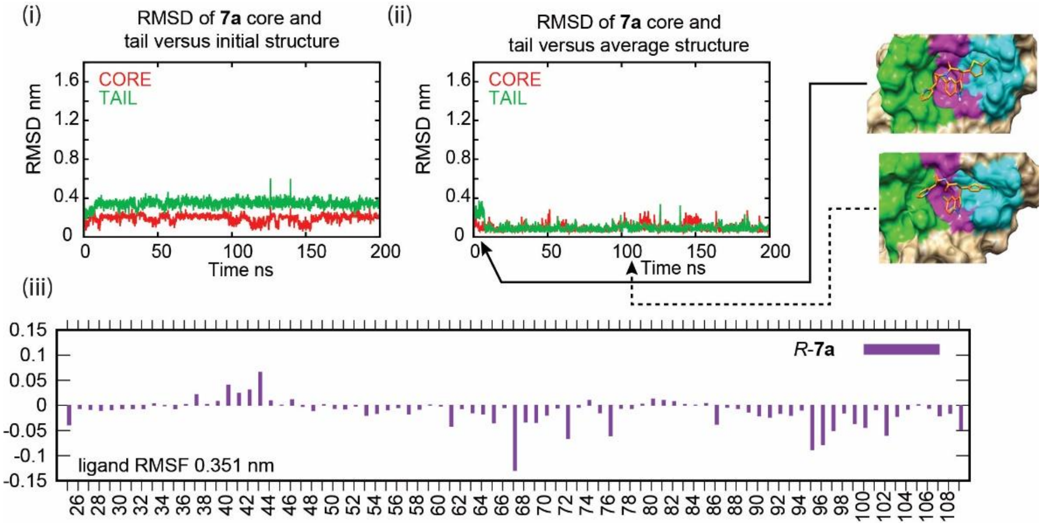

***Figure S20**. Molecular dynamics data for **7a**/hDM2 interaction (200 ns); plots for the root mean square deviation for the core and tail of the ligand (i) with respect to the initial structure and (ii) with respect to the average structure, (iii) plots of the relative fluctuations per residue over the trajectories. Δ-RMSF = $RMSF_{complex}$ - $RMSF_{apo}$. Y ordinates are RMSD (nm) X ordinates are residue number. Left-hand column: RMSD versus structure at t = 0 ns; right-hand column: RMSD versus average coordinates over trajectory. Bars above zero indicate more flexibility of these residues in the complex than the in the apo. Likewise, bars below zero indicate a loss of mobility of these residues in the complex compared with the apo. The RMSF of the ligand in each complex is also shown.*

The final rigidification strategy was to introduce substituents at the indole C2 such as in compound **11b** (CO_2H at the indole 2 position). The *R* stereoisomer of **11b** is less mobile than the *S* isomer and shows low RMSD versus the average structure (see movies R-11b and S-11b and Fig. S20). After 115 ns in the trajectory of **11b** there is a small rearrangement in the pose that corresponds to a change in the interaction between *h*DM2 and the carboxyl group. Freezing of residues around the binding site is most evident in the Δ-RMSF plot of **11b** over 120-200 ns.

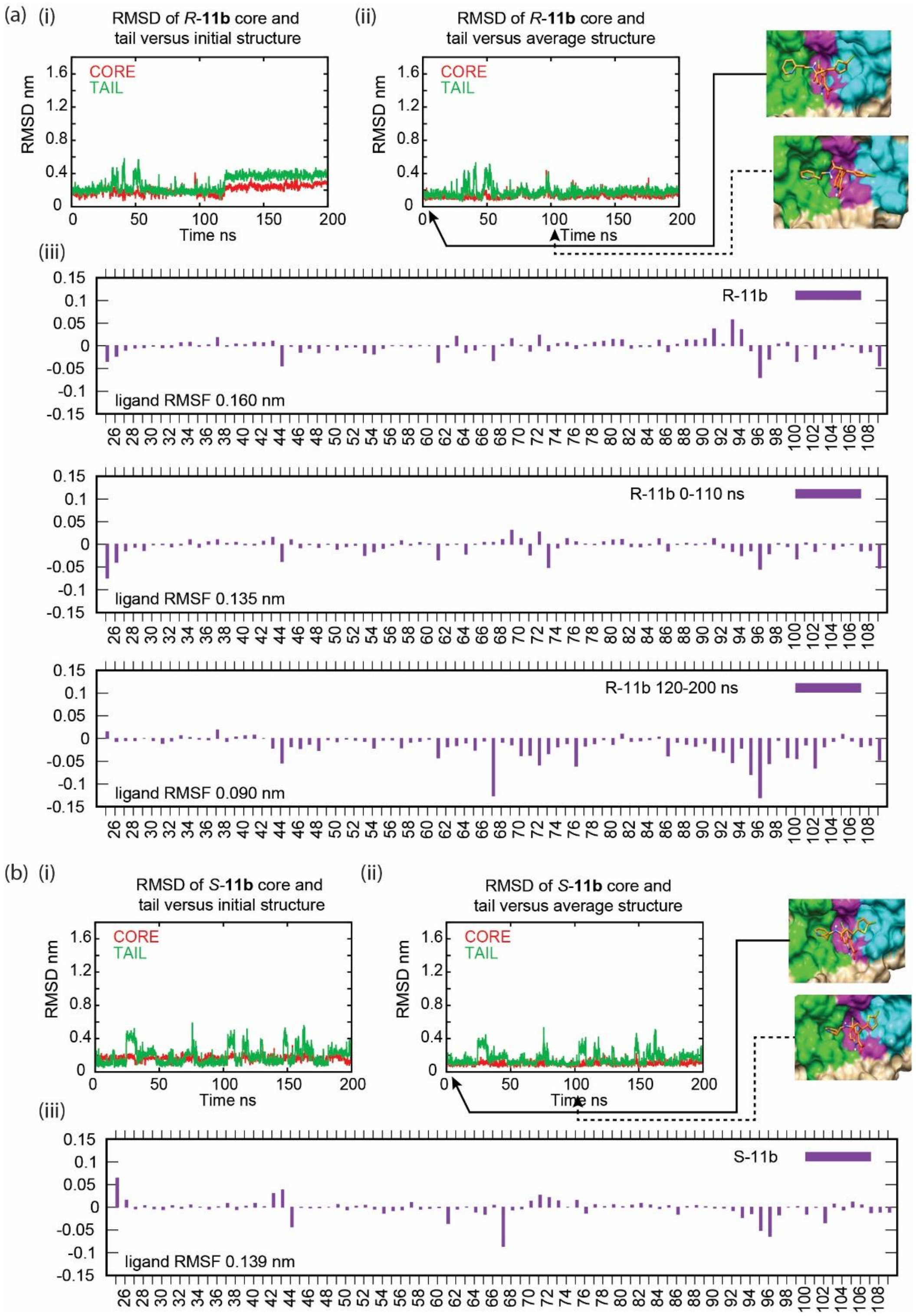

***Figure S21**. Molecular dynamics data for **11b**/hDM2 interaction (200 ns) for R-**11b** (a) and S-**11b** (b); plots for the root mean square deviation for the core and tail of the ligand (i) with respect to the initial structure and (ii) with respect to the average structure, (iii) plots of the relative fluctuations per residue over the trajectories. Δ-RMSF = $RMSF_{complex}$ - $RMSF_{apo}$. Y ordinates are RMSD (nm) X ordinates are residue number. Left-hand column: RMSD versus structure at t = 0 ns; right-hand column: RMSD versus average coordinates over trajectory. Bars above zero indicate more flexibility of these residues in the complex than the in the apo.*

Likewise, bars below zero indicate a loss of mobility of these residues in the complex compared with the apo. The RMSF of the ligand in each complex is also shown.

3. Determination of the relative configuration of diastereomers

The coupling constant between protons H2 and H3 is ^3J = 11.0 Hz for *syn*-**10** and *anti*-**10** isomers and ^3J = 11.5 Hz for *syn*-**49** and *anti*-**49** isomers. Following the Karplus approximation, in which ^3J = 8-15 Hz for vicinal trans protons, we used the Newman projection along the C2-C3 bond to determine the relative *syn*/*anti* configuration by ^{1}H-^{1}H NOE correlation signals.

syn-3-(5-Chloro-1H-indol-3-yl)-3-(5-chlorothiophen-2-yl)-2-methyl-*N*-[2-(pyridin-2-yl)ethyl]propenamide (*syn*-10)

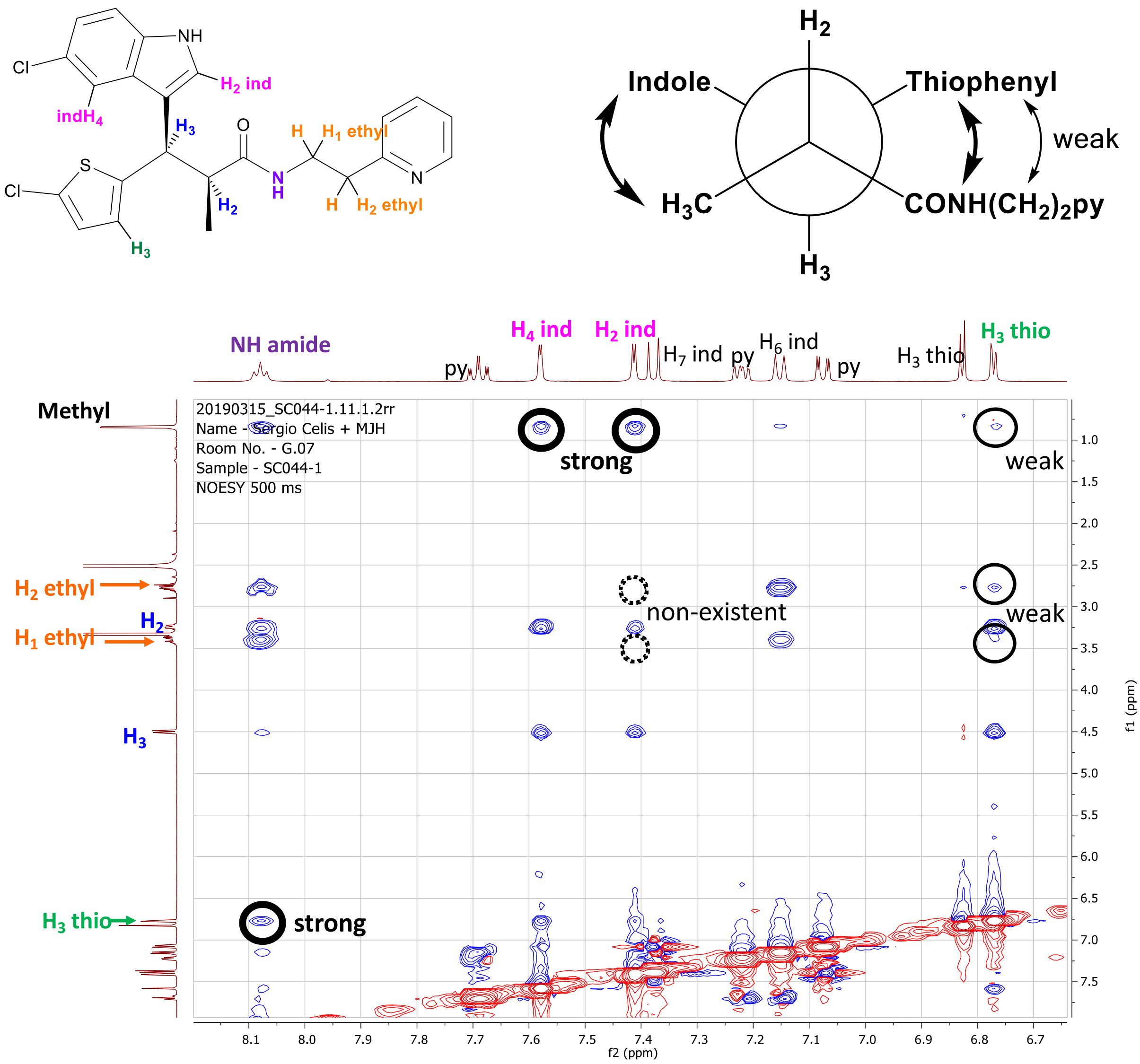

Figure S22. *Resolution of the relative configuration of syn-**10**. Top: chemical structure highlighting relevant protons and Newman projection. Bottom: extract of the ^{1}H-^{1}H NOESY correlation experiment.*

***anti*-3-(5-Chloro-1H-indol-3-yl)-3-(5-chlorothiophen-2-yl)-2-methyl-*N*-[2-(pyridin-2-yl)ethyl]propenamide (*anti*-10)**

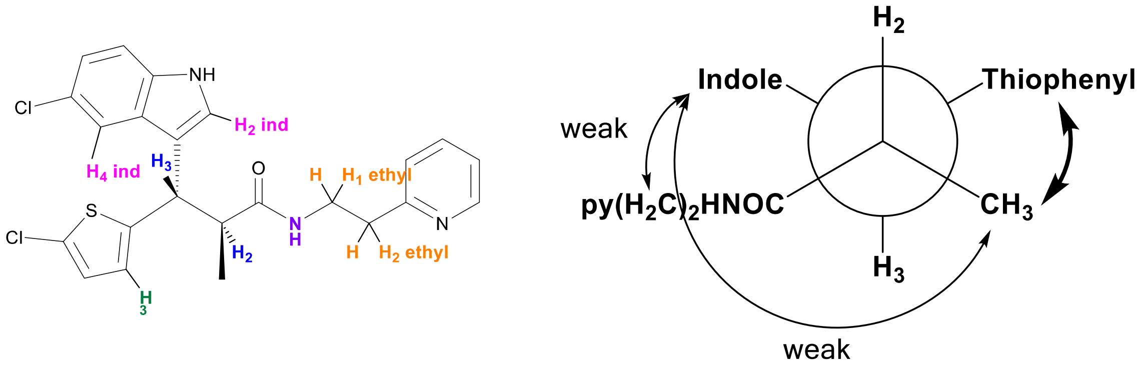

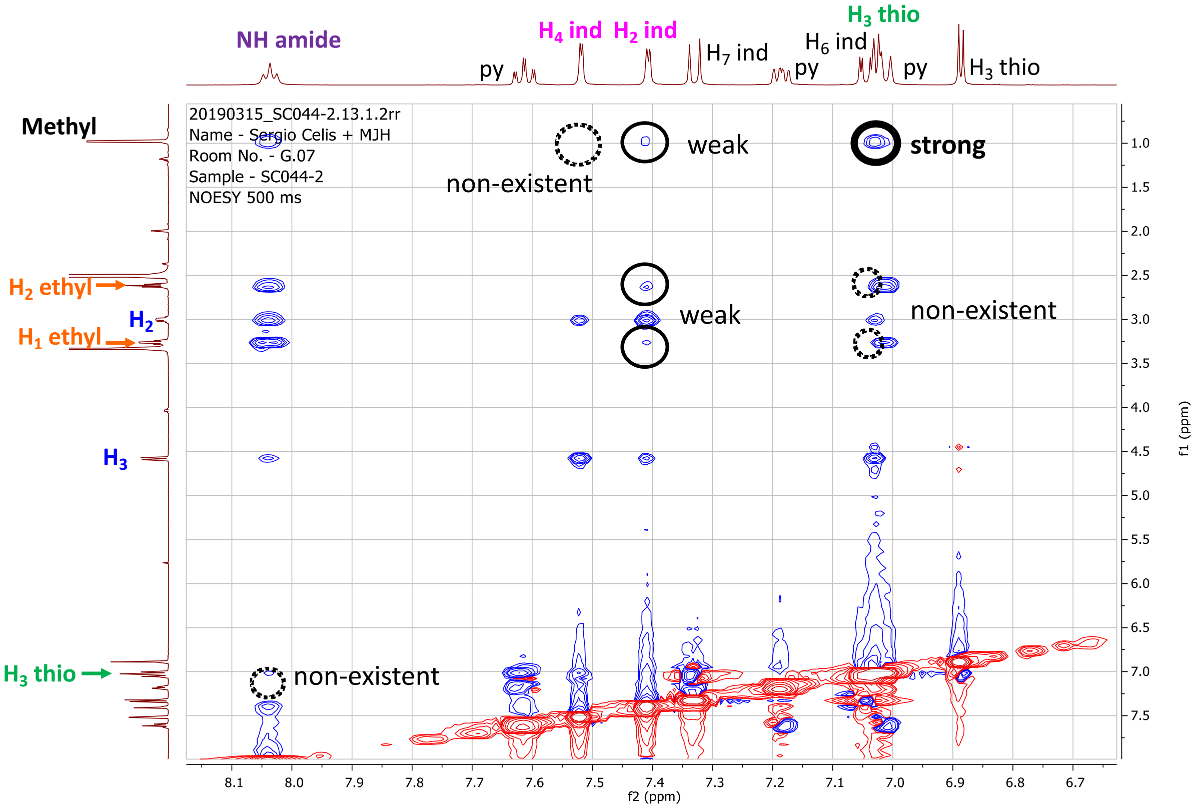

Figure S23. *Resolution of the relative configuration of anti-**10**. Top: chemical structure highlighting relevant protons and Newman projection. Bottom: extract of the ^{1}H-^{1}H NOESY correlation experiment.*

4. Chiral separation of enantiomers (+)-*anti*-10 and (–)-*anti*-10

sepiatec
SEPARATION INNOVATION AUTOMATION

Prep SFC Report

**Samplename:**	SC044-2_LEEDS_02		
**Run Started:**	12/5/2019 6:36:04 PM	**Reported:**	12/9/2019 5:19:18 PM

**Method:**	C1_30_MeOH_NH3		
**Injection:**	1 / 2	**Wavelength [nm]:**	220
**Injectionvolume [ml]:**	1.10	**Modifier:**	C: MEOH+NH3
**Flow [ml/min]:**	90	**Modifierpercent:**	30
**Column:**	Phenomonex C1		

System Settings

**Backpressure [bar]:**	120	**Temp Pumphead [°C]:**	10
**Temp Fraction Module [°C]:**	15	**Temp Columndepartment [°C]:**	40

Chromatogram

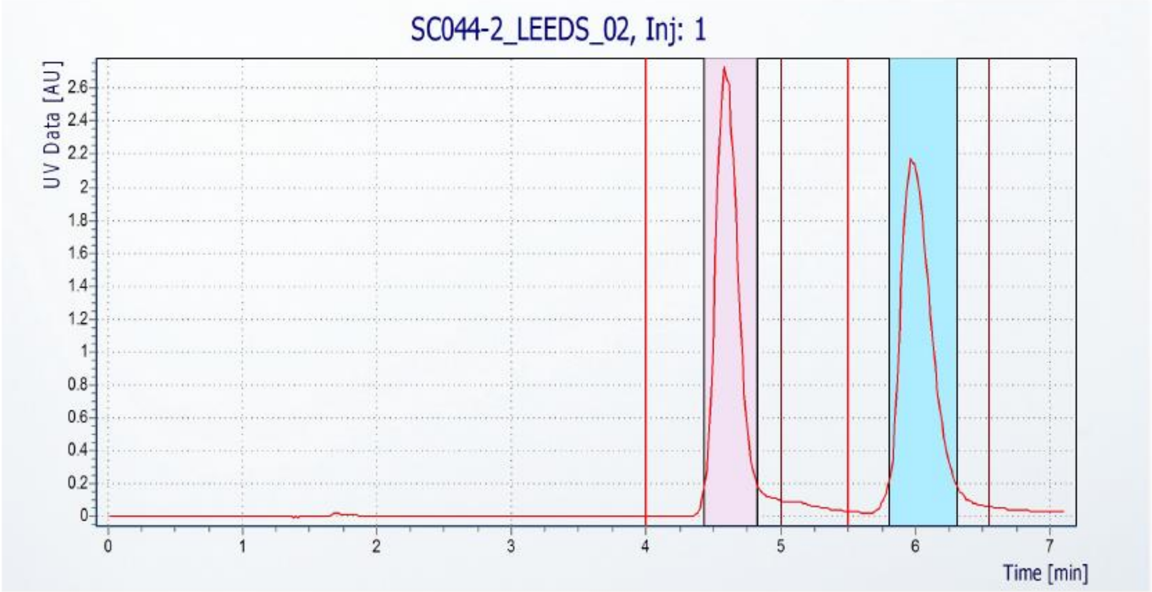

Peak Number	Peak Start	Peak Stop	Vial No.
1	4.00	5.00	1
2	5.50	6.54	2

Figure S24. *Method and conditions for the chiral separation of enantiomers (+)-anti-**10 and** (–)-anti-**10**.*

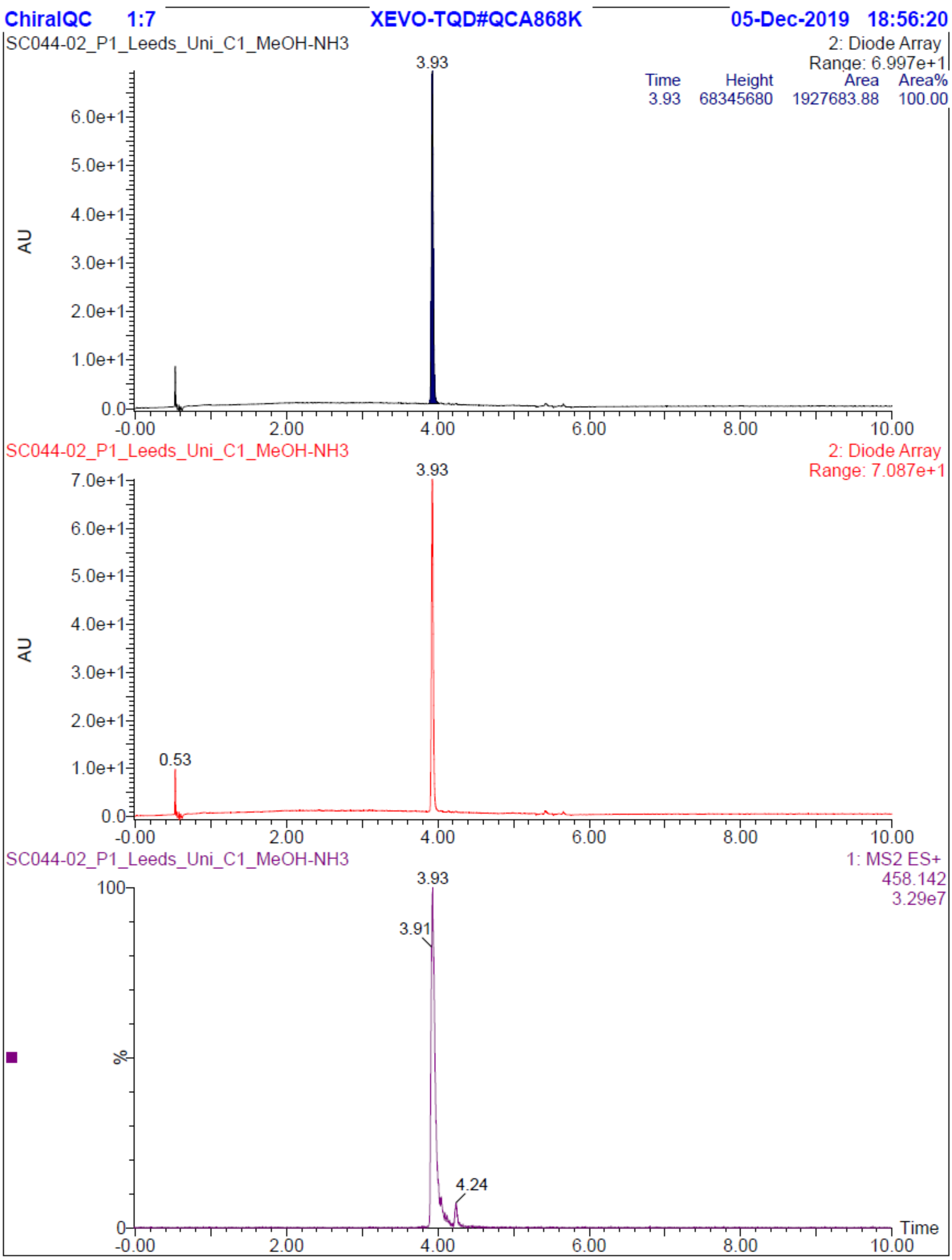

Figure S25. *Analytical chromatogram of enantiomer (+)-anti-**10**.*

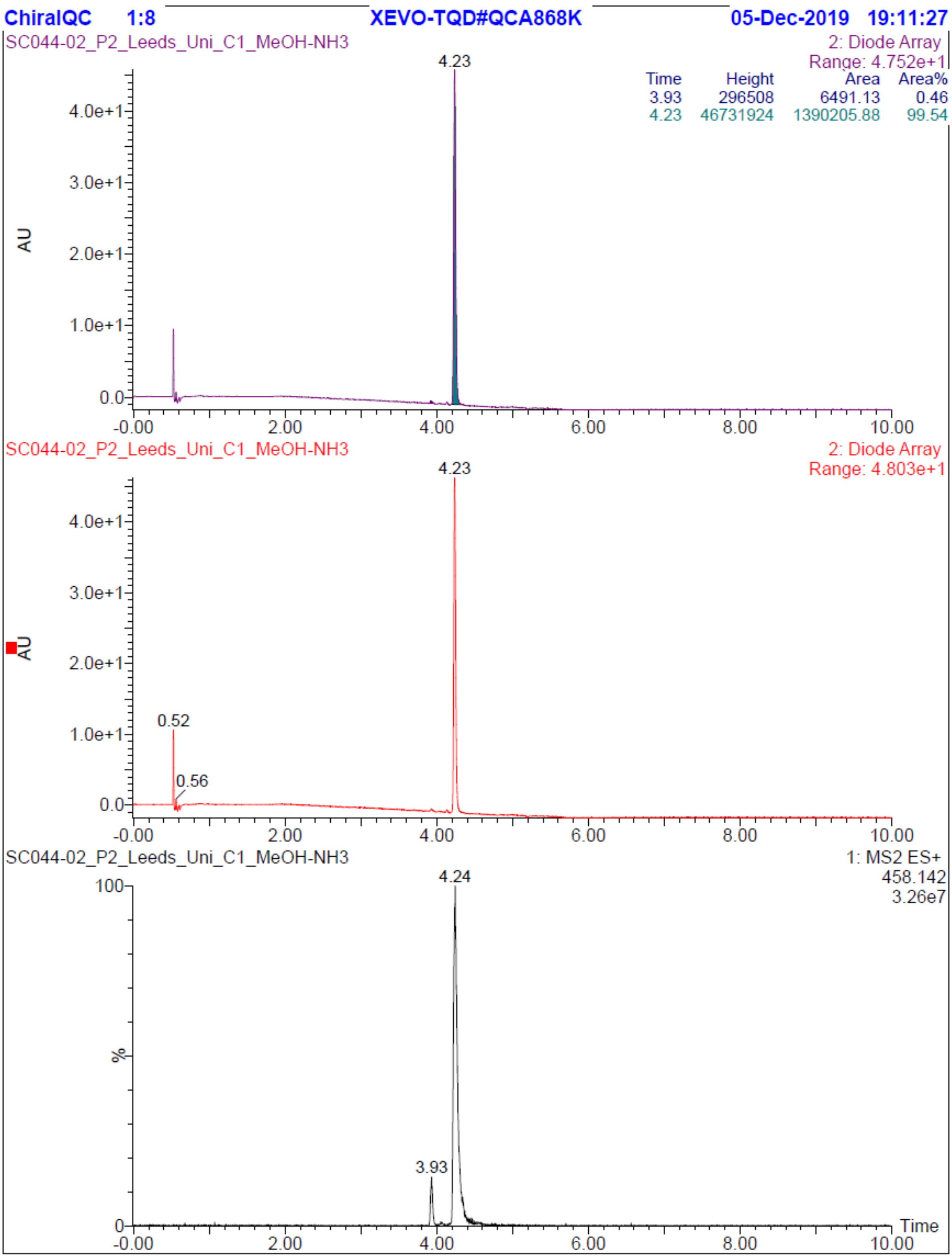

Figure S26. *Analytical chromatogram of enantiomer (–)-anti-**10**.*

5. Supplementary methods

5.1. Biophysical methods

5.1.1. Screening of AZ compounds

Screening of AZ compounds – hDM2

Both for the initial round and for the near neighbours, compound screening was carried out using a competition fluorescence anisotropy assay. 25nM *$p53_{15\text{-}31}Flu$* bound to 150nM *h*DM2 was competed against 10 or 100µM compound in assay buffer containing 40mM phosphate (pH 7.5), 200mM NaCl, 0.02mg/ml Bovine Serum Albumin and 1% DMSO. Results were collected using a Perkin-Elmer Envision 2103 Multilabel Reader using a D505fp dichroic mirror, 480(104) nm excitation filter (band width 30 nm), and 535(208-S) and 535(209-P) nm emission filters (band width 40 nm) after 0, 1, 4 and 24 hours of incubation at room temperature (*Plate Reader I*) or Tecan Spark plate reader using a 485 nm excitation filter (band width 20 nm),, and 535 (S) and 535 (P) nm emission filters (band width 25 nm) after 0, 1, 4 and 24 hours of incubation at room temperature (*Plate Reader II*). Wells containing only hDM2 in assay buffer were used as a blank and Nutlin 3 was used as positive control. Assays were carried out as triplicates.

Fluorescence anisotropy data was processed as described previously.[1-4] The data from both the P (perpendicular intensity) and S (parallel intensity) channels, resulting from this measurement was corrected by subtracting the corresponding control wells, and used to calculate the intensity and anisotropy for each well following Equations 1 and 2. The average anisotropy (across all replicates) and the standard deviation of these values were then calculated and fit to a sigmoidal logistic model using Origin to determine the maximum (r_{max}) and minimum (r_{min}) anisotropies. These were used to calculate the fraction ligand bound (Lb) (Equation S3). The fraction ligand bound was multiplied by the [FL] and fit to the model shown in Equation S4 to determine a K_d:

$$I = (2PG)+S \quad Equation\ 1$$

$$r = (S-PG) \quad Equation\ 2$$

$$L_b = (r-r_{min})/\lambda(r_{max}-r)+r-r_{min} \quad Equation\ 3$$

$$y = \{(k+x+[FL])-\surd\{k+x+[FL]^2-4x[FL]\}\}/2 \quad Equation\ 4$$

r = anisotropy, I = total intensity, P = perpendicular intensity, S = parallel intensity, L_b = fraction ligand bound, λ = Ibound/Iunbound = 1, [FL] = concentration of fluorescent ligand, k = K_d , y = L_b Flu-trimer and x = [added titrant], G is an instrument gain factor.*

Percentage inhibition values were then calculated using Nutlin 3 as positive control for both 10 and 100µM compound concentrations.

$$\% \, inhibition = \frac{DMSO\ control\ anisotropy - sample\ anisotropy}{DMSO\ control\ anisotropy - nutlin\ 3\ anisotropy} \times 100$$

The determined values were plotted as a column graph for visualisation, the 4h time point was used to select compounds for further testing.

Screening of AZ compounds – SHANK1 PDZ

The screening of compounds was carried out similarly as described for ***h***DM2 using 50nM FITC labelled GKAP bound to 1µM SHANK1-PDZ, in TRIS buffer (20 mM TRIS, 50 mM NaCl, pH 7.4, 3% DMSO).

Compounds were screened at 30 and 300µM concentration, unlabelled GKAP peptide was used as a positive control.

5.1.2. IC_{50} determination – fluorescence anisotropy competition assays

Selected compounds both for *h*MD2/p53, MCL-1/NOXA-B and SHANK/GKAP were tested in competition assays as previously described.[1, 2]

5.1.3. NMR spectroscopy

All NMR experiments were carried out using Bruker Avance NMR spectrometers operating at 750, MHz ^{1}H frequency. Temperature was maintained at 298 K. Data were processed using Topspin and analysed in NMRFAM-Sparky.[5, 6]

NMR titrations for *h*DM2 were performed in 100mM Na_2HPO_4, 2.5% glycerol, 1mM DTT pH 7.4 buffer by recording ^{1}H-^{15}N SOFAST NMR spectra of protein-small

molecule complexes. A 50 µM sample of *h*DM2(17-125) was titrated with various compounds at 0, 0.5, 1, 2, 4 or 0, 0.25, 0.5, 0.75, 1, 1.5, 2 compound:protein ratios. NMR titrations for SHANK1 PDZ were performed in 5 mM TRIS, 100 mM NaCl, pH 7.4.

5.1.4. Crystallography

SHANK1-PDZ was incubated with L6F-GKAP at 1:2 protein:ligand molar ratio in 25 mM Tris, 150 mM NaCl pH 7.5 buffer having 10.5 mg/ml final protein concentration. Conditions were screened using sitting-drop vapor-diffusion method using JCSG Core suites (Quiagen) with mixing 1:1 drops (0.2 µl) at 20 °C. Initial hit conditions were further optimized by screening HEPES pH 7 – 8.25 in 0.25 pH steps and PEG 400 concentration from 25-40% in 5% steps, crystals grew within 3-8 days. Crystals were frozen in liquid nitrogen without addition of any further cryorprotectant and sent to Diamond Lightsource (DLS) for data collection. Data was collected at 100K. Data were processed with the xia2[7] bundle using XDS[8] for integration and Pointless,[9] Aimless[10] for scaling and merging. Phasing was performed by molecular replacement using Phaser[11] and using Chain A from PDB ID: 1Q3O as a search model. Refinement was done using REFMAC[12] and model building in COOT[13] using the CCP4i2[14] software package. TLS refinement was done in PHENIX[15] and structures were analysed by Molprobity.[16]

5.1.5. Protein expression and purification

*h*DM2 17-125 was expressed and purified as described previously.[1]
MCL-1 (172-327) was expressed and purified as described previously.[2]
Shank 1 PDZ domain (656-762) was expressed and purified as described previously.[2]
^{15}N labelled samples were prepared following the same protocols as above, with the expression being carried out in M9 minimal media supplemented with ^{15}N NH_4Cl.

5.1.6. Isothermal titration calorimetry experiments

ITC experiments of SHANK with GKAP was carried out as previously described.[2]

5.2. Computational methods

Scaffold preparation Hot residues at the interface of a given protein-protein complex were predicted using the *in silico* alanine scanning web application BAlaS.[17] A set of neighbouring hotspot residues were selected by inspection from the protein partner to be mimicked by the small molecule. We developed a Python application, scaffoldSearch.py, to facilitate the subsequent steps of the search process. ScaffoldSearch.py is run in one of four modes: 'query', 'search', 'dock', 'results' or 'full' meaning run all four modes in order. Query mode takes a list of residues and generates the query structure for the next step. Search mode takes the query structure and a database of small molecules and passes this to ROCS (OpenEye)[18-24] for similarity searching. Dock mode takes the best hits from 'search' and passes these to BUDE for (re)docking (this code can be readily modified to use other virtual screening or docking tools).[25, 26] Results mode compiles the results into human-friendly format.

This code is available on GitHub https://github.com/richardbsessions

Structure similarity searching, protein docking and scoring In this work a modified workflow was used to identify hits as follows. A query protein with a defined receptor binding surface was uploaded into VIDA (https://www.eyesopen.com/vida) along with the relevant peptide query. A virtual library of ~42 million conformers representing the AstraZeneca screening collection was shape-matched using FastRocs.[18-24] The sum of the ShapeTanimoto and ColorTanimoto scoring functions (TanimotoCombo) was used as the primary filter to select 1000 top scoring hits. The hits were then docked rigidly into the receptor surface using OEDocking (also in VIDA). A significant number of compounds had flipped through ~180 degree during docking and these were removed by applying a script which calculated RMS difference of initial pose to docked pose. High RMS difference compounds were removed

A large number of fragment like hits were observed to be well-scoring two-pocket binders. A handful (<20) were retained, but most were discounted so as to maximise the number of three-pocket binders selected for further profiling. Tanimoto clustering was performed and representative cluster members were selected plus a small number of structurally distinct singletons. Once the *in silico* hit list had been reduced to 100 potential hits per query, a random set of 100 compounds was selection from the AZ screening collection, aiming to explore similar logD, rotatable bond and

molecular weight space to the selected compounds. A comparison of binned rotatable bonds and calculated logD are shown as two examples:

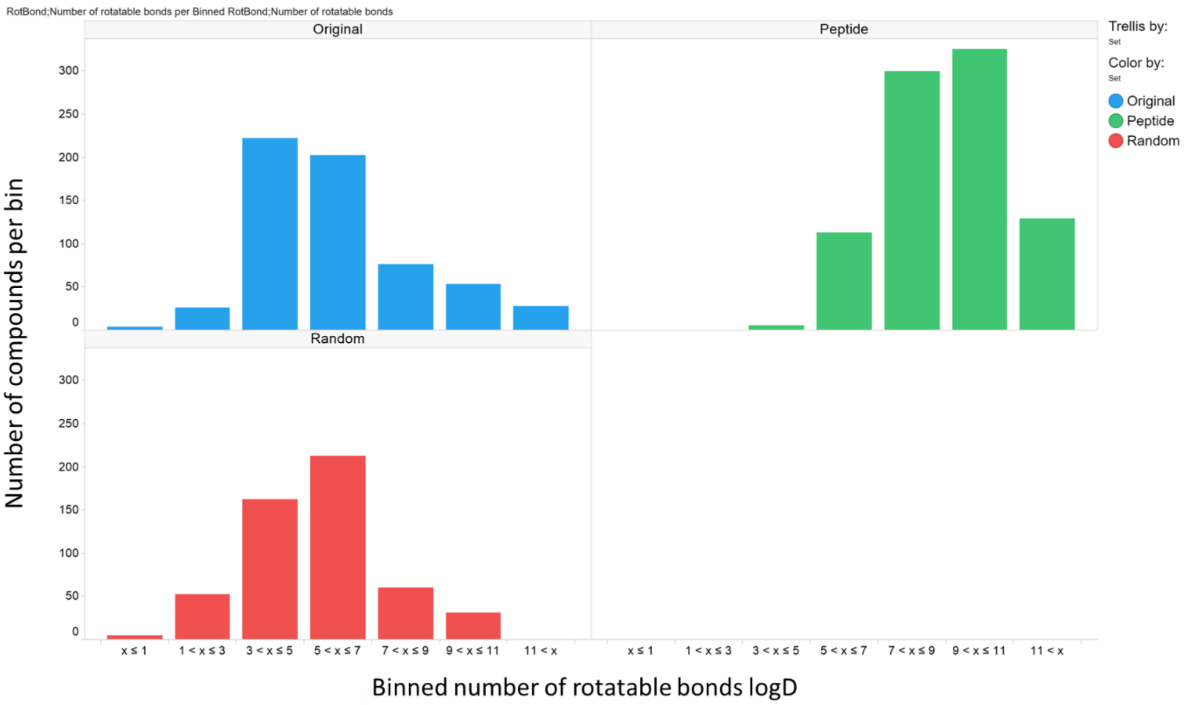

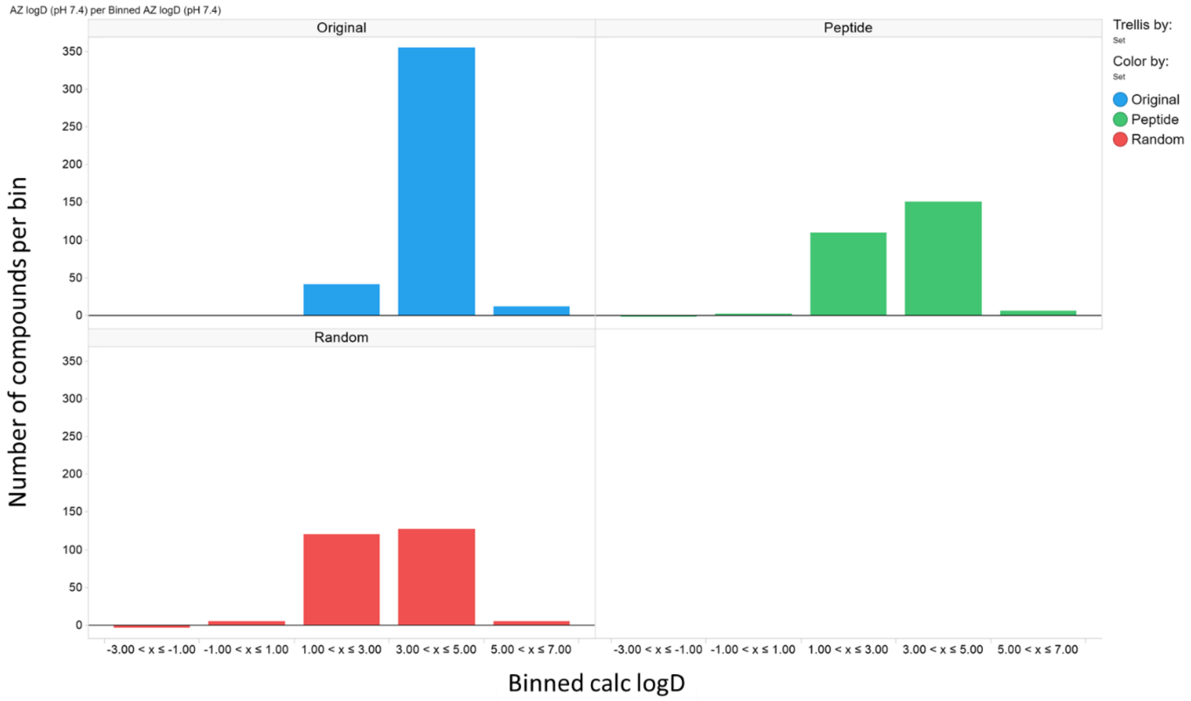

All compounds from the three sets (the Hydrocarbon query – ‘Original’; the peptidic query – ‘Peptide’; compounds hitting both queries – ‘Both’ were checked for frequent hitter behaviour against the control set of compounds. Frequent hitters are identified by virtue of a highly negative logBSF score. Anything more negative than -5 is regarded as questionable.[27] In the figure below, anything not blue is a frequent-hitter as was removed from consideration for IC_{50} follow-up.

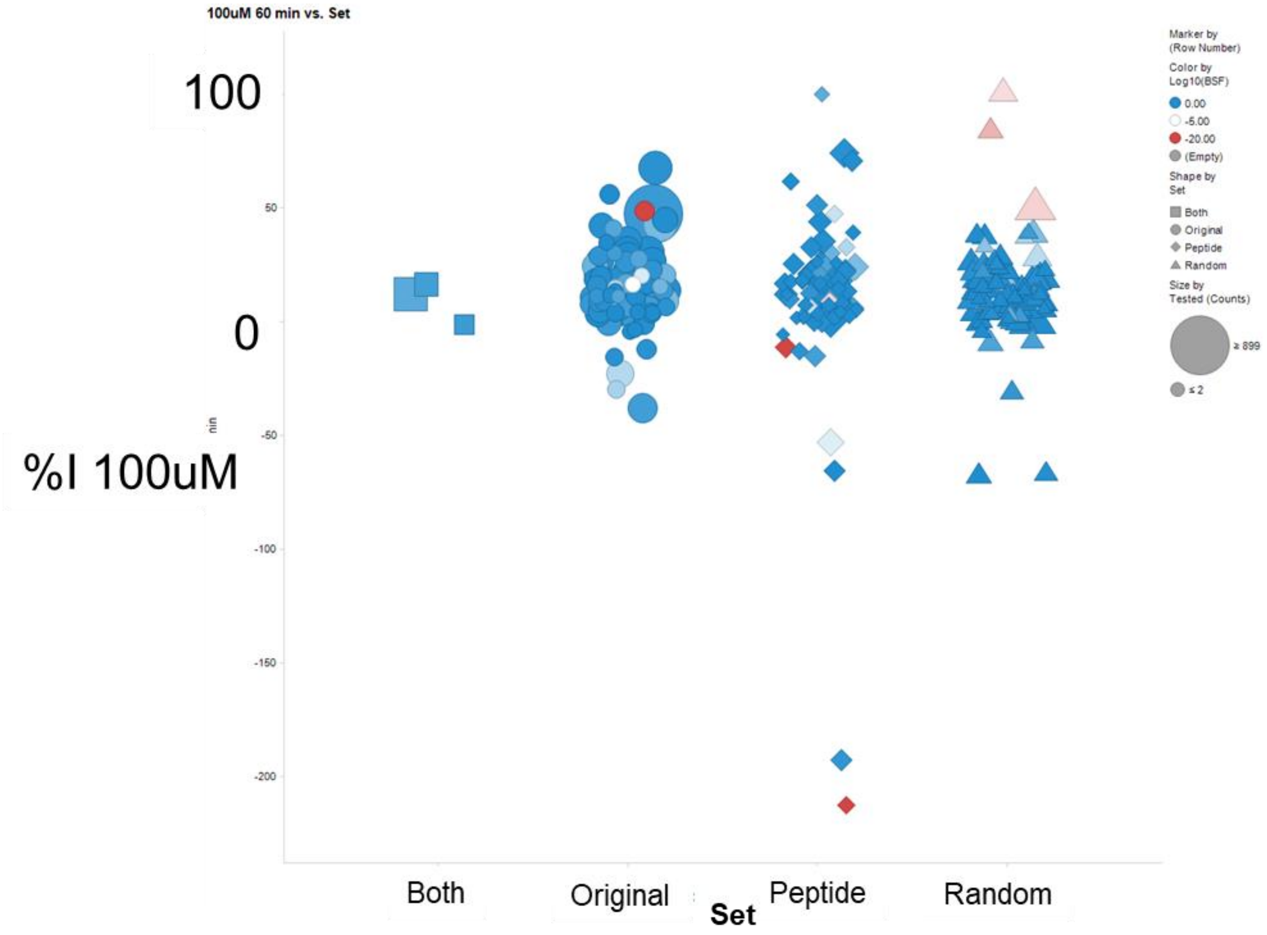

Compound sets – Frequent hitter status

The percent inhibition data at 100 uM on the 200 compounds from the ‘Original’ and ‘Peptide’ sets was reclustered at Tanimoto 0.7 level of similarity; some clusters were manually combined due to highly similar substructures/embedded scaffolds:

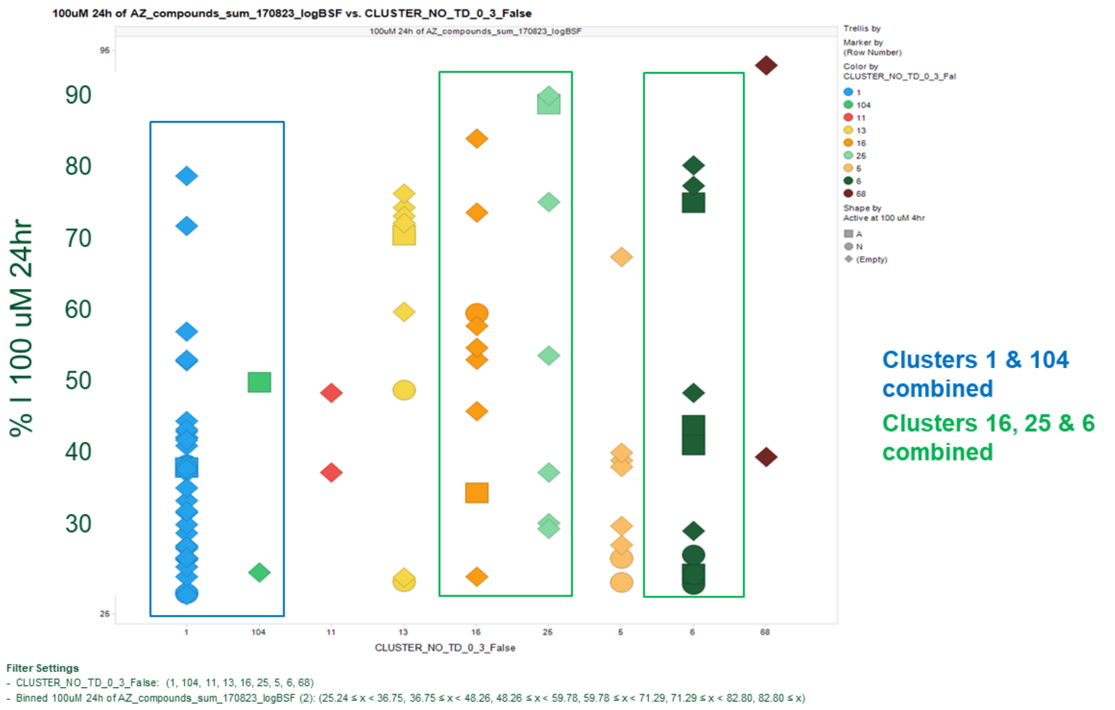

Clusters (including combined clusters) by %Inhibition

Following clustering, the larger AZ screening collection was mined for near neighbours and the selected hits (43) augmented with NNs (257) for the IC_{50} round of testing.

5.3. Molecular Dynamics Methods

The protein-ligand complexes selected for simulation were all treated the same way. The protein was parameterised with the amber99SB-ildn forcefield[28] and the small molecules with GAFF.[29] All simulations were performed using GROMACS 2019.3.[30] using the following general protocol. Hydrogen atoms were added consistent with pH 7. The protein-ligand complex was placed in a cubic box 3 nm larger than the longest dimension of the protein and filled with TIP3P water containing 0.15 M sodium chloride ions to give a charge-neutral system overall. After 10000 steps of steepest descent minimisation, molecular dynamics was initiated with random velocities while restraining the protein backbone to its original position with a force constant of 1000 kJ/nm for 0.2 ns. Simulations were developed for a further 200 ns without the backbone position restraints under periodic boundary conditions. The Particle Mesh Ewald's method was used for long range electrostatic interactions while short range Coulombic and van de Waals energies were truncated at 1.4 nm. The temperature

was maintained at 300 K using the v-rescale method and the pressure at 1 bar with the Parrinello-Rahman barostat and a 2 fs time step for the leapfrog integrator. Bond constraints were implemented with the LINCS method and SETTLE used for waters. Trajectories were processed and analysed with the GROMACS tools and visualised with VMD 1.9.3[31] and PyMol 1.8.x. Movies were created using Chimera 1.14[32] and plots made with gnuplot 5.2.2.

5.4. Synthetic methods

Reactions were carried out in clean and dried glassware under normal atmosphere unless otherwise stated. Solvents and reagents were purchased from Sigma-Aldrich or Fisher and used without further purification unless otherwise stated. Anhydrous dimethyl formamide was obtained from Sigma Aldrich equipped with Sure/Seal™ caps. For reactions under non-anhydrous conditions, the solvents used were HPLC quality and provided by Sigma Aldrich or Fisher. Water in aqueous solutions and used for work up/quenching was deionised. Mixtures of solvents are quoted as ratios and correspond to a volume: volume ratio. Analytical thin layer chromatography was performed on Merck Kieselgel 60 F254 0.25 mm pre-coated aluminium plates. Product spots were visualised under UV light (λ_{max} = 254 nm) and/or stains on heating such as phosphomolybdic acid, potassium permanganate and ninhydrin. Purification by flash chromatography was mainly carried out using automated purification by Isolera One (Biotage®) using silica column cartridges from RediSepRf®, Claricep® and Phenomenex®. Alternative non-automated flash chromatography purification was carried out using Merck Kieselgel 60 silica gel 60 (0.043 – 0.063 mm VWR) using pressure by means of head bellows. Nuclear magnetic resonance spectra were obtained at 298 K, unless otherwise stated, using a Bruker AV500 spectrometer operating at 11.4 T (500 MHz for ^{1}H and 125 MHz for ^{13}C) or a Bruker AV3 HD-400 spectrometer (400 MHz for ^{1}H and 100 MHz for ^{13}C) as stated and references to the solvent peak. Infra-red spectra were obtained using a Perkin-Elmer FTIR spectrometer where absorption maxima (υ_{max}) are quoted in wavenumbers (cm^{-1}) and only structurally relevant absorptions have been included. High Resolution Mass Spectra (HRMS) were recorded on a Bruker Daltonics micro TOF using electrospray ionisation (ESI). Liquid Chromatography and Mass Spectrometry (LC-MS) was performed using an Agilent Technologies 1200 series LC and a Bruker HCT ultra ion-trap MS. The

assignment of the ^{1}H and ^{13}C nuclei to their corresponding NMR signals was done according to their IUPAC nomenclature unless otherwise indicated. When mixtures of diastereomers have been characterised, ^{1}H and ^{13}C signals have been described as 'maj' or 'min' according to the major or minor isomer, respectively. Integration of the ^{1}H signal from major and minor isomers associated to the same structural proton has been normalised to sum to the unity. Chiral separation of enantiomers was performed by supercritical fluid chromatography in Sepiatec 100 preparative instrument using Phenomenex® C1 columns (30 x 250 mm, 5 micron). Method conditions employed a back-pressure regulator at 120 bar, 40 °C of temperature and a UV υ_{max} = 210 nm. The collected fractions were dried down into vials using Genevac Rocket.

5.1. Peptides

The p53 tracer $p53_{15\text{-}31}Flu$ (Ac-SQETFSDLWKLLPENNVC(Flu)-NH_2), where Flu is a N-(5-Fluoresceinyl)maleimide adduct, was purchased from Peptide Protein Research Ltd. As in prior studies.[3, 4, 33, 34] wt GKAP (Ac-EAQTRL-CO_2H) and FITC-Ahx-GKAP (FITC-Ahx-EAQTRL-CO_2H, where FITC is fluoresceine isothiourea) were prepared previously[2] whilst L6F GPAK (Ac-EAQTRF-CO_2H) was prepared using methods as described in this prior report.[2]

5.2. General synthetic procedures

For methodologies that have been described once, individual procedures are described at their corresponding experimental description.

5.2.1. Procedure A: multicomponent reactions

A1: Multicomponent reaction with C2-H indole derivatives

To a solution of the indole (1.1 eq.) and *p*-toluenesulfinic acid (2.0 eq.) in dichloromethane (5 mL/mmol), the aldehyde (1 eq.) was added and the reaction mixture stirred at room temperature for 1 to 20 h. The mixture was partitioned between dichloromethane/saturated aqueous sodium hydrogen carbonate solution (the added volume of hydrogen carbonate solution was equal to the total reaction volume). The combined organic layers were dried over magnesium sulfate, filtered and concentrated *in vacuo*.

A2: Multicomponent reaction with C2-substituted indole derivatives

To a solution of the indole (1.1 eq.), *p*-toluenesulfinic acid (4 to 5 eq.) and *p*-toluenesulfonic acid monohydrate (1 eq.) in tetrahydrofuran (5 mL/mmol), 5-chloro-2-thiophenecarboxaldehyde (1 eq.) was added and the reaction mixture stirred at 80 °C for 16 to 30 h. The mixture was partitioned between ethyl acetate/saturated aqueous sodium hydrogen carbonate solution (the added volume of hydrogen carbonate solution is equal to the total reaction volume). The combined organic layers were dried over magnesium sulfate, filtered and concentrated *in vacuo*.

In the case of C2-CO_2H, the reaction mixture was concentrated *in vacuo* and the crude material purified by column chromatography (silica) without previous work-up.

5.2.2. Procedure B: methyl carboxylate ester formation

B1: Methyl carboxylate ester formation with C2-H indole derivatives

To a solution of the indole (1 eq.) and silyl enol ether (3 to 5 eq.) in dichloromethane (5 to 20 mL/mmol) at 0 °C, trifluoromethanesulfonic acid (5 to 40%) was added and the reaction mixture stirred at room temperature for 3 to 16 h. The mixture was partitioned between dichloromethane/saturated aqueous sodium hydrogen carbonate solution (the added volume of hydrogen carbonate solution is equal to the total reaction volume). The combined organic layers were dried over magnesium sulfate, filtered and concentrated *in vacuo*.

B2: Methyl carboxylate ester formation with C2-substituted indole derivatives

To a solution of the indole (1 eq.) and [(1-methoxy-1-propenyl)oxy](trimethyl)silane or tert-butyl((1-methoxyvinyl)oxy)dimethylsilane (4 to 10 eq.) in tetrahydrofuran (10 to 20 mL/mmol) at room temperature, trifluoromethanesulfonic acid (40%) was added and the reaction mixture stirred at 60 °C for 16 h. The mixture was partitioned between ethyl acetate/saturated aqueous sodium hydrogen carbonate solution (the added volume of hydrogen carbonate solution is equal to the total reaction volume). The combined organic layers were dried over magnesium sulfate, filtered and concentrated *in vacuo*.

In the case of C2-CO_2H, the reaction mixture was concentrated *in vacuo* and the crude material purified by column chromatography (silica) without previous work-up.

5.2.3. Procedure C: ester hydrolysis

To a solution of the ester (1 eq.) in methanol (4 to 20 mL/mmol, 1:1 with tetrahydrofuran where specified), an aqueous solution of sodium hydroxide (3 to 13 eq., 0.5 to 2.5 M) was added and the reaction mixture was stirred at room temperature (unless otherwise specified) for 1 to 72 h. The mixture was cooled down to room temperature, diluted down with saturated brine (the added volume of brine is equal to the total reaction volume) and then cooled down to 0°C. The mixture was acidified to pH 1 using 6 N hydrogen chloride solution. The aqueous layer was extracted with ethyl acetate or dichloromethane (3 x same volume as the previous mixture of organic solvents and brine), the combined organic layers dried over magnesium sulfate, filtered and concentrated *in vacuo* to afford carboxylic acids.

5.2.4. Procedure D: bromination of the C2-H position of an indole

To a solution of the ester (1 eq.) in tetrahydrofuran:chloroform (10 to 20mL/mmol, 1:1) at 0 °C, pyridinium tribromide (1.1 eq.) was added and the reaction mixture was stirred at room temperature for 2 h. The mixture was quenched with saturated aqueous sodium thiosulfate solution (the added volume of sodium thiosulfate solution is equal to the total reaction volume) and extracted with dichloromethane (3 x same volume as the previous mixture of organic solvents and sodium thiosulfate solution). The combined organic layers were dried over magnesium sulfate, filtered and concentrated *in vacuo*.

5.2.5. Procedure E: amide bond formation

E1: T3P® mediated amide formation

To a solution of the carboxylic acid (1.0 eq), the amine (1 to 3 eq.) and pyridine (3.0 eq.) in ethyl acetate (3 mL/mmol), 1-propanephosphonic anhydride solution (2.0 eq., 50% wt) was added and the resulting mixture stirred at room temperature for 16 h. The mixture was washed with saturated aqueous sodium carbonate solution (3 x same volume as the previous mixture of organic solvents and sodium thiosulfate solution) and saturated brine (1 x same volume as the sodium carbonate solution), dried over sodium sulfate and concentrated *in vacuo*.

E2: HCTU mediated amide formation

To a solution of the carboxylic acid (1 eq.), DIPEA (3 eq.) and 2-(2-aminoethyl)pyridine (1.1 to 3 eq.) in dimethylformamide (3 to 10 mL/mmol) at 0°C or room temperature, HCTU (1.1 to 3 eq.) was added and the reaction mixture was stirred at room temperature for 16 h. The mixture was diluted down with ethyl acetate (the added volume of ethyl acetate is equal to the total reaction volume) and washed with saturated aqueous sodium hydrogen carbonate solution (3 x same volume as the added ethyl acetate) and saturated brine (1 x same volume as the added ethyl acetate). The organic layer was dried over magnesium sulfate, filtered and concentrated *in vacuo*.

E3: Acyl chloride mediated amide formation

To a suspension of the carboxylic acid (1 eq.) in dichloromethane (50 mL/mmol) at 0°C, Ghosez's reagent (2 to 6 eq.) was added dropwise while stirring. The reaction mixture was stirred at room temperature until complete consumption of the starting material. The resulting solution was subsequently added to a second solution of the amine (1.2 eq.) and triethylamine (1.2 eq.) in dichloromethane (3 mL/mmol) at 0°C. The reaction mixture was stirred at room temperature for 16 h. The mixture was diluted down with ethyl acetate (the added volume of ethyl acetate is equal to the total reaction volume) and washed with saturated aqueous sodium hydrogen carbonate solution (3 x same volume as the added ethyl acetate) and saturated brine (1 x same volume as the added ethyl acetate). The organic layer was dried over magnesium sulfate, filtered and concentrated *in vacuo*.

5.2.6. Procedure F: aza-Diels-Alder cycloaddition

Aniline derivative (2.5 to 3 mmol) was dissolved in dry acetonitrile (2 mL) and ethyl 2-oxoacetate (1.1 eq., 50% solution in toluene) was added. The mixture was stirred for 30 min under nitrogen atmosphere at room temperature. Then, cyclopentadiene (1.5 eq.) and cooper (II) or ytterbium (III) trifluoromethansulfonate.(5%) were added. The mixture was stirred at room temperature until consumption of the aniline. The mixture was diluted with ethyl acetate (10 mL) and the combined organic phases were washed with saturated aqueous sodium hydrogen carbonate solution (2 x 15 mL). The organic layer was dried over magnesium sulfate, filtered and concentrated *in vacuo*.

5.3. Synthetic schemes

Compounds were synthesized in general using one of four synthetic routes (Fig 4a, Scheme S1-S3). In the subsequent section (5. Synthesis and characterization), for completeness, data are reported for all compounds prepared in the study, a number of which had poor solubility and were not screened.

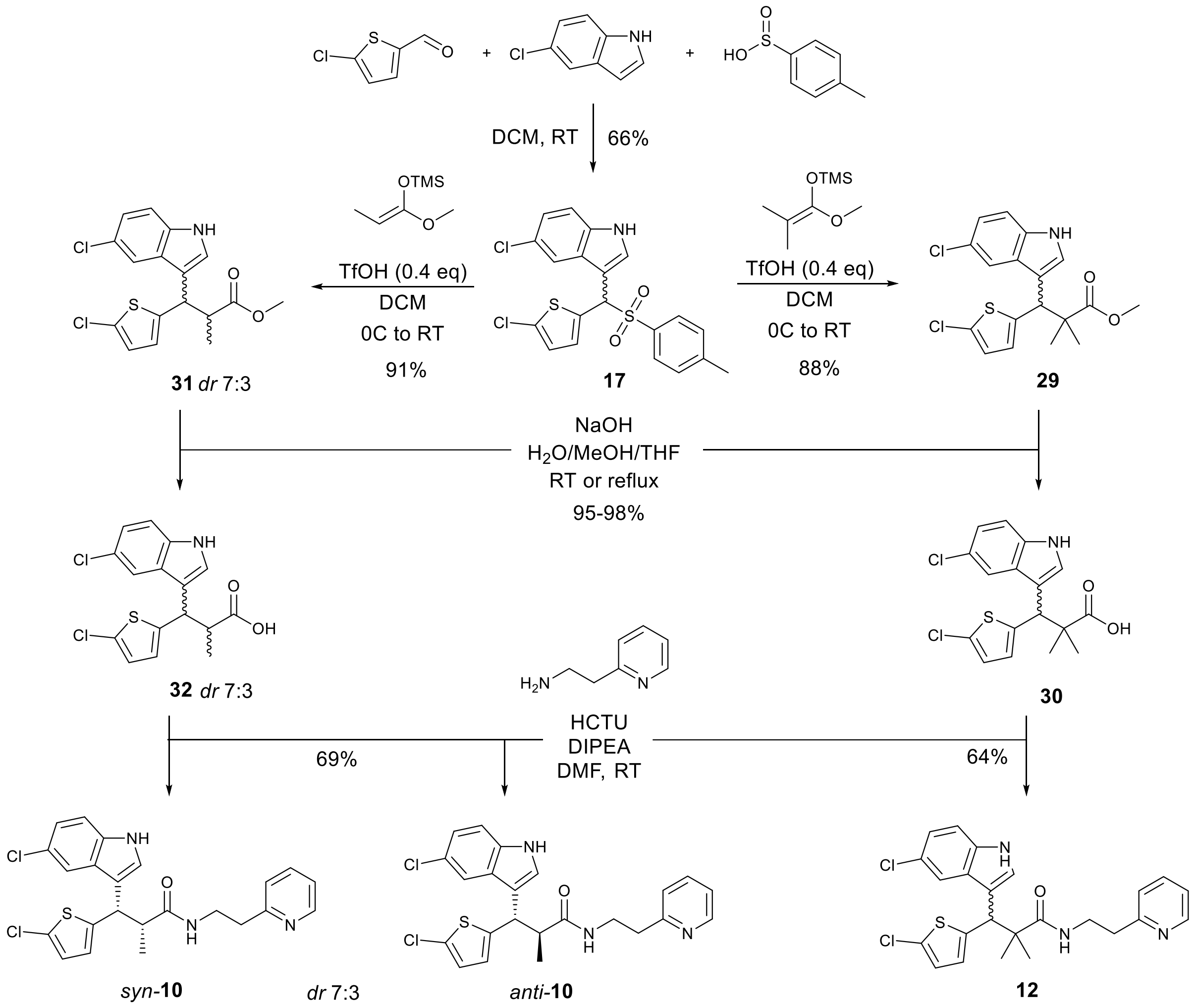

***Scheme S1**. Synthetic approach to prepare C2-H indole analogues. Example of synthetic route to analogues syn-**10**, anti-**10** and **12**.*

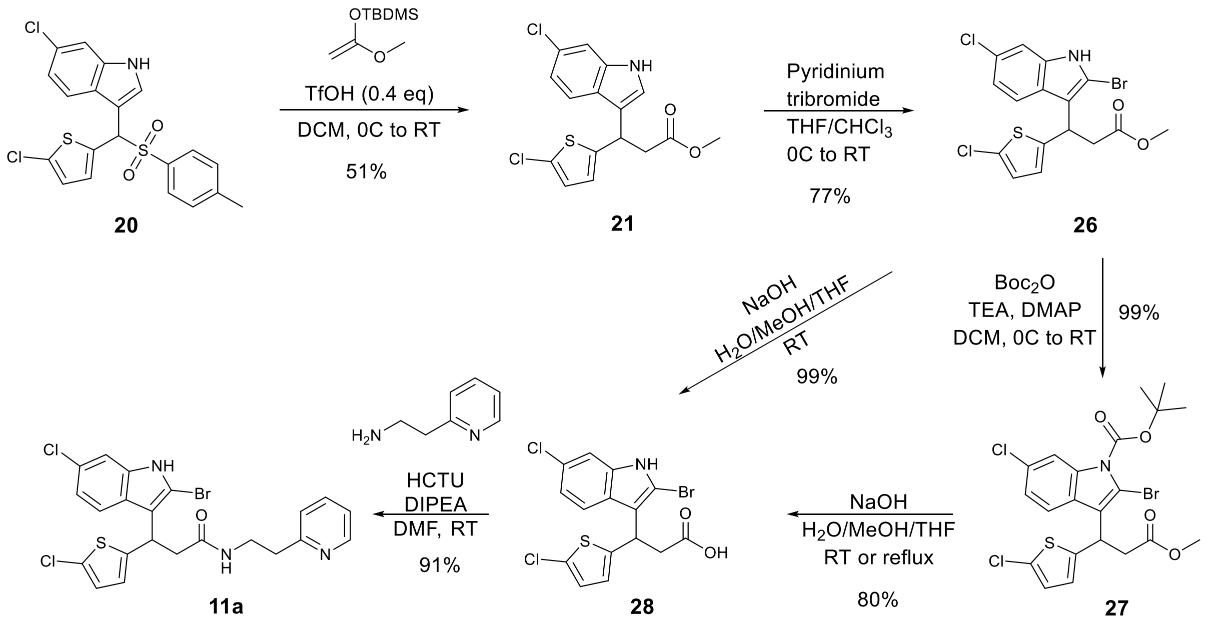

Scheme S2. *Synthetic approach to prepare C2-bromo indole analogues. Example of synthetic route to analogue **11a**.*

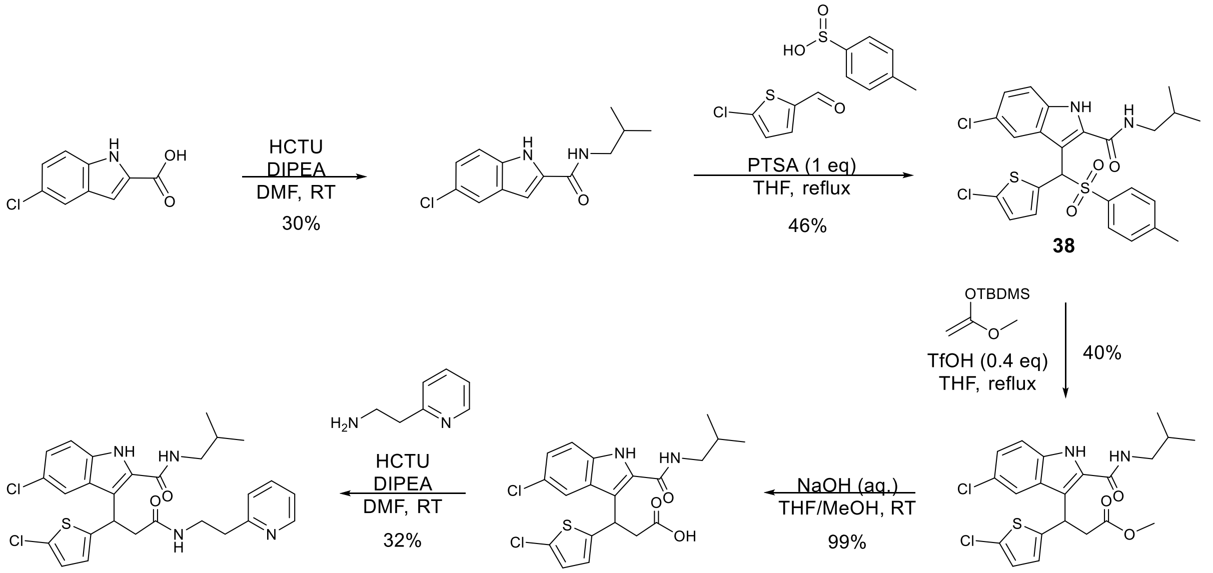

Scheme S3. *Synthetic approach to prepare aromatic and aliphatic C2-amide indole analogues. Example of synthetic route to analogues **11i**. Intermediates without numbering are commercially available or previously reported.*

6. Synthesis and characterisation

Ethyl (3aS,4R,9bR)-8-sulfamoyl-3H,3aH,4H,5H,9bH-cyclopenta[c]quinoline-4-carboxylate 16

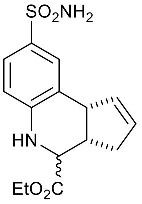

According to general procedure F, 4-aminobenzene-1-sulfonamide (0.50 g, 2.9 mmol), ethyl 2-oxoacetate 50% toluene solution (0.64 mL, 3.2 mmol), cyclopentadiene (0.36 mL, 4.4 mmol) and ytterbium (III) trifluoromethansulfonate (90 mg, 0.15 mmol) in acetonitrile (4 mL) at RT gave the crude product. Purification by column chromatography (silica, eluent: gradient 0:100 → 40:60 of ethyl acetate:hexane) afforded *ester* **16** as a pink solid (526 mg, 56%). δ_H (500 MHz, chloroform-d) 7.56 (1 H, dd, *J* 2.2 and 1.0, 9-H), 7.51 (1 H, app ddd, *J* 8.5, 2.2 and 0.6, 7-H), 6.66 (1 H, d, *J* 8.5, 6-H), 5.81–5.76 (1 H, m, 2-H), 5.71–5.65 (1 H, m, 1-H), 4.74–4.70 (1H, m, NH), 4.67 (2H, s, NH_2), 4.39–4.28 (1 H, m, ethyl 1-H_A), 4.31–4.19 (1 H, m, ethyl 1-H_B), 4.17 (1 H, app dd, *J* 3.6 and 1.0, 4-H), 4.13–4.05 (1 H, m, 9b-H), 3.40–3.31 (1 H, m, 3a-H), 2.46–2.37 (1 H, m, 3-H_A), 2.37–2.29 (1 H, m, 3-H_B) and 1.33 (3 H, t, *J* 7.1, ethyl 2-H_3). δ_C (125 MHz, chloroform-d) 171.2, 147.8, 133.6, 130.7, 130.5, 127.7, 125.5, 125.4, 115.2, 61.7, 55.4, 45.7, 40.6, 32.4 and 14.3. HRMS *m/z* calculated for $C_{13}H_{14}N_2NaO_4S$ $(M+Na)^+$: 345.0885; Found: 345.0879.

(3a*S*,4*R*,9b*R*)-8-Sulfamoyl-3H,3aH,4H,5H,9bH-cyclopenta[c]quinoline-4-carboxylic acid (Z-1)

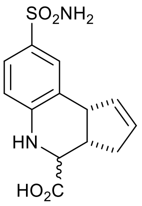

According to general procedure C, ethyl ester **16** (50 mg, 0.16 mmol) and sodium hydroxide solution (2 mL, 0.5 M) in methanol (5 mL) at RT afforded *acid* **Z-1** as white solid (41 mg, 90%). δ_H (500 MHz, deuterium oxide) 7.40 – 7.34 (1 H, m, 9-H), 7.27 (1 H, dd, *J* 8.4 and 2.2, 7-H), 6.64 (1 H, d, *J* 8.4, 6-H), 5.72 – 5.66 (1 H, m, 1-H), 5.62 – 5.56 (1 H, m, 2-H), 4.03 – 3.94 (1 H, m, 4-H), 3.77 – 3.70 (1 H, m, 9b-H), 3.14 – 3.06

(1 H, m, 3a-H) and 2.24 – 2.13 (2 H, m, 3-H_2). HRMS *m/z* calculated for $C_{13}H_{14}N_2NaO_4S$ $(M+Na)^+$: 317.0572; Found: 317.0564.

9-Nitro-3a,4,5,9b-tetrahydro-3H-cyclopenta[c]quinoline-4,6-dicarboxylic Acid Z-2

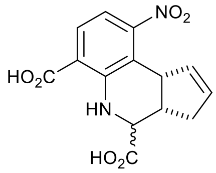

Was synthesized as previously described – characterization matched literature values[35]

5-Chloro-3-[(5-chlorothiophen-2-yl)(4-methylbenzenesulfonyl)methyl]-1H-indole

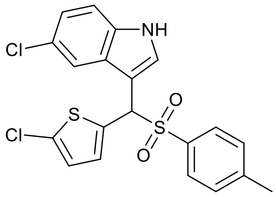

According to general procedure A1, 5-chloroindole (1.0 g, 6.5 mmol), 5-chloro-2-thiophenecarboxaldehyde (0.63 mL, 5.9 mmol) and *p*-toluenesulfinic acid (2.0 g, 13 mmol) in dichloromethane (30 mL) gave the crude product. The product was precipitated in cold diethyl ether (30 mL), filtered, washed with diethyl ether (3 x 10 mL) and dried *in vacuo* to afford *indole* **17** as a light red solid (1.8 g, 66%); δ_H (400 MHz, DMSO-d_6) 11.56 (1 H, d, *J* 2.8, NH), 7.71 (1 H, d, *J* 2.0, 4-H), 7.65 (1 H, d, *J* 2.8, 2-H), 7.58 (2 H, d, *J* 8.1, benzenesulfonyl 2- and 6-H), 7.38 (1 H, d, *J* 8.7, 7-H), 7.29 (2 H, d, *J* 8.1, benzenesulfonyl 3- and 5-H), 7.08 (1 H, dd, *J* 8.7 and 2.0, 6-H), 7.03–6.98 (2 H, m, thiophenyl 3- and 4-H), 6.66 (1 H, s, CHS) and 2.33 (3H, s, benzenesulfonyl 4-methyl). δ_C (100 MHz, DMSO-d_6) 144.8, 135.4, 135.0, 134.4, 130.1, 129.8 (x2), 129.6, 129.1 (x2), 128.3, 128.2, 127.0, 124.5, 122.1, 118.8, 113.7, 106.3, 62.8 and 21.5. HRMS *m/z* calculated for $C_{13}H_8Cl_2NS$ $(M-SO_2Tol)^+$: 279.9749; Found: 279.9662. IR (film): 3384, 1436, 1287, 1135.

Methyl 3-(5-chloro-1H-indol-3-yl)-3-(5-chlorothiophen-2-yl)propanoate

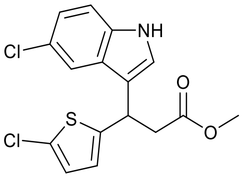

According to general procedure B1, indole **17** (600 mg, 1.4 mmol), tert-butyl((1-methoxyvinyl)oxy)dimethylsilane (0.98 mL, 4.5 mmol) and trifluoromethanesulfonic acid (6.0 µL, 0.07) in dichloromethane (15 mL) gave the crude product. Purification by column chromatography (silica, eluent: gradient 0:100 → 20:80 of ethyl acetate:hexane) afforded *ester* **18** as a colourless oil (400 mg, 76%); δ_H (400 MHz, chloroform-d) 8.11 (1 H, s, NH), 7.45 (1 H, d, *J* 1.9, indolyl 4-H), 7.27 (1 H, d, *J* 8.1, indolyl 7-H), 7.17–7.10 (2 H, m, indolyl 2- and 6-H), 6.70 (1 H, d, *J* 3.8, thiophenyl 4-H), 6.68 (1 H, dd, *J* 3.8 and 0.9, thiophenyl 3-H), 4.91 (1 H, app. t, *J* 7.7, 3-H), 3.64 (3 H, s, methyl), 3.12 (1 H, dd, *J* 14.3 and 6.4, 2-H_A) and 3.06 (1 H, dd, *J* 14.3 and 6.7, 2-H_B). δ_C (100 MHz, chloroform-d) 171.8, 146.5, 134.9, 128.3, 127.3, 125.8, 125.7, 123.7, 123.0, 122.9, 118.8, 117.6, 112.5, 52.0, 41.7 and 34.8. HRMS *m/z* calculated for $C_{16}H_{13}Cl_2NNaO_2S$ $(M+Na)^+$: 375.9942; Found: 375.9734.

3-(5-Chloro-1H-indol-3-yl)-3-(5-chlorothiophen-2-yl)propanoic acid

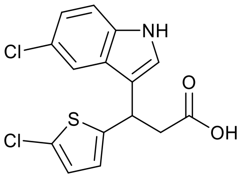

According to general procedure C, ester **18** (580 mg, 1.6 mmol) and sodium hydroxide solution (6 mL, 1.3 M) in methanol (6 mL) afforded *acid* **19** as a light brown oil (539 mg, 96%); δ_H (400 MHz, chloroform-d) 8.11 (1 H, s, NH), 7.44 (1 H, d, *J* 1.9, indolyl 4-H), 7.27 (1 H, d, *J* 8.7, indolyl 7-H), 7.17–7.12 (2 H, m, indolyl 2- and 6-H), 6.72–6.68 (2 H, m, thiophenyl 3- and 4-H), 4.89 (1 H, app. t, *J* 7.6, 3-H), 3.15 (1 H, dd, *J* 15.8 and 7.2, 2-H_A) and 3.09 (1 H, dd, *J* 15.8 and 7.6, 2-H_B). δ_C (125 MHz, chloroform-d) 176.4, 146.4, 135.0, 128.6, 127.4, 126.0, 125.9, 123.9, 123.2, 123.0, 118.9, 117.4, 112.6, 41.5 and 34.7. HRMS *m/z* calculated for $C_{15}H_{10}Cl_2NO_2S$ $(M-H)^-$: 337.9815; Found: 337.9830. IR (film): 3271 (broad), 1720.

3-(5-Chloro-1H-indol-3-yl)-3-(5-chlorothiophen-2-yl)-*N*-[2-(pyridin-2-yl)ethyl] propanamide A1

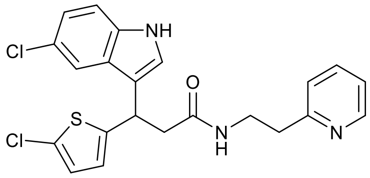

According to general procedure E1, carboxylic acid **19** (100 mg, 0.30 mmol), 2-(2-pyridyl)ethylamine (0.11 mL, 0.90 mmol), pyridine (0.10 mL, 1.2 mmol) and T3P (0.35 mL, 0.6 mmol) in ethyl acetate (3 mL) gave the crude product. Purification by column chromatography (silica, eluent: gradient 0:100 → 20:80 of ethyl acetate:hexane) afforded *amide* **A1** as a colourless oil which solidified on standing (51 mg, 40%); δ_H (500 MHz, DMSO-d$_6$) 11.17 (1 H, d, *J* 2.6, NH$_{indole}$), 8.47 (1 H, ddd, *J* 4.9, 1.9 and 0.9, pyridinyl 6-H), 8.02 (1 H, t, *J* 5.7, NH$_{amide}$), 7.62 (1 H, td, *J* 7.7 and 1.9, pyridinyl 4-H), 7.39 (1 H, d, *J* 2.1, indolyl 4-H), 7.37 (1 H, d, *J* 8.6, indolyl 7-H), 7.35 (1 H, d, *J* 2.6, indolyl 2-H), 7.19 (1 H, ddd, *J* 7.7, 4.9 and 1.2, pyridinyl 5-H), 7.06 (1 H, dd, *J* 8.6 and 2.1, indolyl 6-H), 7.06–7.04 (1 H, m, pyridinyl 3-H), 6.88 (1 H, d, *J* 3.8, thiophenyl 4-H), 6.79 (1 H, dd, *J* 3.8 and 0.9, thiophenyl 3-H), 4.86 (1 H, app. t, *J* 7.7, 3-H), 3.41–3.33 (2 H, m, pyridinylethyl 1-H$_2$), 2.90–2.79 (2 H, m, 2-H$_2$) and 2.74 (2 H, t, *J* 7.2, pyridinylethyl 2-H$_2$). δ_C (125 MHz, DMSO-d$_6$) 169.7, 159.0, 149.0, 148.8, 136.3, 134.9, 127.1, 126.2, 125.5, 124.1, 123.6, 123.2, 123.0, 121.4, 121.2, 117.9, 116.4, 113.2, 42.3, 38.4, 37.3 and 34.3. HRMS *m/z* calculated for $C_{22}H_{19}Cl_2N_3NaOS$ $(M+Na)^+$: 466.0518; Found: 466.0512. IR (film): 3340, 1629.

3-(5-Chloro-1H-indol-3-yl)-3-(5-chlorothiophen-2-yl)-*N*-(2-phenylethyl) propanamide 7b

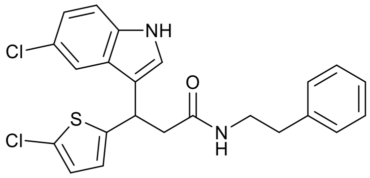

By general procedure E1, carboxylic acid **19** (50 mg, 0.15 mmol), 2-phenylethylamine (60 µL, 0.48 mmol), pyridine (50 µL, 0.6 mmol) and T3P (175 µL, 0.3 mmol) in ethyl acetate (0.10 mL) gave the crude product. Purification by column chromatography (silica, Eluent: gradient 0:100 → 20:80 of ethyl acetate:hexane) afforded *amide* **7b** as a light brown oil which solidified on standing (23 mg, 36%); δ_H (400 MHz, chloroform-d) 8.51 – 8.33 (1 H, m, NH$_{indole}$), 7.42 (1 H, d, *J* 2.0, indolyl 4-H), 7.36–7.15 (4 H, m, indolyl 7-H and phenyl 3-, 4- and 5-H), 7.14 (1 H, dd, *J* 8.6 and 2.0, indolyl 6-H), 7.05

(1 H, d, *J* 2.5, indolyl 2-H), 6.93 (2 H, m, phenyl 2- and 6-H), 6.69 (1 H, d, *J* 3.8, thiophenyl 4-H), 6.64 (1 H, d, *J* 3.8, thiophenyl 3-H), 5.53–5.34 (1 H, m, NH_{amide}), 4.91 (1 H, t, *J* 7.5, 3-H), 3.47–3.34 (2 H, m, pyridinylethyl 1-H_2), 2.95–2.76 (2 H, m, 2-H_2) and 2.68–2.49 (2 H, m, pyridinylethyl 2-H_2). δ_C (100 MHz, chloroform-d) 170.7, 146.7, 138.7, 135.1, 128.9, 128.77, 128.75, 128.2, 127.1, 126.7, 126.6, 125.8, 125.6, 123.8, 123.5, 122.9, 119.0, 117.1, 112.7, 43.9, 40.7, 35.60 and 35.57. HRMS *m/z* calculated for $C_{23}H_{20}Cl_2N_2NaO_2S$ $(M+Na)^+$: 465.0566; Found: 465.0562. IR (film): 3416, 3275, 1644.

***N*-Benzyl-3-(5-chloro-1H-indol-3-yl)-3-(5-chlorothiophen-2-yl)propenamide 7c**

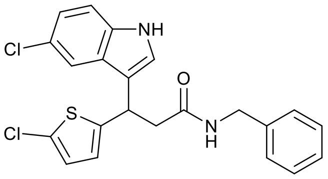

According to general procedure E2, carboxylic acid **19** (20 mg, 0.38 mmol), 1-phenylmethanamine (20 µL, 0.18 mmol), DIPEA (35 µL, 0.18 mmol) and HCTU (50 mg, 0.12 mmol) in dimethylformamide (2 mL) at RT gave the crude product. Purification by column chromatography (silica, eluent: gradient 0:100 → 50:50 of ethyl acetate:hexane) afforded *amide* **7c** as a white solid (25 mg, 96%). δ_H (500 MHz, chloroform-d) 8.25 (1 H, s, NH_{indole}), 7.44 (1 H, d, *J* 2.0, indolyl 4-H), 7.31–7.25 (1 H, m, indolyl 7-H), 7.24–7.20 (3 H, benzyl 4-H, 5-H and 6-H), 7.14 (1 H, dd, *J* 8.6 and 2.0, indolyl 6-H), 7.04 (1 H, d, *J* 2.5, indolyl 2-H), 6.95–6.90 (2 H, m, benzyl 3-H and 7-H), 6.70 (1 H, d, *J* 3.8, thiophenyl 4-H), 6.66 (1 H, dd, *J* 3.8 and 1.0, thiophenyl 3-H), 5.71–5.62 (1 H, m, NH_{amide}), 4.94 (1 H, tt, *J* 7.7 and 0.9, 3-H), 4.32 (2 H, d, *J* 5.8, benzyl 1-H_2), 3.00 (1 H, dd, *J* 13.9 and 7.7, 2-H_A) and 2.93 (1 H, dd, *J* 13.9 and 7.7, 2-H_B). δ_C (125 MHz, chloroform-d) 170.4, 146.6, 137.9, 135.1, 128.7 (x2), 128.3, 127.6 (x3), 127.1, 125.9, 125.7, 124.0, 123.5, 123.0, 118.9, 117.1, 112.6, 44.1, 43.6 and 35.7. HRMS *m/z* calculated for $C_{22}H_{19}Cl_2N_2OS$ $(M+H)^+$: 429.0595; Found: 429.0582.

3-(5-Chloro-1H-indol-3-yl)-3-(5-chlorothiophen-2-yl)-*N*-(2-methoxyethyl) propanamide 7d

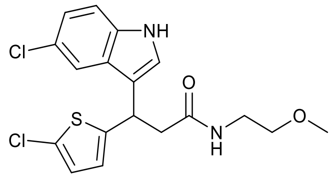

According to general procedure E1, carboxylic acid **19** (50 mg, 0.15 mmol), 2-methoxyethylamine (14 µL, 0.16 mmol), pyridine (50 µL, 0.6 mmol) and T3P (175 µL, 0.3 mmol) in ethyl acetate (0.10 mL) gave the crude product. Purification by column chromatography (silica, eluent: gradient 0:100 → 20:80 of ethyl acetate:hexane) afforded *amide* **7d** as a light brown oil which solidified on standing (53 mg, 90%); δ_H (400 MHz, chloroform-d) 8.71–8.60 (1 H, m, NH_{indole}), 7.43 (1 H, d, *J* 2.0, indolyl 4-H), 7.23 (1 H, d, *J* 8.6, indolyl 7-H), 7.10 (1 H, dd, *J* 8.6 and 2.0, indolyl 6-H), 7.04 (1 H, d, *J* 2.6, indolyl 2-H), 6.68 (1 H, d, *J* 3.8, thiophenyl 4-H), 6.64 (1 H, dd, *J* 3.8 and 1.0, thiophenyl 3-H), 5.95–5.86 (1 H, m, NH_{amide}), 4.92 (1 H, app. t, *J* 7.5, 3-H), 3.42–3.23 (4 H, m, methoxyethyl 1- and 2-H_2), 3.22 (3 H, s, methoxy), 2.95 (1 H, dd, *J* 14.1 and 7.3, 2-H_A) and 2.88 (1 H, dd, *J* 14.1 and 7.9, 2-H_B). δ_C (125 MHz, chloroform-d) 170.7, 146.8, 135.0, 128.2, 127.2, 125.8, 125.5, 123.8, 123.3, 122.8, 118.9, 117.3, 112.6, 71.1, 58.8, 44.0, 39.3 and 35.5. HRMS *m/z* calculated for $C_{18}H_{18}Cl_2N_2NaO_2S$ $(M+Na)^+$: 419.0358; Found: 419.0356. IR (film): 3420, 3286, 1645.

6-Chloro-3-[(5-chlorothiophen-2-yl)(4-methylbenzenesulfonyl)methyl]-1H-indole

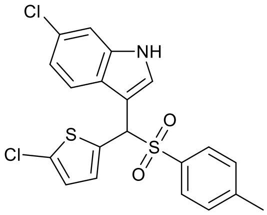

By general procedure A1, 6-chloroindole (0.94 g, 6.2 mmol), 5-chloro-2-thiophenecarboxaldehyde (0.60 mL, 5.6 mmol) and *p*-toluenesulfinic acid (2.0 g, 13 mmol) in dichloromethane (30 mL) gave the crude product. The product was precipitated in cold diethyl ether (30 mL), filtered, washed with diethyl ether (3 x 10 mL) and dried *in vacuo* to afford *indole* **20** as a light pink solid (1.3 g, 49%); δ_H (400 MHz, DMSO-d_6) 11.46 (1 H, d, *J* 2.8, NH), 7.70 (1 H, d, *J* 8.6, 4-H), 7.61–7.54 (3 H, m, 2-H and benzenesulfonyl 2- and 6-H), 7.40 (1 H, d, *J* 1.9, 7-H), 7.28 (2 H, app. d, *J* 8.0, benzenesulfonyl 3- and 5-H), 7.04–6.96 (3 H, m, 5-H, thiophenyl 3- and 4-H), 6.63

(1 H, s, CHS) and 2.31 (3 H, s, benzenesulfonyl 4-methyl). δ_C (125 MHz, DMSO-d_6) 144.4, 134.9, 134.5, 133.9, 129.6, 129.3 (x2), 129.2, 128.6 (x2), 127.80, 127.75, 126.5, 124.0, 121.6, 118.3, 113.2, 105.8, 62.3 and 21.0. HRMS *m/z* calculated for $C_{13}H_8Cl_2NS$ (M-SO_2Tol)$^+$: 279.9749; Found: 279.9658. IR (film): 3387, 1437, 1283, 1143, 1135.

Methyl 3-(6-chloro-1H-indol-3-yl)-3-(5-chlorothiophen-2-yl)propanoate

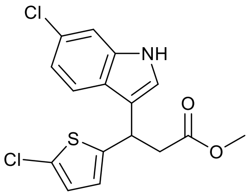

According to general procedure B1, indole **20** (1.0 g, 2.3 mmol), tert-butyl((1-methoxyvinyl)oxy)dimethylsilane (1.7 mL, 7.8 mmol) and trifluoromethanesulfonic acid (11.0 µL, 0.12 mmol) in dichloromethane (25 mL) gave the crude product. Purification by column chromatography (silica, eluent: gradient 0:100 → 20:80 of ethyl acetate:hexane) afforded *ester* **21** as a light yellow oil (0.41 g, 51%); δ_H (400 MHz, chloroform-d) 8.10 (1 H, app. s, NH), 7.38 (1 H, d, *J* 8.5, indolyl 4-H), 7.34 (1 H, d, *J* 1.8, indolyl 7-H), 7.09 (1 H, d, *J* 2.4, indolyl 2-H), 7.05 (1 H, dd, *J* 8.5 and 1.8, indolyl 5-H), 6.69 (1 H, d, *J* 3.8, thiophenyl 4-H), 6.67 (1 H, dd, *J* 3.8 and 0.9, thiophenyl 3-H), 4.93 (1 H, app. t, *J* 7.7, 3-H), 3.64 (3 H, s, methyl) and 3.17–3.01 (2 H, m, 2-H_2). δ_C (100 MHz, chloroform-d) 171.8, 146.5, 134.9, 128.3, 127.3, 125.8, 125.7, 123.7, 123.0, 122.9, 118.8, 117.6, 112.5, 52.0, 41.7 and 34.8. HRMS *m/z* calculated for $C_{16}H_{13}Cl_2NNaO_2S$ (M+Na)$^+$: 375.9942; Found: 375.9932.

3-(6-Chloro-1H-indol-3-yl)-3-(5-chlorothiophen-2-yl)propanoic acid

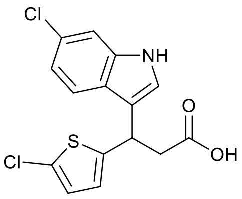

According to general procedure C, ester **21** (43 mg, 0.12 mmol) and sodium hydroxide solution (1 mL, 0.5 M) in methanol:tetrahydrofuran (2 mL) afforded *acid* **22** as a brown solid (40 mg, 99%). δ_H (500 MHz, methanol-d_4) 7.49–7.43 (1 H, m, indolyl 4-H), 7.31 (1 H, dd, J 2.0 and 0.8, indolyl 7-H), 7.08–7.03 (1 H, m, indolyl 2-H), 6.98 (1 H, ddd, J 8.6, 2.0 and 0.8, indolyl 5-H), 6.72–6.64 (2 H, m, thiophenyl 3- and 4-H), 5.23–5.16 (1

H, m, 3-H), 3.34 (1 H, dd, J 15.7 and 7.9, 2-H_A) and 3.06 (1 H, dd, J 15.7 and 7.5, 2-H_B). δ_C (125 MHz, methanol-d_4) 174.9, 148.4, 137.6, 134.0, 129.9, 129.8, 127.0, 126.7, 126.6, 124.3, 121.8, 121.0, 112.1, 40.9 and 36.2. HRMS *m/z* calculated for $C_{15}H_{10}Cl_2NO_2S$ $(M-H)^-$: 337.9815; Found: 337.9941.

3-(6-Chloro-1H-indol-3-yl)-3-(5-chlorothiophen-2-yl)-*N*-[2-(pyridin-2-yl)ethyl] propanamide 8a

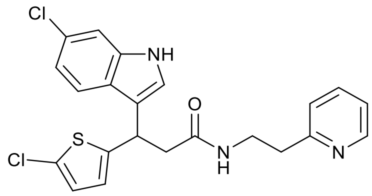

According to general procedure E1, carboxylic acid **22** (50 mg, 0.15 mmol), 2-(2-pyridyl)ethylamine (20 µL, 0.16 mmol), pyridine (50 µL, 0.6 mmol) and T3P (175 µL, 0.3 mmol) in ethyl acetate (0.10 mL) gave the crude product. Purification by column chromatography (silica, eluent: gradient 0:100 → 20:80 of ethyl acetate:hexane) afforded *amide* **8a** as a colourless solid (32 mg, 49%); δ_H (500 MHz, DMSO-d_6) 11.30 (1 H, app. s, NH_{indole}), 8.47 (1 H, dd, *J* 5.0 and 1.8, pyridinyl 6-H), 8.17 (1 H, br s, NH_{amide}), 7.63 (1 H, td, *J* 7.6 and 1.8, pyridinyl 4-H), 7.41 (1 H, d, *J* 1.9, indolyl 7-H), 7.38 (1 H, d, *J* 8.5, indolyl 4-H), 7.33 (1 H, d, *J* 2.4, indolyl 2-H), 7.19 (1 H, dd, *J* 7.6 and 5.0, pyridinyl 5-H), 7.08 (1 H, d, *J* 7.6, pyridinyl 3-H), 6.95 (1 H, dd, *J* 8.5 and 1.9, indolyl 5-H), 6.86 (1 H, d, *J* 3.8, thiophenyl 4-H), 6.77 (1 H, d, *J* 3.8, thiophenyl 3-H), 4.87 (1 H, app. t, *J* 7.7, 3-H), 3.44–3.28 (2 H, m, pyridinylethyl 1-H_2), 2.92–2.80 (2 H, m, 2-H_2) and 2.75 (2 H, t, *J* 7.2, pyridinylethyl 2-H_2). δ_C (125 MHz, DMSO-d_6) 169.8, 159.0, 149.0, 148.9, 136.8, 136.3, 126.1, 125.9, 125.5, 124.8, 123.6, 123.3, 123.0, 121.4, 120.0, 118.8, 116.7, 111.2, 42.4, 38.4, 37.3 and 34.3. HRMS *m/z* calculated for $C_{22}H_{19}Cl_2N_3NaOS$ $(M+Na)^+$: 466.0518; Found: 466.0512. IR (film): 3340, 1630.

3-(6-Chloro-1H-indol-3-yl)-3-(5-chlorothiophen-2-yl)-*N*-(2-phenylethyl) propanamide 8b

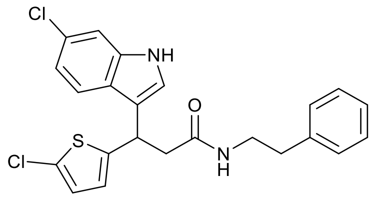

According to general procedure E1, carboxylic acid **22** (50 mg, 0.15 mmol), 2-phenylethylamine (20 µL, 0.16 mmol), pyridine (50 µL, 0.6 mmol) and T3P (175 µL,

0.3 mmol) in ethyl acetate (0.10 mL) gave the crude product. Purification by column chromatography (silica, eluent: gradient 0:100 → 20:80 of ethyl acetate:hexane) afforded *amide* **8b** as a light purple oil which solidified on standing (62 mg, 95%); δ_H (400 MHz, chloroform-d) 8.60 (1 H, s, NH_{indole}), 7.38 (2 H, m, indolyl 4- and 7-H), 7.34–7.20 (3 H, m, phenyl 3-, 4- and 5-H), 7.08–7.03 (2 H, m, indolyl 2- and 5-H), 7.01–6.94 (2 H, m, phenyl 2- and 6-H), 6.73 (1 H, d, *J* 3.8, thiophenyl 4-H), 6.68 (1 H, d, *J* 3.8, thiophenyl 3-H), 5.50 (1 H, t, *J* 6.3, NH_{amide}), 4.97 (1 H, t, *J* 7.5, 3-H), 3.45 (2 H, q, *J* 6.3, pyridinylethyl 1-H_2), 2.94 (1 H, dd, *J* 14.0 and 7.5, 2-H_A), 2.86 (1 H, dd, *J* 14.0 and 7.5, 2-H_B) and 2.63 (2 H, m, pyridinylethyl 2-H_2). δ_C (100 MHz, chloroform-d) 170.7, 146.9, 138.7, 137.1, 128.84, 128.80, 128.75, 128.7, 128.4, 128.2, 126.6, 125.8, 124.7, 123.8, 122.6, 120.5, 120.4, 117.5, 111.5, 44.0, 40.7, 35.61 and 35.57. HRMS *m/z* calculated for $C_{23}H_{20}Cl_2N_2NaO_2S$ $(M+Na)^+$: 465.0566; Found: 465.0560. IR (film): 3387, 3279, 1631.

3-(6-Chloro-1H-indol-3-yl)-3-(5-chlorothiophen-2-yl)-*N*-(2-methoxyethyl) propanamide 8c

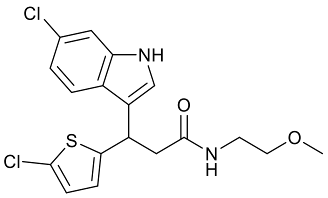

According to general procedure E1, carboxylic acid **22** (50 mg, 0.15 mmol), 2-methoxyethylamine (20 µL, 0.23 mmol), pyridine (50 µL, 0.6 mmol) and T3P (175 µL, 0.3 mmol) in ethyl acetate (0.10 mL) gave the crude product. Purification by column chromatography (silica, Eluent: gradient 0:100 → 20:80 of ethyl acetate:hexane) afforded *amide* **8c** as a light purple oil which solidified on standing (61 mg, 99%); δ_H (400 MHz, chloroform-d) 8.60 (1 H, app. s, NH_{indole}), 7.35 (1 H, d, *J* 8.5, indolyl 4-H), 7.32 (1 H, d, *J* 1.9, indolyl 7-H), 7.03 (1 H, d, *J* 2.4, indolyl 2-H), 7.01 (1 H, dd, *J* 8.5 and 1.9, indolyl 5-H), 6.68 (1 H, d, *J* 3.7, thiophenyl 4-H), 6.66–6.63 (1 H, m, thiophenyl 3-H), 5.96–5.85 (1 H, m, NH_{amide}), 4.93 (1 H, app. t, *J* 7.6, 3-H), 3.40–3.19 (4 H, m, methoxyethyl 1- and 2-H_2), 3.22 (3 H, s, methoxy), 2.96 (1 H, dd, *J* 14.0 and 7.4, 2-H_A) and 2.88 (1 H, dd, *J* 14.0 and 7.9, 2-H_B). δ_C (100 MHz, chloroform-d) 170.9, 146.9, 137.1, 128.4, 128.1, 125.7, 124.8, 123.8, 122.5, 120.5, 120.3, 117.6, 111.5, 71.0, 58.7, 44.0, 39.4 and 35.5. HRMS *m/z* calculated for $C_{18}H_{18}Cl_2N_2NaO_2S$ $(M+Na)^+$: 419.0358; Found: 419.0355. IR (film): 3283, 1644;

5-Chloro-3-[(4-methylbenzenesulfonyl)(phenyl)methyl]-1H-indole

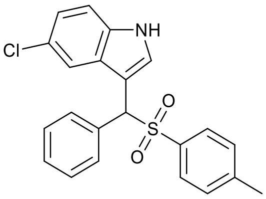

According to general procedure A1, 5-chloroindole (1.0 g, 6.6 mmol), benzaldehyde (0.61 mL, 6.0 mmol) and *p*-toluenesulfinic acid (2.0 g, 13 mmol) in dichloromethane (30 mL) gave the crude product. The product was precipitated in cold diethyl ether (30 mL), filtered, washed with diethyl ether (3 x 10 mL) and dried *in vacuo* to afford indole **23**[1] as a light pink solid (1.3 g, 48%); δ_H (400 MHz, DMSO-d$_6$) 11.46 (1 H, d, *J* 2.7, NH), 7.73 (1 H, d, *J* 2.7, 2-H), 7.68 (1 H, d, *J* 2.0, 4-H), 7.59–7.56 (2 H, m, phenyl 2- and 6-H), 7.56–7.52 (2 H, m, benzenesulfonyl 2- and 6-H), 7.32 (1 H, d, *J* 8.7, 7-H), 7.32–7.22 (3 H, m, phenyl 3-, 4- and 5-H), 7.21 (2 H, app. d, *J* 8.1, benzenesulfonyl 3- and 5-H), 7.02 (1 H, dd, *J* 8.7 and 2.0, 6-H), 6.24 (1 H, s, CHS) and 2.26 (3 H, s, benzenesulfonyl 4-methyl). δ_C (100 MHz, DMSO-d$_6$) 143.9, 135.6, 133.9, 133.8, 130.1 (x2), 129.1 (x2), 128.5 (x2), 128.2 (x3), 128.1, 127.2, 123.8, 121.4, 118.0, 113.0, 106.5, 66.0 and 21.0. HRMS *m/z* calculated for $C_{22}H_{22}Cl_2N_2S$ $(M+NH_4)^+$: 413.1080; Found: 413.1085. IR (film): 3356, 1453, 1282, 1138.

Methyl 3-(5-chloro-1H-indol-3-yl)-3-phenylpropanoate

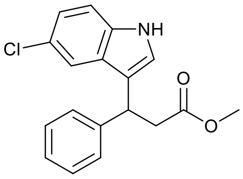

According to general procedure B1, indole **23** (0.50 g, 1.2 mmol), tert-butyl((1-methoxyvinyl)oxy)dimethylsilane (0.80 mL, 3.7 mmol) and trifluoromethanesulfonic acid (6.0 µL, 0.07 mmol) in dichloromethane (15 mL) gave the crude product. Purification by column chromatography (silica, eluent: gradient 0:100 → 20:80 of ethyl acetate:hexane) afforded ester **24**[2] as a light orange solid (0.32 g, 80%); δ_H (400 MHz, chloroform-d) 8.08 (1 H, app. s, NH), 7.37 (1 H, d, *J* 1.9, indolyl 4-H), 7.32–7.27 (4 H, m, phenyl 2-, 3-, 5- and 6-H), 7.24–7.16 (2 H, m, indolyl 7-H and phenyl 4-H), 7.10 (1 H, dd, *J* 8.6 and 1.9, indolyl 6-H), 7.07 (1 H, d, *J* 2.5, indolyl 2-H), 4.75 (1 H, app. t, *J* 7.9, 3-H), 3.60 (3 H, s, methyl), 3.13 (1 H, dd, *J* 15.2 and 8.0, 2-H$_A$) and 3.02 (1 H, dd, *J* 15.2 and 7.8, 2-H$_B$). δ_C (100 MHz, chloroform-d) 172.5, 143.3, 135.0, 128.7 (x3),

127.7 (x2), 126.8, 125.4, 122.7, 122.6, 119.1, 118.7, 112.2, 51.9, 41.4 and 39.2. HRMS *m/z* calculated for $C_{18}H_{16}ClNNaO_2$ $(M+Na)^+$: 336.0762; Found: 336.0756.

3-(5-Chloro-1H-indol-3-yl)-3-phenylpropanoic acid

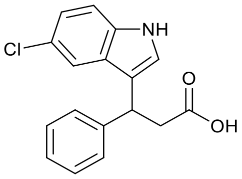

According to general procedure C, ester **24** (100 mg, 0.3 mmol) and sodium hydroxide solution (3 mL, 1.3 M) in methanol (2 mL) afforded acid **25**[3] as a light yellow oil which solidified upon standing (95 mg, 99%); δ_H (400 MHz, methanol-d_4) 7.34 – 7.19 (7 H, m, indolyl 2-, 4- and 7-H and phenyl 2-, 3-, 5- and 6-H), 7.19–7.13 (1 H, m, phenyl 4-H), 7.00 (1 H, dd, *J* 8.6 and 2.1, indolyl 6-H), 4.67 (1 H, app. t, *J* 7.9, 3-H), 3.11 (1 H, dd, *J* 15.3 and 7.8, 2-H_A) and 2.97 (1 H, dd, *J* 15.2 and 7.9, 2-H_B). δ_C (100 MHz, DMSO-d_6) 172.8, 144.5, 134.8, 128.2 (x2), 127.6 (x2), 127.4, 126.1, 123.7, 122.9, 120.9, 117.8, 117.4, 112.9, 54.9 and 38.4. HRMS *m/z* calculated for $C_{17}H_{14}ClNNaO_2$ $(M+Na)^-$: 322.0611; Found: 322.0682.

3-(5-Chloro-1H-indol-3-yl)-3-phenyl-N-[2-(pyridin-2-yl)ethyl]propanamide 9a

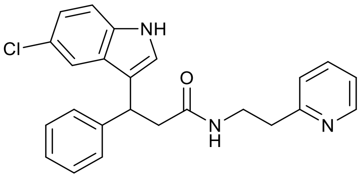

According to general procedure E1, carboxylic acid **25** (50 mg, 0.17 mmol), 2-(2-pyridyl)ethylamine (110 µL, 0.90 mmol), pyridine (50 µL, 0.6 mmol) and T3P (175 µL, 0.3 mmol) in ethyl acetate (3 mL) gave the crude product. Purification by column chromatography (silica, eluent: gradient 0:100 → 20:80 of ethyl acetate:hexane) afforded *amide* **9a** as a colourless oil which solidified on standing (39 mg, 59%); δ_H (500 MHz, chloroform-d) 8.52 – 8.45 (1 H, m, pyridinyl 6-H), 8.37 (1 H, s, NH_{indole}), 7.58 (1 H, td, *J* 7.7 and 1.9, pyridinyl 4-H), 7.39 (1 H, d, *J* 2.0, indolyl 4-H), 7.36–7.18 (6 H, m, indolyl 7-H and phenyl 2- to 6-H), 7.20–7.15 (1 H, m, pyridinyl 5-H), 7.15–7.11 (2 H, m, indolyl 2- and 6-H), 6.98 (1 H, d, *J* 7.7, pyridinyl 3-H), 6.41 (1 H, t, *J* 6.1, NH_{amide}), 4.77 (1 H, app. t, *J* 7.8, 3-H), 3.60 (2 H, q, *J* 6.1, pyridinylethyl 1-H_2), 3.05 (1 H, dd, *J* 14.1 and 8.0, 2-H_A), 2.90 (1 H, dd, *J* 14.1 and 7.6, 2-H_B) and 2.80 (2 H, t, *J*

6.1, pyridinylethyl 2-H_2). δ_C (125 MHz, DMSO-d_6) 170.3, 158.9, 148.9, 144.8, 136.5, 134.8, 128.2 (x2), 127.53 (x2), 127.51, 126.0, 123.7, 123.1, 122.8, 121.4, 120.9, 117.9, 117.5, 112.9, 54.9, 42.2, 37.3 and 30.7. HRMS *m/z* calculated for $C_{24}H_{22}ClN_3NaO$ $(M+Na)^+$: 426.1344; Found: 426.1335. IR (film): 3324, 1628.

3-(5-Chloro-1H-indol-3-yl)-3-phenyl-*N*-(2-phenylethyl)propenamide 9b

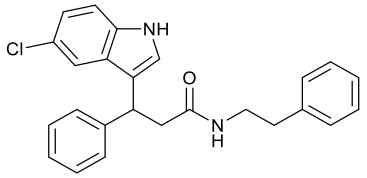

According to general procedure E1, carboxylic acid **25** (50 mg, 0.15 mmol), 2-phenylethylamine (20 µL, 0.16 mmol), pyridine (50 µL, 0.6 mmol) and T3P (175 µL, 0.3 mmol) in ethyl acetate (0.10 mL) gave the crude product. Purification by column chromatography (silica, eluent: gradient 0:100 → 20:80 of ethyl acetate:hexane) afforded *amide* **9b** as a light purple oil which solidified on standing (47 mg, 70%); δ_H (400 MHz, chloroform-d) 8.73 (1 H, app. s, NH_{indole}), 7.40 (1 H, d, *J* 2.0, indolyl 4-H), 7.37–6.93 (11 H, m, indolyl 7-H, phenyl 2- to 6-H and ethylphenyl 2- to 6-H), 7.14 (1 H, dd, *J* 8.6 and 2.0, indolyl 6-H), 7.04 (1 H, d, *J* 2.5, indolyl 2-H), 5.52 (1 H, t, *J* 5.9, NH_{amide}), 4.77 (1 H, app. t, *J* 7.7, 3-H), 3.52–3.32 (2 H, m, phenylethyl 1-H_2), 3.00 (1 H, dd, *J* 14.1 and 7.6, 2-H_A), 2.83 (1 H, dd, *J* 14.1 and 7.8, 2-H_B) and 2.66–2.53 (2 H, m, phenylethyl 2-H_2). δ_C (125 MHz, chloroform-d) 171.2, 143.5, 139.0, 135.1, 128.80 (x2), 128.76 (x2), 128.71 (x2), 127.8 (x3), 126.8, 126.5, 125.4, 123.0, 122.7, 119.3, 118.6, 112.3, 43.8, 40.6, 39.7 and 35.7. HRMS *m/z* calculated for $C_{25}H_{24}ClN_2O$ $(M+H)^+$: 403.1578; Found: 403.1589. IR (film): 3420, 3276, 1645.

***N*-Benzyl-3-(5-chloro-1H-indol-3-yl)-3-phenylpropanamide 9c**

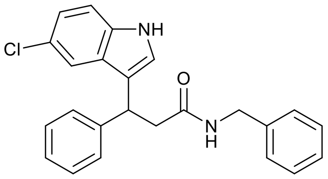

According to general procedure E1, carboxylic acid **25** (50 mg, 0.15 mmol), benzylamine (20 µL, 0.18 mmol), pyridine (50 µL, 0.6 mmol) and T3P (175 µL, 0.3 mmol) in ethyl acetate (0.10 mL) gave the crude product. Purification by column chromatography (silica, eluent: gradient 0:100 → 20:80 of ethyl acetate:hexane)

afforded *amide* **9c** as a colourless oil (49 mg, 69%); δ_H (400 MHz, chloroform-d) 8.66 (1 H, app. s, NH_{indole}), 7.38 (1 H, d, *J* 2.0, indolyl 4-H), 7.32–6.79 (11 H, m, indolyl 7-H, phenyl 2- to 6-H and benzyl 3 to 7-H), 7.08 (1 H, dd, *J* 8.6 and 2.0, indolyl 6-H), 6.95 (1 H, d, *J* 2.5, indolyl 2-H), 5.90 (1 H, app. t, *J* 5.8, NH_{amide}), 4.75 (1 H, app. t, *J* 7.8, 3-H), 4.31 (1 H, dd, *J* 15.0 and 5.9, benzyl 1-H_A), 4.24 (1 H, dd, *J* 15.0 and 5.6, benzyl 1-H_B), 3.03 (1 H, dd, *J* 13.9 and 7.6, 2-H_A) and 2.87 (1 H, dd, *J* 13.9 and 8.0, 2-H_B). δ_C (100 MHz, chloroform-d) 171.5, 143.3, 137.8, 135.0, 128.7 (x2), 128.5 (x2), 127.7 (x2), 127.6, 127.31, 127.27, 126.7, 125.0, 123.2, 122.4, 118.8, 117.9, 112.4, 104.2, 43.6, 43.4 and 39.7. HRMS *m/z* calculated for $C_{24}H_{21}ClN_2NaO$ $(M+Na)^+$: 411.1235; Found: 411.1232. IR (film): 3268, 1641.

3-(5-Chloro-1H-indol-3-yl)-*N*-(2-methoxyethyl)-3-phenylpropanamide 9d

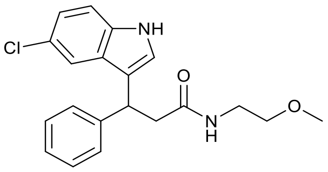

According to general procedure E1, carboxylic acid **25** (50 mg, 0.15 mmol), 2-methoxyethylamine (20 µL, 0.23 mmol), pyridine (50 µL, 0.6 mmol) and T3P (175 µL, 0.3 mmol) in ethyl acetate (0.10 mL) gave the crude product. Purification by column chromatography (silica, Eluent: gradient 0:100 → 20:80 of ethyl acetate:hexane) afforded *amide* **9d** as a colourless oil which solidified on standing (40 mg, 67%); δ_H (400 MHz, chloroform-d) 8.65 (1 H, app. s, NH_{indole}), 7.36 (1 H, d, *J* 2.0, indolyl 4-H), 7.27 (4 H, m, phenyl 2-, 3-, 5- and 6-H), 7.22–7.16 (2 H, m, indolyl 7-H and phenyl 4-H), 7.07 (1 H, dd, *J* 8.6 and 2.0, indolyl 6-H), 7.00 (1 H, d, *J* 2.4, indolyl 2-H), 5.78 (1 H, t, *J* 5.6, NH_{amide}), 4.72 (1 H, app. t, *J* 7.7, 3-H), 3.37–3.25 (2 H, m, methoxyethyl 1-H_2), 3.27–3.11 (2 H, m, methoxyethyl 2-H_2), 3.19 (3 H, s, methoxy), 2.99 (1 H, dd, *J* 14.0 and 7.4, 2-H_A) and 2.83 (1 H, dd, *J* 14.0 and 8.0, 2-H_B). δ_C (125 MHz, chloroform-d) 171.3, 143.5, 135.1, 128.7 (x2), 127.84, 127.79 (x2), 126.8, 125.4, 122.9, 122.7, 119.2, 118.8, 112.2, 71.2, 58.7, 43.8, 39.7 and 39.2. HRMS *m/z* calculated for $C_{20}H_{21}ClN_2NaO_2S$ $(M+Na)^+$: $C_{20}H_{21}ClN_2NaO_2$ 379.1184; Found: 379.1183. IR (film): 3323, 1617.

Methyl 3-(2-bromo-6-chloro-1H-indol-3-yl)-3-(5-chlorothiophen-2-yl)propanoate

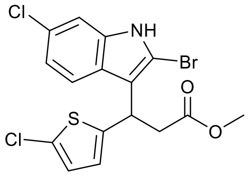

According to general procedure D, ester **21** (870 mg, 2.46 mmol) and pyridinium tribromide (960 mg, 2.70 mmol) in tetrahydrofuran:chloroform (20 mL) gave the crude product. Purification by column chromatography (silica, eluent: gradient 0:100 → 20:80 of ethyl acetate:hexane) afforded *ester* **26** as a yellow oil which solidified on standing (823 mg, 77%); R_f = 0.17 (9:1 hexane:ethyl acetate); δ_H (500 MHz, chloroform-d) 8.16 (1 H, s, NH), 7.36 (1 H, dt, *J* 8.6 and 0.6, indolyl 4-H), 7.28 (1 H, dd, *J* 1.9 and 0.6, indolyl 7-H), 7.05 (1 H, dd, *J* 8.6 and 1.9, indolyl 5-H), 6.69 (1 H, d, *J* 3.8, thiophenyl 4-H), 6.64 (1 H, dd, *J* 3.8 and 1.3, thiophenyl 3-H), 4.94 (1 H, ddd, *J* 8.4, 7.2 and 1.3, 3-H), 3.63 (3 H, s, methyl), 3.30 (1 H, dd, *J* 15.7 and 7.2, 2-H_A) and 3.16 (1 H, dd, *J* 15.7 and 8.4, 2-H_B). δ_C (125 MHz, chloroform-d) 171.6, 145.1, 136.7, 128.9, 128.5, 125.7, 124.6, 123.4, 121.3, 119.8, 115.8, 110.9, 109.4, 52.1, 39.5 and 35.0. HRMS *m/z* calculated for $C_{16}H_{12}BrCl_2NNaO_2S$ $(M+Na)^+$: 453.9047; Found: 453.9031.

***tert*-Butyl 2-bromo-6-chloro-3-[1-(5-chlorothiophen-2-yl)-3-methoxy-3-oxopropyl]-indole-1-carboxylate**

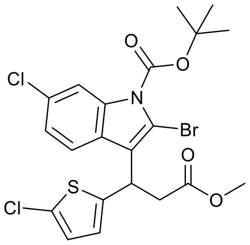

To a solution of **26** (810 mg, 1.87 mmol) and triethylamine (0.80 mL, 5.6 mmol) in dichloromethane (10mL) at 0 °C, di-tert-butyl dicarbonate (817 mg, 3.74 mmol) and DMAP (23 mg, 0.19 mmol) were subsequently added and the reaction mixture was stirred at room temperature for 1.5 h. Saturated aqueous sodium hydrogen carbonate solution (10 mL) was added and the organic layer extracted with dichloromethane (2 x 10 mL). The combined organic layers were dried over magnesium sulfate, filtered and concentrated *in vacuo*. Purification by column chromatography (silica, eluent: gradient 0:100 → 10:90 of ethyl acetate:hexane) afforded *indole* **27** as a yellow oil (997 mg, quantitative yield). R_f = 0.54 (8:2 hexane:ethylacetate); δ_H (500 MHz,

chloroform-d) 8.15 (1 H, d, *J* 1.9, 7-H), 7.29 (1 H, d, *J* 8.5, 4-H), 7.15 (1 H, dd, *J* 8.5 and 1.9, 5-H), 6.70 (1 H, d, *J* 3.8, thiophenyl 4-H), 6.65 (1 H, dd, *J* 3.8 and 1.4, thiophenyl 3-H), 5.10 (1 H, app. td, *J* 7.7 and 1.4, oxopropyl 1-H), 3.65 (3 H, s, methoxy), 3.30 (1 H, dd, *J* 15.8 and 7.4, oxopropyl 2-H_A), 3.12 (1 H, dd, *J* 15.8 and 7.9, oxopropyl 2-H_B) and 1.70 (9 H, s, tert-butyl). δ_C (125 MHz, chloroform-d) 171.3, 148.7, 143.8, 137.3, 130.9, 128.8, 125.8, 125.4, 123.68, 123.66, 122.3, 119.8, 116.0, 110.7, 86.2, 52.2, 38.6, 35.5 and 28.3 (x3). HRMS *m/z* calculated for $C_{21}H_{20}BrCl_2NNaO_4S$ $(M+Na)^+$: 553.9571; Found: 553.9556.

3-(2-Bromo-6-chloro-1H-indol-3-yl)-3-(5-chlorothiophen-2-yl)propanoic acid

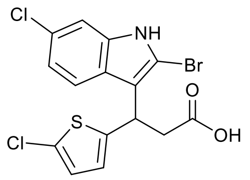

According to general procedure C, ester **26** (100 mg, 0.23 mmol) and sodium hydroxide solution (0.5 mL, 1.4 M) in methanol:tetrahydrofuran (2 mL) afforded *acid* **28** as white solid (96 mg, quantitative yield). δ_H (500 MHz, chloroform-d) 8.13 (1 H, s, NH), 7.36 (1 H, d, *J* 8.6, indolyl 4-H), 7.29 (1 H, d, *J* 1.8, indolyl 7-H), 7.06 (1 H, dd, *J* 8.6 and 1.8, indolyl 5-H), 6.70 (1 H, d, *J* 3.8, thiophenyl 4-H), 6.66 (1 H, dd, *J* 3.8 and 1.0, thiophenyl 3-H), 4.92 (1 H, m, 3-H), 3.33 (1 H, dd, *J* 16.1 and 7.4, 2-H_A) and 3.19 (1 H, dd, *J* 16.1 and 8.0, 2-H_B). δ_C (125 MHz, chloroform-d) 174.8, 144.8, 136.6, 128.9, 128.6, 125.7, 124.6, 123.4, 121.4, 119.8, 115.6, 110.9, 109.4, 39.0 and 34.8. HRMS *m/z* calculated for $C_{15}H_{10}Cl_2NO_2S$ $(M-Br)^+$: 337.9809; Found: 337.9799.

3-(2-Bromo-6-chloro-1H-indol-3-yl)-3-(5-chlorothiophen-2-yl)-*N*-[2-(pyridin-2-yl) ethyl]propanamide 11a

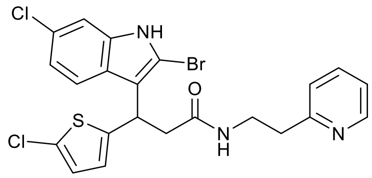

According to general procedure E2, carboxylic acid **28** (402 mg, 0.96 mmol), 2-(2-pyridyl)ethylamine (163 µL, 1.36 mmol), DIPEA (0.50 mL, 2.7 mmol) and HCTU (414 mg, 1.0 mmol) in dimethylformamide (3 mL) at RT gave the crude product. Purification by column chromatography (silica, eluent: gradient 0:100 → 100:0 of ethyl

acetate:hexane) afforded *amide* **11a** as a white solid (484 mg, 91%); δ_H (500 MHz, chloroform-d) 8.39–8.31 (2 H, m, pyridinyl 6-H and NH_{indole}), 7.55 (1 H, td, *J* 7.7 and 1.8, pyridinyl 4-H), 7.33 (1 H, d, *J* 8.5, indolyl 4-H), 7.17 (1 H, d, *J* 1.8, indolyl 7-H), 7.12 (1 H, ddd, *J* 7.7, 4.9 and 1.1, pyridinyl 5-H), 7.00 (1 H, dd, *J* 8.5 and 1.8, indolyl 5-H), 6.97–6.91 (1 H, m, pyridinyl 3-H), 6.66 (1 H, d, *J* 3.8, thiophenyl 4-H), 6.62–6.55 (1 H, m, thiophenyl 3-H), 6.44–6.35 (1 H, m, NH_{amide}), 4.96 (1 H, ddd, *J* 9.1, 6.4 and 1.3, 3-H), 3.64–3.54 (1 H, m, pyridinylethyl 1-H_A), 3.53–3.44 (1 H, m, pyridinylethyl 1-H_B), 3.10 (1 H, dd, *J* 13.9 and 6.4, 2-H_A), 2.97 (1 H, dd, *J* 13.9 and 9.1, 2-H_B), 2.81 (1 H, ddd, *J* 15.1, 7.0 and 4.8, pyridinylethyl 2-H_A) and 2.64 (1 H, ddd, *J* 15.1, 8.0 and 5.0, pyridinylethyl 2-H_B). δ_C (125 MHz, chloroform-d) 170.0, 159.4, 149.1, 145.6, 136.7 (x2), 128.7, 128.2, 125.7, 124.6, 123.43, 123.41, 121.6, 121.2, 119.8, 115.8, 110.9, 109.8, 42.1, 38.5, 36.6 and 35.7. HRMS *m/z* calculated for $C_{22}H_{19}BrCl_2N_3OS$ $(M+H)^+$: 521.9809; Found:: 521.9823.

Methyl 3-(5-chloro-1H-indol-3-yl)-3-(5-chlorothiophen-2-yl)-2,2-dimethyl propanoate

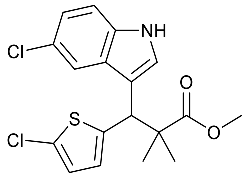

According to general procedure B2, indole **17** (200 mg, 0.46 mmol), [(1-methoxy-2-methyl-1-propenyl)oxy](trimethyl)silane (0.47 mL, 2.3 mmol) and trifluoromethanesulfonic acid (16 µL, 0.18 mmol) in dichloromethane (5 mL) gave the crude product. Purification by column chromatography (silica, eluent: gradient 0:100 → 20:80 of ethyl acetate:hexane) afforded *ester* **29** as an off-white solid (158 mg, 88%); R_f = 0.17 (9:1 hexane:ethyl acetate); δ_H (500 MHz, chloroform-d) 8.16 (1 H, s, NH), 7.56–7.49 (1 H, m, indolyl 4-H), 7.31 (1 H, d, *J* 2.3, indolyl 7-H), 7.28–7.24 (1 H, m, indolyl 2-H), 7.13 (1 H, dd, *J* 8.6 and 2.3, indolyl 6-H), 6.73–6.67 (2 H, m, thiophenyl 3- and 4-H), 4.90 (1 H, s, 3-H), 3.57 (3 H, s, methyl), 1.36 (3 H, s, 2-$methyl_A$) and 1.28 (3 H, s, 2-$methyl_B$). δ_C (125 MHz, chloroform-d) 177.6, 143.7, 133.8, 129.0, 128.4, 126.0, 125.8, 125.4, 123.9, 122.9, 118.7, 115.2, 112.2, 52.1, 47.5, 45.5, 23.8 and 23.7. HRMS *m/z* calculated for $C_{18}H_{17}Cl_2NNaO_2S$ $(M+Na)^+$: 404.0255; Found: 404.0247.

3-(5-Chloro-1H-indol-3-yl)-3-(5-chlorothiophen-2-yl)-2,2-dimethylpropanoic acid

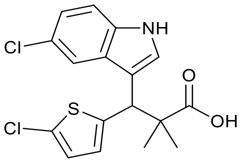

According to general procedure C, ester **29** (150 mg, 0.41 mmol) and sodium hydroxide solution (1.5 mL, 2.5 M) in methanol:tetrahydrofuran (2 mL) at 60 °C afforded *acid* **30** as white solid (166 mg, 95%). δ_H (500 MHz, chloroform-d) 8.15 (1 H, app. s, NH), 7.52 (1 H, d, *J* 2.0, indolyl 4-H), 7.31 (1 H, d, *J* 2.5, indolyl 2-H), 7.26 (1 H, d, *J* 8.6, indolyl 7-H), 7.14 (1 H, dd, *J* 8.6 and 2.0, indolyl 6-H), 6.73 (1 H, d, *J* 3.8, thiophenyl 3-H), 6.69 (1 H, d, *J* 3.8, thiophenyl 4-H), 4.89 (1 H, s, 3-H), 1.38 (3 H, s, 2-Me_A) and 1.30 (3 H, s, 2-Me_B). δ_C (125 MHz, chloroform-d) 182.0, 143.5, 133.8, 129.0, 128.5, 126.3, 125.8, 125.5, 123.9, 122.9, 118.7, 115.1, 112.3, 47.3, 45.3, 23.9 and 23.5. HRMS *m/z* calculated for $C_{17}H_{15}Cl_2NNaO_2S$ $(M+Na)^+$: 390.0098; Found: 390.0091.

3-(5-Chloro-1H-indol-3-yl)-3-(5-chlorothiophen-2-yl)-2,2-dimethyl-*N*-[2-(pyridin-2-yl)ethyl]propanamide 12

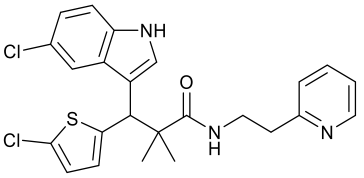

According to general procedure E2, carboxylic acid **30** (140 mg, 0.38 mmol), 2-(2-pyridyl)ethylamine (69 µL, 0.57 mmol), DIPEA (0.20 mL, 1.1 mmol) and HCTU (173 mg, 0.42 mmol) in dimethylformamide (2 mL) at RT gave the crude product. Purification by column chromatography (silica, eluent: gradient 0:100 → 100:0 of ethyl acetate:hexane) afforded 129 mg of impure material. The material was precipitated in diethyl ether/hexane (2 mL, 1:1), filtered and washed with diethyl ether (2 x 2 mL) to afford *amide* **12** as a white solid (116 mg, 64%). R_f = 0.18 (ethyl acetate); δ_H (500 MHz, chloroform-d) 8.48 – 8.41 (1 H, m, pyridinyl 6-H), 8.23 (1 H, app. s, NH_{indole}), 7.60–7.52 (2 H, m, pyridinyl 4-H and indolyl 4-H), 7.31 (1 H, d, *J* 2.5, indolyl 2-H), 7.21 (1 H, d, *J* 8.6, indolyl 7-H), 7.13 (1 H, ddd, *J* 7.7, 5.0 and 1.1, pyridinyl 5-H), 7.10 (1 H, dd, *J* 8.6 and 2.0, indolyl 6-H), 7.03–6.96 (2 H, m, pyridinyl 3-H and NH_{amide}), 6.68 (1 H, dd, *J* 3.8 and 0.8, thiophenyl 3-H), 6.61 (1 H, d, *J* 3.8, thiophenyl 4-H), 4.91 (1 H, s, 3-H), 3.59–3.50 (2 H, m, pyridinylethyl 1-H_2), 2.83–2.69 (2 H, m, pyridinylethyl 2-H_2),

1.31 (3 H, s, 2-methyl$_A$) and 1.21 (3 H, s, 2-methyl$_B$). δ_C (125 MHz, chloroform-d) 176.7, 159.9, 149.1, 144.2, 136.8, 133.8, 129.2, 128.0, 126.1, 125.7, 125.3, 124.2, 123.6, 122.7, 121.7, 119.0, 115.3, 112.1, 47.2, 46.0, 38.9, 36.3, 24.6 and 23.3. HRMS *m/z* calculated for $C_{24}H_{24}Cl_2N_3OS$ (M+H)$^+$: 472.1017; Found: 472.1020.

***syn-* and *anti*-Methyl 3-(5-chloro-1H-indol-3-yl)-3-(5-chlorothiophen-2-yl)-2-methylpropanoate**

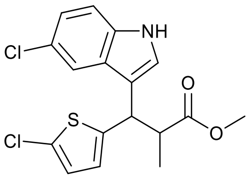

According to general procedure B2, indole **17** (200 mg, 0.46 mmol), [(1-methoxy-1-propenyl)oxy](trimethyl)silane (0.42 mL, 2.3 mmol) and trifluoromethanesulfonic acid (16 µL, 0.18 mmol) in dichloromethane (5 mL) gave the crude product. Purification by column chromatography (silica, eluent: gradient 0:100 → 20:80 of ethyl acetate:hexane) afforded *ester* **31** as a colourless oil (161 mg, 91%); R_f = 0.20 (8:2 hexane:ethyl acetate); approximately a 7:3 mixture of diastereomers. δ_H (500 MHz, chloroform-d) 8.14 (0.7 H, s, NH^{maj}), 8.06 (0.3 H, s, NH^{min}), 7.54 (0.7 H, d, *J* 2.0, indolyl 4-H^{maj}), 7.49 (0.3 H, d, *J* 2.0, indolyl 4-H^{min}), 7.28 (0.7 H, d, *J* 8.6, indolyl 7-H^{maj}), 7.24 (0.3 H, d, *J* 8.6, indolyl 7-H^{min}), 7.22–7.10 (2 H, m, indolyl 2-$^{maj/min}$ and 6-H$^{maj/min}$), 6.77–6.66 (2 H, m, thiophenyl 3-$^{maj/min}$ and 4-H$^{maj/min}$), 4.64–4.58 (1 H, m, 3-H$^{maj/min}$), 3.64 (2.1 H, s, methyl^{maj}), 3.52 (0.9 H, s, methyl^{min}), 3.28 (0.7 H, dq, *J* 10.4 and 7.0, 2-H^{maj}), 3.16 (0.3 H, dq, *J* 10.9 and 7.0, 2-H^{min}), 1.24 (0.9 H, d, *J* 7.0, 2-methyl^{min}) and 1.16 (2.1 H, d, *J* 7.0, 2-methyl^{maj}). δ_C (125 MHz, chloroform-d) 175.9 (maj), 175.7 (min), 146.2 (maj), 145.3 (min), 134.8 (maj), 134.6 (min), 128.3 (min), 127.7 (maj), 125.8 (min), 125.7 (min), 125.6 (x2 maj), 124.9 (min), 123.8 (maj), 123.6 (maj), 123.0 (maj/min), 122.2 (min), 118.83 (x2 maj), 118.76 (min), 117.8 (min), 116.0 (maj/min), 112.5 (maj), 112.3 (min), 52.04 (maj), 52.00 (min), 46.1 (maj), 45.9 (min), 41.6 (maj), 41.5 (min), 16.74 (min) and 16.69 (maj). HRMS *m/z* calculated for $C_{17}H_{15}Cl_2NNaO_2S$ (M+Na)$^+$: 390.0098; Found: 390.0092.

syn- and anti-3-(5-Chloro-1H-indol-3-yl)-3-(5-chlorothiophen-2-yl)-2-methylpropanoic acid

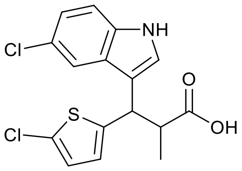

According to general procedure C, ester **31** (154 mg, 0.42 mmol) and sodium hydroxide solution (5 mL, 0.5 M) in methanol (10 mL) at 60 °C afforded *acid* **32** as white solid (161 mg, 98%); approximately a 7:3 mixture of diastereomers. δ_H (500 MHz, chloroform-d) 8.14 (0.7 H, s, NH^{maj}), 8.06 (0.3 H, s, NH^{min}), 7.53 (0.7 H, d, *J* 2.0, indolyl 4-H^{maj}), 7.48 (0.3 H, d, *J* 2.0, indolyl 4-H^{min}), 7.31–7.22 (1 H, m, indolyl 7-$H^{maj/min}$), 7.26–7.10 (2 H, m, indolyl 2-$^{maj/min}$ and 6-$H^{maj/min}$), 6.79–6.76 (0.3 H, m, thiophenyl 3-H^{min}), 6.73 (0.7 H, dd, *J* 3.8 and 0.9, thiophenyl 3-H^{maj}), 6.71 (0.3 H, d, *J* 3.7, thiophenyl 4-H^{min}), 6.66 (0.7 H, d, *J* 3.8, thiophenyl 4-H^{maj}), 4.62 (0.7 H, app. d, *J* 10.1, 3-H^{maj}), 4.58 (0.3 H, d, *J* 10.4, 3-H^{min}), 3.29 (0.7 H, dq, *J* 10.1 and 7.0, 2-H^{maj}), 3.16 (0.3 H, dq, *J* 10.4 and 7.0, 2-H^{min}), 1.27 (0.9 H, d, *J* 7.0, 2-$mehtyl^{min}$) and 1.19 (2.1 H, d, *J* 7.0, 2-$methyl^{maj}$). δ_C (125 MHz, chloroform-d) 180.2 (maj), 180.1 (min), 145.9 (maj), 144.9 (min), 134.74 (maj), 134.65 (min), 128.8 (min), 128.4 (maj), 127.73 (min), 127.68 (maj), 125.8 (min), 125.7 (x2 maj), 125.1 (min), 123.9 (x2 maj), 123.7 (min), 123.10 (min), 123.07 (maj), 122.2 (min), 118.8 (maj), 118.7 (min), 117.6 (min), 115.8 (maj), 112.5 (maj), 112.3 (min), 45.7 (maj), 45.4 (min), 41.3 (min), 41.2 (maj), 16.8 (min) and 16.6 (maj). HRMS *m/z* calculated for $C_{16}H_{13}Cl_2NNaO_2S$ $(M+Na)^+$: 375.9942; Found: 375.9935.

syn- and anti-3-(5-Chloro-1H-indol-3-yl)-3-(5-chlorothiophen-2-yl)-2-methyl-*N*-[2-(pyridin-2-yl)ethyl]propanamide 10

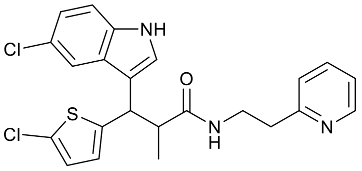

According to general procedure E2, carboxylic acid **32** (157 mg, 0.44 mmol), 2-(2-pyridyl)ethylamine (78 µL, 0.66 mmol), DIPEA (0.24 mL, 1.3 mmol) and HCTU (200 mg, 0.48 mmol) in dimethylformamide (2 mL) at RT gave the crude product. Purification by column chromatography (silica, eluent: gradient 0:100 → 100:0 of ethyl acetate:hexane) afforded two products as colourless materials. Diastereomers *syn*-**10** and *anti*-**10** were isolated in a 7:3 ratio, respectively.

Fraction one was precipitated in diethyl ether/hexane (2 mL, 1:1), filtered and washed with diethyl ether (2 x 2 mL) to afford *amide syn*-**10** as a white solid (97 mg, 69%). R_f = 0.26 (ethyl acetate); δ_H (500 MHz, DMSO-d_6) 11.19 (1 H, d, *J* 2.5, NH_{indole}), 8.50 (1 H, ddd, *J* 4.9, 1.9 and 0.9, pyridinyl 6-H), 8.07 (1 H, t, *J* 5.7, NH_{amide}), 7.68 (1 H, td, *J* 7.6 and 1.9, pyridinyl 4-H), 7.57 (1 H, d, *J* 2.0, indolyl 4-H), 7.41 (1 H, d, *J* 2.5, indolyl 2-H), 7.37 (1 H, d, *J* 8.6, indolyl 7-H), 7.21 (1 H, ddd, *J* 7.6, 4.9 and 1.2, pyridinyl 5-H), 7.17–7.12 (1 H, m, pyridinyl 3-H), 7.07 (1 H, dd, *J* 8.6 and 2.0, indolyl 6-H), 6.82 (1 H, d, *J* 3.8, thiophenyl 4-H), 6.76 (1 H, dd, *J* 3.8 and 0.9, thiophenyl 3-H), 4.49 (1 H, app. d, *J* 11.1, 3-H), 3.47–3.31 (2 H, m, pyridinylethyl 1-H_2), 3.28–3.18 (1 H, m, 2-H), 2.83–2.68 (2 H, m, pyridinylethyl 2-H_2) and 0.84 (3 H, d, *J* 6.8, 2-methyl). δ_C (125 MHz, DMSO-d_6) 174.7, 159.0, 149.1, 147.8, 136.4, 135.1, 126.8, 125.8, 125.5, 125.3, 123.7, 123.2, 123.0, 121.5, 121.1, 118.0, 115.0, 113.3, 44.3, 41.0, 38.2, 37.3 and 17.6. HRMS *m/z* calculated for $C_{23}H_{22}Cl_2N_3OS$ $(M+H)^+$: 458.0861; Found: 458.0848.
Fraction two was precipitated in diethyl ether/hexane (2 mL, 1:1), filtered and washed with diethyl ether (2 x 2 mL) to afford *amide anti*-**10** as a white solid (41 mg, 68%). R_f = 0.13 (ethyl acetate); δ_H (500 MHz, DMSO-d_6) 11.10 (1 H, d, *J* 2.5, NH_{indole}), 8.45 (1 H, ddd, *J* 4.9, 1.9 and 0.9, pyridinyl 6-H), 8.03 (1 H, t, *J* 5.7, NH_{amide}), 7.61 (1 H, td, *J* 7.6 and 1.9, pyridinyl 4-H), 7.51 (1 H, app. d, *J* 2.1, indolyl 4-H), 7.40 (1 H, d, *J* 2.5, indolyl 2-H), 7.32 (1 H, dd, *J* 8.6 and 0.6, indolyl 7-H), 7.18 (1 H, ddd, *J* 7.6, 4.9 and 1.2, pyridinyl 5-H), 7.07–6.97 (3 H, m, pyridinyl 3-H, indolyl 6-H and thiophenyl 3-H), 6.88 (1 H, d, *J* 3.7, thiophenyl 4-H), 4.57 (1 H, d, *J* 11.0, 3-H), 3.25 (2 H, td, *J* 7.2 and 5.7, pyridinylethyl 1-H_2), 3.00 (1 H, dq, *J* 11.0 and 6.8, 2-H), 2.61 (2 H, t, *J* 7.2, pyridinylethyl 2-H_2) and 0.97 (3 H, d, *J* 6.8, 2-methyl). δ_C (125 MHz, DMSO-d_6) 174.1, 158.9, 149.0, 148.1, 136.3, 134.4, 127.6, 126.1 (x2), 125.2, 123.13, 123.10, 123.0, 121.4, 121.1, 117.8, 116.9, 112.9, 45.1, 40.5, 38.2, 37.3 and 17.5. HRMS *m/z* calculated for $C_{23}H_{22}Cl_2N_3OS$ $(M+H)^+$: 458.0861; Found: 458.0851.

From a racemic mixture of *anti*-**10** (27 mg), enantiomers (+)-*anti*-**10** (6.5 mg, >99.0 %ee) and (–)-*anti*-**10** (1.0 mg, >99.0 %ee) were isolated using the following supercritical fluid chromatography conditions:
Mobile phase: A= 70% $scCO_2$, B= 30% MeOH + 0.1% NH_3
Flow rate: 90 ml/min

The collected fractions were analysed using the following conditions:

Column: Phenomenex C1, 3 x 150 mm, 3 micron
Mobile phase: A = 60% $scCO_2$, B = 20% MeOH 0.1% NH_3
Gradient 0-1 min 5%B, 1-5 min 5-50%B, 5-10 min 50%B
Flow rate: 2.0 ml/min

Right and left optical rotations have been arbitrarily assigned.

Methyl 3-(2-bromo-5-chloro-1H-indol-3-yl)-3-(5-chlorothiophen-2-yl)propanoate

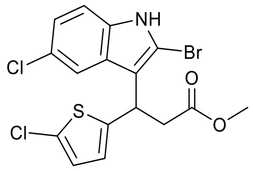

According to general procedure D, ester **18** (850 mg, 2.40 mmol) and pyridinium tribromide (945 mg, 2.64 mmol) in tetrahydrofuran:chloroform (20 mL) gave the crude product. Purification by column chromatography (silica, eluent: gradient 0:100 → 20:80 of ethyl acetate:hexane) afforded *ester* **33** as a yellow oil which solidified on standing (729 mg, 70%); δ_H (500 MHz, chloroform-d) 8.15 (1 H, s, NH), 7.42 (1 H, d, *J* 1.9, indolyl 4-H), 7.20 (1 H, d, *J* 8.7, indolyl 7-H), 7.13 (1 H, dd, *J* 8.7 and 1.9, indolyl 6-H), 6.71 (1 H, d, *J* 3.8, thiophenyl 4-H), 6.65 (1 H, dd, *J* 3.8 and 1.3, thiophenyl 3-H), 4.97–4.90 (1 H, m, 3-H), 3.63 (3 H, s, methyl), 3.30 (1 H, dd, *J* 15.8 and 7.2, 2-H_A) and 3.16 (1 H, dd, *J* 15.8 and 8.2, 2-H_B). δ_C (125 MHz, chloroform-d) 171.6, 145.0, 134.7, 128.5, 127.0, 126.4, 125.8, 123.4, 123.2, 118.4, 115.5, 111.9, 110.3, 52.1, 39.3 and 35.0. HRMS *m/z* calculated for $C_{16}H_{12}BrCl_2NNaO_2S$ $(M+Na)^+$: 453.9047; Found: 453.9033.

3-(2-Bromo-5-chloro-1H-indol-3-yl)-3-(5-chlorothiophen-2-yl)propanoic acid

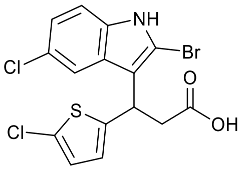

According to general procedure C, ester **33** (740 mg, 1.71 mmol) and sodium hydroxide solution (10 mL, 1.4 M) in methanol:tetrahydrofuran (13 mL) afforded *acid* **34** as white solid (717 mg, quantitative yield). δ_H (500 MHz, methanol-d_4) 7.39 (1 H, dd, *J* 2.0 and 0.6, indolyl 4-H), 7.26 (1 H, app. d, *J* 8.6, indolyl 7-H), 7.07 (1 H, dd, *J* 8.7 and 2.0, indolyl 6-H), 6.79–6.71 (2 H, m, thiophenyl 3- and 4-H), 4.92 (1 H, ddd, *J* 8.2, 7.4 and 1.2, 3-H), 3.36–3.27 (1 H, m, 2-H_A) and 3.11 (1 H, dd, *J* 15.6 and 8.2, 2-H_B). δ_C (125 MHz, methanol-d_4) 174.7, 147.6, 136.5, 128.9, 128.1, 126.8, 126.5,

124.4, 123.1, 118.8, 115.6, 113.2, 111.9, 40.2 and 36.2. HRMS *m/z* calculated for $C_{15}H_{10}Cl_2NO_2S$ (M-Br)$^+$: 337.9809; Found: 337.9799.

3-(2-Bromo-5-chloro-1H-indol-3-yl)-3-(5-chlorothiophen-2-yl)-*N*-[2-(pyridin-2-yl) ethyl]propanamide 11d

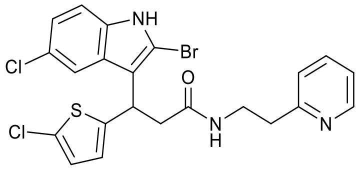

According to general procedure E2, carboxylic acid **34** (717 mg, 1.71 mmol), 2-(2-pyridyl)ethylamine (313 µL, 2.57 mmol), DIPEA (0.90 mL, 5.1 mmol) and HCTU (793 mg, 1.89 mmol) in dimethylformamide (5 mL) at RT gave the crude product. Purification by column chromatography (silica, eluent: gradient 0:100 → 100:0 of ethyl acetate:hexane) afforded a colourless material. The material was precipitated in diethyl ether/hexane (2 mL, 1:1), filtered and washed with diethyl ether (2 x 2 mL) to afford *amide* **11d** as a white solid (713 mg, 78%). δ_H (500 MHz, chloroform-d) 8.46 (1 H, s, NH_{indole}), 8.42–8.37 (1 H, m, pyridinyl 6-H), 7.54 (1 H, td, *J* 7.7 and 1.9, pyridinyl 4-H), 7.40 (1 H, d, *J* 2.0, indolyl 4-H), 7.16–7.07 (2 H, m, pyridinyl 5-H and indolyl 7-H), 7.07 (1 H, dd, *J* 8.6 and 2.0, indolyl 6-H), 6.99–6.94 (1 H, m, pyridinyl 3-H), 6.67 (1 H, d, *J* 3.8, thiophenyl 4-H), 6.60 (1 H, dd, *J* 3.8 and 1.3, thiophenyl 3-H), 6.42 (1 H, t, *J* 5.8, NH_{amide}), 4.96 (1 H, ddd, *J* 8.9, 6.6 and 1.3, 3-H), 3.63–3.54 (1 H, m, pyridinylethyl 1-H_A), 3.54–3.45 (1 H, m, pyridinylethyl 1-H_B), 3.11 (1 H, dd, *J* 14.1 and 6.6, 2-H_A), 2.97 (1 H, dd, *J* 14.1 and 8.9, 2-H_B), 2.86–2.81 (1 H, m, pyridinylethyl 2-H_A) and 2.71–2.63 (1 H, m, pyridinylethyl 2-H_B). δ_C (125 MHz, chloroform-d) 170.0, 159.5, 149.2, 145.5, 136.7, 134.8, 128.2, 127.0, 126.2, 125.8, 123.4 (x2), 123.0, 121.7, 118.3, 115.4, 112.0, 110.7, 42.0, 38.6, 36.7 and 35.6. HRMS *m/z* calculated for $C_{22}H_{19}BrCl_2N_3OS$ (M+H)$^+$: 523.9789; Found: 523.9800.

N-Benzyl-5-chloro-3-[(5-chlorothiophene-2-yl)(4-methylbenzenesulfonyl) methyl]-1H-indole-2-carboxamide

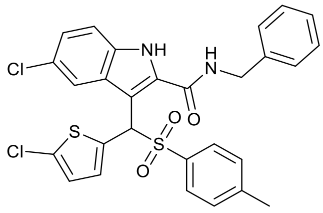

According to general procedure A2, *N*-benzyl-5-chloro-1H-indole-2-carboxamide (190 mg, 0.67 mmol), 5-chloro-2-thiophenecarboxaldehyde (65 µL, 0.61 mmol), *p*-toluenesulfinic acid (334 mg, 2.1 mmol) and *p*-toluenesulfonic acid monohydrate (120 mg, 0.62 mmol) in tetrahydrofuran (7 mL) gave the crude product. The product was precipitated in cold diethyl ether (10 mL), filtered, washed with diethyl ether (2 x 2 mL) and dried *in vacuo* to afford *indole* **35** as a light pink solid (98 mg, 28%). δ_H (500 MHz, DMSO-d_6) 11.92 (1 H, s, NH_{indole}), 8.72 (1 H, app. t, *J* 5.7, $NH_{carboxamide}$), 7.91 (1 H, d, *J* 2.0, indolyl 4-H), 7.60 (1 H, d, *J* 1.1, CHS), 7.50 (1 H, d, *J* 8.8, indolyl 7-H), 7.43–7.34 (4 H, m, benzenesulfonyl 2- and 6-H and benzyl 4- and 6-H), 7.33–7.27 (4 H, m, indolyl 6-H and benzyl 3-, 5- and 7-H), 7.24 (2 H, app. d, *J* 8.1, benzenesulfonyl 3- and 5-H), 7.04 (1 H, d, *J* 3.9, thiophenyl 4-H), 6.87 (1 H, dd, *J* 3.9 and 1.1, thiophenyl 3-H), 4.45 (1 H, dd, *J* 14.9 and 5.9, benzyl 1-H_A), 4.38 (1 H, dd, *J* 14.9 and 5.7, benzyl 1-H_B) and 2.32 (3 H, s, benzenesulfonyl 4-methyl). δ_C (125 MHz, DMSO-d_6) 160.6, 144.8, 138.5, 134.2, 133.9, 133.1, 131.1, 129.5 (x2), 129.3, 129.2, 128.4 (x2), 128.1 (x2), 127.7 (x2), 127.1, 126.6, 125.8, 124.9, 124.5, 121.9, 114.3, 109.4, 62.8, 42.5 and 21.1. HRMS *m/z* calculated for $C_{21}H_{15}Cl_2N_2OS$ $(M\text{-}SO_2Tol)^+$: 413.0282; Found: 413.0276.

Methyl 3-[2-(benzylcarbamoyl)-5-chloro-1H-indol-3-yl]-3-(5-chlorothiophen-2-yl) propanoate

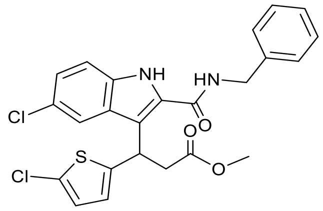

According to general procedure B2, indole **35** (95 mg, 0.17 mmol), tert-butyl((1-methoxyvinyl)oxy)dimethylsilane (182 µL, 0.83 mmol) and trifluoromethanesulfonic acid (6.0 µL, 0.07 mmol) in tetrahydrofuran (3 mL) gave the crude product. Purification by column chromatography (silica, eluent: gradient 0:100 → 50:50 of ethyl acetate:hexane) afforded *ester* **36** as a white solid (18 mg, 22%). R_f = 0.5 (6:4

hexane:ethyl acetate); δ_H (400 MHz, chloroform-d) 9.34 (1 H, s, NH_{indole}), 8.74–8.57 (1 H, m, $NH_{carbamoyl}$), 7.41–7.32 (5 H, m, indolyl 4-H and benzyl 3-, 4-, 6- and 7-H), 7.31–7.27 (2 H, m, indolyl 7-H and benzyl 5-H), 7.17 (1 H, dd, *J* 8.8 and 1.9, indolyl 6-H), 6.65 (1 H, d, *J* 3.9, thiophenyl 4-H), 6.45 (1 H, dd, *J* 3.9 and 1.5, thiophenyl 3-H), 5.23–5.14 (1 H, m, 3-H), 4.74 (1 H, dd, *J* 14.9 and 5.7, benzyl 1-H_A), 4.67 (1 H, dd, *J* 14.9 and 5.7, benzyl 1-H_B), 3.57 (3 H, s, methyl), 3.44 (1 H, dd, *J* 16.9 and 11.4, 2-H_A) and 3.28 (1 H, dd, *J* 17.0 and 3.8, 2-H_B). δ_C (100 MHz, chloroform-d) 173.7, 160.3, 147.1, 135.4, 132.7, 130.9, 129.8, 128.8 (x2), 127.9 (x2), 127.6 (x2), 125.9 (x2), 125.0 (x2), 123.7, 120.5, 113.5, 52.5, 44.2, 38.9 and 33.9. HRMS *m/z* calculated for $C_{24}H_{21}Cl_2N_2O_3S$ $(M+H)^+$: 487.0650; Found: 486.9292.

3-[2-(Benzylcarbamoyl)-5-chloro-1H-indol-3-yl]-3-(5-chlorothiophen-2-yl) propanoic acid

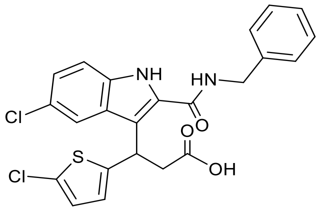

According to general procedure C, ester **36** (18 mg, 0.037 mmol) and sodium hydroxide solution (0.6 mL, 0.3 M) in methanol:tetrahydrofuran (1.4 mL) afforded *acid* **37** as white solid (18 mg, quantitative yield). δ_H (500 MHz, methanol-d_4) 7.44 (1 H, d, *J* 2.0, indolyl 4-H), 7.42–7.36 (3 H, m, indolyl 7-H and benzyl 3- and 7-H), 7.35–7.30 (2 H, m, benzyl 4- and 6-H), 7.28–7.23 (1 H, m, benzyl 5-H), 7.16 (1 H, dd, *J* 8.7 and 2.0, indolyl 6-H), 6.75 (1 H, d, *J* 3.9, thiophenyl 4-H), 6.74 (1 H, dd, *J* 3.9 and 1.3, thiophenyl 3-H), 5.40 (1 H, ddd, *J* 9.5, 6.0 and 1.3, 3-H), 4.68–4.58 (2 H, m, benzyl 1-H_2), 3.39 (1 H, dd, *J* 16.4 and 6.0, 2-H_A) and 3.38–3.30 (1 H, m, 2-H_B). δ_C (125 MHz, methanol-d_4) 175.9, 164.3, 148.0, 139.7, 136.1, 131.4, 129.6 (x2), 129.1, 128.7 (x2), 128.3, 127.9, 126.8, 126.5, 125.3, 124.4, 121.1, 118.4, 114.7, 44.5, 39.8 and 35.0. HRMS *m/z* calculated for $C_{23}H_{18}Cl_2N_2NaO_3S$ $(M+Na)^+$: 495.0313; Found: 495.0305.

_N_-Benzyl-5-chloro-3-[1-(5-chlorothiophene-2-yl)-2-{[2-(pyridin-2-yl)ethyl] carbamoyl}ethyl]-1H-indole-2-carboxamide 11h

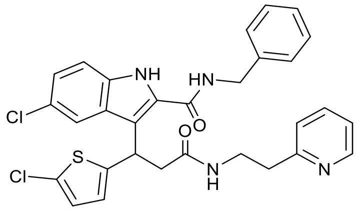

According to general procedure E2, carboxylic acid **37** (18 mg, 0.037 mmol), 2-(2-pyridyl)ethylamine (16 µL, 0.13 mmol), DIPEA (42 µL, 0.24 mmol) and HCTU (25 mg, 0.063 mmol) in dimethylformamide (0.5 mL) at RT gave the crude product. Purification by column chromatography (silica, eluent: gradient 0:100 → 1:99 of methanol:dichloromethane) afforded *indole* **11h** as a white solid (12.8 mg, 55%). δ_H (500 MHz, DMSO-d$_6$) 11.70 (1 H, s, NH$_{indole}$), 9.38 (1 H, t, *J* 5.9, NH$_{carboxamide}$), 8.41 (1 H, ddd, *J* 4.9, 1.9 and 0.9, pyridinyl 6-H), 8.23 (1 H, t, *J* 5.7, NH$_{carbamoyl}$), 7.49 (1 H, td, *J* 7.6 and 1.9, pyridinyl 4-H), 7.42 (1 H, d, *J* 8.7, indolyl 7-H), 7.43–7.37 (3 H, m, indolyl 4-H and benzyl 3- and 7-H), 7.33 (2 H, app. dd, *J* 8.4 and 6.8, benzyl 4- and 6-H), 7.28–7.23 (1 H, m, benzyl 5-H), 7.19 (1 H, dd, *J* 8.7 and 2.0, indolyl 6-H), 7.15 (1 H, ddd, *J* 7.6, 4.9 and 1.2, pyridinyl 5-H), 6.90 (1 H, d, *J* 3.8, thiophenyl 4-H), 6.84 (1 H, app. d, *J* 7.8, pyridinyl 3-H), 6.77 (1 H, dd, *J* 3.8 and 1.4, thiophenyl 3-H), 5.43 (1 H, ddd, *J* 9.4, 6.5 and 1.4, carbamoylethyl 1-H), 4.57 (2 H, d, *J* 5.9, benzyl 1-H$_2$), 3.41–3.22 (3 H, m, carbamoylethyl 2-H$_A$, pyridinylethyl 1-H$_2$), 3.02 (1 H, dd, *J* 14.9 and 9.4, carbamoylethyl 2-H$_B$) and 2.67 (2 H, td, *J* 7.0 and 3.6, pyridinylethyl 2-H$_2$). δ_C (125 MHz, DMSO-d$_6$) 170.8, 161.5, 158.7, 148.9, 147.4, 139.1, 136.3, 134.3, 130.4, 128.3 (x2), 127.3 (x2), 126.9, 126.3, 126.2, 125.9, 123.8, 123.4, 123.2, 122.9, 121.4, 119.8, 116.4, 114.1, 42.6, 39.2, 38.5, 37.0 and 33.2. HRMS *m/z* calculated for $C_{30}H_{27}Cl_2N_4O_2S$ $(M+H)^+$: 577.1232; Found: 577.1223.

5-Chloro-3-[(5-chlorothiophene-2-yl)(4-methylbenzenesulfonyl)methyl]-*N*-(2-methylpropyl)-1H-indole-2-carboxamide

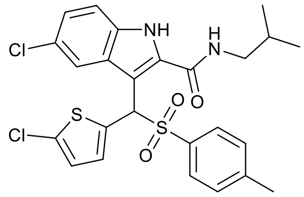

According to general procedure A2, 5-chloro-1H-indole-2-carboxamide (150 mg, 0.60 mmol), 5-chloro-2-thiophenecarboxaldehyde (64 µL, 0.60 mmol), *p*-toluenesulfinic acid (375 mg, 2.4 mmol) and *p*-toluenesulfonic acid monohydrate (114 mg, 0.60 mmol) in tetrahydrofuran (5 mL) gave the crude product. The product was precipitated in cold diethyl ether (10 mL), filtered, washed with diethyl ether (2 x 2 mL) and dried *in vacuo* to afford *indole* **38** as a light pink solid (152 mg, 46%). R_f = 0.38 (1:1 hexane:ethyl acetate); δ_H (500 MHz, DMSO-d$_6$) 11.87 (1 H, s, NH$_{indole}$), 8.20 (1 H, t, *J* 5.7, NH$_{carboxamide}$), 7.90 (1 H, d, *J* 2.0, indolyl 4-H), 7.63 (1 H, d, *J* 1.1, CHS), 7.54–7.48 (1 H, m, indolyl 7-H), 7.40–7.33 (2 H, m, benzenesulfonyl 2- and 6-H), 7.30 (1 H, dd, *J* 8.8 and 2.0, indolyl 6-H), 7.29–7.22 (2 H, m, benzenesulfonyl 3- and 5-H), 7.03 (1 H, d, *J* 3.9, thiophenyl 4-H), 6.87 (1 H, dd, *J* 3.9 and 1.1, thiophenyl 3-H), 3.10–3.03 (1 H, m, methylpropyl 1-H$_A$), 2.96 (1 H, ddd, *J* 12.8, 6.7 and 5.7, methylpropyl 1-H$_B$), 2.32 (3 H, s, benzenesulfonyl 4-methyl), 1.75 (1 H, hept, *J* 6.7, methylpropyl 2-H), 0.87 (3 H, d, *J* 6.7, methylpropyl 3-H$_3$) and 0.86 (3 H, d, *J* 6.7, propyl 2-methyl). δ_C (125 MHz, DMSO-d$_6$) 160.6, 144.8, 134.2, 133.8, 133.1, 131.3, 129.5 (x2), 129.3, 129.2, 128.2 (x2), 126.6, 125.9, 124.9, 124.4, 121.9, 114.3, 109.2, 62.7, 46.3, 27.9, 21.1 and 20.2 (x2). HRMS *m/z* calculated for $C_{18}H_{16}Cl_2N_2NaOS$ (M-SO$_2$Tol+Na)$^+$: 401.0258; Found: 401.0250.

Methyl 3-{5-chloro-2-[(2-methylpropyl)carbamoyl]-1H-indol-3-yl}-3-(5-chlorothiophen-2-yl)propanoate

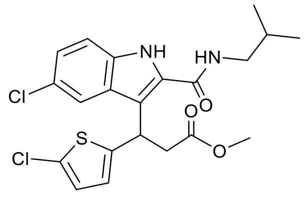

According to general procedure B2, **38** (148 mg, 0.28 mmol), tert-butyl((1-methoxyvinyl)oxy)dimethylsilane (610 µL, 2.8 mmol) and trifluoromethanesulfonic acid (10 µL, 0.11 mmol) in tetrahydrofuran (10 mL) gave the crude product. Purification by column chromatography (silica, eluent: gradient 0:100 → 50:50 of ethyl

acetate:hexane) afforded *ester* **39** as a yellow oil (50 mg, 40%). R_f = 0.29 (8:2 hexane:ethyl acetate); δ_H (500 MHz, chloroform-d) 9.83–9.73 (1 H, m, NH_{indole}), 8.41–8.34 (1 H, m, $NH_{carbamoyl}$), 7.36 (1 H, d, *J* 2.0, indolyl 4-H), 7.33 (1 H, d, *J* 8.7, indolyl 7-H), 7.17 (1 H, dd, *J* 8.7 and 2.0, indolyl 6-H), 6.67 (1 H, d, *J* 3.8, thiophenyl 4-H), 6.48 (1 H, dd, *J* 3.8 and 1.5, thiophenyl 3-H), 5.19 (1 H, ddd, *J* 11.5, 3.7 and 1.5, 3-H), 3.62 (3 H, s, methyl), 3.50 (1 H, dd, *J* 17.0 and 11.5, 2-H_A), 3.44–3.37 (1 H, m, methylpropyl 1-H_A), 3.37–3.28 (2 H, m, methylpropyl 1-H_B and 2-H_B), 1.99 (1 H, hept, *J* 6.7, methylpropyl 2-H), 1.02 (3 H, d, *J* 6.7, methylpropyl 3-H_3) and 1.02 (3 H, d, *J* 6.7, propyl 2-methyl). δ_C (125 MHz, chloroform-d) 173.8, 162.7, 145.2, 134.3, 131.4, 129.0, 126.7, 125.9, 125.8, 124.7, 123.7, 120.5, 113.9, 113.6, 52.6, 47.8, 39.0, 34.0, 28.6, 20.52 and 20.50. HRMS *m/z* calculated for $C_{21}H_{23}Cl_2N_2O_3S$ $(M+H)^+$: 453.0807; Found: 453.0796.

3-{5-Chloro-2-[(2-methylpropyl)carbamoyl]-1H-indol-3-yl}-3-(5-chlorothiophen-2-yl)propanoic acid

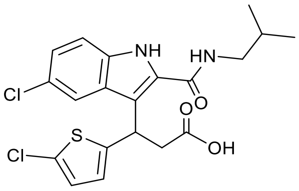

According to general procedure C, ester **39** (50 mg, 0.11 mmol) and sodium hydroxide solution (1.5 mL, 0.4 M) in methanol:tetrahydrofuran (4 mL) afforded *acid* **40** as a white solid (54 mg, quantitative yield). δ_H (500 MHz, methanol-d_4) 7.42 (1 H, d, *J* 2.0, indolyl 4-H), 7.38 (1 H, d, *J* 8.8, indolyl 7-H), 7.16 (1 H, dd, *J* 8.8 and 2.0, indolyl 6-H), 6.77 (2 H, app. s, thiophenyl 3- and 4-H), 5.34 (1 H, dd, *J* 9.9 and 5.5, 3-H), 3.49–3.32 (3 H, m, 2-H_2 and methylpropyl 1-H_A), 3.24 (1 H, dd, *J* 13.2 and 6.6, methylpropyl 1-H_B), 1.95 (1 H, m, methylpropyl 2-H) and 1.01 (6 H, m, methylpropyl 3-H_3 and propyl 2-methyl). δ_C (125 MHz, methanol-d_4) 176.1, 164.5, 148.1, 136.1, 131.9, 129.2, 127.8, 126.8, 126.5, 125.2, 124.3, 121.1, 117.6, 114.7, 48.3, 39.7, 35.1, 30.0 and 20.7 (x2). HRMS *m/z* calculated for $C_{20}H_{21}Cl_2N_2O_3S$ $(M+H)^+$: 439.0650; Found: 439.0643.

5-Chloro-3-[1-(5-chlorothiophene-2-yl)-2-{[2-pyridin-2-yl)ethyl]carbamoyl}ethyl] -*N*-(2-methylpropyl)-1H-indole-2-carboxamide 11i

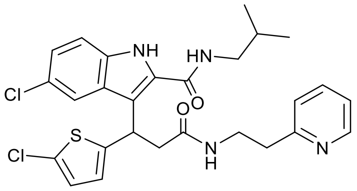

According to general procedure E2, carboxylic acid **40** (50 mg, 0.11 mmol), 2-(2-pyridyl)ethylamine (26 µL, 0.22 mmol), DIPEA (60 µL, 0.33 mmol) and HCTU (60 mg, 0.14 mmol) in dimethylformamide (1 mL) at RT gave the crude product. Purification by column chromatography (silica, eluent: gradient 0:100 → 3:97 of methanol:dichloromethane) afforded 30 mg of a yellow impure material. The material was precipitated in diethyl ether/hexane (2 mL, 1:1), filtered and washed with diethyl ether (2 x 2 mL) to afford *indole* **11i** as a white solid (20 mg, 32%). δ_H (500 MHz, DMSO-d_6) 11.66 (1 H, s, NH_{indole}), 8.98 (1 H, t, *J* 5.7, $NH_{carboxamide}$), 8.39 (1 H, ddd, *J* 4.9, 1.9 and 0.9, pyridinyl 6-H), 8.25 (1 H, t, *J* 5.7, $NH_{carbamoyl}$), 7.45 (1 H, td, *J* 7.6 and 1.9, pyridinyl 4-H), 7.42 (1 H, d, *J* 8.7, indolyl 7-H), 7.36 (1 H, d, *J* 2.0, indolyl 4-H), 7.17 (1 H, dd, *J* 8.7 and 2.0, indolyl 6-H), 7.13 (1 H, ddd, *J* 7.6, 4.9 and 1.2, pyridinyl 5-H), 6.90 (1 H, d, *J* 3.8, thiophenyl 4-H), 6.82–6.75 (2 H, m, pyridinyl 3-H and thiophenyl 3-H), 5.36–5.27 (1 H, m, carbamoylethyl 1-H), 3.39–3.20 (4 H, m, carbamoylethyl 2-H_A, pyridinylethyl 1-H_2 and methylpropyl 1-H_A), 3.19–3.03 (2 H, m, carbamoylethyl 2-H_B and methylpropyl 1-H_B), 2.73–2.62 (2 H, m, pyridinylethyl 2-H_2), 1.89 (1 H, hept, *J* 6.7, methylpropyl 2-H), 0.96 (3 H, d, *J* 6.7, methylpropyl 3-H_3) and 0.95 (3 H, d, *J* 6.7, propyl 2-methyl). δ_C (125 MHz, DMSO-d_6) 170.4, 160.8, 158.2, 148.4, 146.9, 135.6, 133.8, 130.6, 125.8, 125.7, 125.5, 123.1, 122.7 (x2), 122.31, 120.8, 119.2, 114.7, 113.5, 46.0, 38.5, 38.0, 36.5, 32.7, 27.6 and 19.7 (x2). HRMS *m/z* calculated for $C_{27}H_{29}Cl_2N_4O_2S$ $(M+H)^+$: 543.1389; Found: 543.1403.

***tert*-Butyl N-{2-[(5-chloro-1H-indol-2-yl)formamido]ethyl}carbamate**

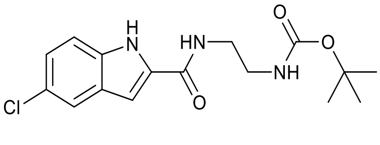

According to general procedure E2, 5-chloroindole-2-carboxylic acid (500 mg, 2.56 mmol), tert-butyl N-(2-aminoethyl)carbamate (1.2 g, 7.7 mmol), DIPEA (1.4 mL, 7.7 mmol) and HCTU (1.1 g, 2.6 mmol) in dimethylformamide (4 mL) gave the crude product. Purification by column chromatography (silica, eluent: gradient 0:100 →

100:0 of ethyl acetate:hexane) afforded *carbamate* **41** as a white solid (178 mg, 20%). δ_H (500 MHz, methanol-d$_4$) 7.58 (1 H, dd, *J* 2.1 and 0.7, indolyl 4-H), 7.41 (1 H, dd, *J* 8.8 and 0.7, indolyl 7-H), 7.17 (1 H, dd, *J* 8.8 and 2.1, indolyl 6-H), 7.00 (1 H, s, indolyl 3-H), 3.46 (2 H, t, *J* 6.2, formamidoethyl 1-H$_2$), 3.28 (2 H, t, *J* 6.2, formamidoethyl 2-H$_2$) and 1.41 (9 H, s, tert-butyl). δ_C (125 MHz, methanol-d$_4$) 164.0, 158.8, 136.6, 133.8, 129.9, 126.7, 125.2, 121.8, 114.4, 103.7, 80.2, 40.99, 40.95 and 28.7 (x3). HRMS *m/z* calculated for $C_{16}H_{20}ClN_3NaO_3$ $(M+H)^+$: 360.1091; Found: 360.1080.

5-Chloro-3-[(5-chlorothiophene-2-yl)(4-methylbenzenesulfonyl)methyl]-1H-indole-2-carboxylic acid

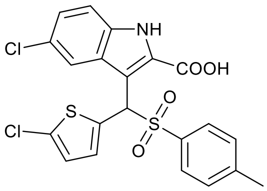

According to general procedure A2, 5-chloroindole-2-carboxylic acid (300 mg, 1.53 mmol), 5-chloro-2-thiophenecarboxaldehyde (163 µL, 1.53 mmol), *p*-toluenesulfinic acid (956 mg, 6.12 mmol) and *p*-toluenesulfonic acid monohydrate (291 mg, 1.53 mmol) in tetrahydrofuran (10 mL) gave the crude product. Purification by column chromatography (silica, eluent: gradient 0:100 → 2:98 of methanol:dichloromethane) afforded *indole* **42** as a salmon solid (335 mg, 46%). δ_H (500 MHz, DMSO-d$_6$) 13.90 (1 H, br s, CO_2H), 12.22 (1 H, s, NH), 7.88 (1 H, d, *J* 2.0, 4-H), 7.47 (1 H, d, *J* 8.8, 7-H), 7.40–7.34 (2 H, m, benzenesulfonyl 2- and 6-H), 7.36–7.28 (2 H, m, 5-H, CHS), 7.30–7.25 (2 H, m, benzenesulfonyl 3- and 5-H), 7.05 (1 H, d, *J* 3.9, thiophenyl 4-H), 6.90 (1 H, dd, *J* 3.9 and 1.1, thiophenyl 3-H) and 2.33 (3 H, s, benzenesulfonyl 4-methyl). δ_C (125 MHz, DMSO-d$_6$) 162.19, 144.98, 134.41, 133.98, 132.49, 129.52 (x2), 129.48, 128.21 (x2), 126.68 (x2), 125.68 (x2), 125.13, 124.98, 122.14, 114.69, 110.54, 63.25 and 21.07. HRMS *m/z* calculated for $C_{14}H_8Cl_2NO_2S$ $(M-SO_2Tol)^+$: 323.9653; Found: 323.9645.

5-Chloro-3-[1-(5-chlorothiophene-2-yl)-3-methoxy-3-oxopropyl]-1H-indole-2-carboxylic acid

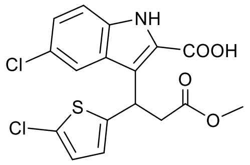

According to general procedure B2, indole **42** (200 mg, 0.42 mmol), tert-butyl((1-methoxyvinyl)oxy)dimethylsilane (454 µL, 2.08 mmol) and trifluoromethanesulfonic acid (15 µL, 0.17 mmol) in 1,4-dioxane (6 mL) gave the crude product. Purification by column chromatography (silica, eluent: gradient 0:100 → 4:96 of methanol:dichloromethane) afforded *indole* **43** as a white solid (130 mg, 74%); δ_H (500 MHz, DMSO-d_6) 13.49 (1 H, br s, CO_2H), 11.87 (1 H, s, NH), 7.64 (1 H, d, *J* 2.0, 4-H), 7.42 (1 H, d, *J* 8.8, 7-H), 7.23 (1 H, dd, *J* 8.8 and 2.0, 6-H), 6.93 (1 H, dd, *J* 3.9 and 1.1, thiophenyl 3-H), 6.92 (1 H, d, *J* 3.9, thiophenyl 4-H), 5.76 (1 H, td, *J* 7.8 and 1.1, oxopropyl 1-H), 3.50 (3 H, s, methoxy), 3.51–3.44 (1 H, m, oxopropyl 2-H_A) and 3.22 (1 H, dd, *J* 16.0 and 7.8, oxopropyl 2-H_B). δ_C (125 MHz, DMSO-d_6) 171.4, 162.8, 146.7, 134.6, 126.3, 126.2, 126.0, 125.6, 124.7, 124.4, 123.5, 121.3, 120.1, 114.6, 51.5, 38.4 and 32.9. HRMS *m/z* calculated for $C_{17}H_{13}Cl_2NNaO_4S$ $(M+Na)^+$: 419.9840; Found: 419.9832.

3-[2-carboxy-1-(5-chlorothiophen-2-yl)ethyl]-5-chloro-1H-indole-2-carboxylic acid

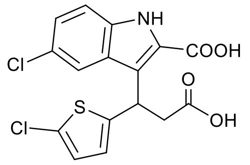

According to general procedure C, indole **43** (40 mg, 0.10 mmol) and sodium hydroxide solution (1.3 mL, 0.4 M) in methanol:tetrahydrofuran (2 mL) afforded *indole* **44** as yellow material (40 mg, quantitative yield). δ_H (500 MHz, DMSO-d_6) 13.45 (1 H, br s, CO_2H_A), 12.23 (1 H, br s, CO_2H_B), 11.86 (1 H, s, NH), 7.64 (1 H, d, *J* 2.0, 4-H), 7.42 (1 H, d, *J* 8.8, 7-H), 7.23 (1 H, dd, *J* 8.8 and 2.0, 6-H), 6.91 (2 H, app. s, thiophenyl 3- and 4-H), 5.73 (1 H, app. t, *J* 7.8, ethyl 1-H), 3.38 (1 H, dd, *J* 16.0 and 8.1, ethyl 2-H_A) and 3.10 (1 H, dd, *J* 16.0 and 7.5, ethyl 2-H_B). δ_C (125 MHz, DMSO-d_6) 172.3, 162.8, 147.1, 134.6, 126.22, 126.21, 125.8, 125.4, 124.6, 124.2, 123.3, 121.6, 120.1, 114.5, 38.8 and 32.9. HRMS *m/z* calculated for $C_{16}H_{11}Cl_2NNaO_4S$ $(M+na)^+$: 405.9676; Found: 405.9684.

5-Chloro-3-[1-(5-chlorothiophen-2-yl)-2-{[2-(pyridin-2-yl)ethyl]carbamoyl}ethyl] -1H-indole-2-carboxylic acid 11b

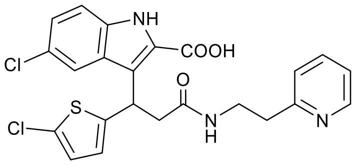

According to general procedure E2, indole **44** (185 mg, 0.48 mmol), 2-(2-pyridyl)ethylamine (65 µL, 0.53 mmol), DIPEA (0.25 mL, 1.4 mmol) and HCTU (220 mg, 0.53 mmol) in dimethylformamide (4 mL) at 0 °C gave the crude product. Purification by column chromatography (silica, eluent: gradient 0:100 → 10:90 of methanol:dichloromethane) afforded 110 mg of a brown impure material. The material was precipitated in diethyl ether/hexane (5 mL, 1:1) to afford *indole* **11b** as a beige solid (77 mg, 33%). δ_H (500 MHz, DMSO-d$_6$) 13.39 (1 H, br s, CO_2H), 11.76 (1 H, s, NH$_{indole}$), 8.51 – 8.44 (1 H, m, pyridinyl 6-H), 8.13 (1 H, s, NH$_{carbamoyl}$), 7.64 (1 H, td, *J* 7.6 and 1.9, pyridinyl 4-H), 7.57 (1 H, s, 4-H), 7.42 (1 H, d, *J* 8.8, 7-H), 7.24–7.16 (2 H, m, pyridinyl 5-H and 6-H), 7.11–7.06 (1 H, m, pyridinyl 3-H), 6.87 (1 H, d, *J* 3.8, thiophenyl 4-H), 6.76 (1 H, dd, *J* 3.8 and 1.3, thiophenyl 3-H), 5.94–5.69 (1 H, m, carbamoylethyl 1-H), 3.44–3.18 (3 H, m, carbamoylethyl 2-H$_A$ and pyridinylethyl 1-H$_2$) and 2.80–2.68 (3 H, m, carbamoylethyl 2-H$_B$ and pyridinylethyl 2-H$_2$). δ_C (125 MHz, DMSO-d$_6$) 169.8, 163.1, 159.0, 149.0, 147.6, 136.4, 134.5, 126.4, 126.0 (x2), 125.5, 124.2, 123.9, 123.03, 123.01 (x2), 121.4, 120.2, 114.4, 40.2, 38.4, 37.3 and 32.9. HRMS *m/z* calculated for $C_{23}H_{20}Cl_2N_3O_3S$ $(M+H)^+$: 488.0626; Found: 488.0618.

Methyl 5-chloro-3-[1-(5-chlorothiophen-2-yl)-2-{[2-(pyridin-2-yl)ethyl] carbamoyl}ethyl]-1H-indole-2-carboxylate

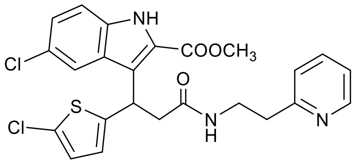

To a solution of crude indole **11b** (310 mg, 0.81 mmol) in methanol (30 mL), 15 drops of neat sulfuric acid were added and the reaction mixture was stirred at reflux for 40 h. The mixture was cooled down and partitioned between ethyl acetate/saturated aqueous sodium hydrogen carbonate solution (20/30 mL). The combined organic layers were dried over magnesium sulfate, filtered and concentrated *in vacuo*. Purification by column chromatography (silica, eluent: gradient 0:100 → 6:94 of

methanol:dichloromethane) afforded *indole* **45** as a beige solid (150 mg, 46% two steps). δ_H (500 MHz, chloroform-d) 8.84 (1 H, s, NH_{indole}), 8.42 (1 H, ddd, *J* 4.9, 1.9 and 0.9, pyridinyl 6-H), 7.58–7.51 (2 H, m, pyridinyl 4-H and 4-H), 7.27–7.22 (1 H, m, 7-H), 7.21 (1 H, dd, *J* 8.8 and 1.9, 6-H), 7.11 (1 H, ddd, *J* 7.6, 4.9 and 1.1, pyridinyl 5-H), 7.00 – 6.95 (1 H, m, pyridinyl 3-H), 6.67 (1 H, d, *J* 3.8, thiophenyl 4-H), 6.65 (1 H, dd, *J* 3.8 and 1.2, thiophenyl 3-H), 6.52–6.43 (1 H, m, $NH_{carbamoyl}$), 5.77 (1 H, ddd, *J* 8.3, 7.2 and 1.2, carbamoylethyl 1-H), 3.95 (3 H, s, methyl), 3.61–3.48 (2 H, m, pyridinylethyl 1-H_2), 3.18 (1 H, dd, *J* 14.3 and 7.2, carbamoylethyl 2-H_A), 3.02 (1 H, dd, *J* 14.3 and 8.3, carbamoylethyl 2-H_B), 2.82 (1 H, ddd, *J* 14.9, 7.1 and 5.0, pyridinylethyl 2-H_A) and 2.68 (1 H, ddd, *J* 14.9, 7.7 and 5.2, pyridinylethyl 2-H_B). δ_C (125 MHz, chloroform-d) 170.1, 162.1, 159.5, 149.2, 145.9, 136.6, 134.3, 128.2, 127.1, 126.5, 126.4, 125.7, 124.6, 123.42, 123.41, 123.1, 121.6, 121.2, 113.4, 52.5, 42.5, 38.5, 36.7 and 34.5. HRMS *m/z* calculated for $C_{24}H_{22}Cl_2N_3O_3S$ $(M+H)^+$: 502.0759; Found: 502.0769.

tert-Butyl *N*-[2-({5-chloro-3-[1-(5-chlorothiophen-2-yl)-2-{[2-(pyridin-2-yl)ethyl] carbamoyl}ethyl]-1H-indol-2-yl}formamido)ethyl]carbamate 11g

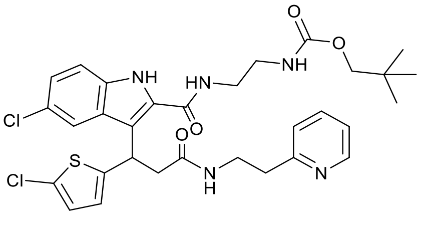

According to general procedure E3, indole **11b** (92 mg, 0.19 mmol) and Ghosez's reagent (50 µL, 0.38 mmol) dichloromethane solution (10 mL) and the N-Boc-ethylenediamine (125 µL, 1.14 mmol) and triethylamine (158 µL, 1.14 mmol) dichloromethane solution (6 mL) at 0°C gave the crude product. Purification by column chromatography (silica, eluent: gradient 0:100 → 5:95 of methanol:dichloromethane) afforded 44 mg of a colourless impure material. The material was precipitated in dichloromethane:hexane (2 mL, 1:1), filtered and washed with diethyl ether (2 x 2 mL) to afford *carbamate* **11g** as a white solid (34 mg, 28%). δ_H (500 MHz, chloroform-d) 9.41–9.28 (1 H, m, NH_{indole}), 9.17 (1 H, s, $NH_{formamido}$), 8.39–8.30 (1 H, m, pyridinyl 6-H), 7.45 (1 H, td, *J* 7.7 and 1.9, pyridinyl 4-H), 7.28–7.22 (2 H, m, indolyl 4- and 7-H), 7.12 (1 H, dd, *J* 8.7 and 2.0, indolyl 6-H), 7.07 (1 H, ddd, *J* 7.7, 4.9 and 1.1, pyridinyl 5-H), 6.99–6.86 (1 H, m, $NH_{carbamoyl}$), 6.82 (1 H, app. d, *J* 7.7, pyridinyl 3-H), 6.66 (1 H, d, *J* 3.8, thiophenyl 4-H), 6.45 (1 H, dd, *J* 3.8 and 1.5, thiophenyl 3-H), 5.96–5.50

(1 H, m, $NH_{carbamate}$), 5.28–5.18 (1 H, m, carbamoylethyl 1-H), 3.81–3.39 (6 H, m, formamidoethyl 1- and 2-H_2 and pyridinylethyl 1-H_2), 3.26 (1 H, dd, *J* 14.8 and 3.8, carbamoylethyl 2-H_A), 3.15 (1 H, dd, *J* 14.8 and 11.7, carbamoylethyl 2-H_B), 2.81 (1 H, ddd, *J* 14.8, 7.3 and 4.4, pyridinylethyl 2-H_A), 2.64 (1 H, ddd, *J* 14.8, 7.7 and 4.5, pyridinylethyl 2-H_B) and 1.41 (9 H, s, tert-butyl). δ_C (125 MHz, chloroform-d) 171.4, 162.8, 159.0, 156.3, 149.2, 145.4, 136.7, 134.1, 131.2, 128.6, 126.9, 125.9, 125.7, 124.7, 123.7, 123.3, 121.8, 120.6, 114.1, 113.4, 79.2, 41.3, 40.8, 40.4, 38.9, 36.2, 34.2 and 28.6 (x3). HRMS *m/z* calculated for $C_{30}H_{34}Cl_2N_5O_4S$ $(M+H)^+$: 630.1709; Found: 630.1706.

_N_-(2-Aminoethyl)-5-chloro-3-[1-(5-chlorothiophen-2-yl)-2-{[2-(pyridin-2-yl)ethyl] carbamoyl}ethyl]-1H-indole-2-carboxamide hydrochloride 11f

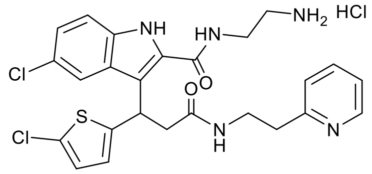

Cold hydrogen chloride (3N in methanol) was added to carbamate **11g** (20 mg, 0.03 mmol) and the reaction mixture was stirred at room temperature for 16 h. The solvent was removed *in vacuo* and the resulting material was precipitated in dichloromethane:hexane (2 mL, 1:1). The solid was filtered, washed with diethyl ether (1 x 3 mL) and dried *in vacuo* to afford *indole hydrochloride salt* **11f** as a white solid (18 mg, quantitative yield). δ_H (500 MHz, DMSO-d_6) 11.94 (1 H, s, NH_{indole}), 8.89 (1 H, t, *J* 5.5, $NH_{carboxamide}$), 8.65 (1 H, app. d, *J* 5.4, pyridinyl 6-H), 8.28 (1 H, app. t, *J* 5.8, $NH_{carbamoyl}$), 8.15–7.97 (4 H, m, pyridinyl 4-H and NH_2·HCl), 7.64 (1 H, br s, pyridinyl 5-H), 7.48–7.36 (3 H, m, 4- and 7-H and pyridinyl 3-H), 7.20 (1 H, dd, *J* 8.6 and 2.1, 6-H), 6.89 (1 H, d, *J* 3.9, thiophenyl 4-H), 6.77 (1 H, dd, *J* 3.9 and 1.3, thiophenyl 3-H), 5.53 (1 H, app. td, *J* 7.9 and 1.3, carbamoylethyl 1-H), 3.64–3.50 (2 H, m, aminoethyl 1-H_2), 3.45–3.42 (2 H, m, pyridinylethyl 1-H_2), 3.27 (1 H, dd, *J* 15.0 and 7.9, carbamoylethyl 2-H_A), 3.10 – 3.01 (2 H, m, aminoethyl 2-H_2), 2.99–2.90 (2 H, m, pyridinylethyl 2-H_2) and 2.84 (1 H, dd, *J* 15.0 and 7.7, carbamoylethyl 2-H_B). δ_C (125 MHz, DMSO-d_6) 170.6, 161.9, 159.4, 147.4, 134.1, 129.4 (x2), 126.3, 126.0 (x2), 125.7, 123.8, 123.7 (x2), 123.2 (x2), 119.8, 118.0, 114.0, 39.8, 38.5, 37.8, 36.8, 34.6 and 32.8. HRMS *m/z* calculated for $C_{25}H_{26}Cl_2N_5O_2S$ $(M+H)^+$: 530.1184; Found: 530.1185.

Ethyl 3-({5-chloro-3-[1-(5-chlorothiophen-2-yl)-2-{[2-(pyridin-2-yl)ethyl] carbamoyl}ethyl]-1H-indol-2-yl}formamido)propanoate 11c

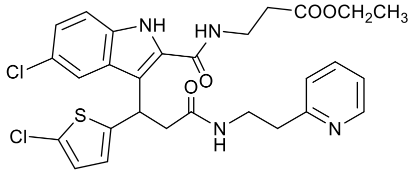

According to general procedure E3, indole **11b** (100 mg, 0.20 mmol) and Ghosez's reagent (166 µL, 1.25 mmol) dichloromethane solution (10 mL) and the ethyl 3-aminopropionate hydrochloride (308 mg, 2.0 mmol) and triethylamine (277 µL, 2.0 mmol) dichloromethane solution (6 mL) at 0°C gave the crude product. Purification by column chromatography (silica, eluent: gradient 0:100 → 4:96 of methanol:dichloromethane) afforded 44 mg of a colourless impure material. The material was precipitated in dichloromethane:hexane (5 mL, 1:1), filtered and washed with diethyl ether (2 x 2 mL) to afford *ester* **11c** as a white solid (32 mg, 27%). δ_H (500 MHz, chloroform-d) 9.38–9.22 (2 H, m, NH_{indole} and $NH_{formamido}$), 8.41 – 8.30 (1 H, m, pyridinyl 6-H), 7.43 (1 H, td, *J* 7.7 and 1.9, pyridinyl 4-H), 7.33–7.20 (2 H, m, indolyl 4- and 7-H), 7.11 (1 H, dd, *J* 8.7 and 2.0, indolyl 6-H), 7.06 (1 H, dd, *J* 7.7 and 5.0, pyridinyl 5-H), 6.77 (1 H, d, *J* 7.7, pyridinyl 3-H), 6.70 (1 H, app. t, *J* 5.5, $NH_{carbamoyl}$), 6.66 (1 H, d, *J* 3.8, thiophenyl 4-H), 6.44 (1 H, dd, *J* 3.8 and 1.5, thiophenyl 3-H), 5.29–5.16 (1 H, m, carbamoylethyl 1-H), 4.20–4.05 (2 H, m, ethyl 1-H_2), 3.90–3.80 (1 H, m, 3-H_A), 3.81–3.71 (1 H, m, 3-H_B), 3.58–3.38 (2 H, m, pyridinylethyl 1-H_2), 3.23 (1 H, dd, *J* 14.7 and 4.0, carbamoylethyl 2-H_A), 3.11 (1 H, dd, *J* 14.7 and 11.8, carbamoylethyl 2-H_B), 2.84–2.67 (3 H, m, 2-H_2 and pyridinylethyl 2-H_A), 2.59 (1 H, ddd, *J* 14.8, 7.9 and 4.6, pyridinylethyl 2-H_B) and 1.22 (3 H, t, *J* 7.1, ethyl 2-H_3). δ_C (125 MHz, chloroform-d) 171.9, 171.2, 162.6, 159.1, 149.2, 145.6, 136.6, 134.1, 131.3, 128.5, 126.9, 125.8, 125.6, 124.6, 123.6, 123.3, 121.7, 120.5, 114.3, 113.4, 60.8, 41.3, 38.7, 36.3, 36.1, 34.3, 34.1, 14.3. HRMS *m/z* calculated for $C_{28}H_{29}Cl_2N_4O_4S$ $(M+H)^+$: 587.1287; Found: 587.1285.

3-({5-Chloro-3-[1-(5-chlorothiophen-2-yl)-2-{[2-(pyridin-2-yl)ethyl]carbamoyl} ethyl]-1H-indol-2-yl}formamido)propanoic acid 11e

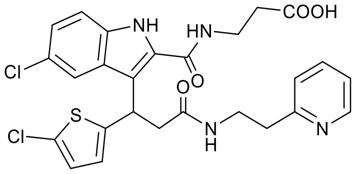

According to general procedure C, ester **11c** (21 mg, 0.04 mmol) and sodium hydroxide solution (0.4 mL, 0.3 M) in methanol:tetrahydrofuran (1 mL) gave the crude product. The material was precipitated in diethyl ether (2 mL), filtered and washed with diethyl ether (2 x 2 mL) to afford *acid* **11e** as a white solid (14 mg, 68%). δ_H (500 MHz, DMSO-d$_6$) 11.79 (1 H, s, NH$_{indole}$), 8.92 (1 H, t, *J* 5.4, NH$_{formamido}$), 8.55 (1 H, app. d, *J* 5.2, pyridinyl 6-H), 8.29 (1 H, t, *J* 5.8, NH$_{carbamoyl}$), 7.85 (1 H, br s, pyridinyl 4-H), 7.47 (1 H, br s, pyridinyl 5-H), 7.43 (1 H, d, *J* 8.7, indolyl 7-H), 7.40 (1 H, d, *J* 2.0, indolyl 4-H), 7.35–7.10 (1 H, m, pyridinyl 3-H), 7.19 (1 H, dd, *J* 8.7 and 2.0, indolyl 6-H), 6.89 (1 H, d, *J* 3.8, thiophenyl 4-H), 6.76 (1 H, dd, *J* 3.8 and 1.3, thiophenyl 3-H), 5.47–5.40 (1 H, m, carbamoylethyl 1-H), 3.62–3.51 (1 H, m, 3-H$_A$), 3.53–3.45 (1 H, m, 3-H$_B$), 3.46–3.39 (2 H, m, pyridinylethyl 1-H$_2$), 3.24 (1 H, dd, *J* 15.2 and 6.8, carbamoylethyl 2-H$_A$), 2.98 (1 H, dd, *J* 15.2 and 9.0, carbamoylethyl 2-H$_B$), 2.92–2.80 (2 H, m, pyridinylethyl 2-H$_2$) and 2.62–2.51 (2 H, m, 2-H$_2$); CO_2H not observed. δ_C (125 MHz, DMSO-d$_6$) 173.3, 171.3, 165.7, 161.8, 148.0, 134.6 (x2), 130.5 (x2), 126.7, 126.6 (x2), 126.3, 124.2, 123.9, 123.7 (x2), 120.3, 117.2, 114.5, 39.9, 38.5, 35.82, 35.78, 34.1, 33.4. HRMS *m/z* calculated for $C_{26}H_{25}Cl_2N_4O_4S$ $(M+H)^+$: 559.0974; Found: 559.0980.

***syn-* and *anti*-Methyl 3-(5-chloro-1H-indol-3-yl)-3-(5-chlorothiophen-2-yl)-2-methylpropanoate**

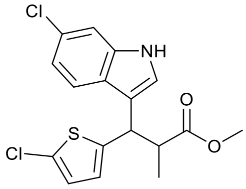

According to general procedure B2, indole **20** (100 mg, 0.23 mmol), [(1-methoxy-1-propenyl)oxy](trimethyl)silane (0.17 mL, 0.93 mmol) and trifluoromethanesulfonic acid (8 µL, 0.09 mmol) in dichloromethane (5 mL) gave the crude product. Purification by column chromatography (silica, eluent: gradient 0:100 → 20:80 of ethyl acetate:hexane) afforded *ester* **46** as a colourless oil (70 mg, 82%); R$_f$ = 0.22 (8:2 hexane:ethyl acetate); approximately a 7:3 mixture of diastereomers. δ_H (500 MHz, chloroform-d) 8.13 (0.7 H, app. s, NH^{maj}), 8.05 (0.3 H, app. s, NH^{min}), 7.48 (0.7 H, dt,

J 8.5 and 0.8, indolyl 4-H^{maj}), 7.43 (0.3 H, dt, *J* 8.5 and 0.7, indolyl 4-H^{min}), 7.35 (0.7 H, d, *J* 1.8, indolyl 7-H^{maj}), 7.31 (0.3 H, d, *J* 1.8, indolyl 7-H^{min}), 7.17 (0.7 H, dd, *J* 2.5 and 0.8, indolyl 2-H^{maj}), 7.13 (0.3 H, app. d, *J* 2.5, indolyl 2-H^{min}), 7.07 (0.7 H, dd, *J* 8.5 and 1.8, indolyl 5-H^{maj}), 7.04 (0.3 H, dd, *J* 8.5 and 1.8, indolyl 5-H^{min}), 6.75 (0.3 H, dd, *J* 3.7 and 0.6, thiophenyl 3-H^{min}), 6.70 (0.3 H, d, *J* 3.7, thiophenyl 4-H^{min}), 6.68 (0.7 H, dd, *J* 3.8 and 0.9, thiophenyl 3-H^{maj}), 6.66 (0.7 H, d, *J* 3.8, thiophenyl 4-H^{maj}), 4.68–4.60 (1 H, m, 3-H$^{maj/min}$), 3.64 (2 H, s, methyl^{maj}), 3.52 (1 H, s, methyl^{min}), 3.29 (0.7 H, dq, *J* 10.2 and 7.0, 2-H^{maj}), 3.18 (0.3 H, dq, *J* 10.3 and 6.9, 2-H^{min}), 1.24 (1 H, d, *J* 6.9, 2-methyl^{min}) and 1.16 (2 H, d, *J* 7.0, 2-methyl^{maj}). δ_C (125 MHz, chloroform-d) 175.9 (maj), 175.8 (min), 146.3 (maj), 145.3 (min), 136.8 (maj), 136.6 (min), 128.6 (maj), 128.2 (min), 125.62 (min), 125.57 (maj), 125.2 (maj), 124.8 (min), 123.8 (maj/min), 122.9 (maj/min), 121.5 (maj/min), 120.8 (maj), 120.6 (min), 120.3 (min), 120.2 (maj), 118.2 (min), 116.4 (maj), 111.4 (maj), 111.2 (min), 52.1 (maj), 52.0 (min), 46.1 (maj), 45.8 (min), 41.62 (maj), 41.57 (min), 16.74 (min) and 16.66 (maj). HRMS *m/z* calculated for $C_{17}H_{15}Cl_2NNaO_2S$ $(M+Na)^+$: 390.0098; Found: 390.0084.

syn- and _anti-_Methyl 3-(2-bromo-5-chloro-1H-indol-3-yl)-3-(5-chloro thiophen-2-yl)-2-methylpropanoate

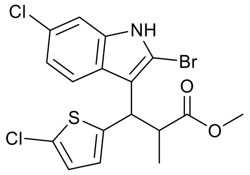

According to general procedure D, ester **46** (156 mg, 0.42 mmol) and pyridinium tribromide (148 mg, 0.46 mmol) in tetrahydrofuran:chloroform (10 mL) gave the crude product. Purification by column chromatography (silica, eluent: gradient 0:100 → 20:80 of ethyl acetate:hexane) afforded *ester* **47** as a colourless oil (184 mg, 95%); approximately a 7:3 mixture of diastereomers. δ_H (500 MHz, chloroform-d) 8.23 (0.7 H, s, NH^{maj}), 8.16 (0.3 H, s, NH^{min}), 7.61 (0.3 H, d, *J* 8.6, indolyl 4-H^{min}), 7.47 (1 H, d, *J* 8.6, indolyl 4-H^{maj}), 7.28 (0.7 H, d, *J* 1.9, indolyl 7-H^{maj}), 7.22 (0.3 H, d, *J* 1.9, indolyl 7-H^{min}), 7.12–7.05 (1 H, m, indolyl 5-H$^{maj/min}$), 6.81 (0.3 H, dd, *J* 3.8 and 0.7, thiophenyl 3-H^{min}), 6.73 (0.7 H, dd, *J* 3.8 and 1.0, thiophenyl 3-H^{maj}), 6.70 (0.3 H, d, *J* 3.8, thiophenyl 4-H^{min}), 6.65 (0.7 H, d, *J* 3.8, thiophenyl 4-H^{maj}), 4.67 (0.7 H, dd, *J* 11.4 and 1.0, 3-H^{maj}), 4.59–4.53 (0.3 H, m, 3-H^{min}), 3.71 (2 H, s, methyl^{maj}), 3.67–3.51 (1 H, m, 2-H$^{maj/min}$), 3.41 (1 H, s, methyl^{min}), 1.34 (1 H, d, *J* 6.9, 2-methyl^{min}) and 1.06 (2 H, d, *J* 7.0, 2-methyl^{maj}). δ_C (125 MHz, chloroform-d) 176.12 (maj), 175.21 (min), 144.67

(maj), 143.42 (min), 136.78 (maj), 136.57 (min), 128.96 (maj), 128.69 (min), 128.54 (min), 128.41 (maj), 125.62 (min), 125.53 (maj), 124.70 (min), 124.53 (maj), 124.38 (min), 123.68 (maj), 121.39 (maj), 121.22 (min), 120.19 (min), 119.82 (maj), 115.83 (min), 114.91 (maj), 110.95 (maj), 110.79 (min), 110.20 (maj), 109.51 (min), 52.25 (maj), 51.86 (min), 44.28 (min), 44.07 (maj), 43.03 (min), 42.02 (maj), 17.33 (min) and 17.04 (maj). HRMS *m/z* calculated for $C_{17}H_{14}BrCl_2NNaO_2S$ $(M+Na)^+$: 467.9204; Found: 467.9186.

***syn*- and *anti*-3-(2-Bromo-6-chloro-1H-indol-3-yl)-3-(5-chlorothiophen-2-yl)-2-methylpropanoic acid**

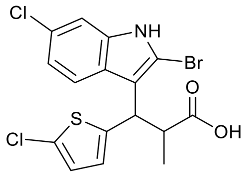

According to general procedure C, ester **47** (161 mg, 0.36 mmol) and sodium hydroxide solution (2 mL, 0.6 M) in methanol:tetrahydrofuran (2 mL) at 60 °C afforded *acid* **48** as white solid (140 mg, 90%); approximately a 7:3 mixture of diastereomers. δ_H (500 MHz, methanol-d$_4$) 7.68 (0.3 H, dd, *J* 8.6 and 0.6, indolyl 4-H^{min}), 7.49 (0.7 H, dd, *J* 8.6 and 0.6, indolyl 4-H^{maj}), 7.31 (0.7 H, dd, *J* 1.9 and 0.6, indolyl 7-H^{maj}), 7.25 (0.3 H, dd, *J* 1.9 and 0.6, indolyl 7-H^{min}), 7.03 (0.3 H, dd, *J* 8.6 and 1.9, indolyl 5-H^{min}), 7.02 (0.7 H, dd, *J* 8.6 and 1.9, indolyl 5-H^{maj}), 6.91 (0.3 H, dd, *J* 3.8 and 0.7, thiophenyl 3-H^{min}), 6.85 (0.7 H, dd, *J* 3.8 and 1.0, thiophenyl 3-H^{maj}), 6.76 (0.3 H, d, *J* 3.8, thiophenyl 4-H^{min}), 6.72 (0.7 H, d, *J* 3.8, thiophenyl 4-H^{maj}), 4.65 (0.7 H, dd, *J* 11.5 and 1.0, 3-H^{maj}), 4.55 (0.3 H, dd, *J* 11.4 and 0.7, 3-H^{min}), 3.67–3.56 (0.3 H, m, 2-H^{min}), 3.54 (0.7 H, dq, *J* 11.5 and 7.0, 2-H^{maj}), 1.30 (1 H, d, *J* 6.8, 2-methyl^{min}) and 1.02 (2 H, d, *J* 7.0, 2-methyl^{maj}). δ_C (125 MHz, methanol-d$_4$) 179.3 (maj), 178.4 (min), 147.1 (maj), 145.7 (min), 138.6 (maj), 138.3 (min), 129.1 (maj), 128.84 (min), 128.79 (min), 128.76 (maj), 126.7 (min), 126.5 (maj), 125.8 (maj), 125.7 (min), 125.6 (min), 124.7 (maj), 121.30 (maj), 121.28 (min), 121.0 (min), 120.6 (maj), 116.1 (min), 115.0 (maj), 111.9 (maj), 111.8 (maj), 111.6 (min), 111.1 (min), 45.3 (min), 45.0 (maj), 44.2 (min), 43.0 (maj), 17.7 (min) and 17.4 (maj). HRMS *m/z* calculated for $C_{16}H_{12}BrCl_2NO_2S$ $(M+H)^+$: 430.9149; Found: 430.9133.

syn- and _anti_-3-(2-Bromo-6-chloro-1H-indol-3-yl)-3-(5-chlorothiophen-2-yl)-2-methyl-_N_-[2-(pyridin-2-yl)ethyl]propenamide

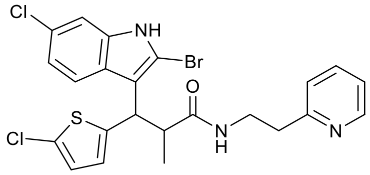

According to general procedure E2, carboxylic acid **48** (130 mg, 0.30 mmol), 2-(2-pyridyl)ethylamine (54 µL, 0.45 mmol), DIPEA (160 µL, 0.92 mmol) and HCTU (165 mg, 0.40 mmol) in dimethylformamide (3 mL) at RT gave the crude product. Purification by column chromatography (silica, eluent: gradient 0:100 → 100:0 of methanol:dichloromethane) afforded two products as colourless materials. Diastereomers *syn*-**49** and *anti*-***49*** were isolated in a 7:3 ratio, respectively.

Fraction one was precipitated in diethyl ether/hexane (2 mL, 1:1), filtered and washed with diethyl ether (2 x 2 mL) to afford *amide syn*-**49** as a white solid (90 mg, 80%). R_f = 0.39 (ethyl acetate); δ_H (500 MHz, DMSO-d_6) 12.04 (1 H, s, NH_{indole}), 8.50 (1 H, ddd, *J* 4.9, 1.9 and 0.9, pyridinyl 6-H), 8.17 (1 H, t, *J* 5.7, NH_{amide}), 7.69 (1 H, td, *J* 7.6 and 1.9, pyridinyl 4-H), 7.57 (1 H, d, *J* 8.6, indolyl 4-H), 7.33 (1 H, d, *J* 2.0, indolyl 7-H), 7.22 (1 H, ddd, *J* 7.6, 4.9 and 1.2, pyridinyl 5-H), 7.17 (1 H, app. dt, *J* 7.6 and 1.2, pyridinyl 3-H), 7.07 (1 H, dd, *J* 8.6 and 2.0, indolyl 5-H), 6.82 (1 H, d, *J* 3.8, thiophenyl 4-H), 6.75 (1 H, dd, *J* 3.8 and 1.0, thiophenyl 3-H), 4.49 (1 H, app. d, *J* 11.4, 3-H), 3.52 – 3.43 (1 H, m, 2-H), 3.47–3.35 (2 H, m, pyridinylethyl 1-H_2), 2.87–2.72 (2 H, m, pyridinylethyl 2-H_2) and 0.75 (3 H, d, *J* 6.7, 2-methyl). δ_C (125 MHz, DMSO-d_6) 174.1, 159.0, 149.1, 145.8, 136.8, 136.4 (x2), 126.6, 126.0, 125.5, 123.6, 123.1 (x2), 121.5, 119.8, 119.6, 113.9, 110.8, 42.6, 40.9, 38.2, 37.2 and 17.4. HRMS *m/z* calculated for $C_{23}H_{21}BrCl_2N_3OS$ $(M+Na)^+$: 535.9966; Found: 535.9978.

Fraction two was precipitated in diethyl ether/hexane (2 mL, 1:1), filtered and washed with diethyl ether (2 x 2 mL) to afford *amide anti*-**49** as a white solid (32 mg, 67%). R_f = 0.19 (ethyl acetate); δ_H (500 MHz, DMSO-d_6) 11.87 (1 H, s, NH_{indole}), 8.39 (1 H, ddd, *J* 4.8, 1.9 and 0.9, pyridinyl 6-H), 7.78 (1 H, d, *J* 8.6, indolyl 4-H), 7.75 (1 H, t, *J* 5.8, NH_{amide}), 7.54 (1 H, td, *J* 7.7 and 1.9, pyridinyl 4-H), 7.25 (1 H, d, *J* 2.0, indolyl 7-H), 7.14 (1 H, ddd, *J* 7.7, 4.8 and 1.2, pyridinyl 5-H), 7.00 (1 H, dd, *J* 8.6 and 2.0, indolyl 5-H), 6.92 (1 H, d, *J* 3.8, thiophenyl 3-H), 6.90 (1 H, d, *J* 3.8, thiophenyl 4-H), 6.78 (1 H, app. dt, *J* 7.7 and 1.2, pyridinyl 3-H), 4.49 (1 H, d, *J* 11.5, 3-H), 3.49 – 3.39 (1 H, m, 2-H), 3.22–3.12 (1 H, m, pyridinylethyl 1-H_A), 3.10–3.01 (1 H, m, pyridinylethyl 1-H_B), 2.46–2.36 (1 H, m, pyridinylethyl 2-H_A), 2.33–2.22 (1H, m, pyridinylethyl 2-H_B)

and 1.06 (3 H, d, *J* 6.6, 2-methyl). δ_C (125 MHz, DMSO-d_6) 173.2, 158.8, 148.9, 145.4, 136.5, 136.2, 126.21, 126.15, 125.8, 124.7, 124.0, 122.7, 121.3, 120.6, 119.5, 114.9, 110.4, 110.3, 43.5, 42.2, 37.9, 37.0 and 17.8. HRMS *m/z* calculated for $C_{23}H_{21}BrCl_2N_3OS$ $(M+Na)^+$: 535.9966; Found: 535.9975.

These compounds were poorly soluble in aqueous buffer and therefore no binding affinity was determined.

syn -3-(2-Bromo-6-chloro-1H-indol-3-yl)-3-(5-chlorothiophen-2-yl)-2-methyl-_N_-[2-(pyridin-2-yl)ethyl]propenamide (_syn_-49)

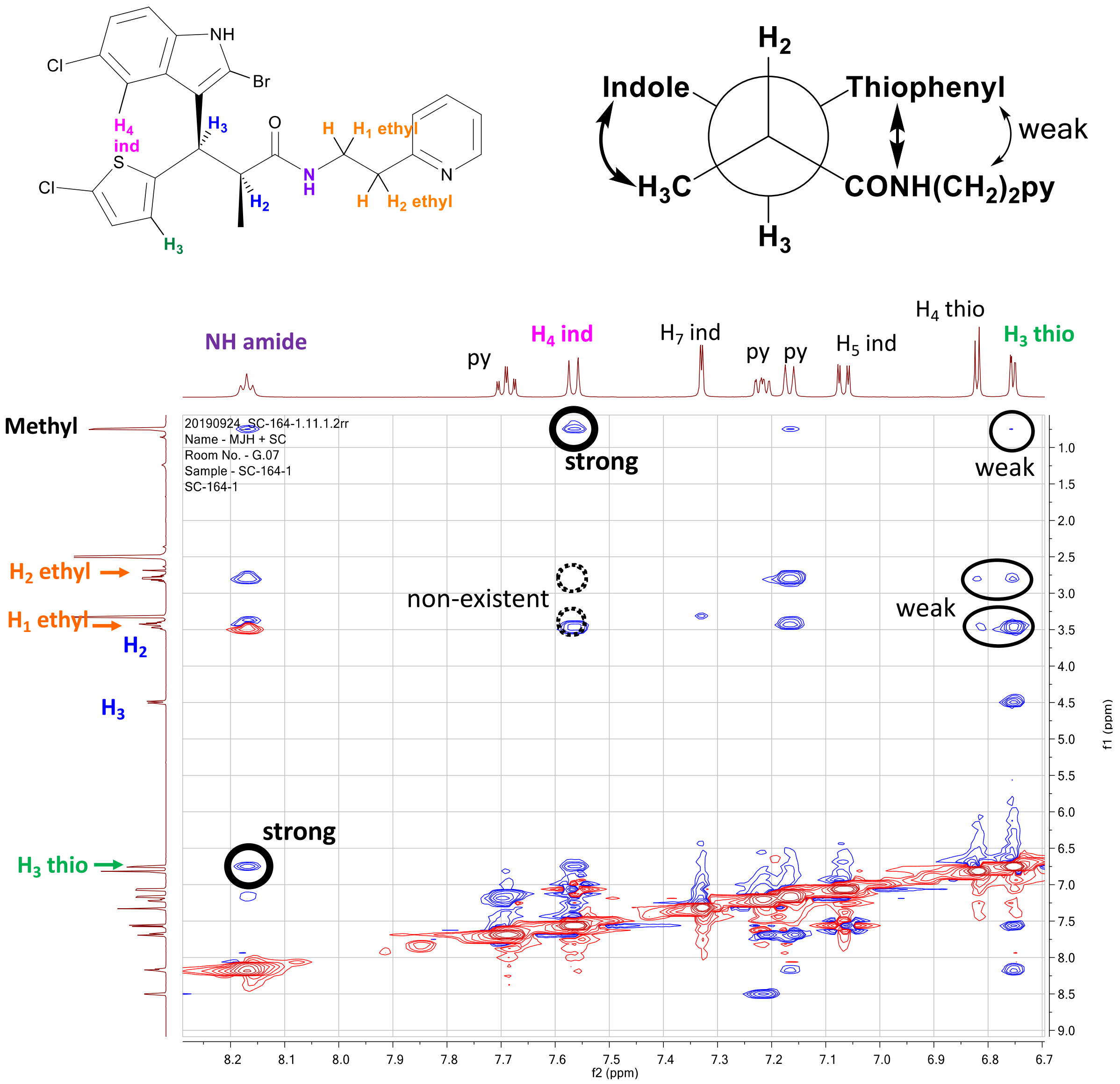

*Resolution of the relative configuration of syn-**49**. Top: chemical structure highlighting relevant protons and Newman projection. Bottom: extract of the ^{1}H-^{1}H NOESY correlation experiment.*

anti-3-(2-Bromo-6-chloro-1H-indol-3-yl)-3-(5-chlorothiophen-2-yl)-2-methyl-_N_-[2-(pyridin-2-yl)ethyl]propenamide (_anti_-49)

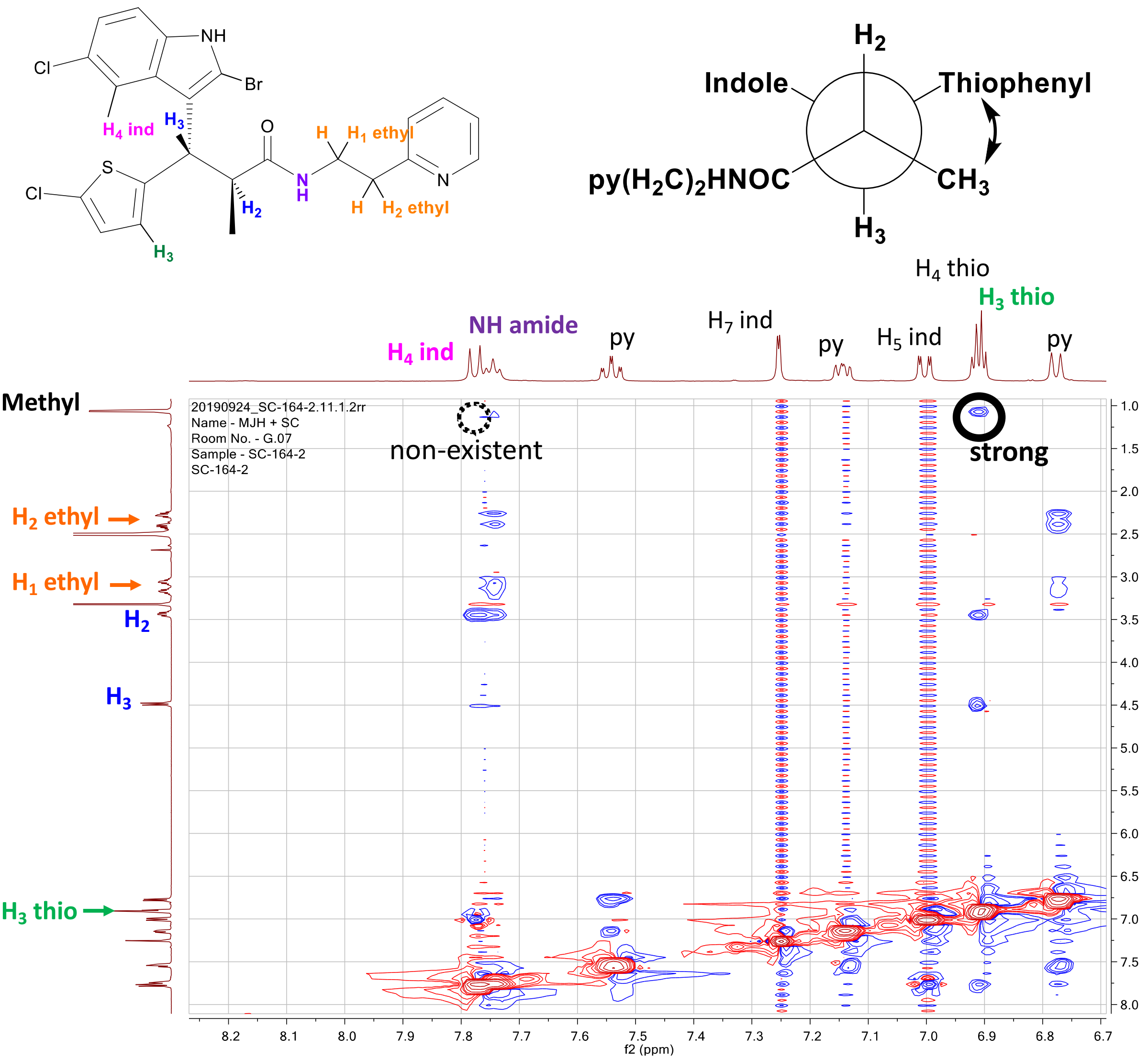

*Resolution of the relative configuration of anti-**49**. Top: chemical structure highlighting relevant protons and Newman projection. Bottom: extract of the ^{1}H-^{1}H NOESY correlation experiment.*

syn- and _anti_-3-(6-Chloro-1H-indol-3-yl)-3-(5-chlorothiophen-2-yl)-2-methylpropanoic acid

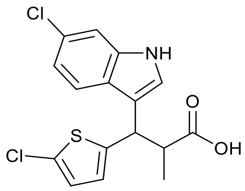

According to general procedure C, ester **46** (130 mg, 0.35 mmol) and sodium hydroxide solution (2 mL, 0.5 M) in methanol (5 mL) at 60 °C afforded *acid* **50** as white solid (128 mg, 99%); approximately a 6:4 mixture of diastereomers. δ_H (500 MHz, methanol-d4) 10.62 (0.6 H, s, NH^{maj}), 10.53 (0.4 H, s, NH^{min}), 7.46 (0.6 H, d, *J* 8.5,

indolyl 4-H^{maj}), 7.42 (0.4 H, d, *J* 8.5, indolyl 4-H^{min}), 7.36 (1 H, d, *J* 1.9, indolyl 7-H^{maj}), 7.32 (1 H, d, *J* 1.8, indolyl 7-H^{min}), 7.29–7.26 (1 H, m, indolyl 2-H^{min}), 7.23 (1 H, d, *J* 2.2, indolyl 2-H^{maj}), 6.97 (1 H, dd, *J* 8.5 and 1.9, indolyl 5-H^{maj}), 6.94 (1 H, dd, *J* 8.5 and 1.9, indolyl 5-H^{min}), 6.83 (1 H, dt, *J* 3.7 and 0.8, thiophenyl 3-H^{min}), 6.79 (1 H, dd, *J* 3.8 and 0.9, thiophenyl 3-H^{maj}), 6.75–6.72 (1 H, m, thiophenyl 4-H^{min}), 6.71–6.68 (1 H, m, thiophenyl 4-H^{maj}), 4.61 (1 H, d, *J* 10.5, 3-H^{min}), 4.61 (1 H, d, *J* 10.4, 3-H^{maj}), 3.31 – 3.23 (1 H, m, 2-H^{maj}), 3.16 (1 H, dq, *J* 10.5 and 6.9, 2-H^{min}), 1.21 (1.2 H, d, *J* 6.9, 2-methyl^{min}) and 1.12 (1.8 H, d, *J* 6.9, 2-methyl^{maj}). δ_C (125 MHz, methanol-d$_4$) 179.35 (C1^{maj}), 179.20 (C1^{min}), 148.76 (C2^{maj}), 147.72 (C2^{min}), 138.65 (C3^{maj}), 138.49 (C3^{min}), 138.47 (C3^{maj}), 138.31 (C3^{min}), 128.96 (C4^{min}), 128.58 (C4^{min}), 128.56 (C4^{maj}), 128.49 (C4^{maj}), 126.66 (C5^{min}), 126.62 (C6^{maj}), 126.59 (C6^{min}), 126.55 (C5^{maj}), 126.52 (C6^{maj}), 126.49 (C6^{min}), 126.03 (C7^{min}), 125.03 (C8^{maj}), 124.86 (C8^{maj}), 124.80 (C7^{maj}), 123.47 (C8^{min}), 123.30 (C8^{min}), 120.81 (C9^{maj}), 120.77 (C9^{min}), 120.51 (C10^{maj}), 120.35 (C10^{min}), 118.48 (C11^{maj}), 118.44 (C11^{min}), 116.49 (C12^{maj}), 116.45 (C12^{min}), 112.33 (C13^{maj}), 112.28 (C13^{maj}), 112.07 (C13^{min}), 112.02 (C13^{min}), 47.15 (C14^{maj}), 46.82 (C14^{min}), 42.70 (C15^{maj}), 42.66 (C15^{min}) and 17.15 (C16). HRMS *m/z* calculated for $C_{16}H_{13}Cl_2NNaO_2S$ $(M+Na)^+$: 375.9942; Found: 375.9930.

***syn-* and *anti*-3-(6-Chloro-1H-indol-3-yl)-3-(5-chlorothiophen-2-yl)-2-methyl-1-[3-(pyridin-2-yl)pyrrolidin-1-yl]propan-1-one 13**

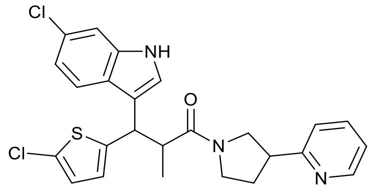

According to general procedure E2, acid **50** (65 mg, 0.18 mmol), 2-(pyrrolidin-3-yl)pyridine (41 mg, 0.28 mmol), DIPEA (96 µL, 0.55 mmol) and HCTU (116 mg, 0.28 mmol) in dimethylformamide (3 mL) at RT gave the crude product. Purification by column chromatography (silica, eluent: gradient 0:100 → 100:0 of ethyl acetate:hexane) afforded *amide* **13** as a white solid (62 mg, 71%); diastereomeric mixture of rotamers. δ_H (500 MHz, methanol-d$_4$) 8.54 – 8.40 (1 H, m, pyridinyl 6-H), 7.82–7.63 (1 H, m, pyridinyl 4-H), 7.57–7.49 (1 H, m, indolyl 4-H), 7.40–6.66 (7 H, m, indolyl 2-, 5- and 7-H, pyridinyl 3- and 5-H and thiophenyl 3- and 4-H), 4.70–4.56 (1 H, m, 3-H), 4.13–2.96 (6 H, m, 2-H and pyrrolidinyl 2-H$_2$, 3-H and 5-H$_2$), 2.43–1.63 (2 H, m, pyrrolidinyl 4-H$_2$) and 1.23–1.04 (3 H, 2-methyl). δ_C (125 MHz, methanol-d$_4$) δ

176.3 to 175.8 (C1), 162.1 to 161.2 (C2), 150.2 to 149.9 (C3), 149.0 to 148.9 (C4), 147.7 to 147.6 (C5), 138.8 to 138.3 (C6), 128.8 to 128.3 (C7), 126.9 to 122.5 (C8, C9, C10, C11, C12, C13 and C14), 121.2 to 120.8 (C15), 120.6 to 120.3 (C16), 118.8 to 116.6 (C17), 112.3 to 112.1 (C18), 53.0 to 51.9 (C19), 48.1 to 45.3 (C20, C21 and C22), 43.3 to 42.9 (C23), 33.4 to 31.4 (C24) and 17.2 to 17.0 (C25). HRMS *m/z* calculated for $C_{25}H_{24}Cl_2N_3OS$ $(M+H)^+$: 484.1017; Found: 484.1023.

***syn-* and *anti*-(6-Chloro-1H-indol-3-yl)-3-(5-chlorothiophen-2-yl)-2-methyl-*N*-[2-phenylcyclopropyl]propanamide**

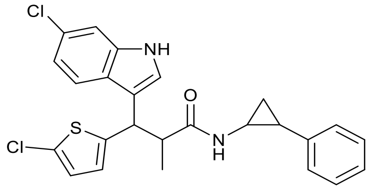

According to general procedure E2, acid **50** (65 mg, 0.18 mmol), 2-phenylcyclopropan-1-amine (37 mg, 0.28 mmol), DIPEA (96 µL, 0.55 mmol) and HCTU (116 mg, 0.28 mmol) in dimethylformamide (3 mL) at RT gave the crude product. Purification by column chromatography (silica, eluent: gradient 0:100 → 50:50 of ethyl acetate:hexane) afforded *amide* **51** as an off-white solid (54 mg, 64%); diastereomeric mixture. δ_H (500 MHz, methanol-d_4) 7.58–7.49 (1 H, m, indolyl 4-H), 7.38–7.32 (1 H, m, indolyl 2-H), 7.28–6.76 (6 H, m, indolyl 7-H and phenyl 2-, 3-, 4-, 5- and 6-H), 7.02–6.97 (1 H, m, indolyl 5-H), 6.90–6.69 (2 H, m, thiophenyl 3- and 4-H), 4.59–4.49 (1 H, m, 3-H), 3.19–2.98 (1 H, m, 2-H), 2.78–2.49 (1 H, m, cyclopropyl 1-H), 1.93–1.02 (1 H, m, cyclopropyl 2-H), 1.22–1.05 (3 H, m, 2-methyl) and 1.19–0.53 (2 H, m, cyclopropyl 3-H_2). δ_C (125 MHz, methanol-d_4) 179.2 to 178.9 (C1), 148.7 to 148.3 (C2), 142.2 to 142.1 (C3), 138.6 to 138.1 (C4), 129.3 to 125.2 (C5, C6, C7, C8, C9, C10, C11, C12, C13 and C14), 124.6 to 123.7 (C15), 121.2 to 120.9 (C16), 120.5 to 120.4 (C17), 118.3 to 116.9 (C18), 112.3 and 112.1 (C19), 48.3 to 47.9 (C20), 43.3 to 43.1 (C21), 33.1 to 32.8 (C22), 25.6 to 25.0 (C23), 17.6 to 17.4 (C24) and 16.5 to 15.4 (C25). HRMS *m/z* calculated for $C_{25}H_{22}Cl_2N_2NaOS$ $(M+Na)^+$: 491.0728; Found: 470.0722.

This compound was poorly soluble in aqueous buffer and therefore no binding affinity was determined.

(3R*)-3-(5-chloro-1H-indol-3-yl)-3-(5-chlorothiophen-2-yl)-1-[(3R*)-3-(pyridin-2-yl)pyrrolidin-1-yl]propan-1-one and (3R*)-3-(5-chloro-1H-indol-3-yl)-3-(5-chlorothiophen-2-yl)-1-[(3S*)-3-(pyridin-2-yl)pyrrolidin-1-yl]propan-1-one

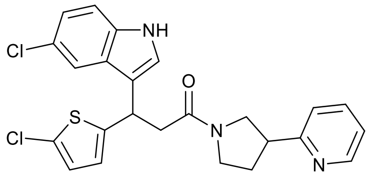

According to general procedure E2, the carboxylic acid **19** (65 mg, 0.19 mmol), 2-(pyrrolidin-3-yl)pyridine (54 mg, 0.38 mmol), DIPEA (100 µL, 0.57 mmol) and HCTU (160 mg, 0.38 mmol) in dimethylformamide (3 mL) at RT gave the crude product. Purification by column chromatography (silica, eluent: gradient 0:100 → 100:0 of ethyl acetate:hexane) afforded *amide* **52** as a white solid (90 mg, quantitative yield); diastereomeric mixture of rotamers. δ_H (500 MHz, methanol-d_4) 8.53-8.38 (1 H, m, pyridinyl 6-H), 7.79-7.60 (1 H, m, pyridinyl 4-H), 7.41–7.36 (1 H, m, indolyl 4-H), 7.36-7.28 (1 H, m, indolyl 7-H), 7.28–7.24 (2 H, m, indolyl 2-H and pyridinyl 5-H), 7.24-7.03 (2 H, m, indolyl 6-H and pyridinyl 3-H), 6.82–6.68 (2 H, m, thiophenyl 3- and 4-H), 5.00-4.91 (1 H, m, 3-H), 3.90–3.22 (5 H, m, pyrrolidinyl 3-H and 2- and 5-H_2), 3.21-3.08 (2 H, m, 2-H_2) and 2.27–1.83 (2 H, m, pyrrolidinyl 4-H_2). δ_C (125 MHz, methanol-d_4) 172.01 (C1), 171.99 (C1), 171.97 (C1), 171.96 (C1), 161.78 (C2), 161.72 (C2), 161.29 (C2), 161.21 (C2), 150.13 (C3), 150.10 (C3), 150.07 (C3), 150.03 (C3), 149.22 (C4), 149.17 (C4), 149.09 (C4), 138.74 (C5), 138.73 (C5), 138.71 (C5), 138.70 (C5), 136.68 (C6), 136.66 (C6), 128.71 (C7), 128.68 (C7), 128.66 (C7), 128.61 (C8), 128.59 (C8), 128.57 (C8), 126.91 (C9), 126.89 (C9), 125.74 (C10), 125.72 (C10), 125.13 (C11), 125.09 (C11), 125.06 (C11), 124.99 (C11), 124.91 (C12), 124.89 (C12), 124.85 (C12), 124.84 (C12), 123.58 (C13), 123.55 (C13), 123.52 (C13), 123.46 (C14), 123.28 (C14), 123.22 (C14), 122.91 (C15), 122.88 (C15), 119.23 (C16), 119.21 (C16), 117.93 (C17), 117.89 (C17), 117.74 (C17), 117.65 (C17), 113.76 (C18), 113.76 (C18), 113.73 (C18), 53.06 (C19), 52.96 (C19), 52.08 (C19), 52.04 (C19), 47.94 (C20), 47.90 (C20), 47.07 (C20), 47.02 (C20), 46.83 (C21), 45.58 (C21), 45.51 (C21), 42.70 (C22), 42.58 (C22), 42.45 (C22), 42.37 (C22), 36.49 (C23), 36.41 (C23), 36.37 (C23), 33.24 (C24), 33.17 (C24), 31.72 (C24) and 31.60 (C24). HRMS *m/z* calculated for $C_{24}H_{22}Cl_2N_3OS$ $(M+H)^+$: 470.0861; Found: 470.0875.

This compound was poorly soluble in aqueous buffer and therefore no binding affinity was determined.

3-(5-Chloro-1H-indol-3-yl)-3-(5-chlorothiophen-2-yl)-*N*-[2-phenyl cyclopropyl]propanamide 7a

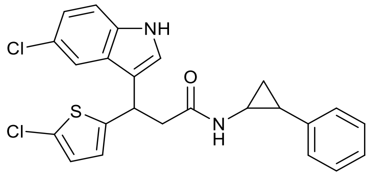

According to general procedure E2, acid **19** (65 mg, 0.19 mmol), 2-phenylcyclopropan-1-amine (45 mg, 0.38 mmol), DIPEA (100 µL, 0.57 mmol) and HCTU (160 mg, 0.38 mmol) in dimethylformamide (3 mL) at RT gave the crude product. Purification by column chromatography (silica, eluent: gradient 0:100 → 50:50 of ethyl acetate:hexane) afforded *amide* **7a** as an off-white solid (47 mg, 55%); diastereomeric mixture (it seems that 3 main isomers coexist). δ_H (500 MHz, methanol-d_4) 7.41–7.37 (1 H, m, indolyl 4-H), 7.32 (1 H, app. d, *J* 8.6, indolyl 7-H), 7.23 (1 H, s, indolyl 2-H), 7.24–7.16 (2 H, m, phenyl 3- and 5-H), 7.15–7.10 (1 H, m, phenyl 4-H), 7.08–7.04 (1 H, m, indolyl 6-H), 7.08–6.99 (2 H, m, phenyl 2- and 6-H), 6.80–6.77 (1 H, m, thiophenyl 4-H), 6.77–6.74 (1 H, m, thiophenyl 3-H), 4.92–4.87 (1 H, m, 3-H), 2.97–2.89 (2 H, m, 2-H_2), 2.76–2.68 (1 H, m, cyclopropyl 1-H), 1.79–1.63 (1 H, m, cyclopropyl 2-H), 1.13–1.06 (1 H, m, cyclopropyl 3-H_A) and 0.95–0.90 (1 H, m, cyclopropyl 3-H_B). δ_C (125 MHz, methanol-d_4) 174.81 (C1), 174.79 (C1), 149.08 (C2), 149.04 (C2), 142.15 (C3), 142.12 (C3), 136.69 (C4), 136.64 (C4), 129.30 (C5), 129.26 (C6), 128.68 (C7), 128.62 (C7), 128.60 (C7), 127.17 (C8, C9, C10), 126.95 (C11), 126.92 (C11), 126.80 (C12), 126.79 (C12), 125.73 (C13), 125.70 (C13), 124.94 (C14), 124.86 (C14), 124.82 (C15), 124.78 (C15), 122.88 (C16), 119.31 (C17), 119.30 (C17), 117.44 (C18), 113.67 (C19), 44.22 (C20), 44.16 (C20), 36.59 (C21), 36.56 (C21), 33.11 (C22), 33.09 (C22), 25.27 (C23), 25.18 (C23), 15.95 (C24) and 15.89 (C24). HRMS *m/z* calculated for $C_{24}H_{20}Cl_2N_2NaOS$ $(M+Na)^+$: 477.0571; Found: 477.0564.

5-chloro-3-[1-(5-chlorothiophen-2-yl)-2-{[2-(pyridin-2-yl)cyclopropyl]carbamoyl} ethyl]-1H-indole-2-carboxylic acid 14

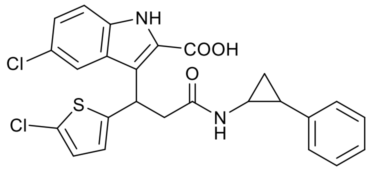

According to general procedure E2, indole **44** (160 mg, 0.41 mmol), 2-phenylcyclopropan-1-amine (54 mg, 0.41 mmol), DIPEA (0.22 mL, 1.2 mmol) and HCTU (169 mg, 0.41 mmol) in dimethylformamide (9 mL) at 0 °C gave the crude product. Purification by column chromatography (silica, eluent: gradient 0:100 → 5:95 of methanol:dichloromethane) afforded 160 mg of an impure material. Dichloromethane (3 mL) was added to the material and 6-chloro-1-hydroxybenzotriazole from HCTU precipitated. The filtrate was concentrated *in vacuo* and purification by column chromatography (silica, eluent: gradient 0:100 → 2.5:97.5 of methanol:dichloromethane) afforded *amide* **14** as a white solid (15 mg, 7%). δ_H (500 MHz, DMSO-d_6) 11.23 (1 H, s, CO_2H), 8.98 – 8.64 (1 H, m, NH_{indole}), 7.45 – 7.37 (2 H, m, indolyl 4-H and indolyl 7-H), 7.25 – 7.19 (2 H, m, phenyl 2-H and 6-H), 7.15 – 7.10 (1 H, m, phenyl 4-H), 7.10 – 6.99 (3 H, m, indolyl 6-H and phenyl 3-H and 5-H), 6.92 – 6.86 (1 H, m, thiophenyl 4-H), 6.84 – 6.80 (1 H, m, thiophenyl 3-H), 6.20 – 6.11 (1 H, m, carbamoylethyl 1-H), 3.37 – 3.24 (1 H, m, carbamoylethyl 2-H_A), 2.83 – 2.71 (2H, carbamoylethyl 2-H_B and cyclopropyl 1-H), 1.90 – 1.69 (1 H, m, cyclopropyl 2-H), 1.17 – 1.02 (2 H, m, cyclopropyl 3-H_A and 3-H_B). $NH_{carbamoyl}$ is not observed. δ_C (125 MHz, DMSO-d_6) δ 171.13, 171.05, 148.81, 141.50, 141.42, 133.39, 128.17 (x2), 127.00, 125.90, 125.72, 125.69, 125.51, 125.32, 125.30, 122.87, 122.78, 122.73, 122.03, 119.48, 113.92, 40.67, 40.62, 32.73, 32.46, 32.36, 23.85, 23.70, 15.32, 14.97. HRMS *m/z* calculated for $C_{25}H_{21}Cl_2N_2O_3S$ $(M+H)^+$: 499.0650; Found: 499.0646.

3-{5-Chloro-2-[3-(pyridin-2-yl)pyrrolidine-1-carbonyl]-1H-indol-3-yl}-3-(5-chlorothiophen-2-yl)-1-[3-(pyridin-2-yl)pyrrolidin-1-yl]propan-1-one

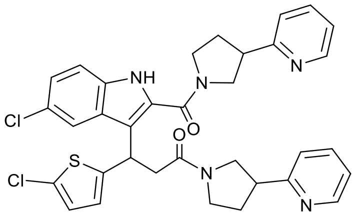

According to general procedure E2, indole **44** (20 mg, 0.05 mmol), 2-(pyrrolidin-3-yl)pyridine (11 mg, 0.08 mmol), DIPEA (26 µL, 0.15 mmol) and HCTU (31 mg, 0.08 mmol) in dimethylformamide (2 mL) at 0 °C gave the crude product. Purification by column chromatography (silica, eluent: gradient 0:100 → 5:95 of methanol:dichloromethane) afforded *ketone* **15** as a colourless material (11 mg, 33%). δ_H (500 MHz, methanol-d$_4$) 8.56–8.20 (2 H, m, pyridinyl 6-H$_A$ and 6-H$_B$), 7.86–6.70 (11 H, m, pyridinyl 3-, 4- and 5-H$_A$ and pyridinyl 3-, 4- and 5-H$_B$, indolyl 4-, 6- and 7-H and thiophenyl 3- and 4-H), 5.22–5.01 (1 H, m, 3-H), 4.10–3.05 (10 H, m, 2-H$_2$, pyrrolidinyl 2- and 5-H$_{2A}$ and pyrrolidinyl 2- and 5-H$_{2B}$) and 2.48–1.23 (6 H, m, pyrrolidinyl 3-H$_A$ and 4-H$_{2A}$ and pyrrolidinyl 3-H$_B$ and 4-H$_{2B}$). δ_C (125 MHz, methanol-d$_4$) 171.76, 171.74, 171.72, 171.58, 171.56, 165.22, 165.17, 165.08, 165.03, 165.00, 164.98, 162.14, 162.08, 161.89, 161.74, 161.59, 161.46, 161.42, 161.19, 161.12, 160.80, 150.24, 150.15, 150.13, 150.12, 150.11, 150.00, 149.95, 149.90, 148.00, 147.92, 147.89, 138.76, 138.68, 138.65, 138.63, 138.62, 138.58, 138.53, 135.59, 135.53, 135.46, 132.18, 128.76, 128.71, 128.20, 127.99, 126.80, 126.68, 126.61, 124.92, 124.85, 124.74, 124.65, 124.01, 123.90, 123.73, 123.70, 123.62, 123.58, 123.53, 123.51, 123.43, 123.34, 123.28, 123.05, 122.92, 122.90, 122.64, 120.48, 120.45, 120.41, 120.38, 120.33, 117.52, 117.48, 117.40, 117.32, 117.24, 114.44, 114.37, 114.33, 54.90, 54.85, 54.80, 54.73, 54.12, 53.04, 52.98, 52.95, 52.93, 52.92, 52.24, 52.22, 52.17, 52.12, 52.10, 52.02, 47.89, 47.12, 47.08, 47.06, 47.05, 47.00, 46.98, 46.87, 46.82, 46.79, 46.73, 46.08, 45.56, 45.50, 45.43, 41.12, 41.04, 40.87, 40.76, 40.69, 40.51, 35.62, 35.58, 35.55, 35.47, 35.43, 35.41, 33.39, 33.33, 33.31, 33.18, 33.14, 33.13, 33.06, 33.03, 33.00, 31.96, 31.75, 31.71, 31.66, 31.66, 31.62, 31.61, 31.59, 31.51, 31.45, 31.44, 30.45, 27.55, 27.28.. HRMS *m/z* calculated for $C_{23}H_{20}Cl_2N_3O_3S$ $(M+H)^+$: 488.0626; Found: 488.0618.

This compound was poorly soluble in aqueous buffer and therefore no binding affinity was determined.

7. Peptide Characterization data

HPLC and ESI-MS data for wt GKAP peptide

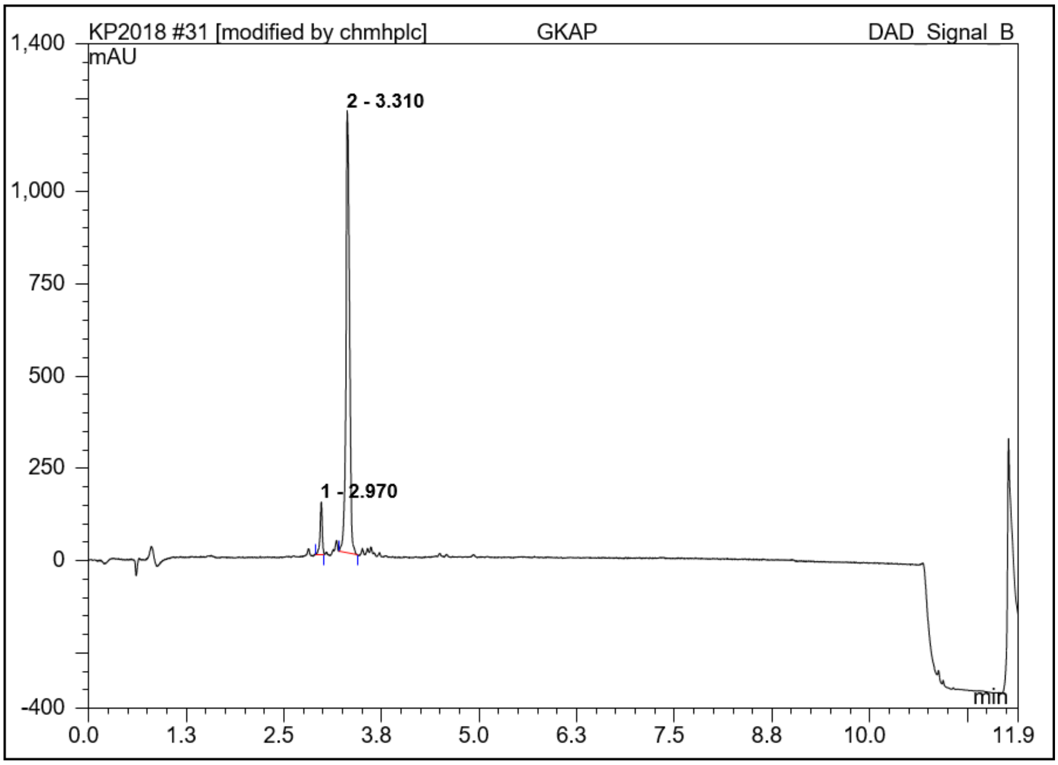

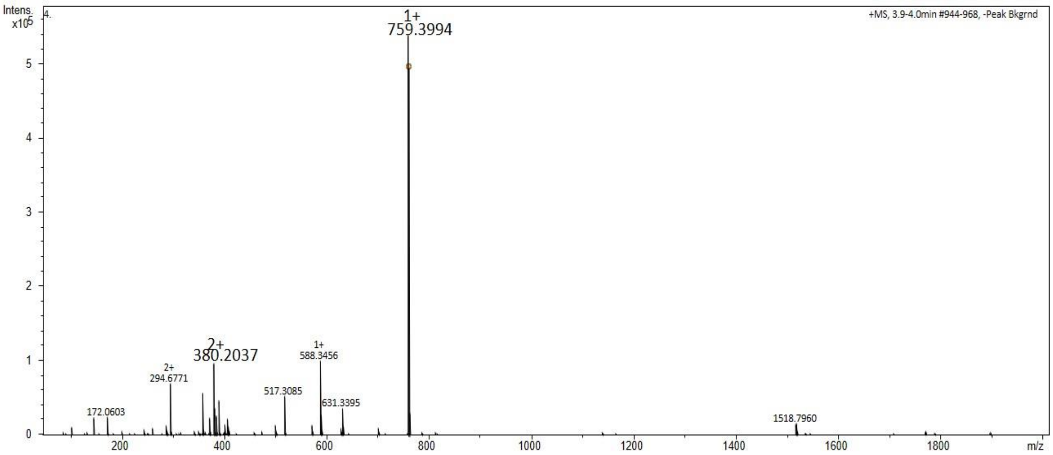

HPLC and ESI-MS data for FITC-Ahx-GKAP peptide

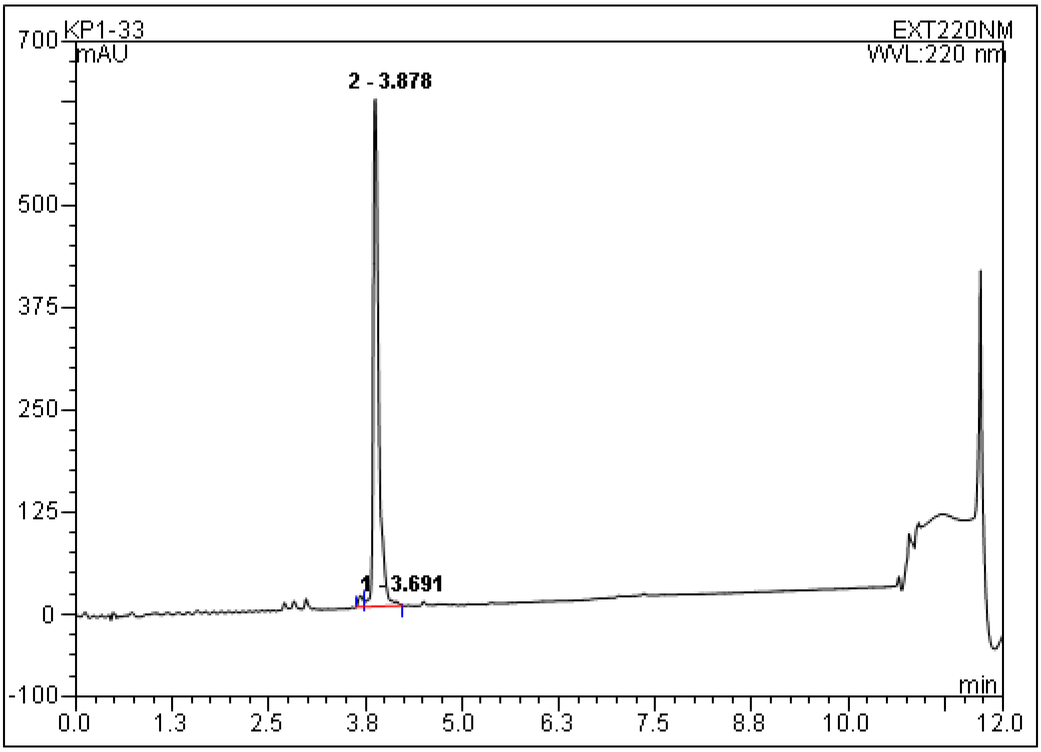

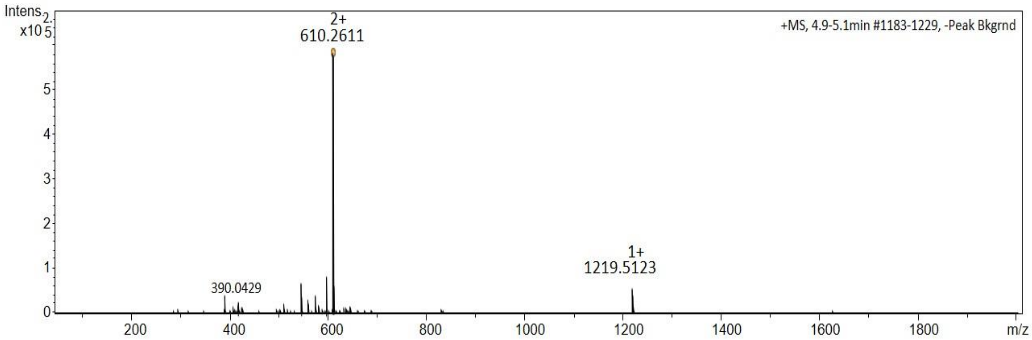

HPLC and ESI-MS data for L6F GKAP peptide

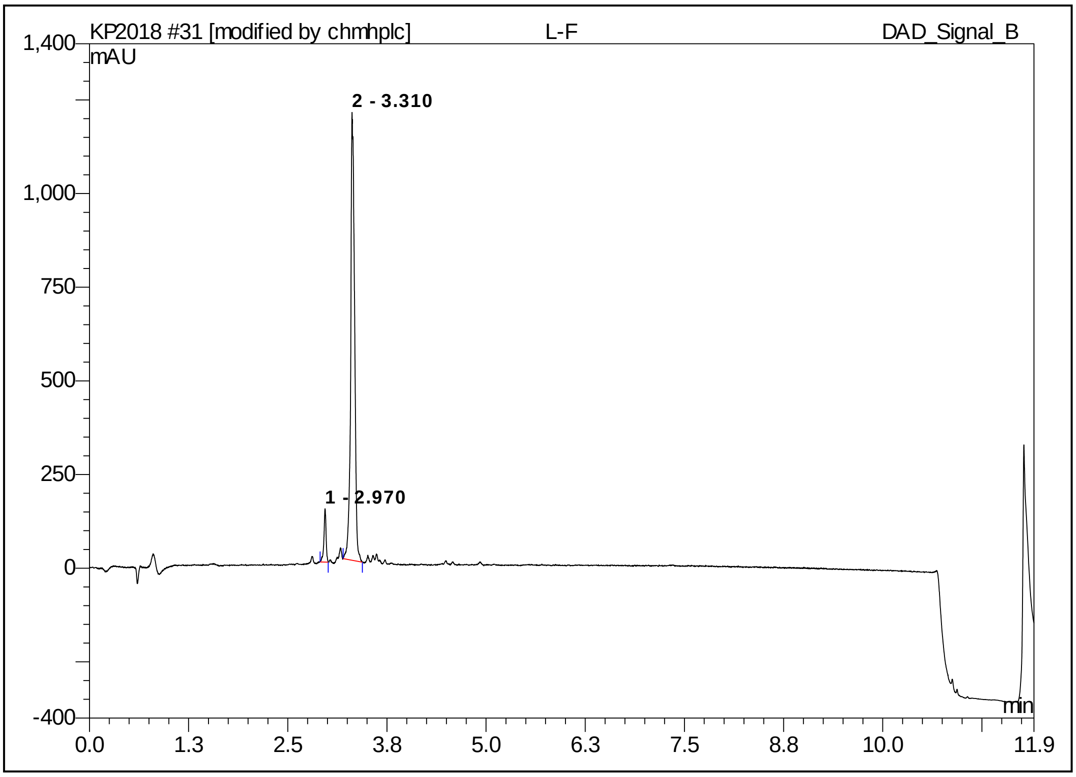

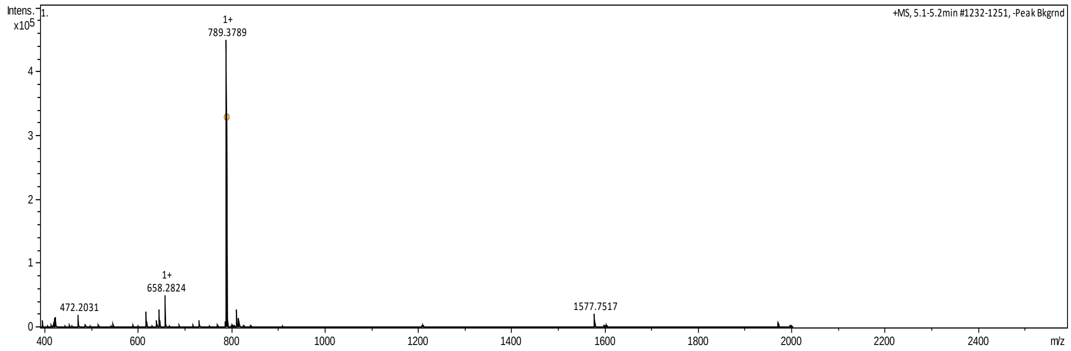

8. Protein Characterization

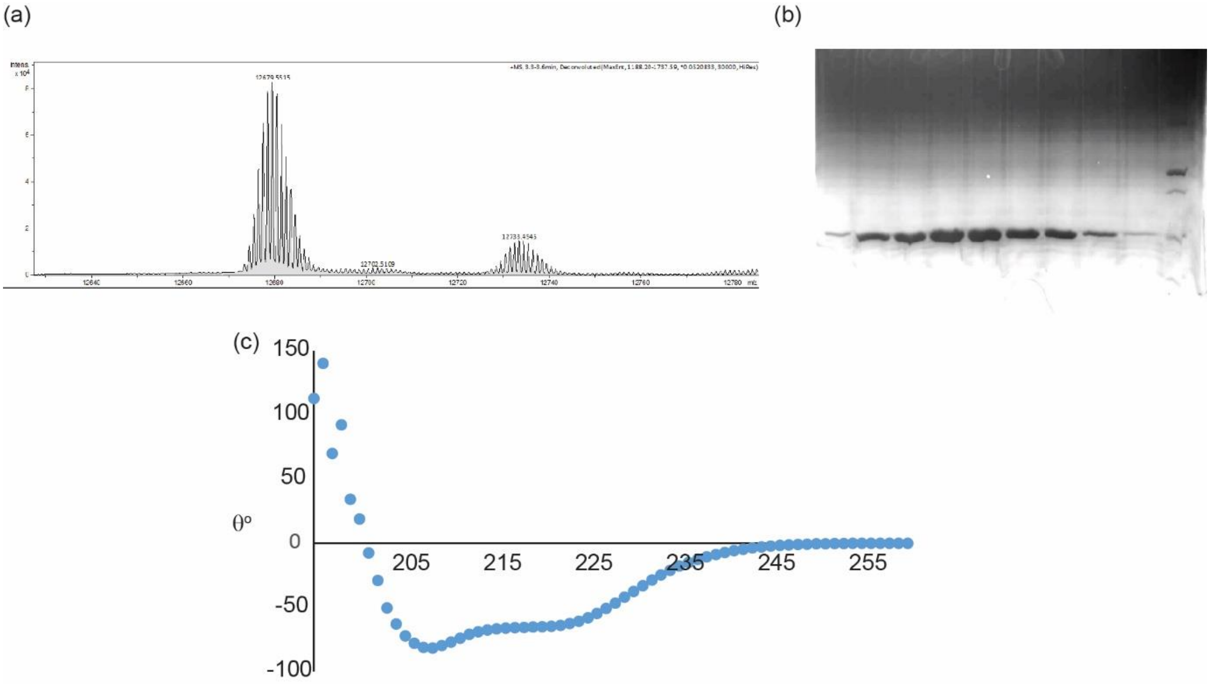

Characterization of hDM2 (a) deconvoluted mass spectra (b) SDS-PAGE of newly prepared protein after size-exclusion chromatography and (c) CD of purified protein.

9. Small Molecule Spectra

Ethyl (3a*S*,4*R*,9b*R*)-8-sulfamoyl-3H,3aH,4H,5H,9bH-cyclopenta[c]quinoline-4-carboxylate

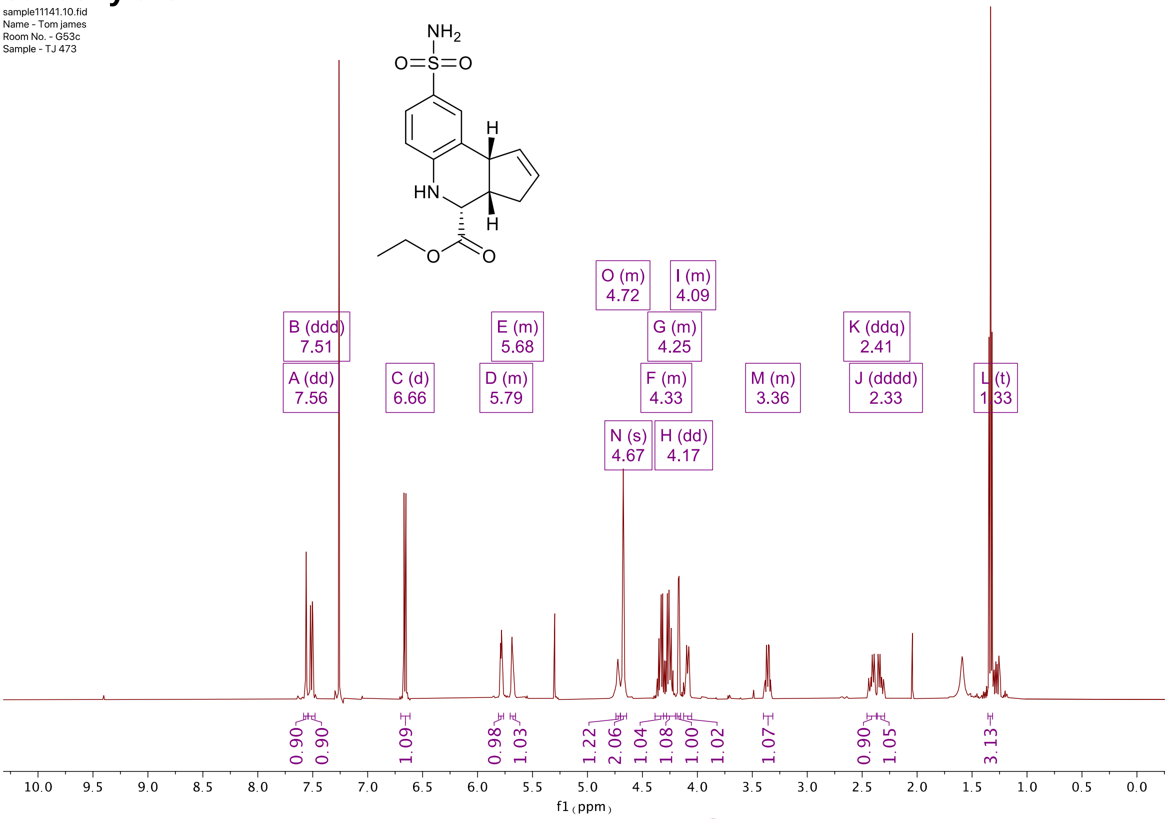

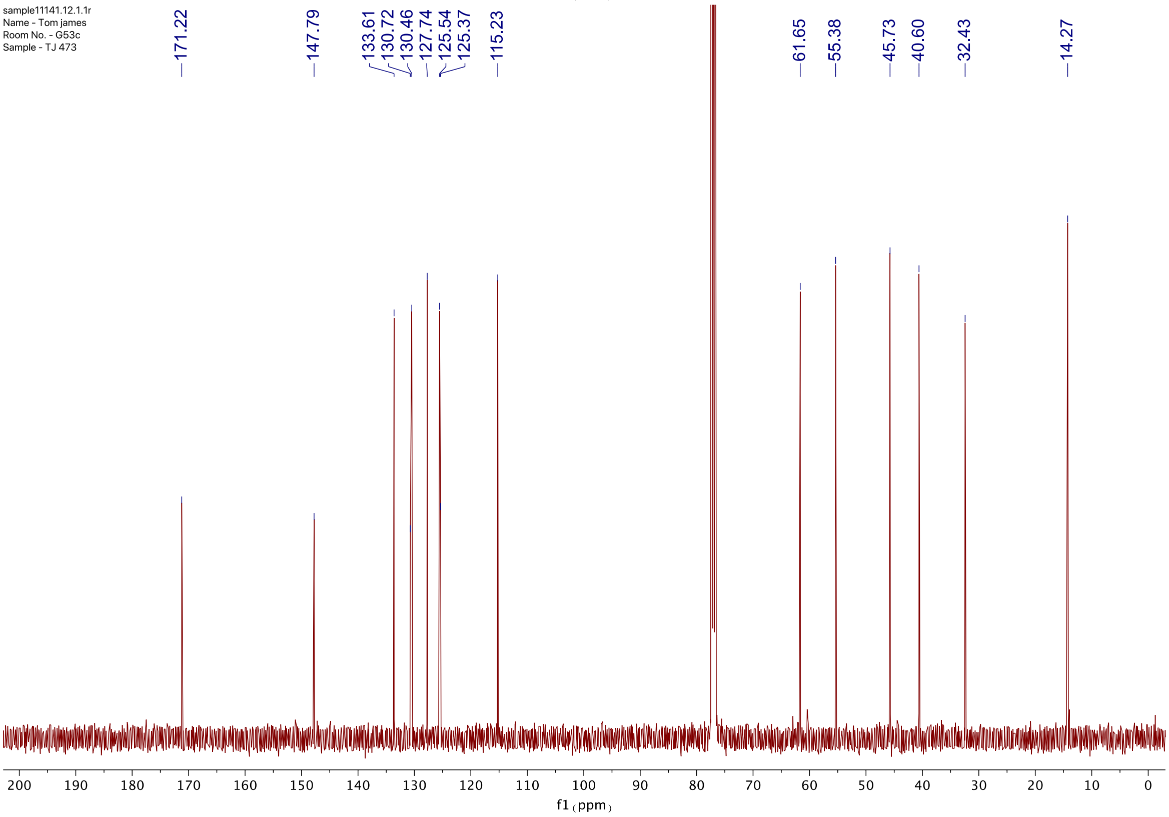

(3a*S*,4*R*,9b*R*)-8-Sulfamoyl-3H,3aH,4H,5H,9bH-cyclopenta[c]quinoline-4-carboxylic acid

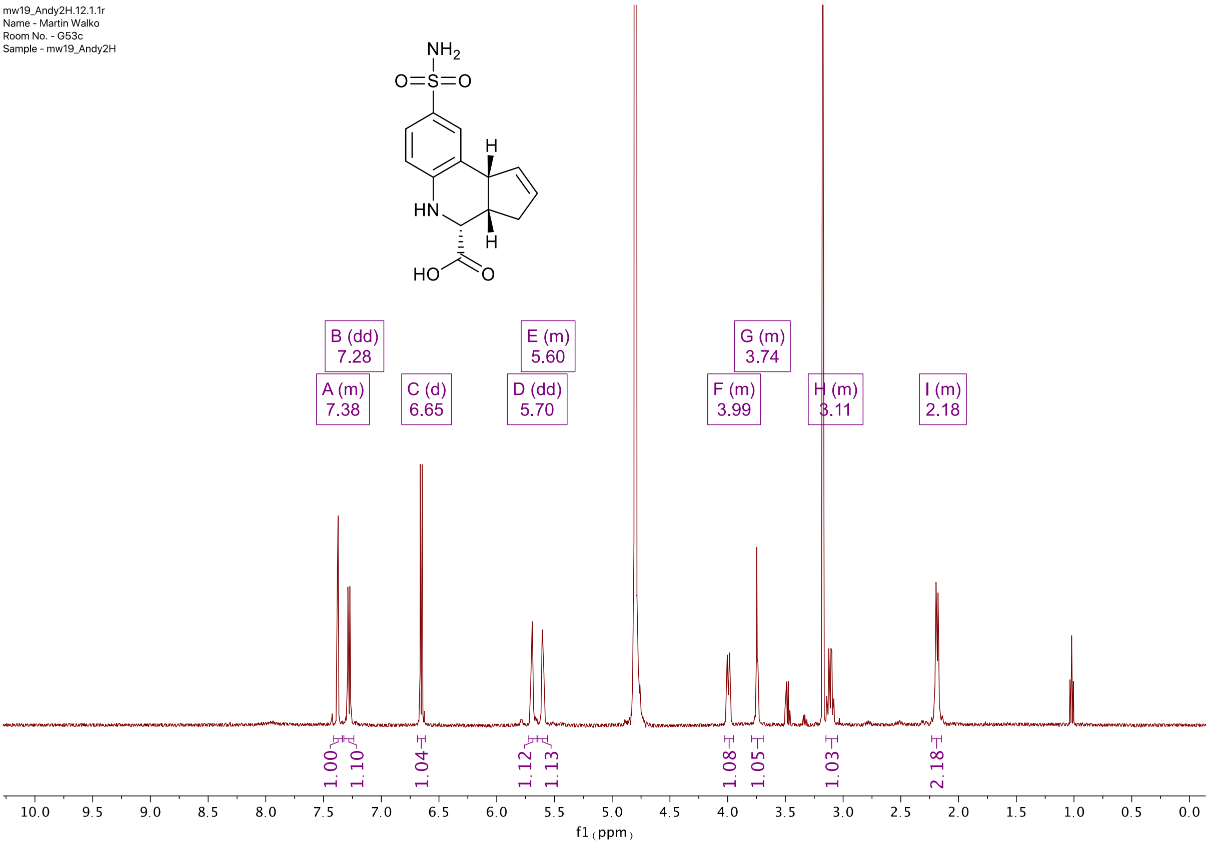

5-Chloro-3-[(5-chlorothiophen-2-yl)(4-methylbenzenesulfonyl)methyl]-1H-indole

Name - TOm james
Room No. - G53c
Sample - TJ382 ppt

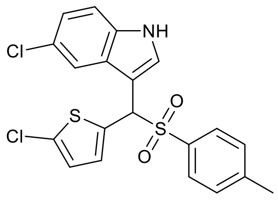

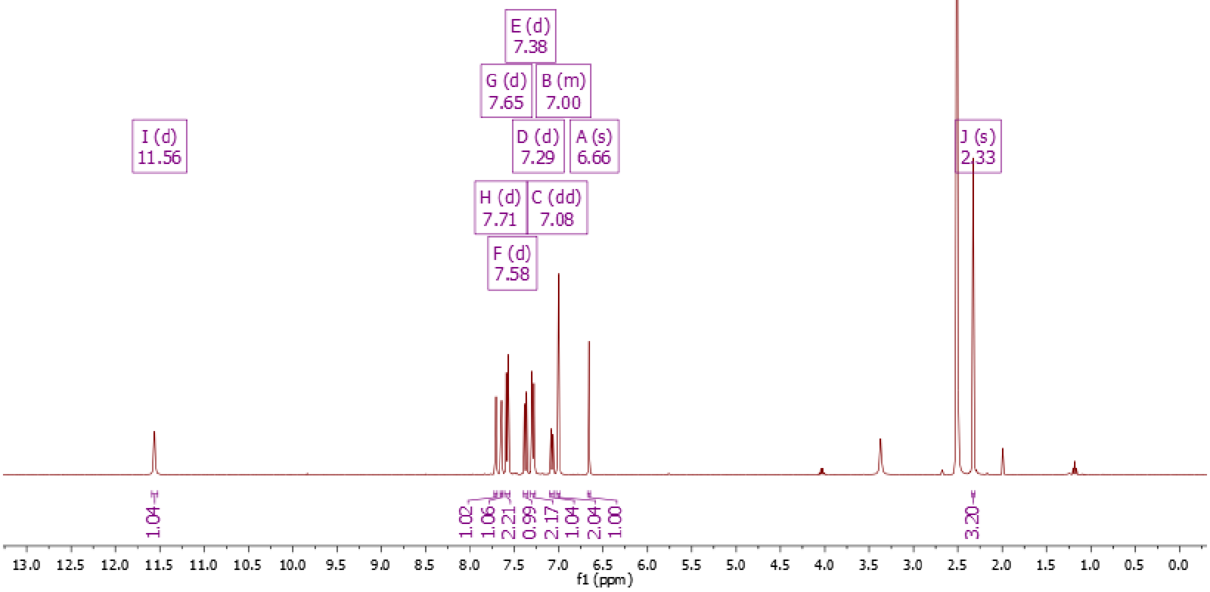

Name - TOm james
Room No. - G53c
Sample - TJ382 ppt

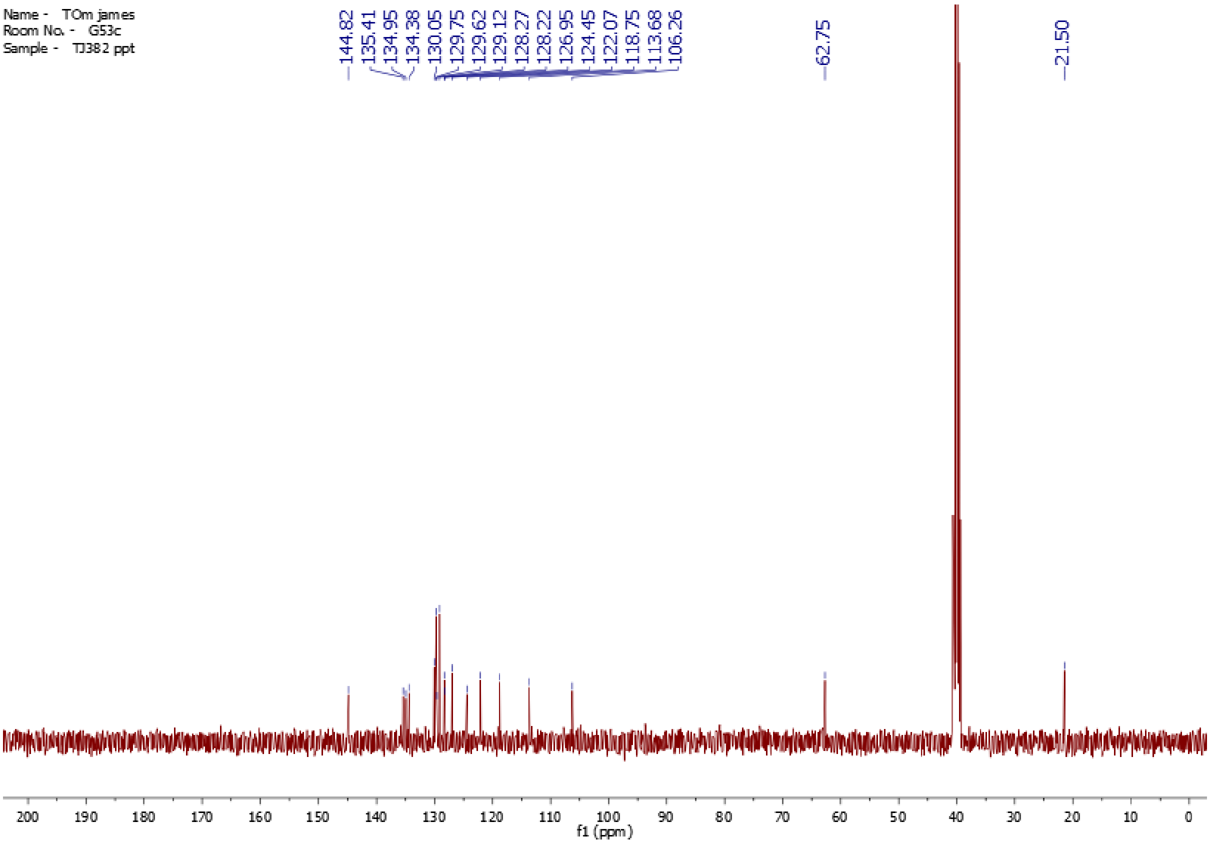

Methyl 3-(5-chloro-1H-indol-3-yl)-3-(5-chlorothiophen-2-yl)propanoate

Name - Tom James
Room No. - G53c
Sample - TJ 409 F1

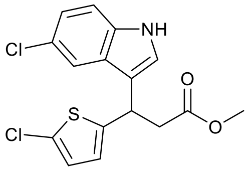

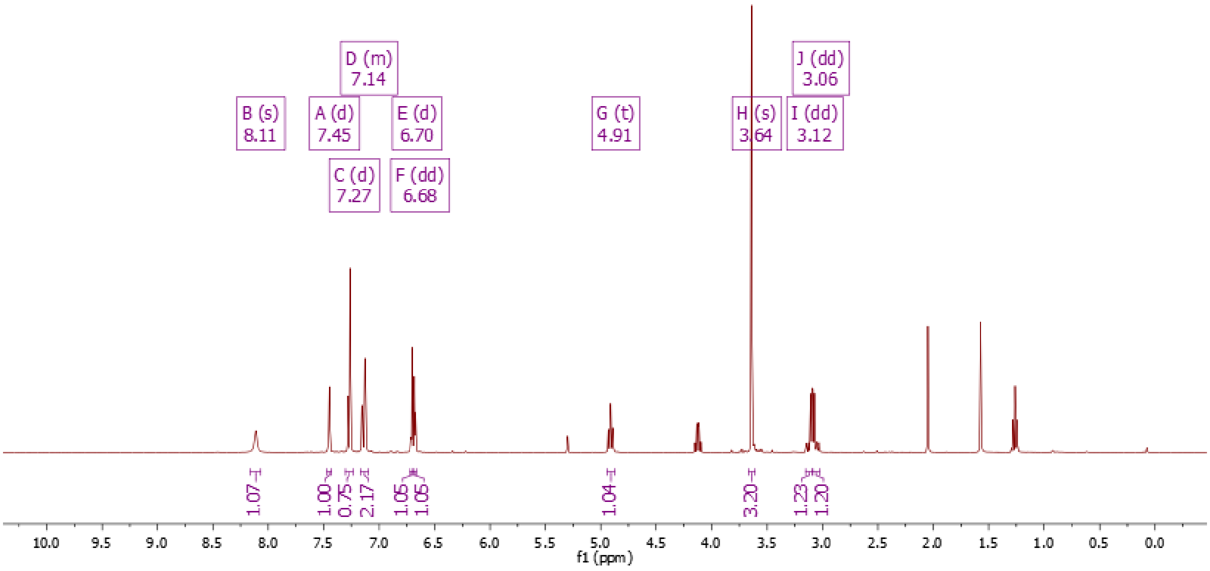

Name - TOm James
Room No. - G53c
Sample - TJ 383 F1

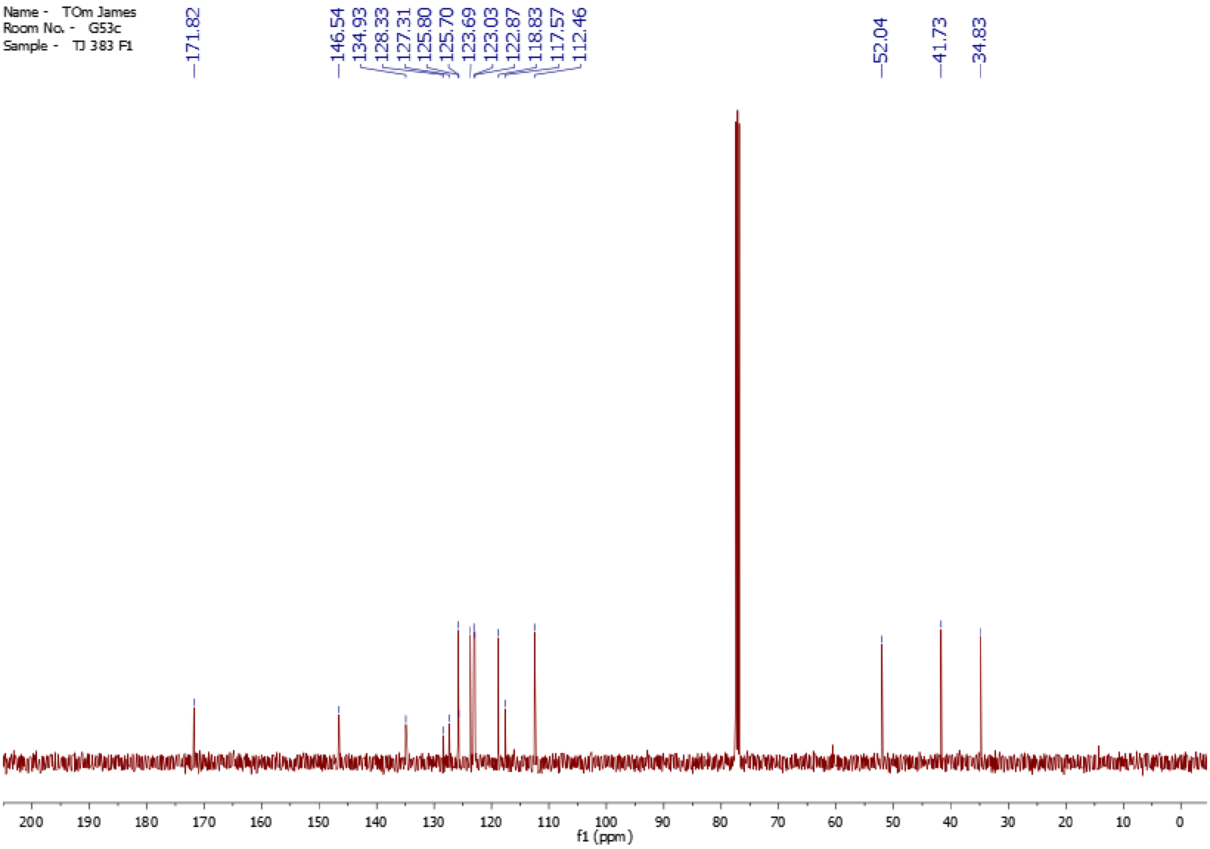

3-(5-Chloro-1H-indol-3-yl)-3-(5-chlorothiophen-2-yl)propanoic acid

Name - Tom James
Room No. - G53c
Sample - TJ 411 WU

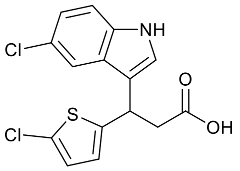

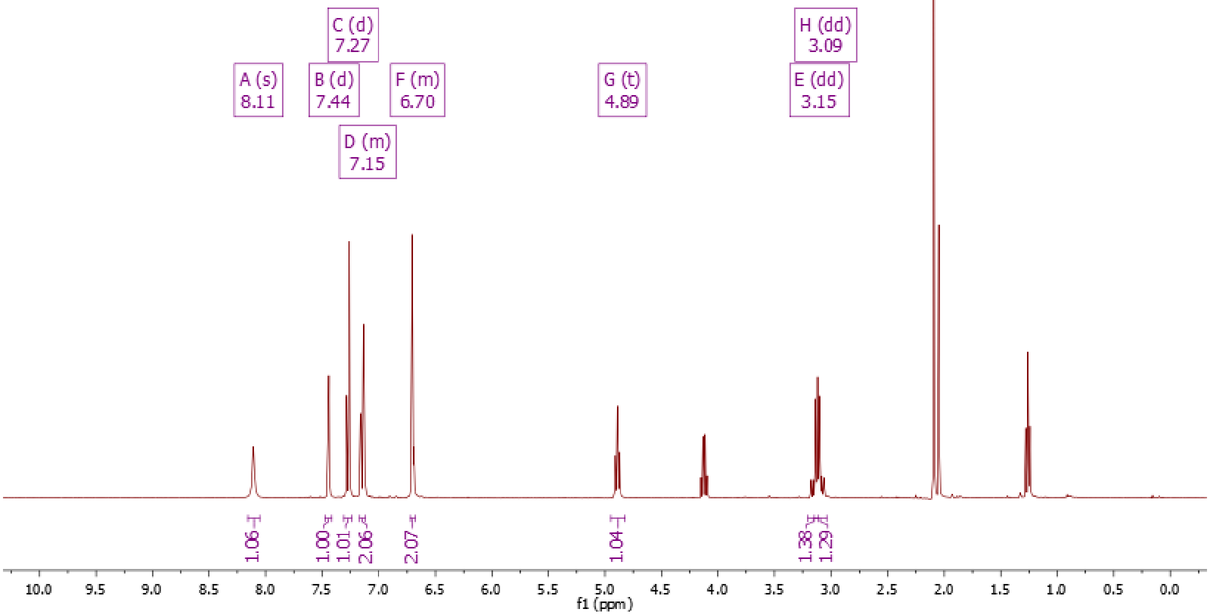

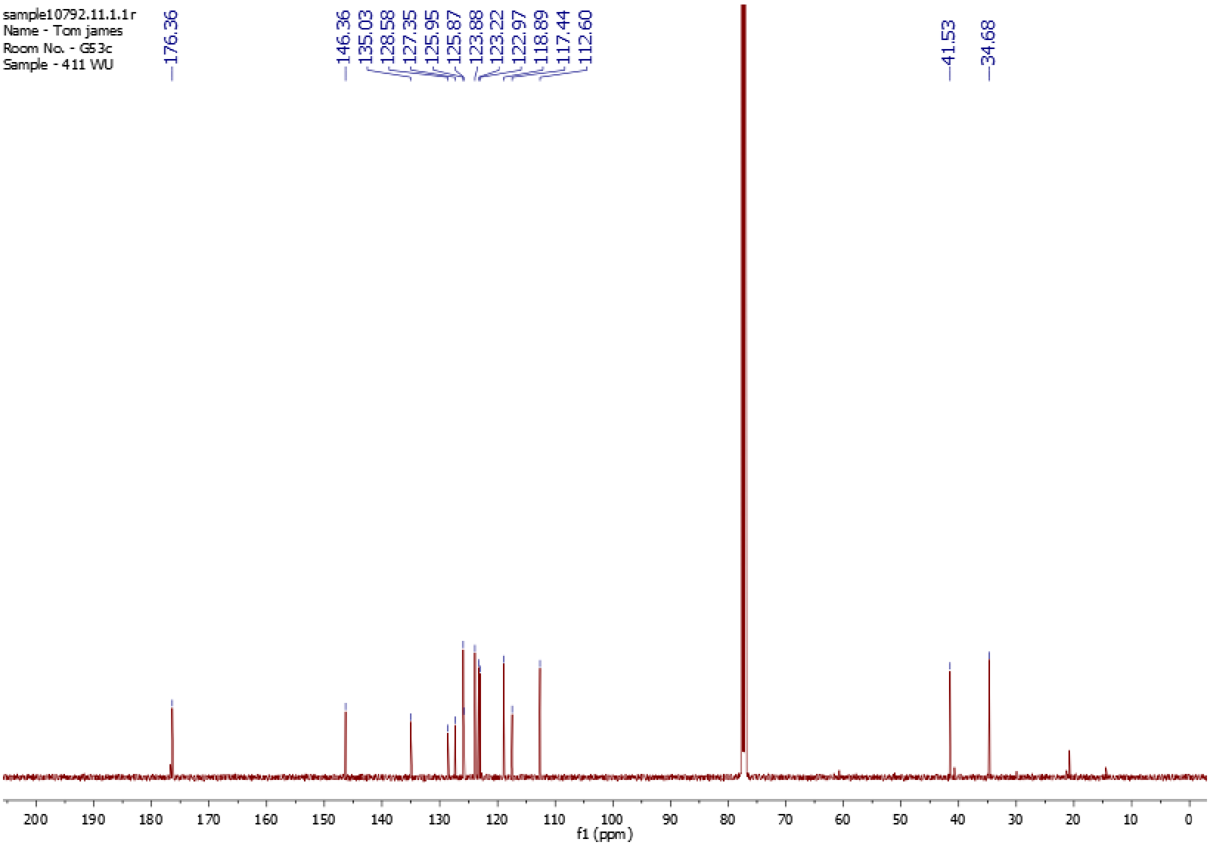

3-(5-Chloro-1H-indol-3-yl)-3-(5-chlorothiophen-2-yl)-*N*-[2-(pyridin-2-yl)ethyl] propanamide

sample10780.10.1.1r
Name - Tom James
Room No. - G53c
Sample - TJ 385

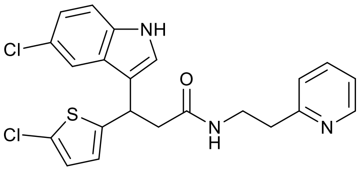

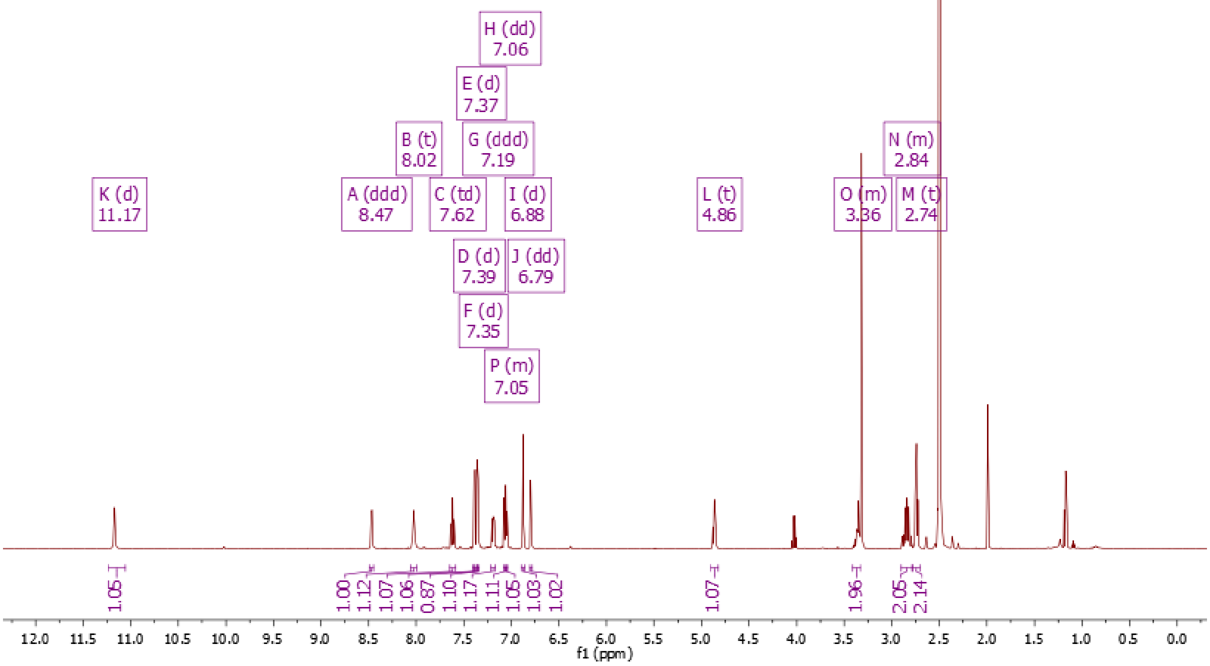

sample10780.11.1.1r
Name - Tom James
Room No. - G53c
Sample - TJ 385

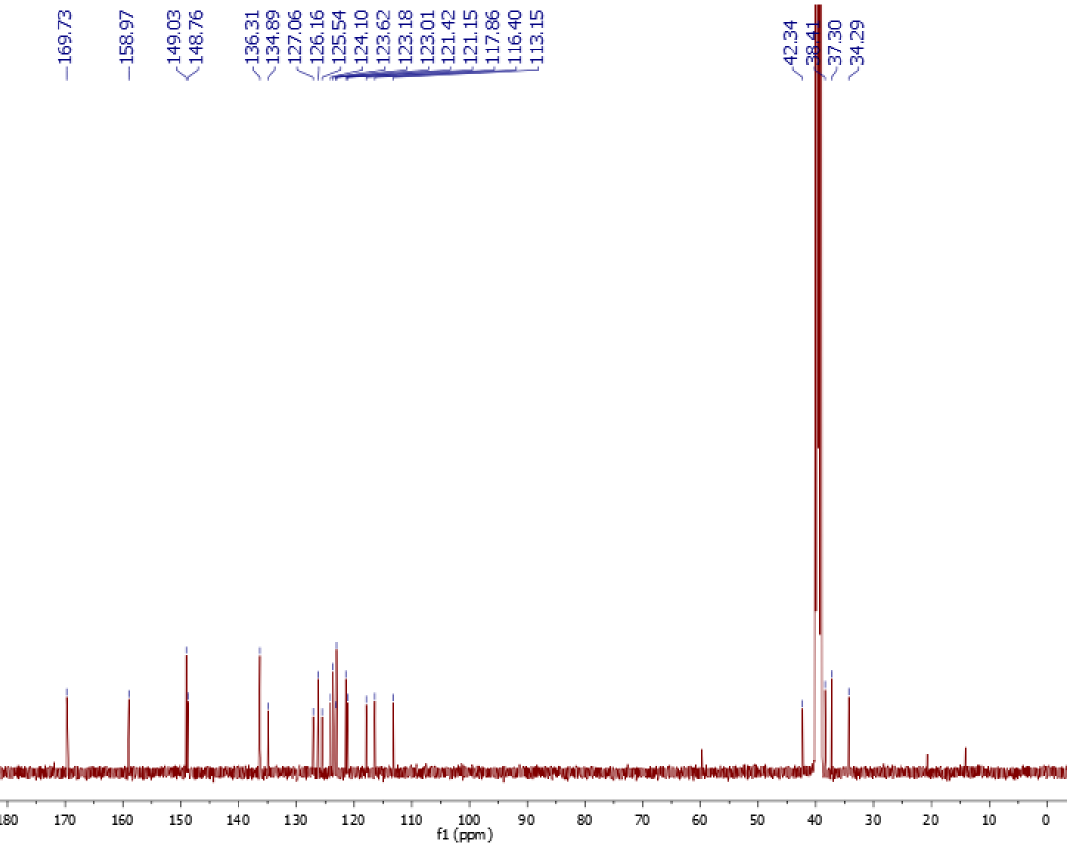

3-(5-Chloro-1H-indol-3-yl)-3-(5-chlorothiophen-2-yl)-*N*-(2-phenylethyl) propanamide

Name - Tom james
Room No. - G53c
Sample - TJ 423 B F1

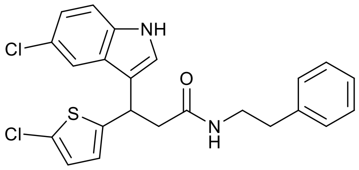

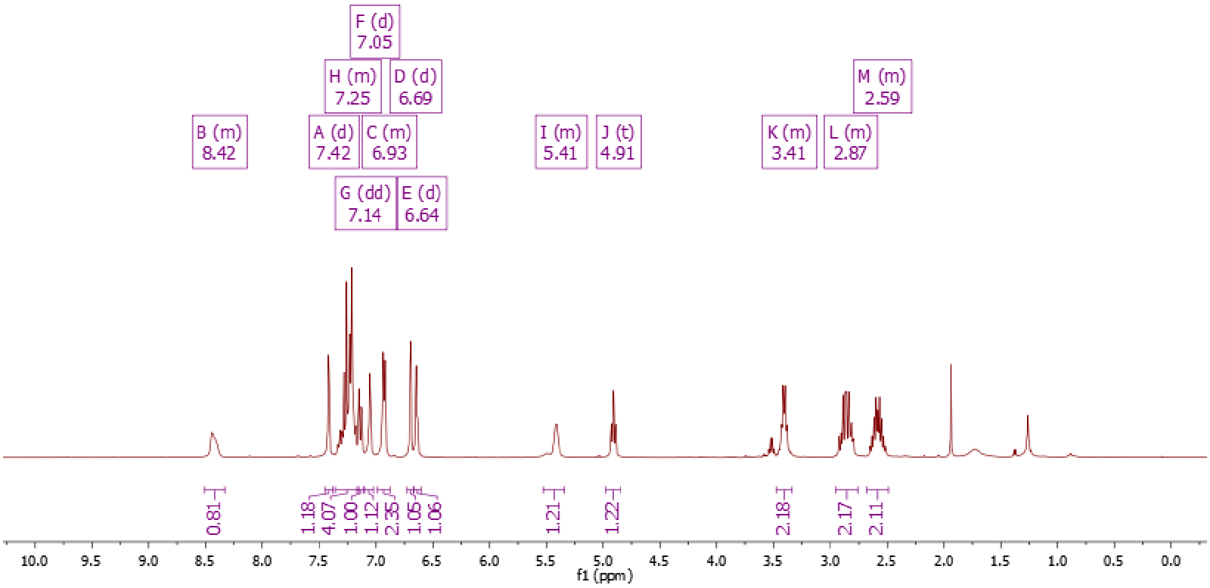

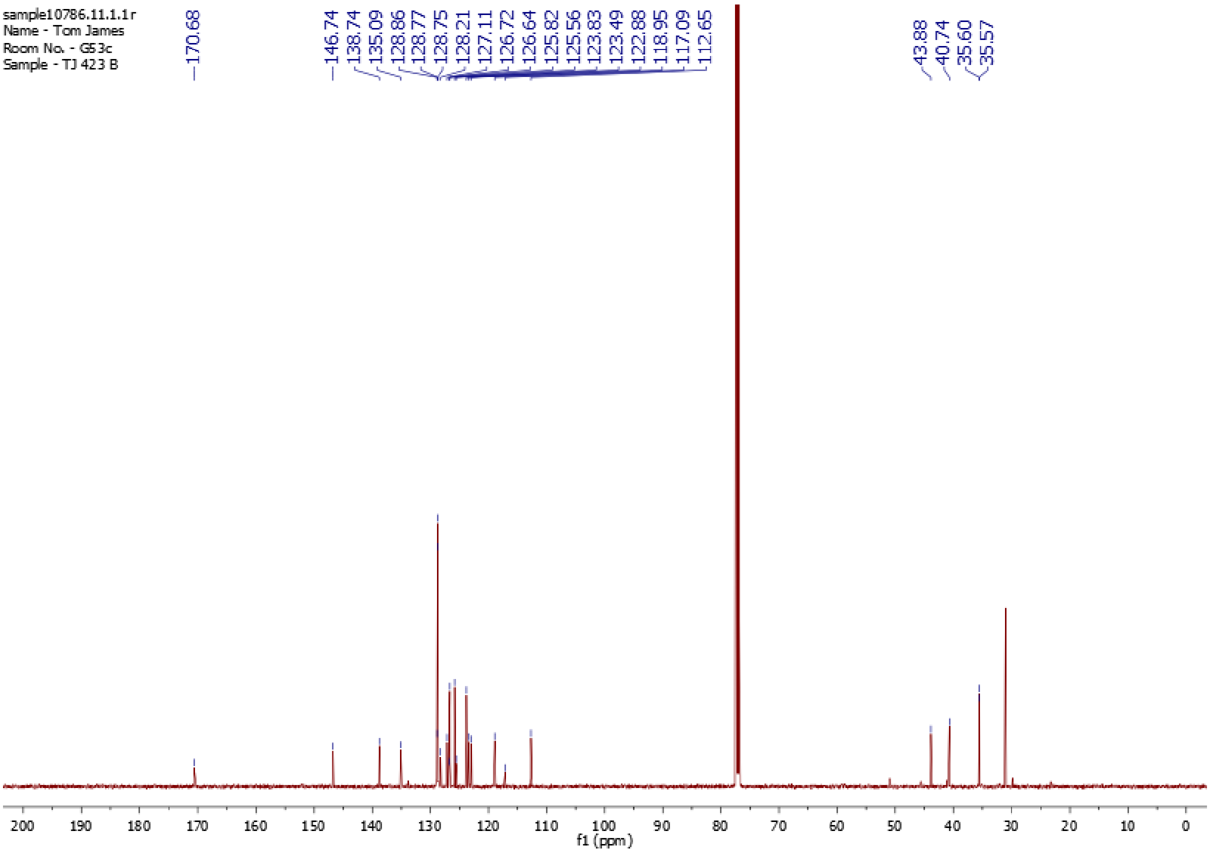

N-Benzyl-3-(5-chloro-1H-indol-3-yl)-3-(5-chlorothiophen-2-yl)propanamide

sample17285.10.1.1r
Name - Sergio
Room No. - G53c
Sample - SC144-1

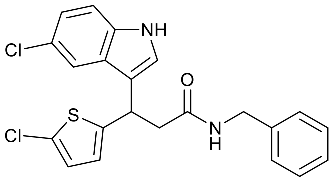

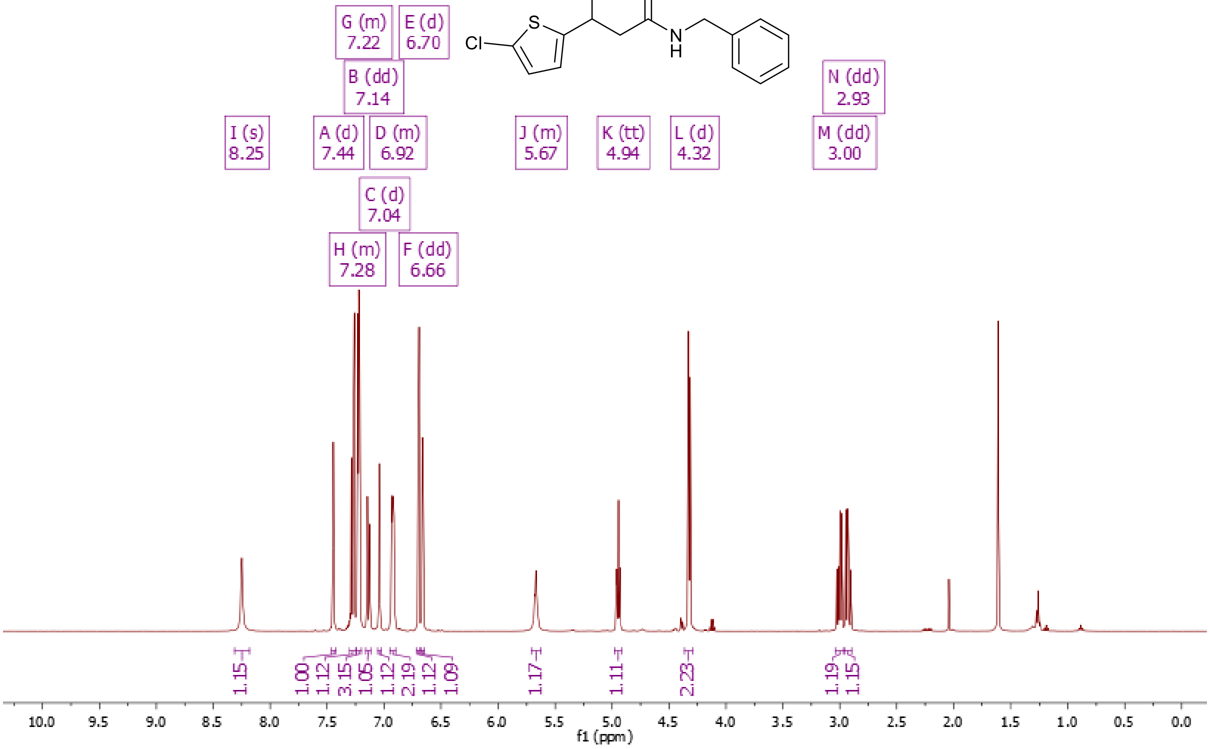

sample17289.10.1.1r
Name - Sergio
Room No. - G53c
Sample - SC144-1

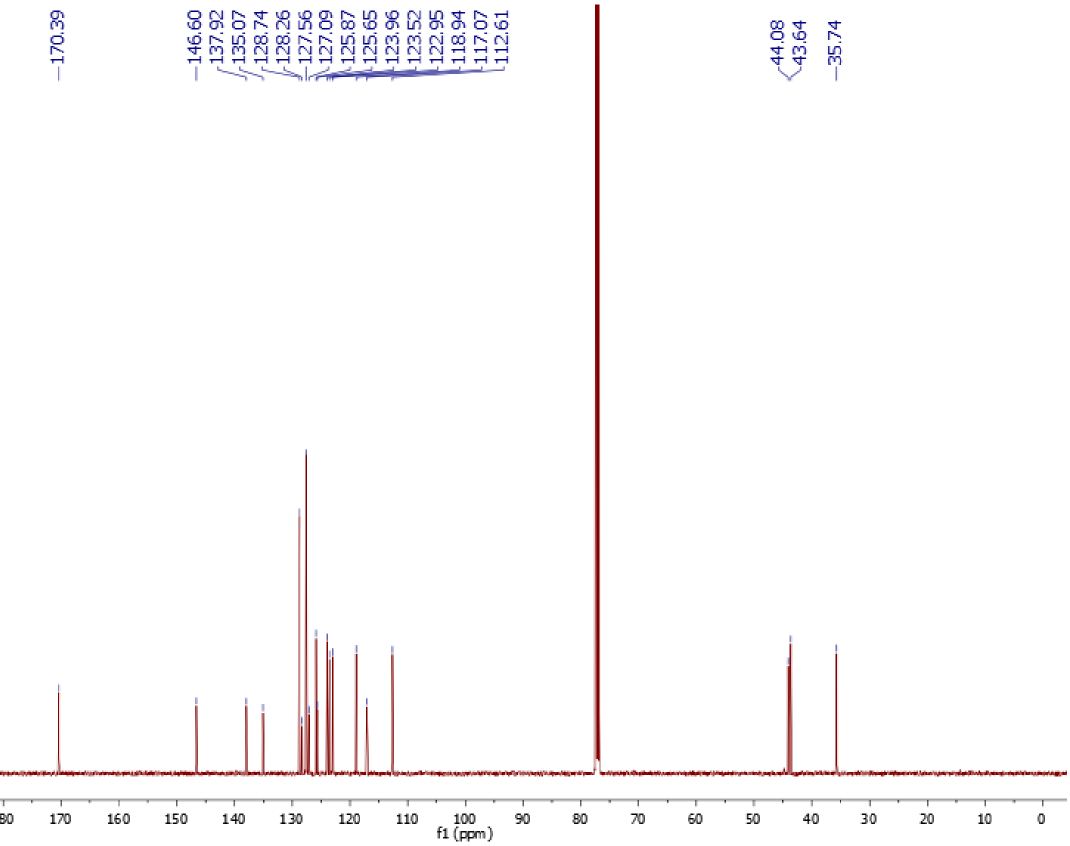

3-(5-Chloro-1H-indol-3-yl)-3-(5-chlorothiophen-2-yl)-*N*-(2-methoxyethyl) propanamide

Name - Tom James
Room No. - G53c
Sample - TJ 423 C F1

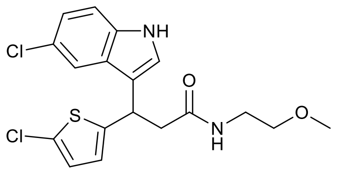

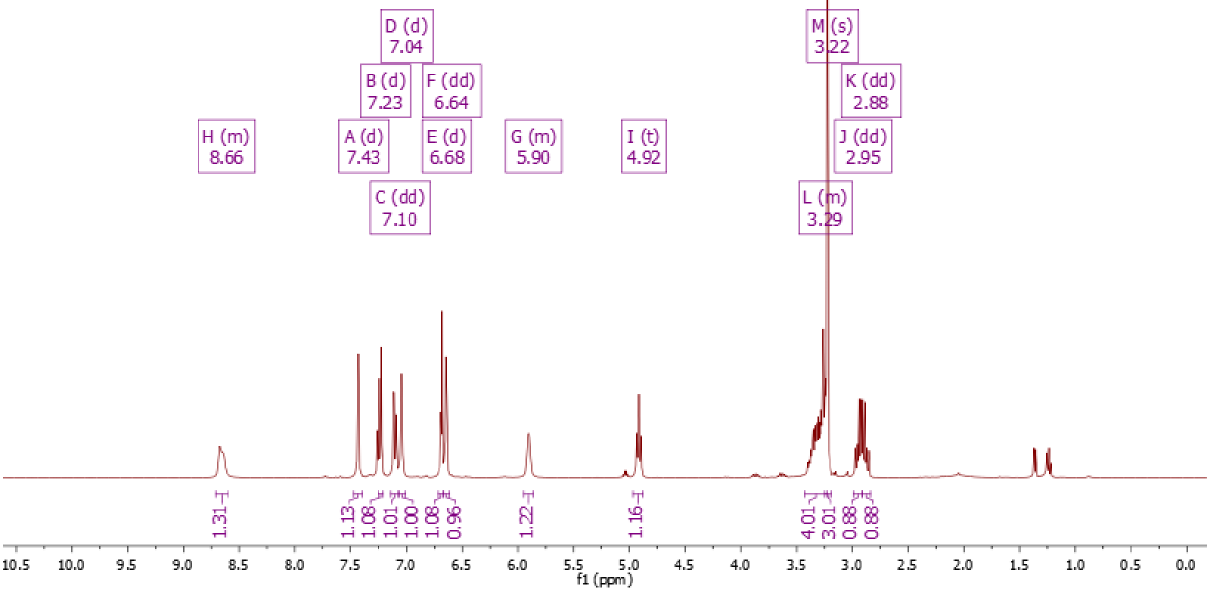

sample10790.11.1.1r
Name - Tom James
Room No. - G53c
Sample - TJ 423 C

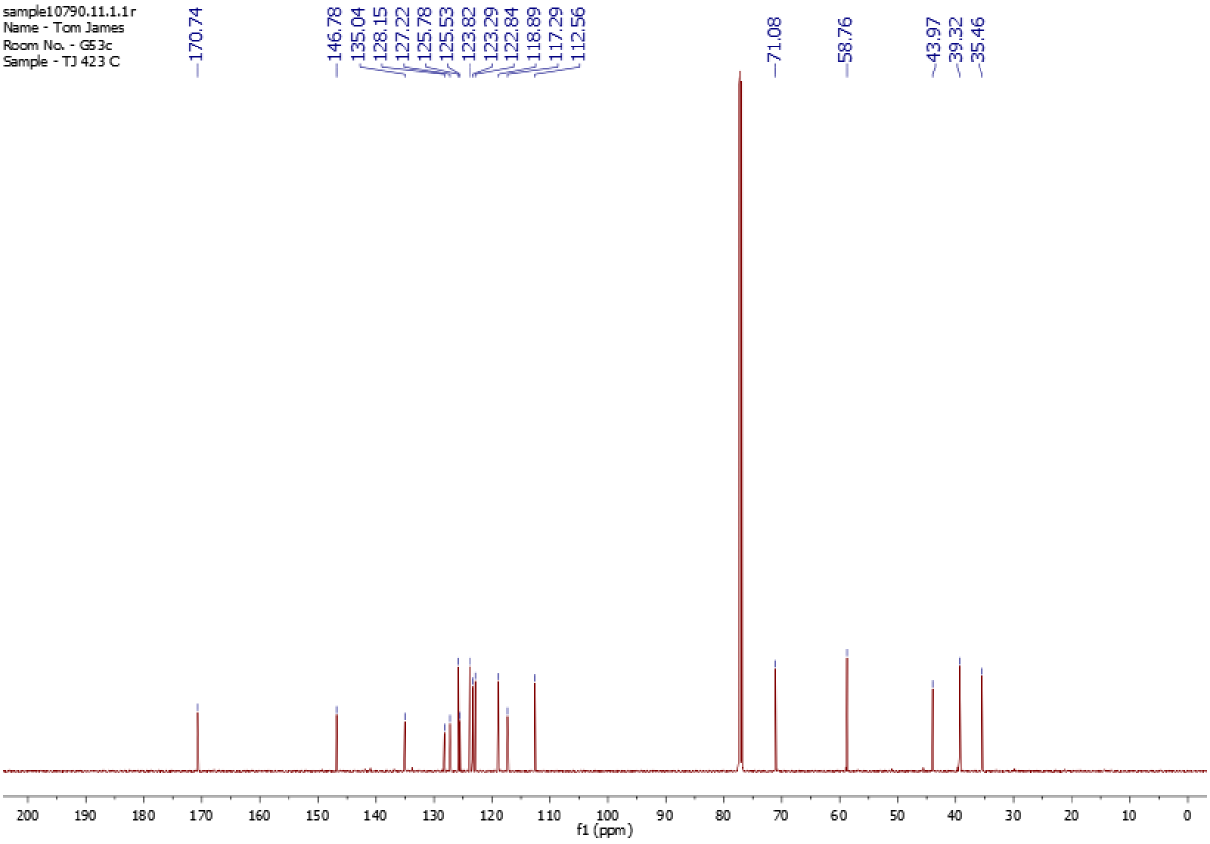

6-Chloro-3-[(5-chlorothiophen-2-yl)(4-methylbenzenesulfonyl)methyl]-1H-indole

Name - Tom James
Room No. - G53c
Sample - TJ372 ppt

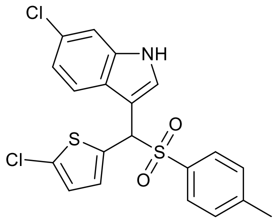

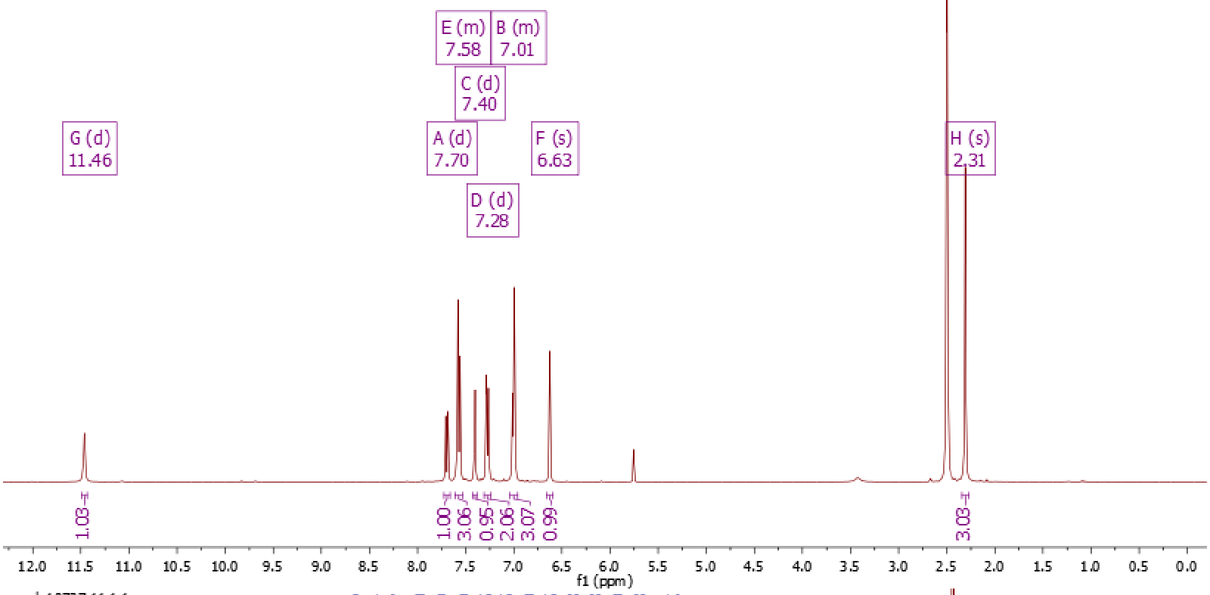

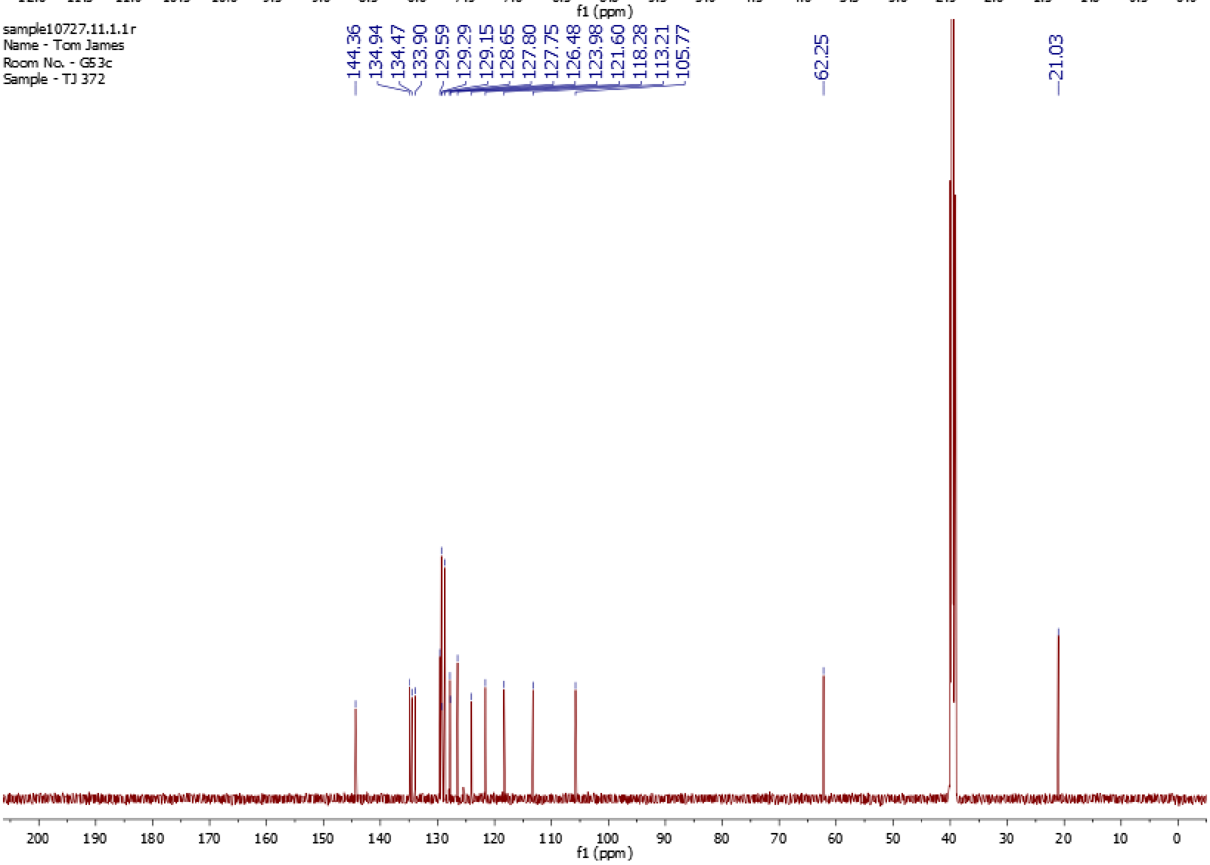

Methyl 3-(6-chloro-1H-indol-3-yl)-3-(5-chlorothiophen-2-yl)propanoate

Name - Tom james
Room No. - G53c
Sample - TJ 410 f1

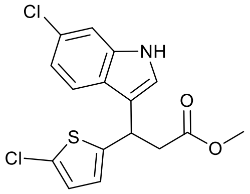

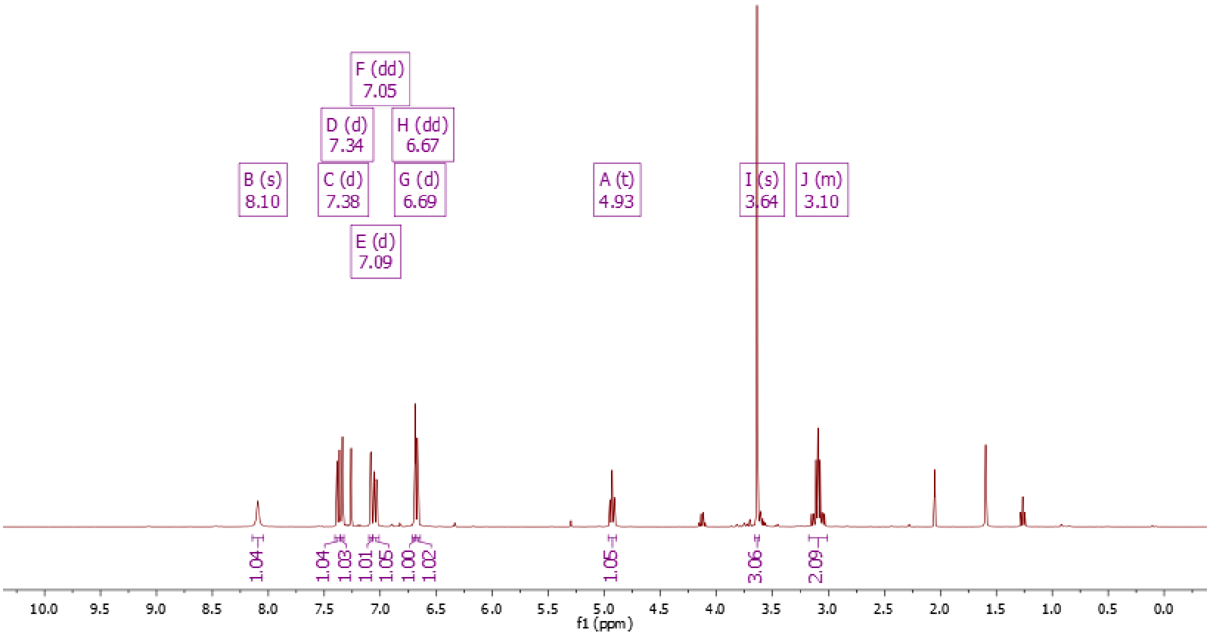

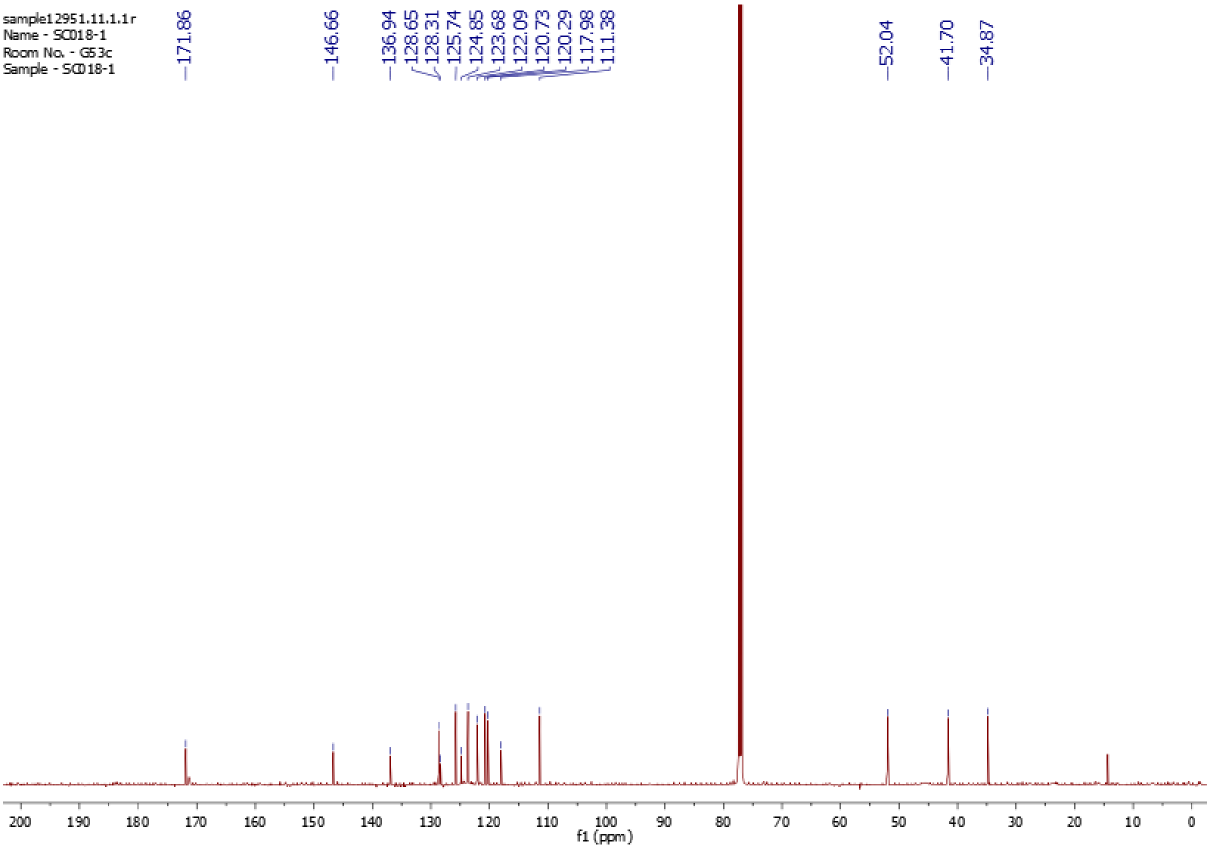

3-(6-Chloro-1H-indol-3-yl)-3-(5-chlorothiophen-2-yl)propanoic acid

sample21871.10.1.1r
Name - Sergio1
Room No. - G53d
Sample - SC acid

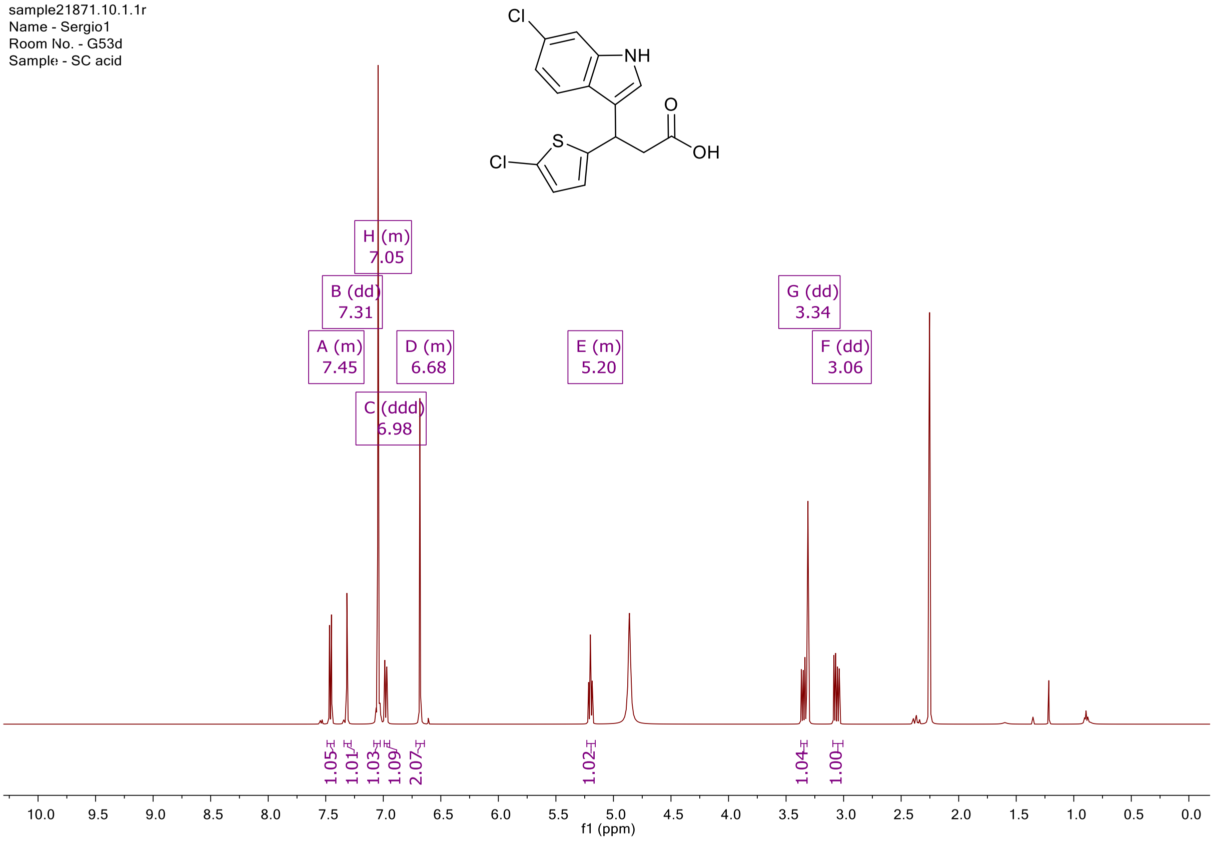

sample21788.11.1.1r
Name - Sergio
Room No. - G53d
Sample - SC220-1

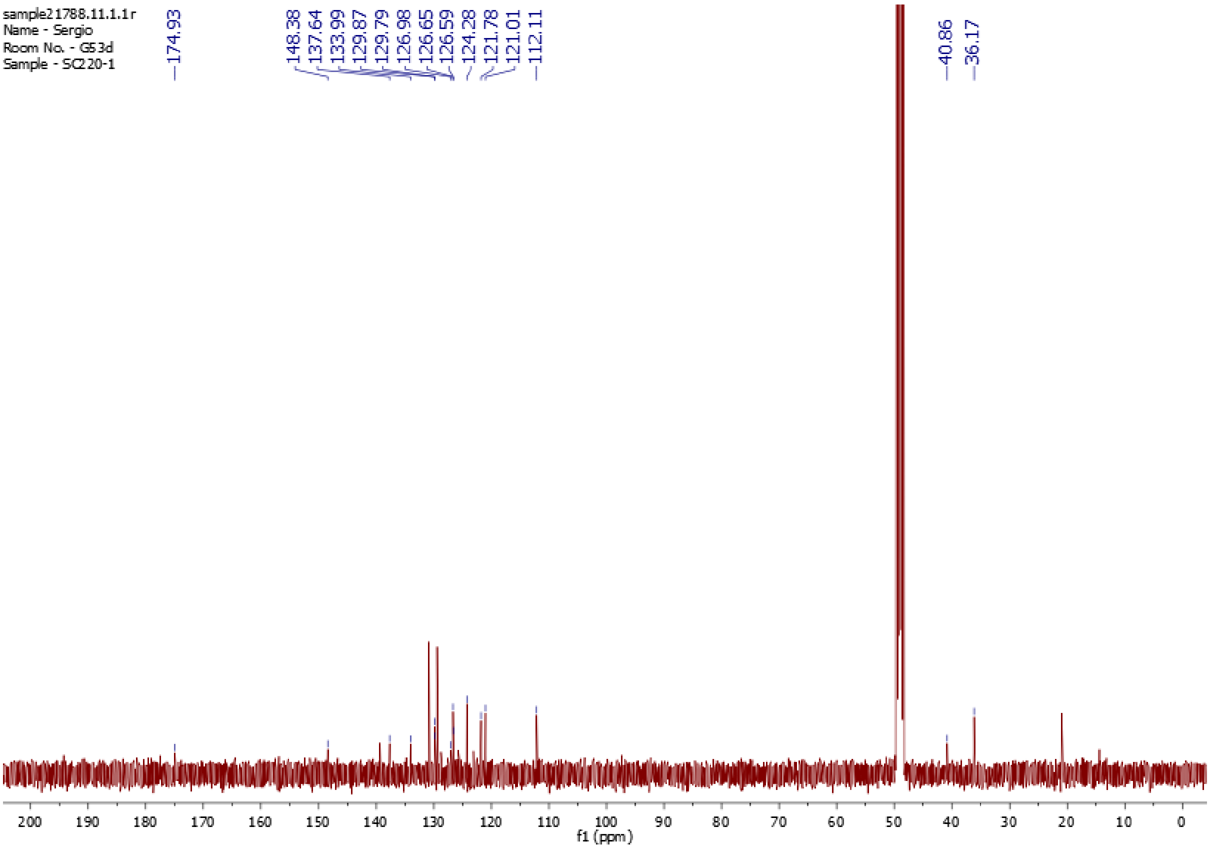

3-(6-Chloro-1H-indol-3-yl)-3-(5-chlorothiophen-2-yl)-*N*-[2-(pyridin-2-yl) ethyl] propanamide

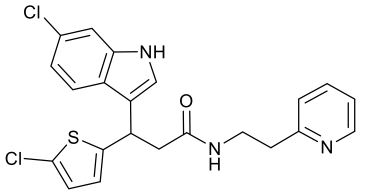

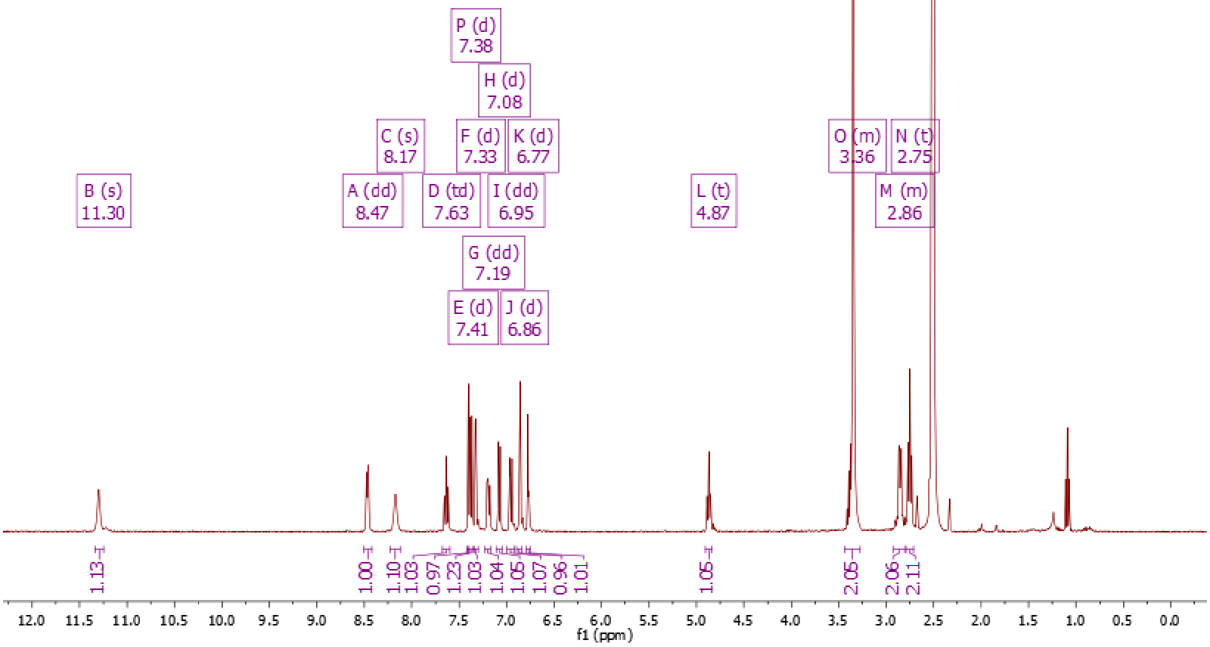

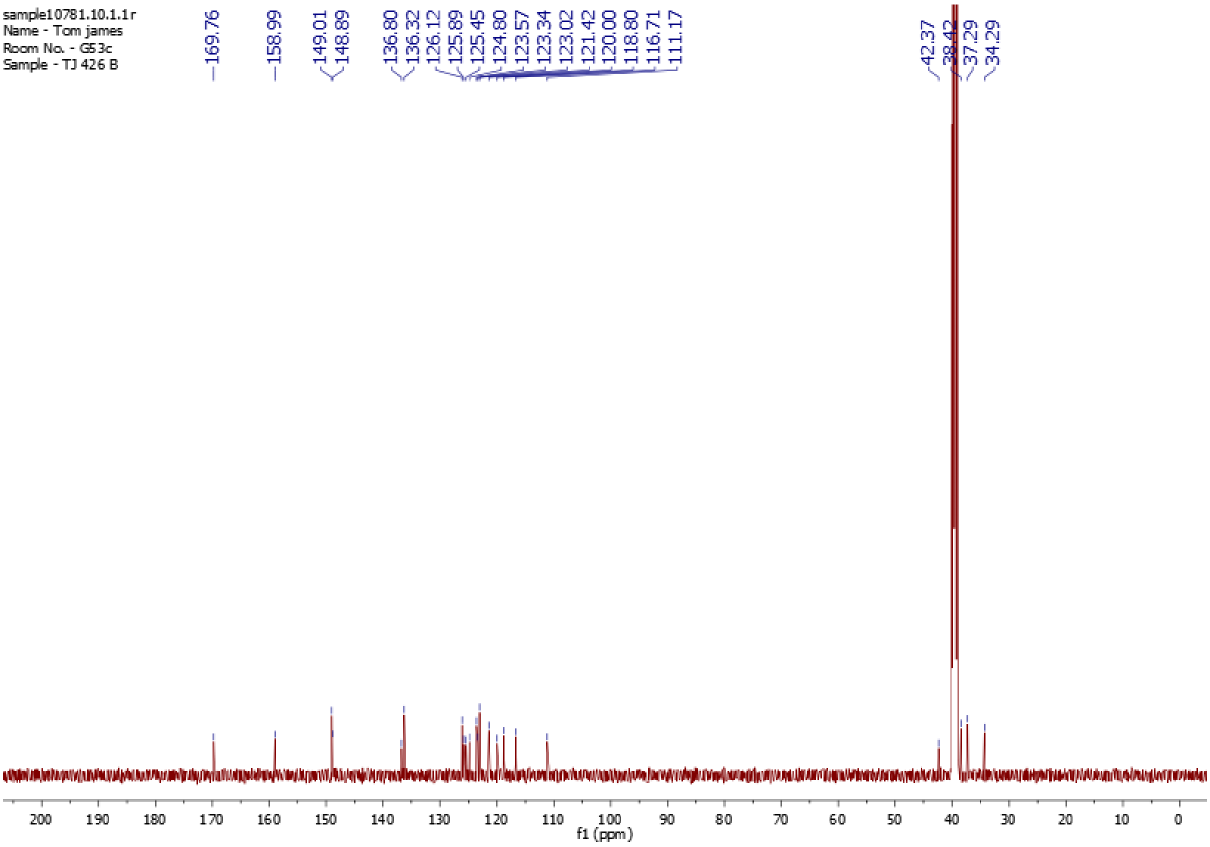

3-(6-Chloro-1H-indol-3-yl)-3-(5-chlorothiophen-2-yl)-*N*-(2-phenylethyl) propanamide

Name - TOm James
Room No. - G53c
Sample - TJ424 B F1

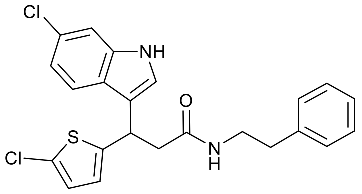

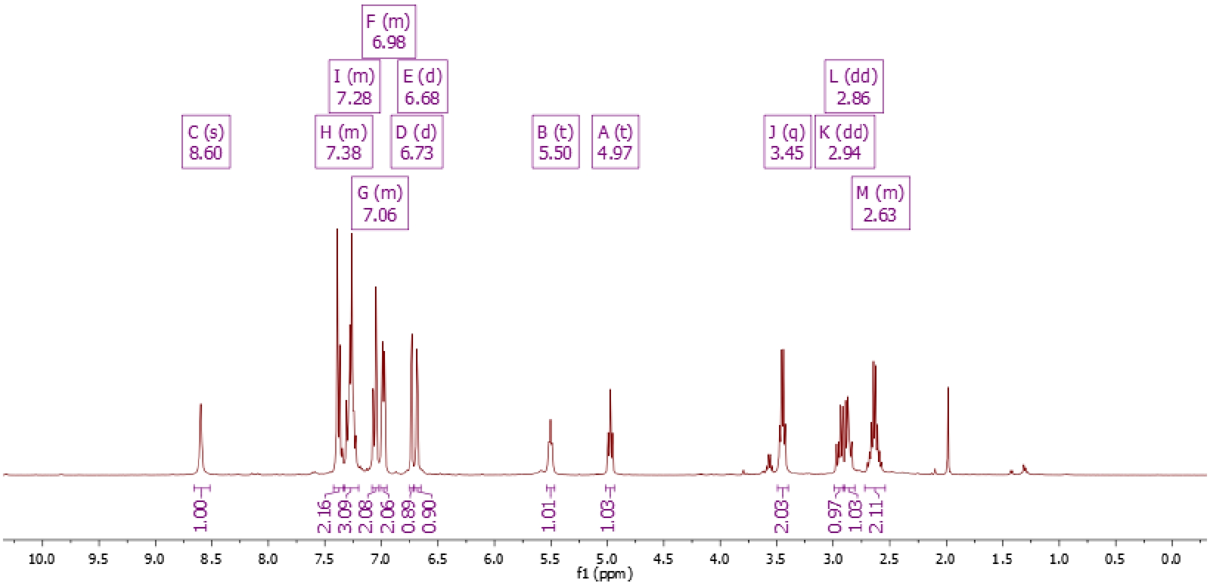

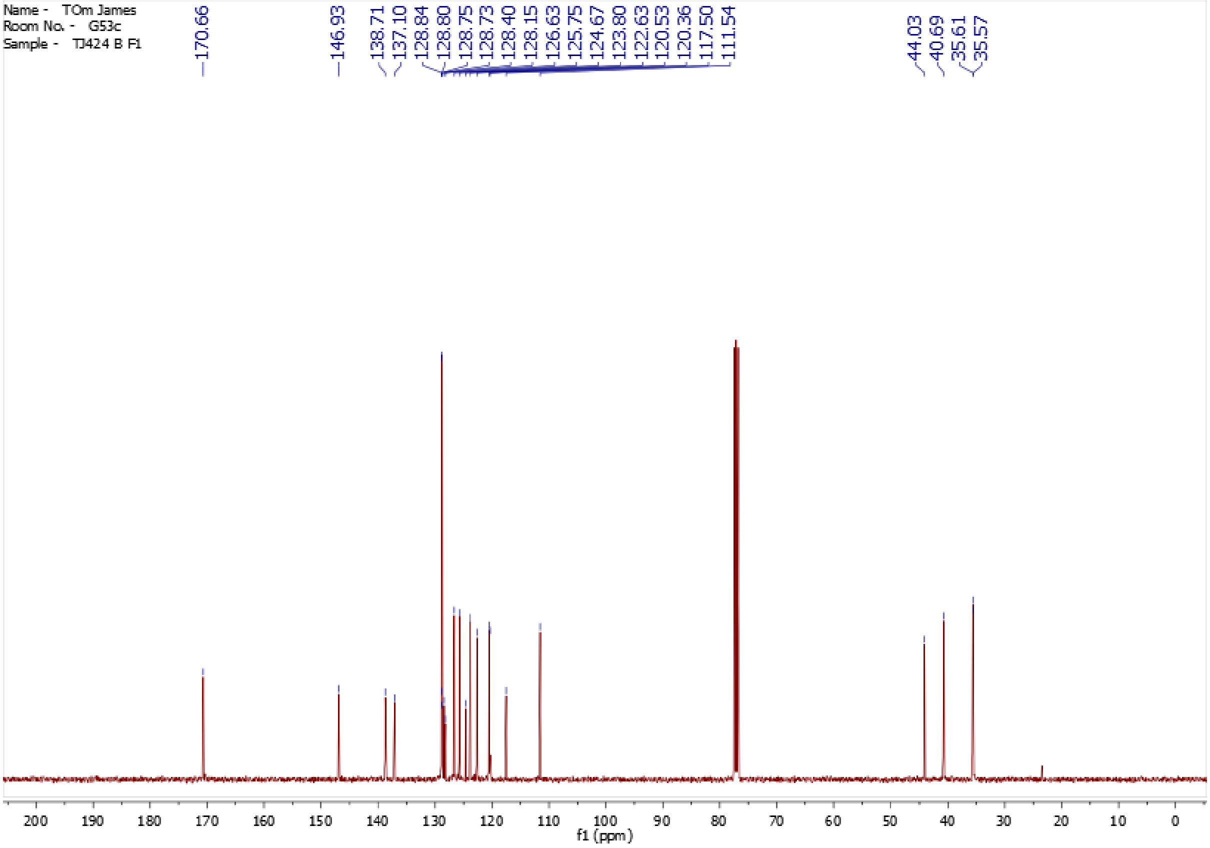

3-(6-Chloro-1H-indol-3-yl)-3-(5-chlorothiophen-2-yl)-*N*-(2-methoxyethyl) propanamide

Name - TOm James
Room No. - G53c
Sample - TJ424 C F1

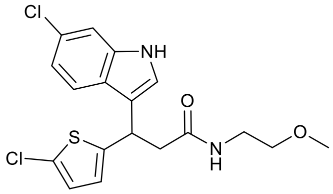

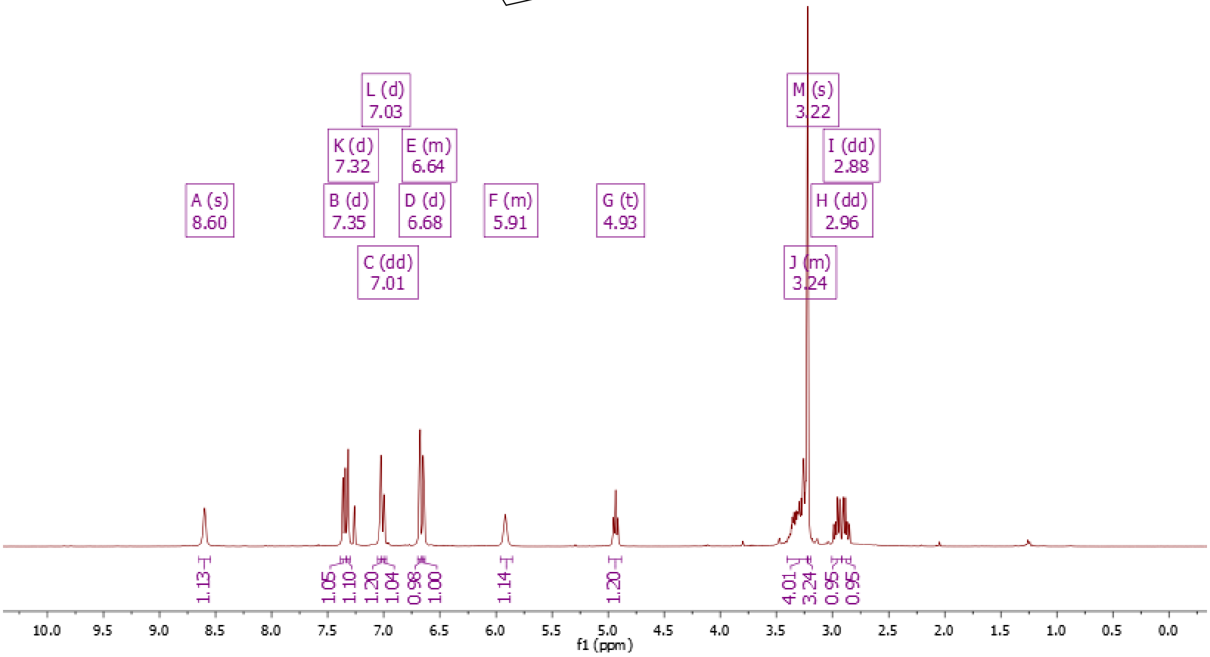

Name - TOm James
Room No. - G53c
Sample - TJ424 C F1

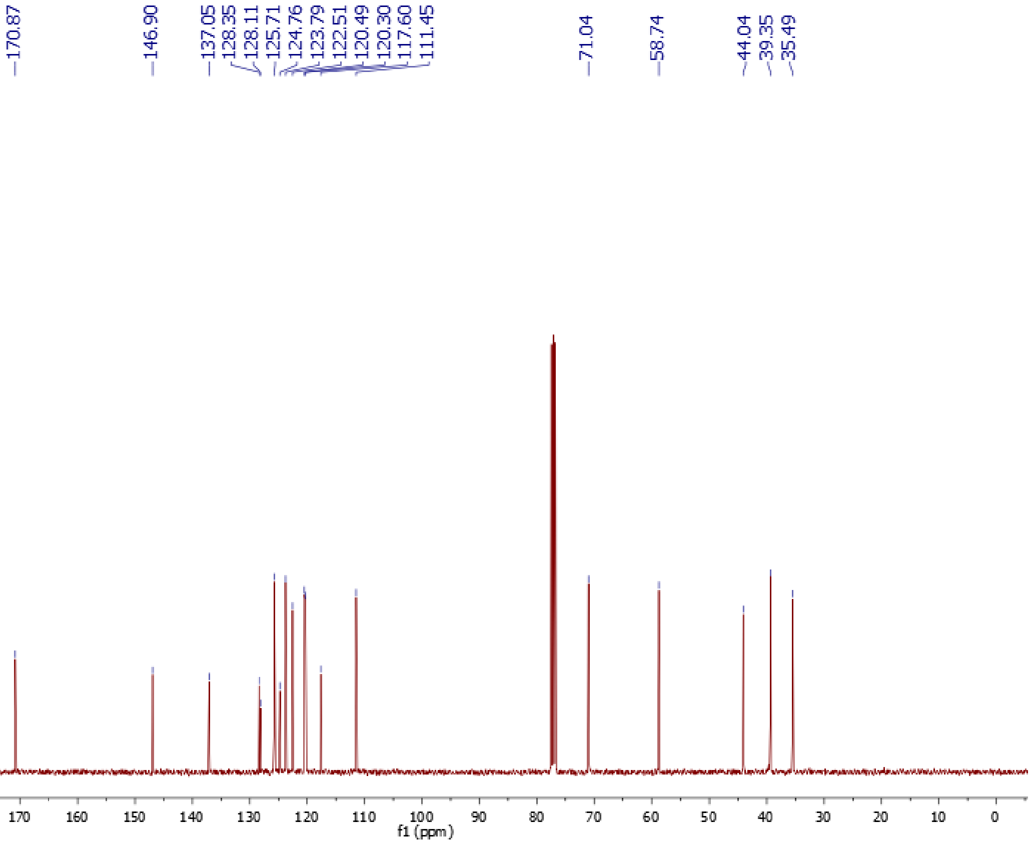

5-Chloro-3-[(4-methylbenzenesulfonyl)(phenyl)methyl]-1H-indole

Name - Tom James
Room No. - G53c
Sample - TJ 462

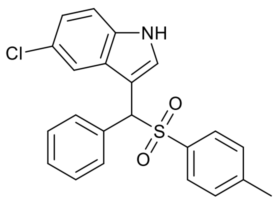

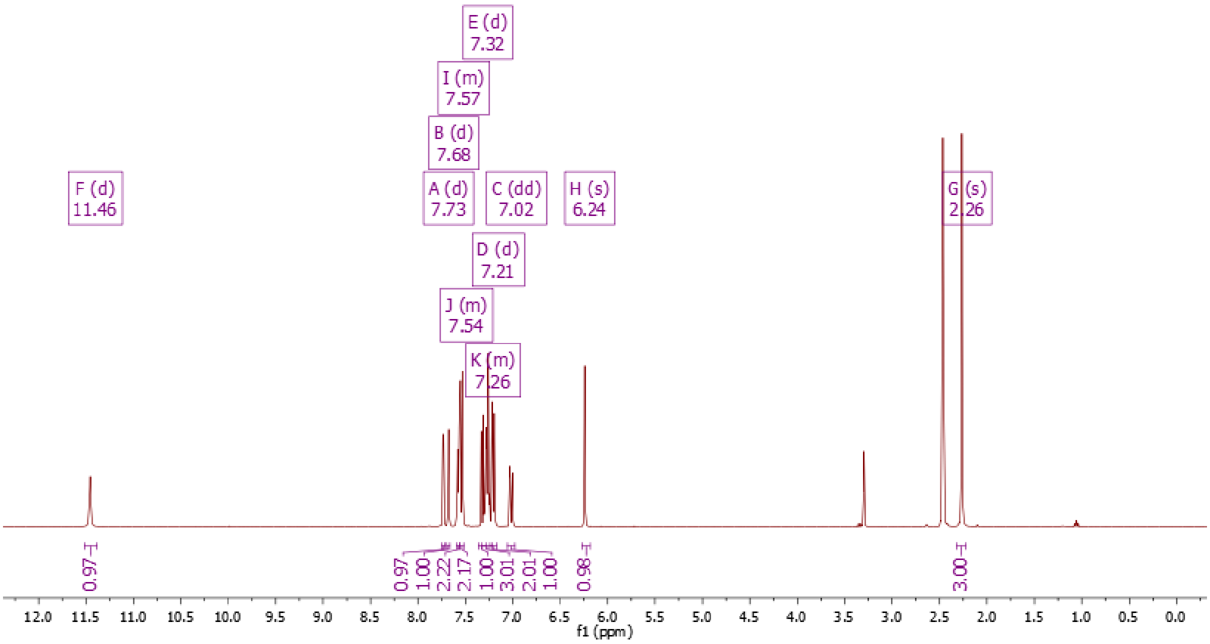

Name - Tom James
Room No. - G53c
Sample - TJ 462

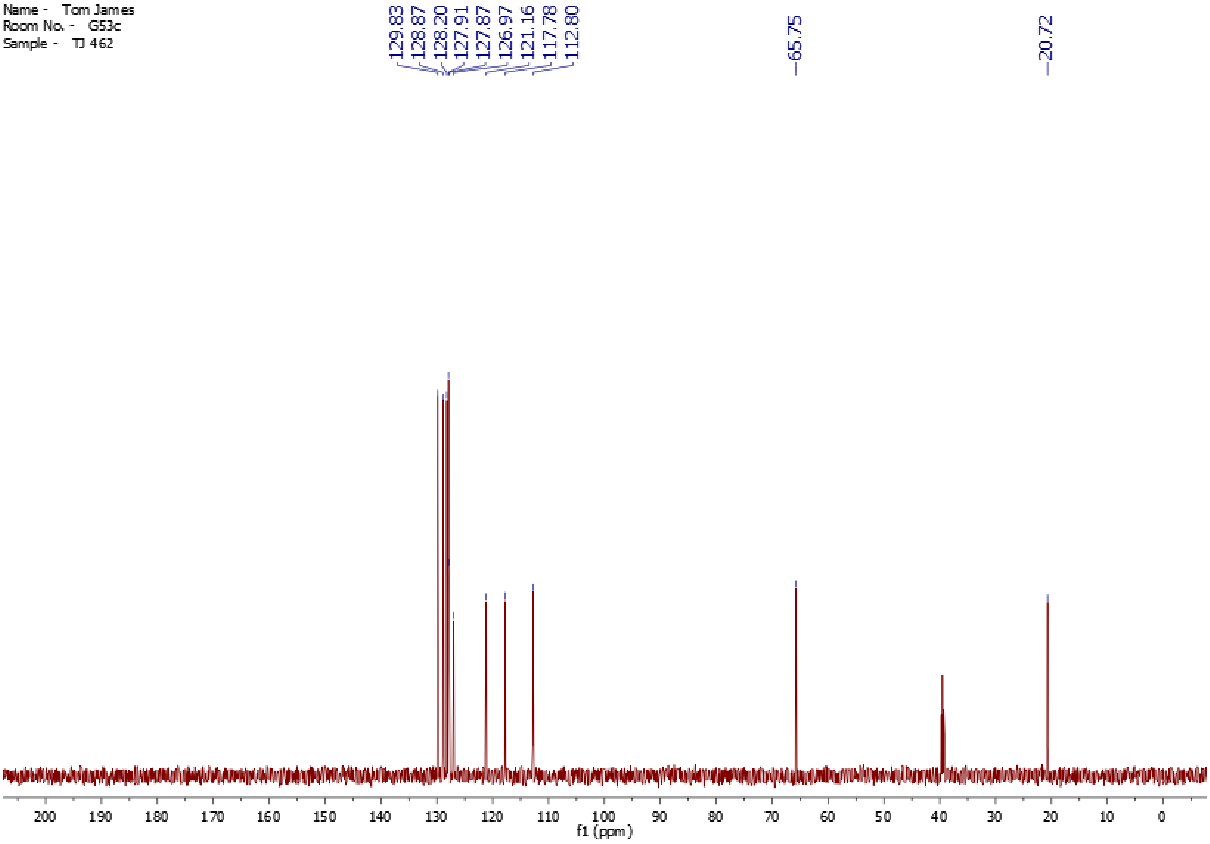

Methyl 3-(5-chloro-1H-indol-3-yl)-3-phenylpropanoate

Name - Tom James
Room No. - G53c
Sample - TJ 416 F1

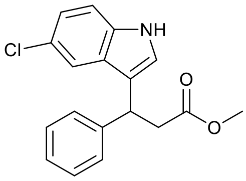

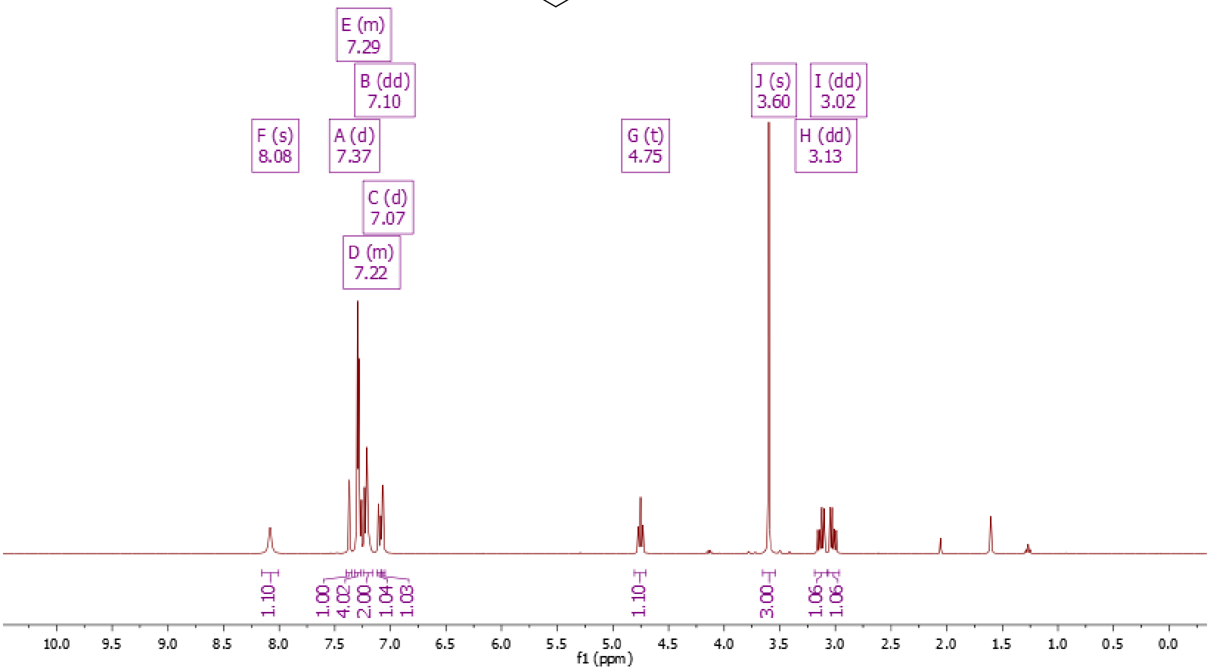

Name - Tom James
Room No. - G53c
Sample - TJ 463 F1

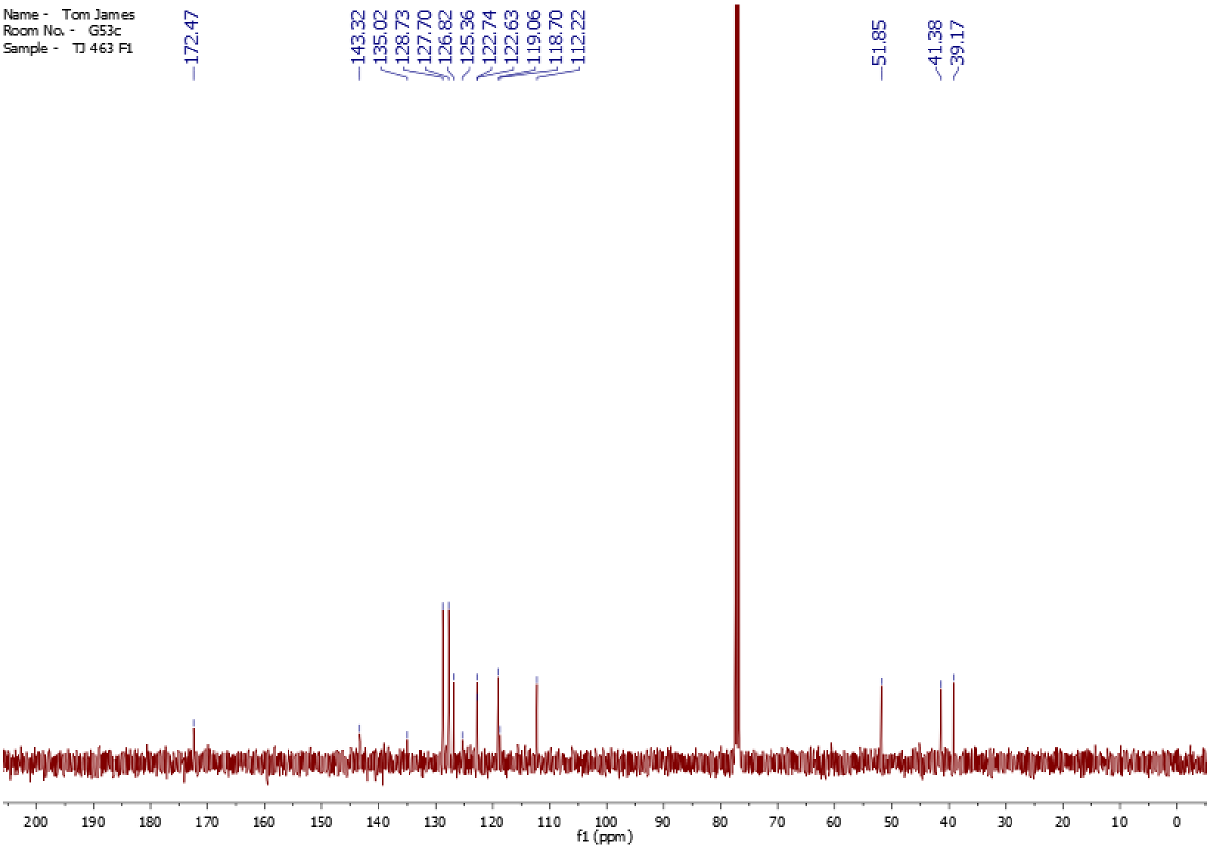

3-(5-Chloro-1H-indol-3-yl)-3-phenylpropanoic acid

Name - TOm James
Room No. - G53c
Sample - TJ 395 WU

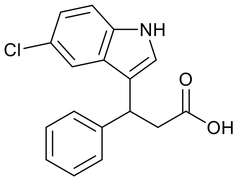

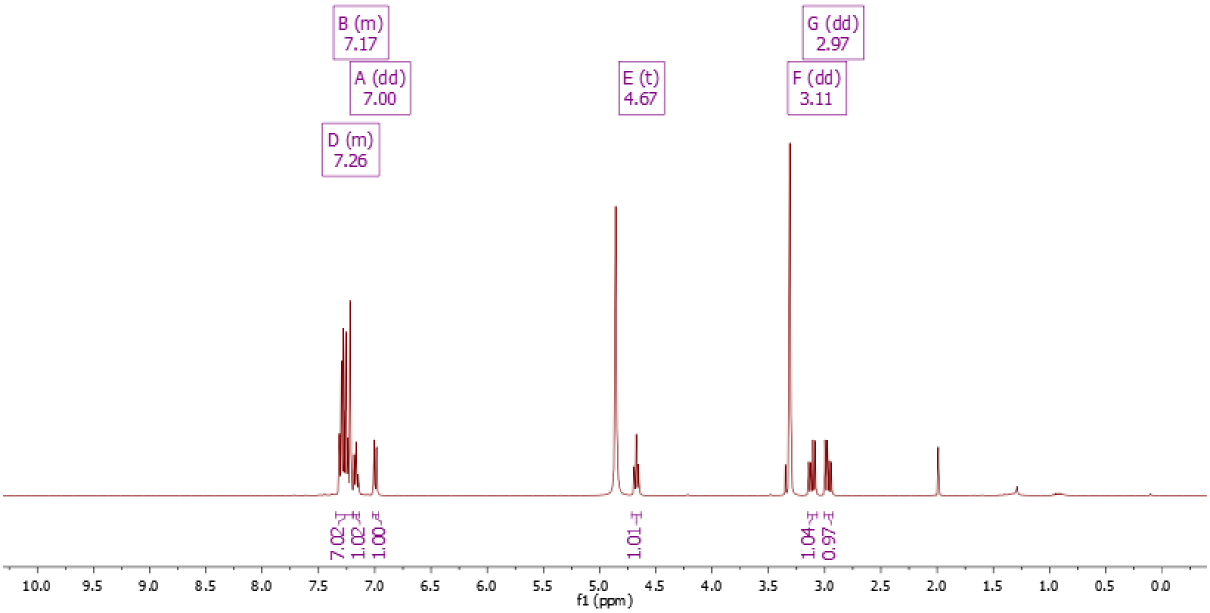

Name - Tom James
Room No. - G53c
Sample - TJ418

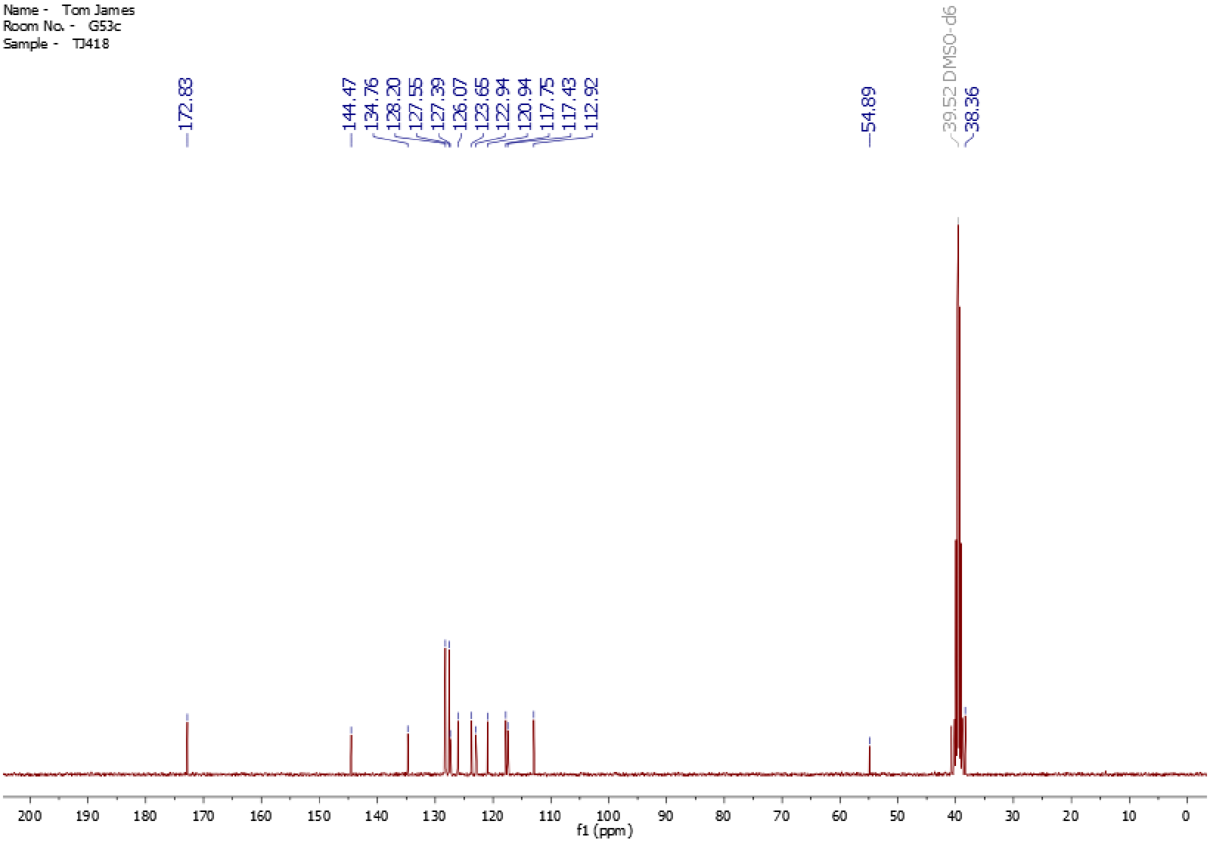

3-(5-Chloro-1H-indol-3-yl)-3-phenyl-*N*-[2-(pyridin-2-yl)ethyl]propanamide

Name - Tom James
Room No. - G53c
Sample - TJ 396 F1

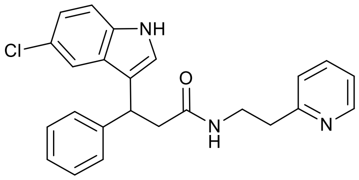

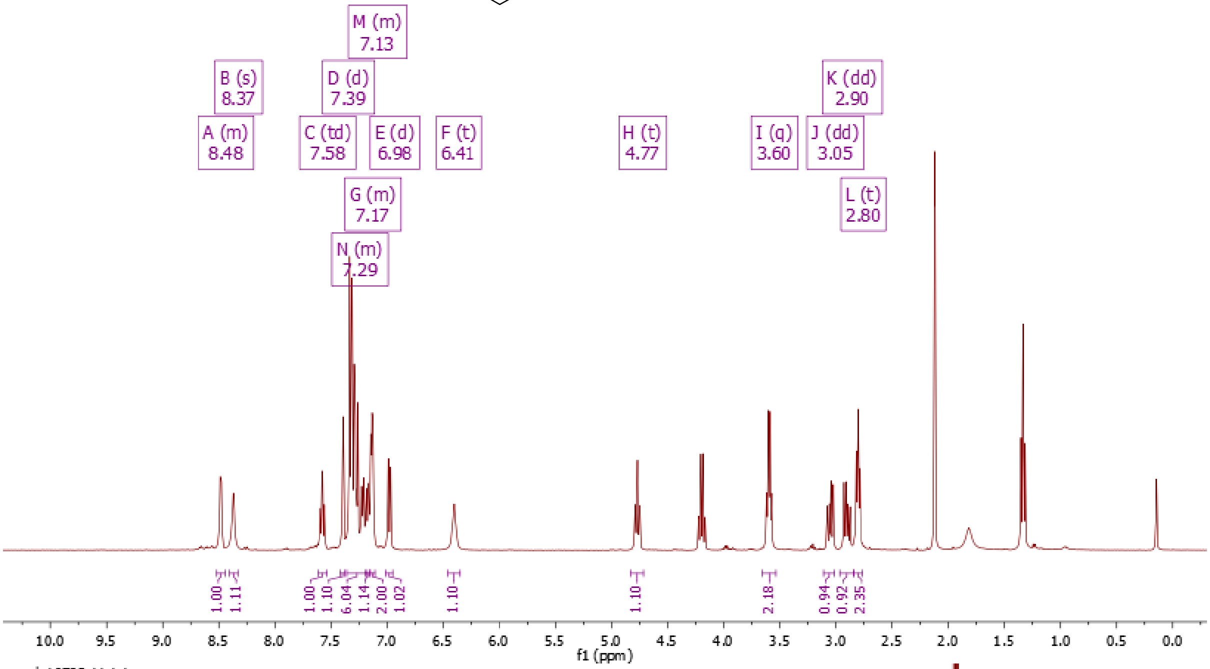

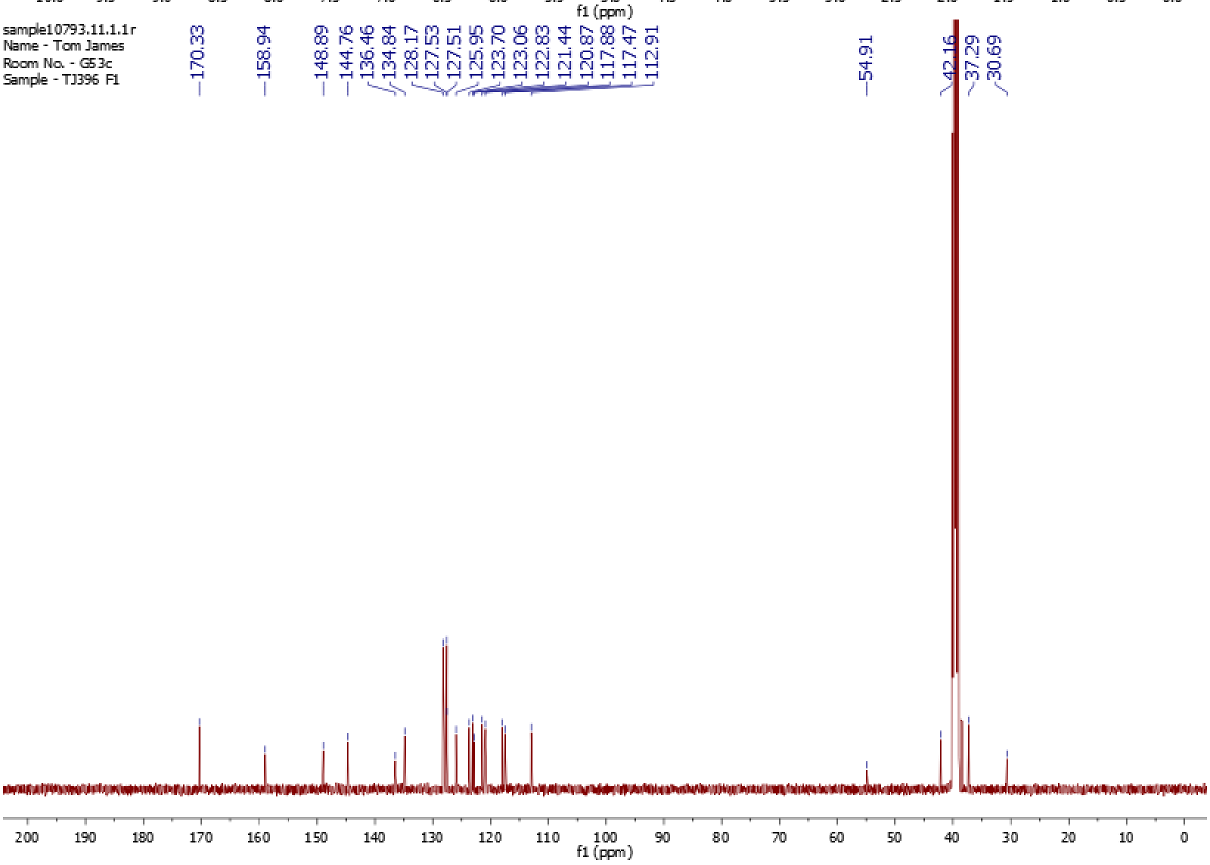

3-(5-Chloro-1H-indol-3-yl)-3-phenyl-*N*-(2-phenylethyl)propanamide

Name - Tom James
Room No. - G53c
Sample - TJ 422 B F1

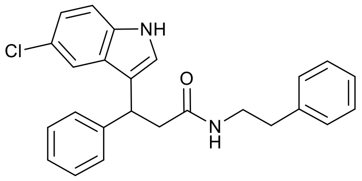

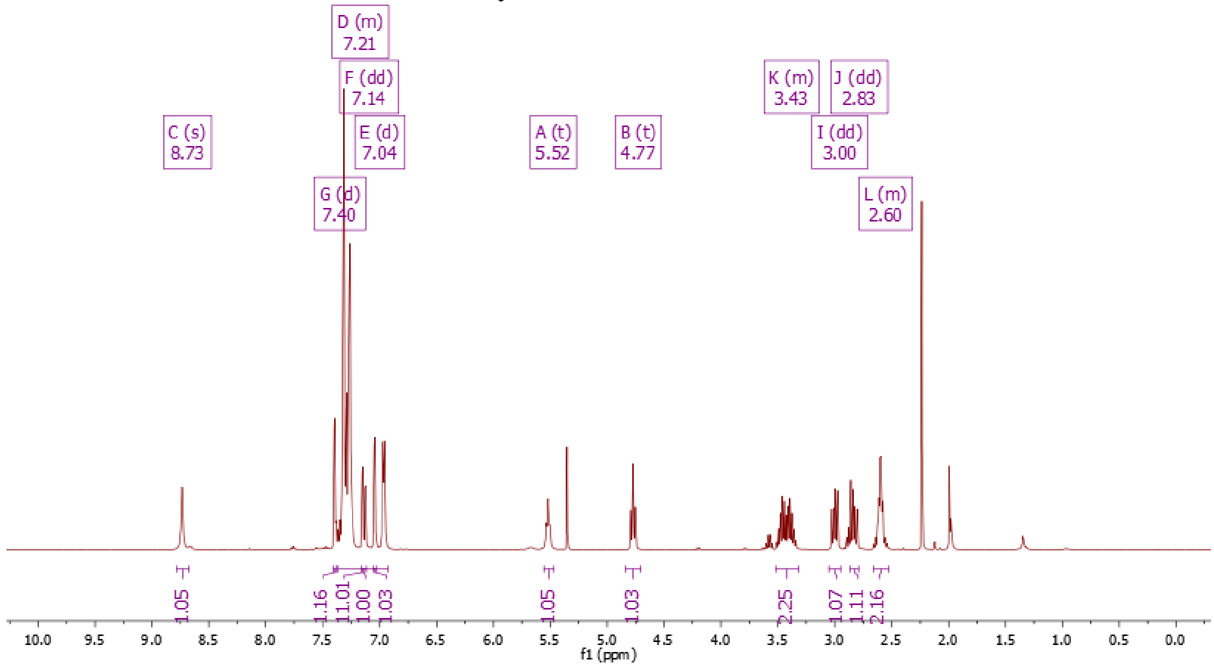

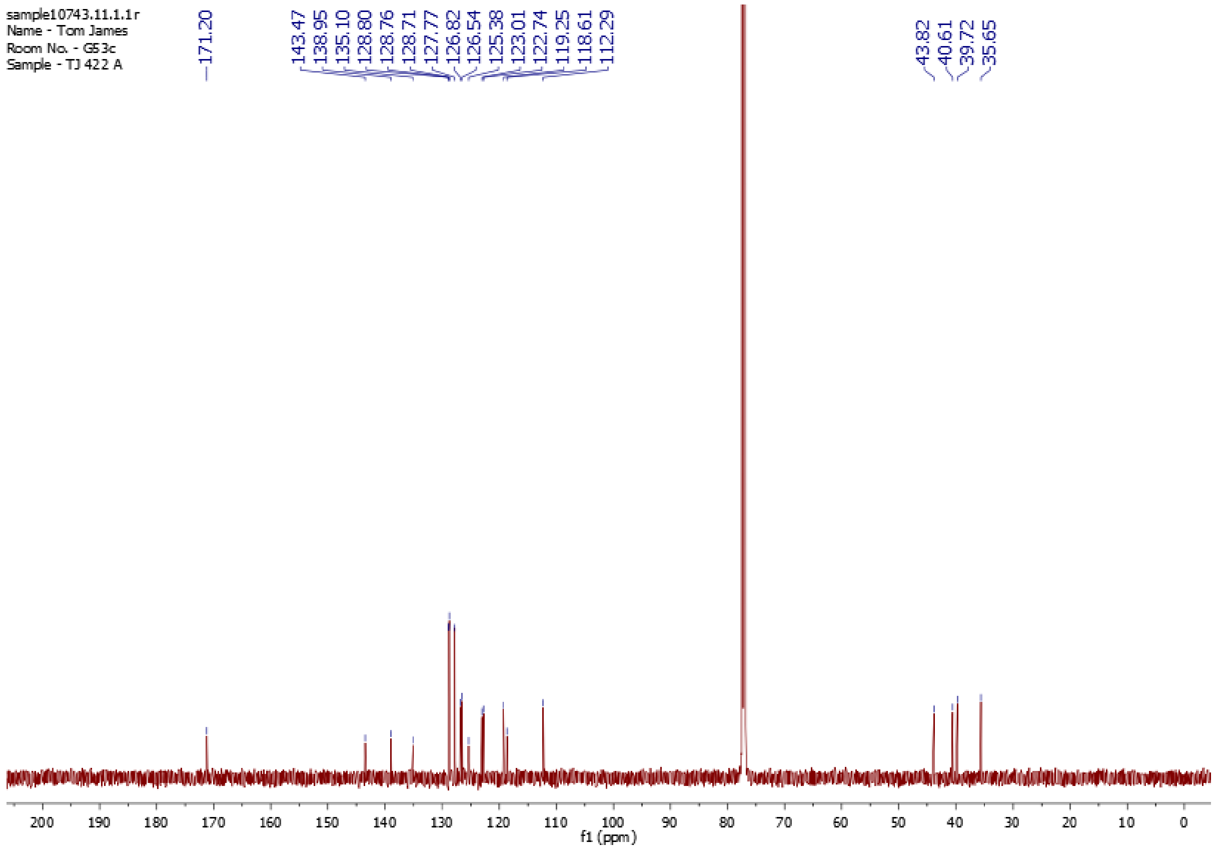

N-Benzyl-3-(5-chloro-1H-indol-3-yl)-3-phenylpropanamide

Name - Tom james
Room No. - G53c
Sample - TJ 420 B F1

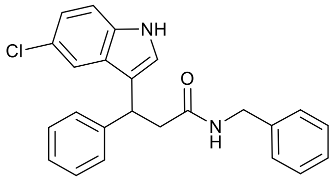

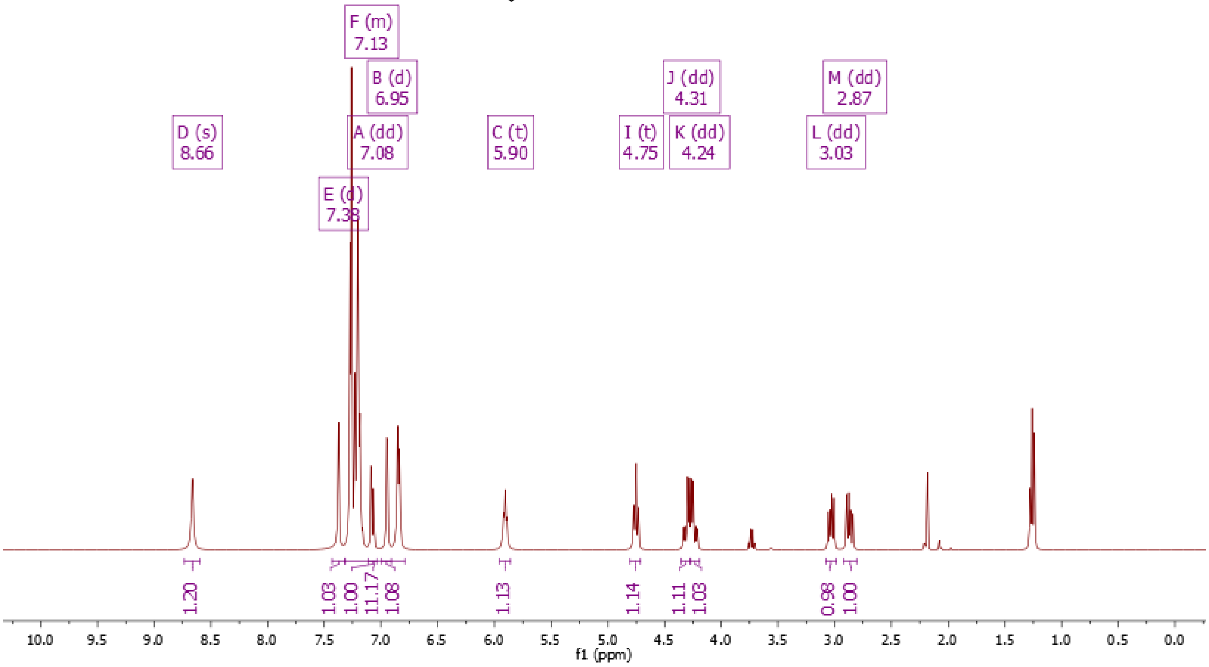

Name - Tom James
Room No. - G53c
Sample - TJ 420 B F1

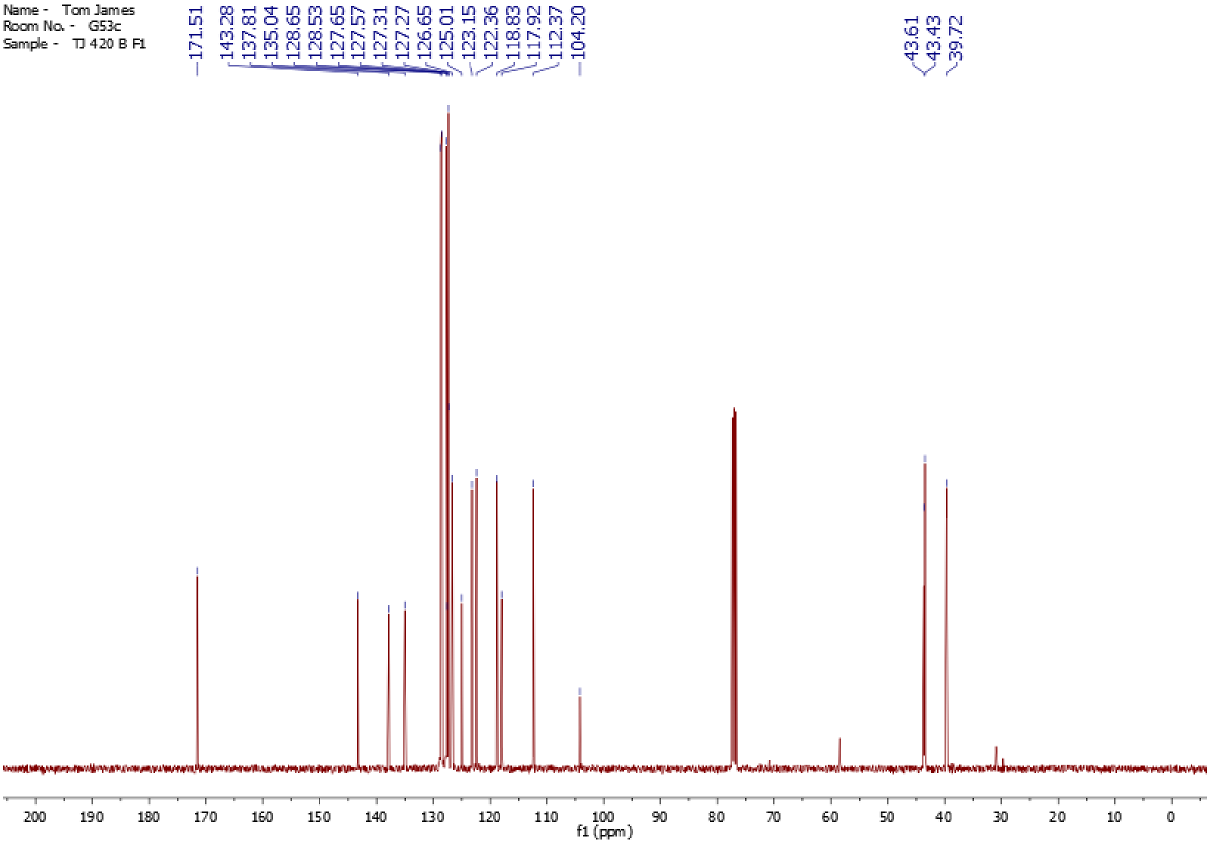

3-(5-Chloro-1H-indol-3-yl)-*N*-(2-methoxyethyl)-3-phenylpropanamide

Name - Tom james
Room No. - G53c
Sample - TJ 422 A F1

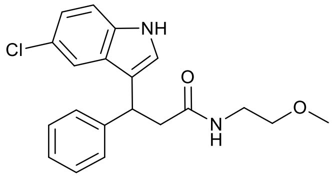

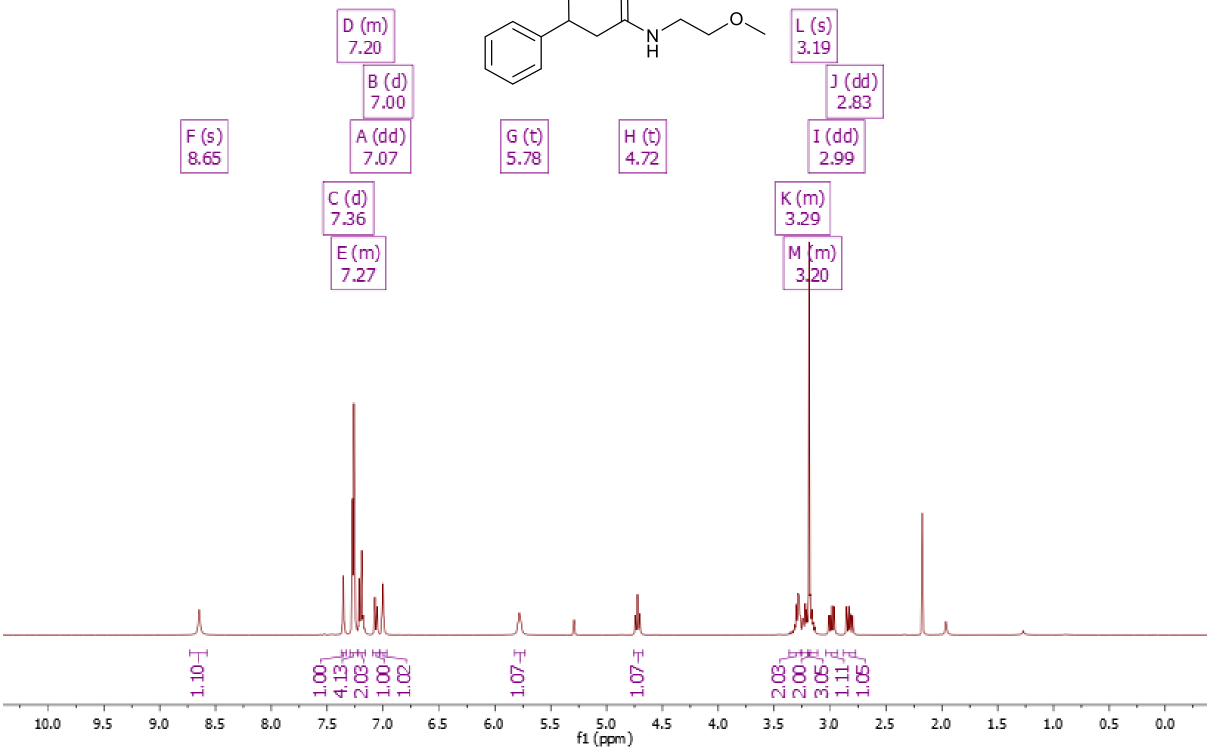

sample10744.11.1.1r
Name - Tom James
Room No. - G53c
Sample - TJ 422 B

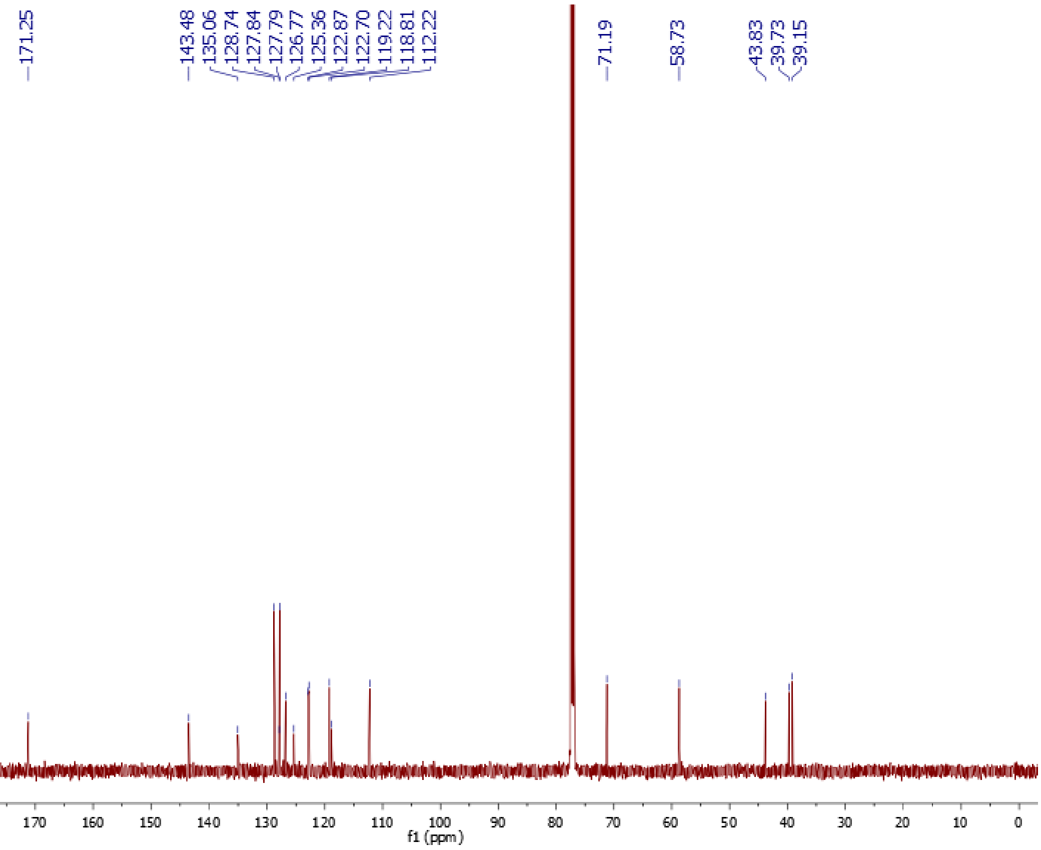

Methyl 3-(2-bromo-6-chloro-1H-indol-3-yl)-3-(5-chlorothiophen-2-yl)propanoate

sample12980.10.1.1r
Name - SC022-1
Room No. - G53c
Sample - SC022-1

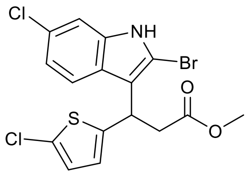

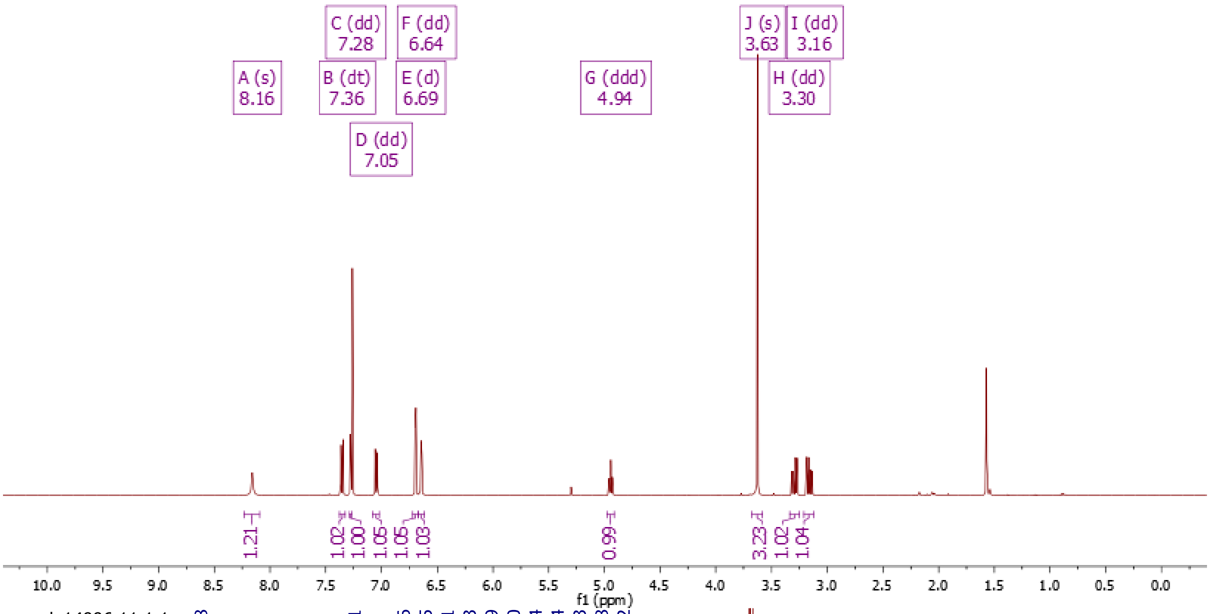

sample14996.11.1.1r
Name - Sergio
Room No. - G53c
Sample - SC022-1

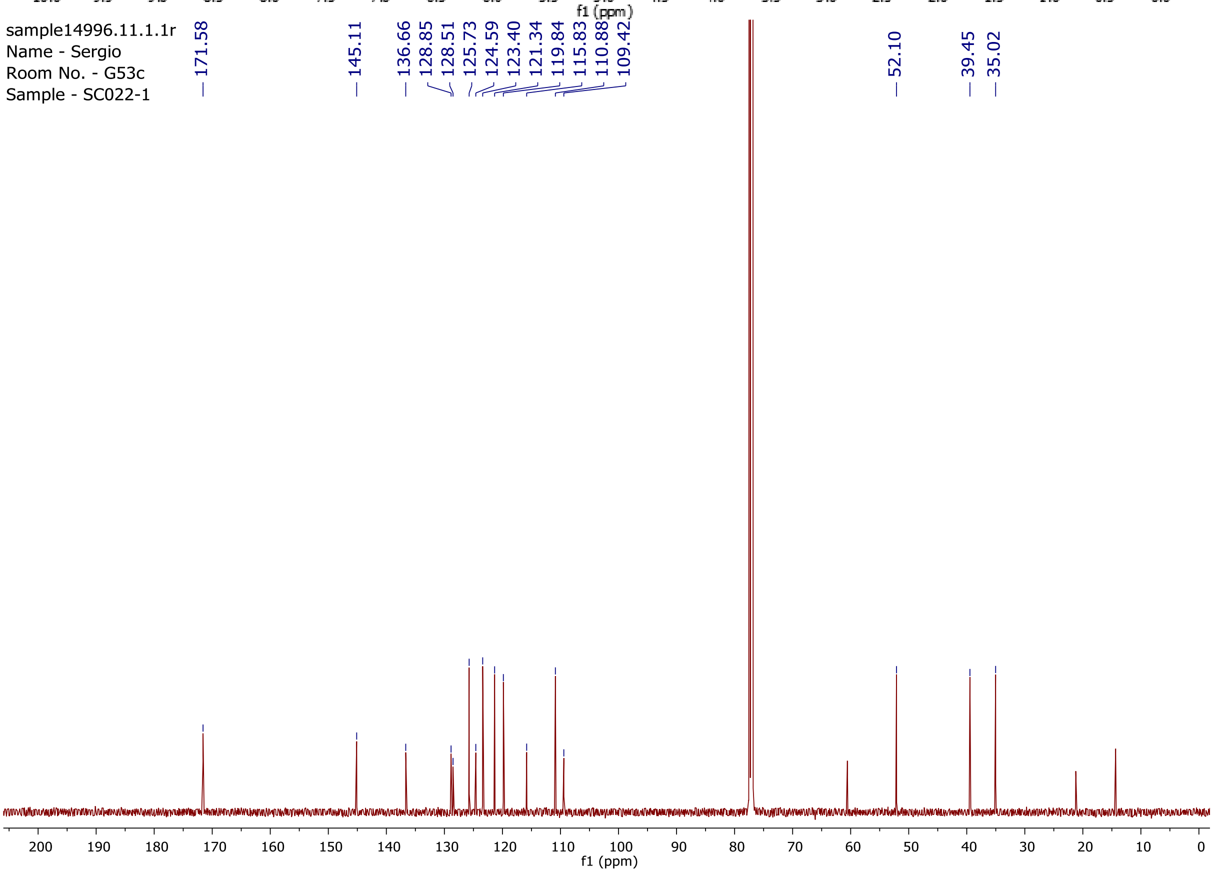

tert-Butyl 2-bromo-6-chloro-3-[1-(5-chlorothiophen-2-yl)-3-methoxy-3-oxopropyl]-indole-1-carboxylate

sample13015.10.1.1r
Name - SC021-1
Room No. - G53c
Sample - SC021-1

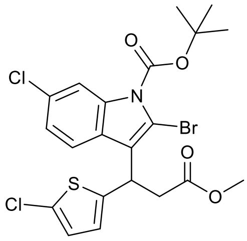

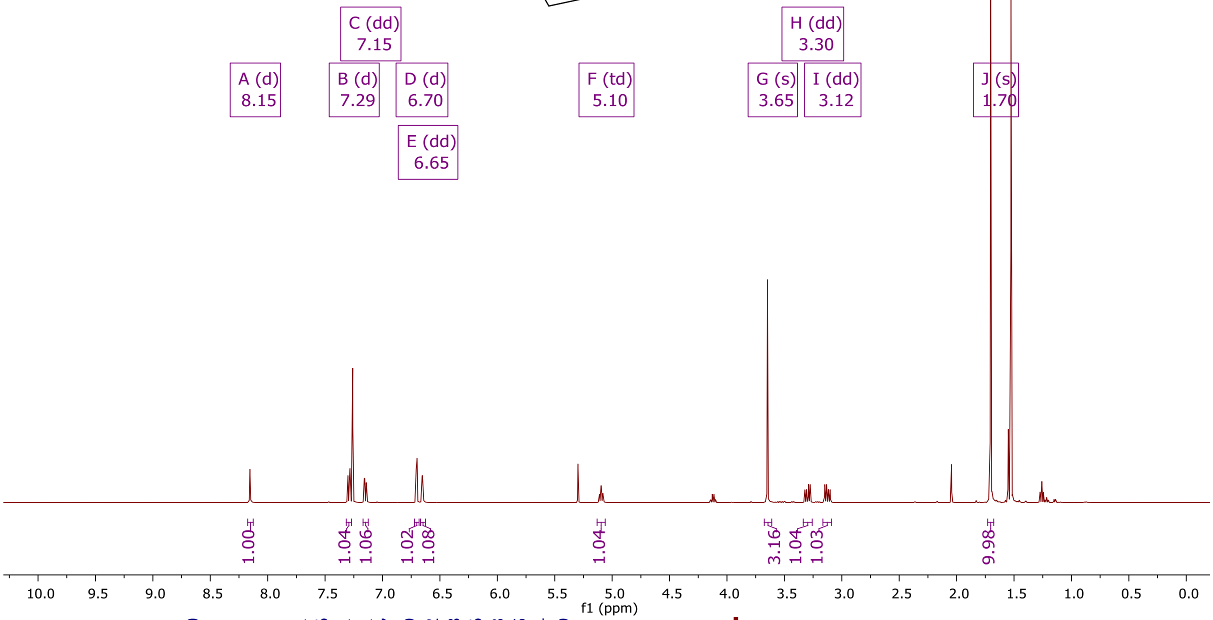

sample13015.11.1.1r
Name - SC021-1
Room No. - G53c
Sample - SC021-1

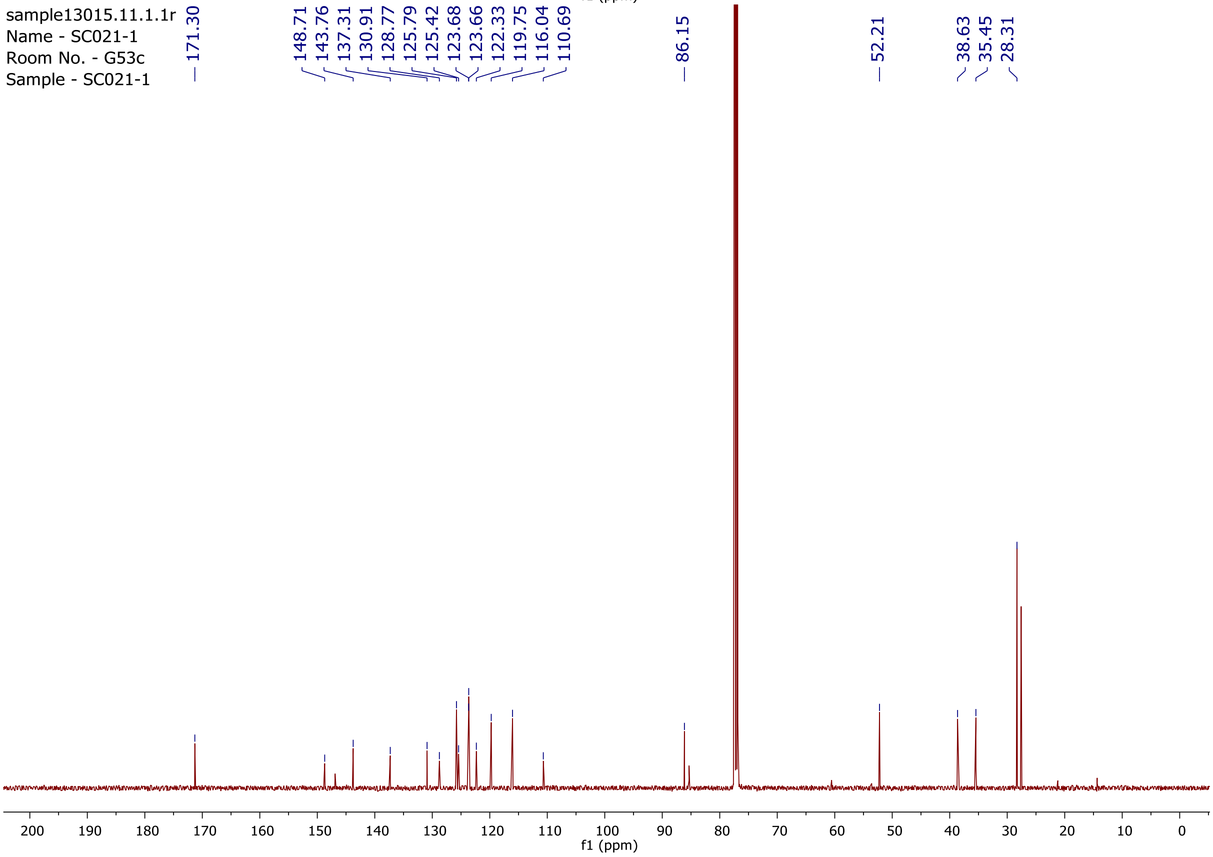

3-(2-Bromo-6-chloro-1H-indol-3-yl)-3-(5-chlorothiophen-2-yl)propanoic acid

sample13048.10.1.1r
Name - SC023-1
Room No. - G53c
Sample - SC023-1

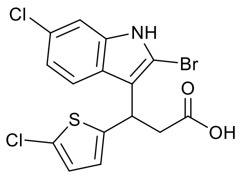

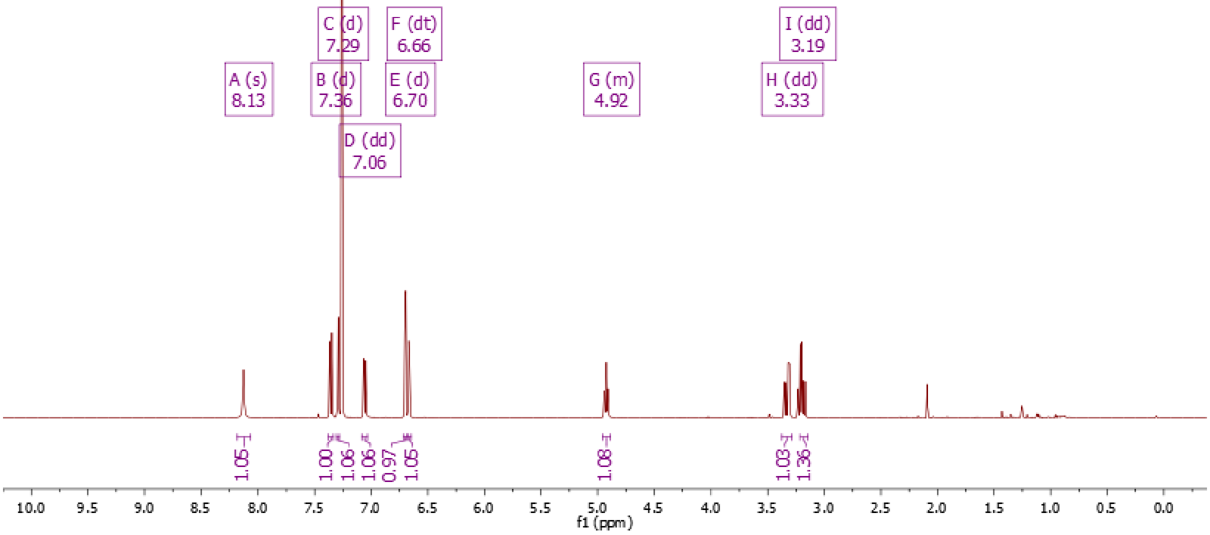

sample13048.11.1.1r
Name - SC023-1
Room No. - G53c
Sample - SC023-1

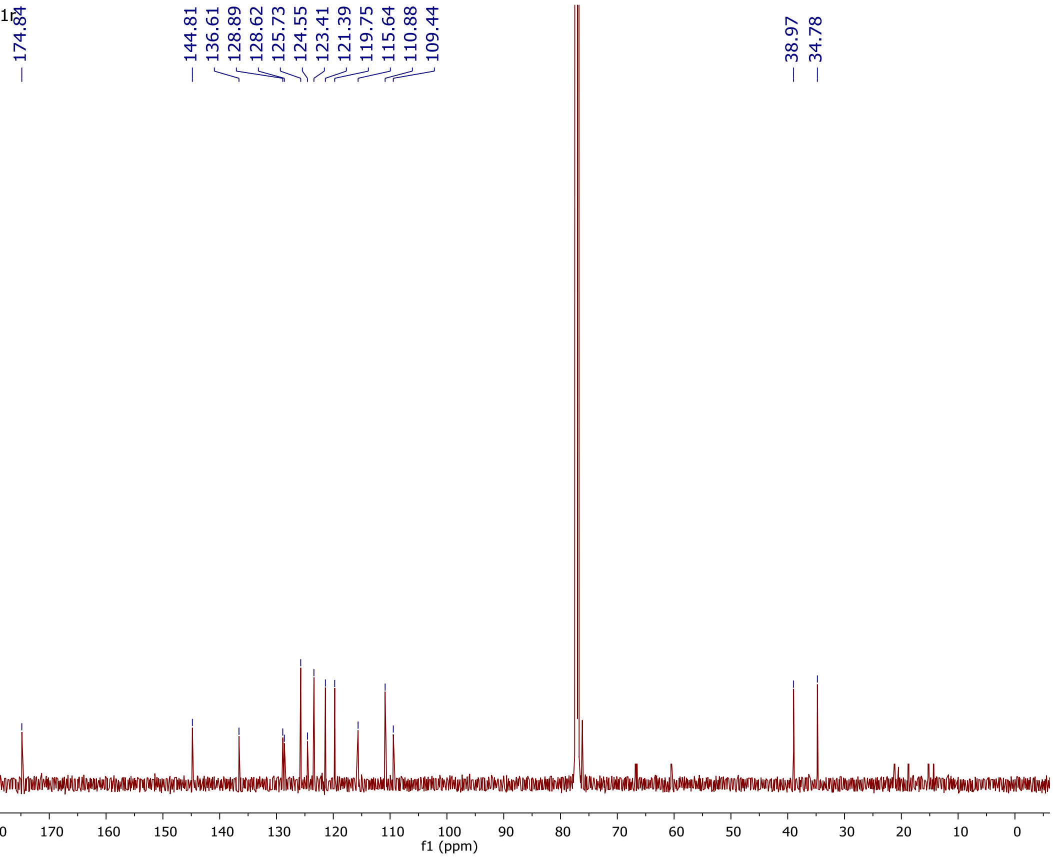

3-(2-Bromo-6-chloro-1H-indol-3-yl)-3-(5-chlorothiophen-2-yl)-*N*-[2-(pyridin-2-yl)ethyl]propanamide

sample13573.10.1.1r
Name - Sergi Celis
Room No. - G53c
Sample - SC025-1

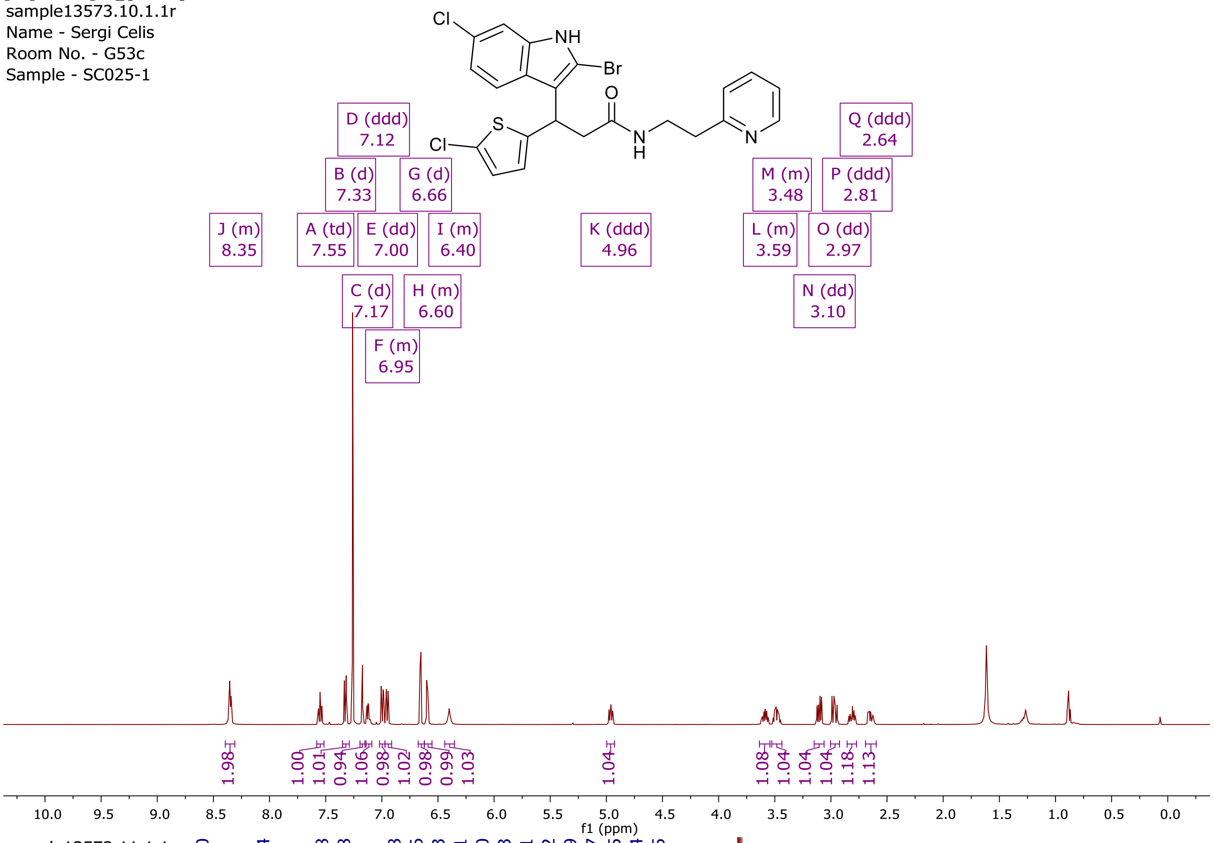

sample13573.11.1.1r
Name - Sergi Celis
Room No. - G53c
Sample - SC025-1

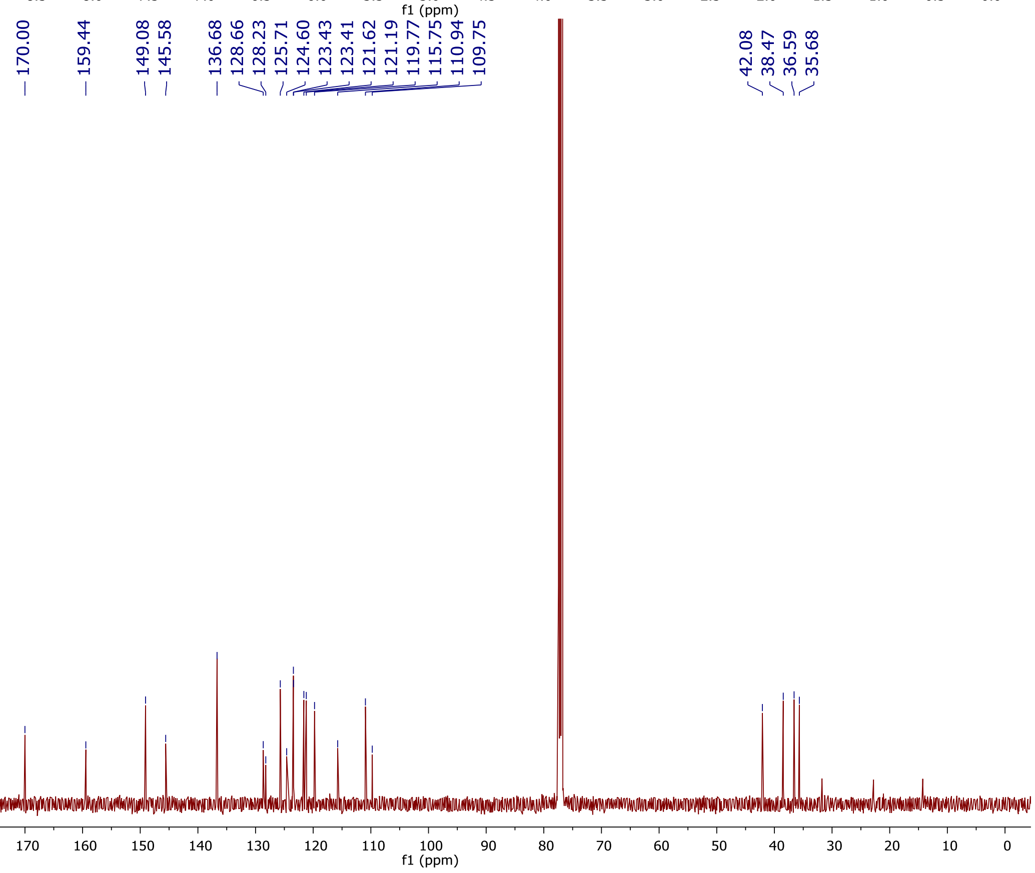

Methyl 3-(5-chloro-1H-indol-3-yl)-3-(5-chlorothiophen-2-yl)-2,2-dimethyl propanoate

sample13318.10.1.1r
Name - Sergi Celis
Room No. - G53c
Sample - SC035-1

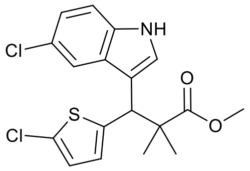

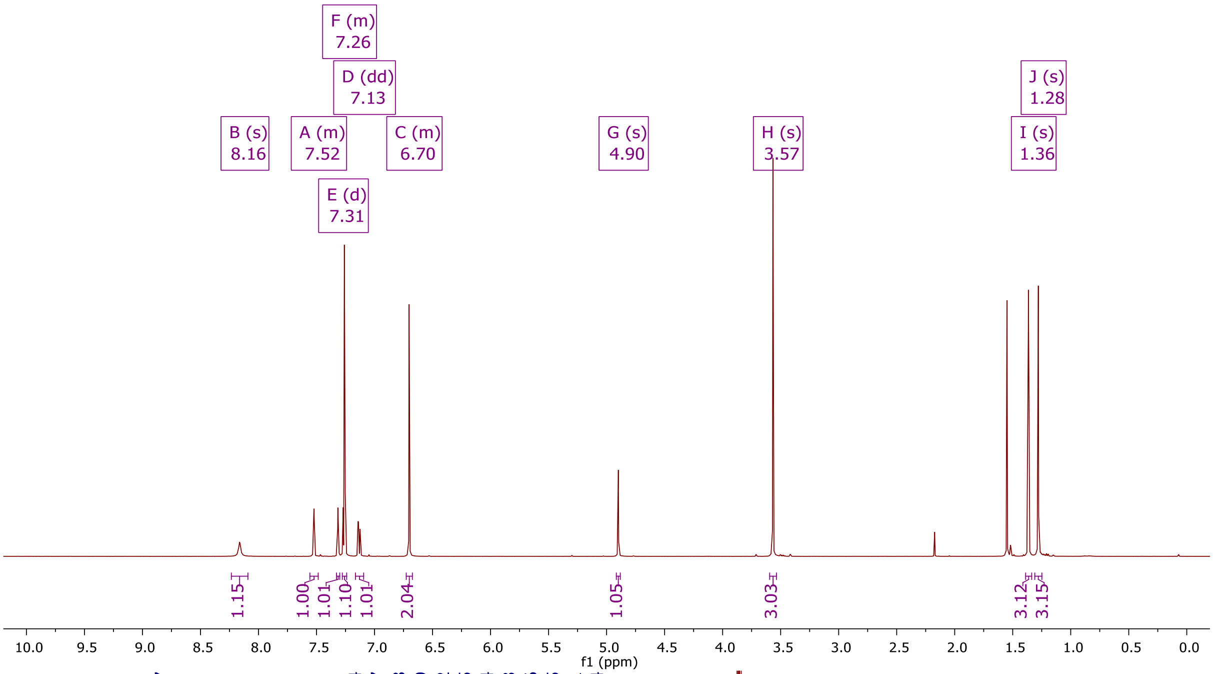

sample13318.11.1.1r
Name - Sergi Celis
Room No. - G53c
Sample - SC035-1

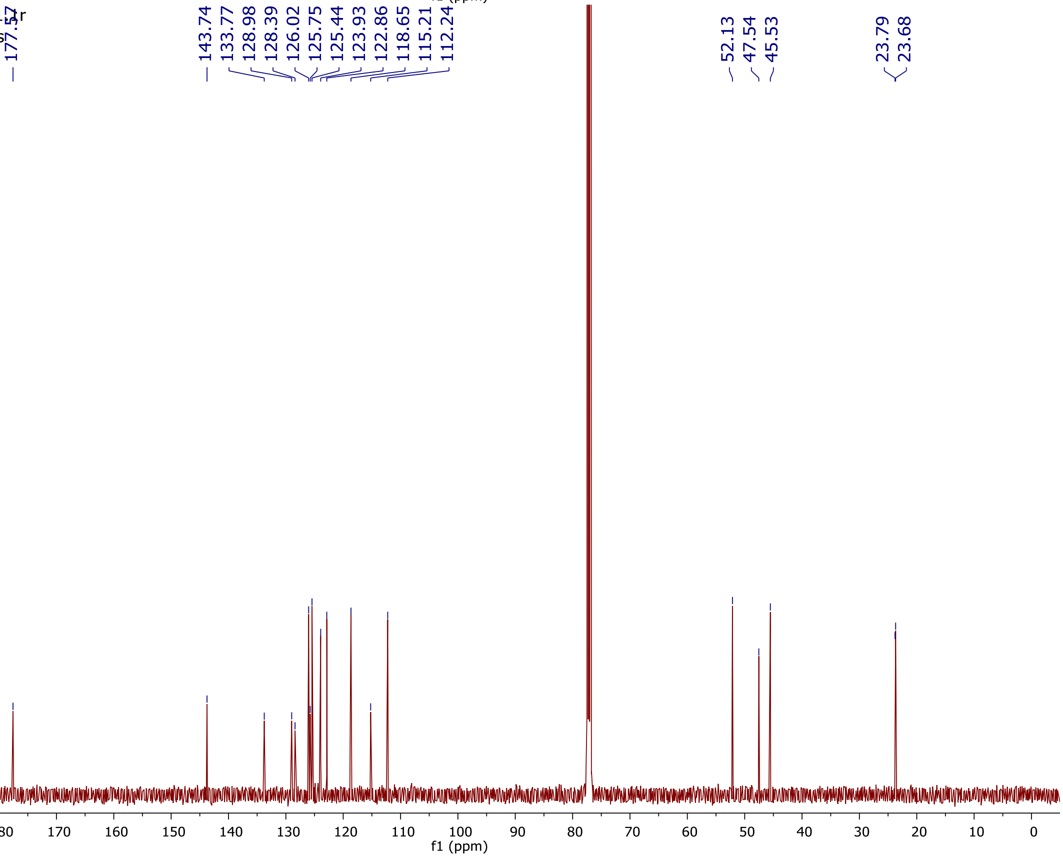

3-(5-Chloro-1H-indol-3-yl)-3-(5-chlorothiophen-2-yl)-2,2-dimethylpropanoic acid

sample13452.10.1.1r
Name - Sergi Celis
Room No. - G53c
Sample - SC038-1

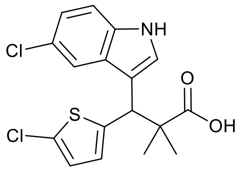

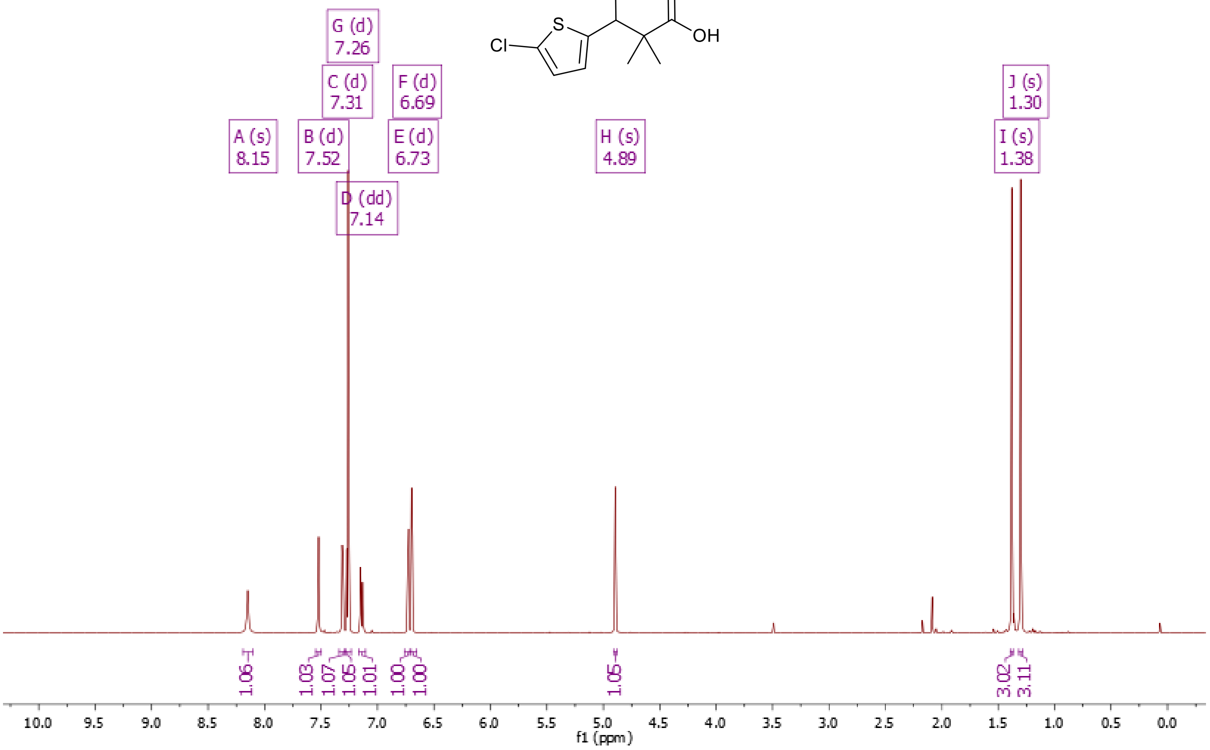

sample13452.11.1.1r
Name - Sergi Celis
Room No. - G53c
Sample - SC038-1

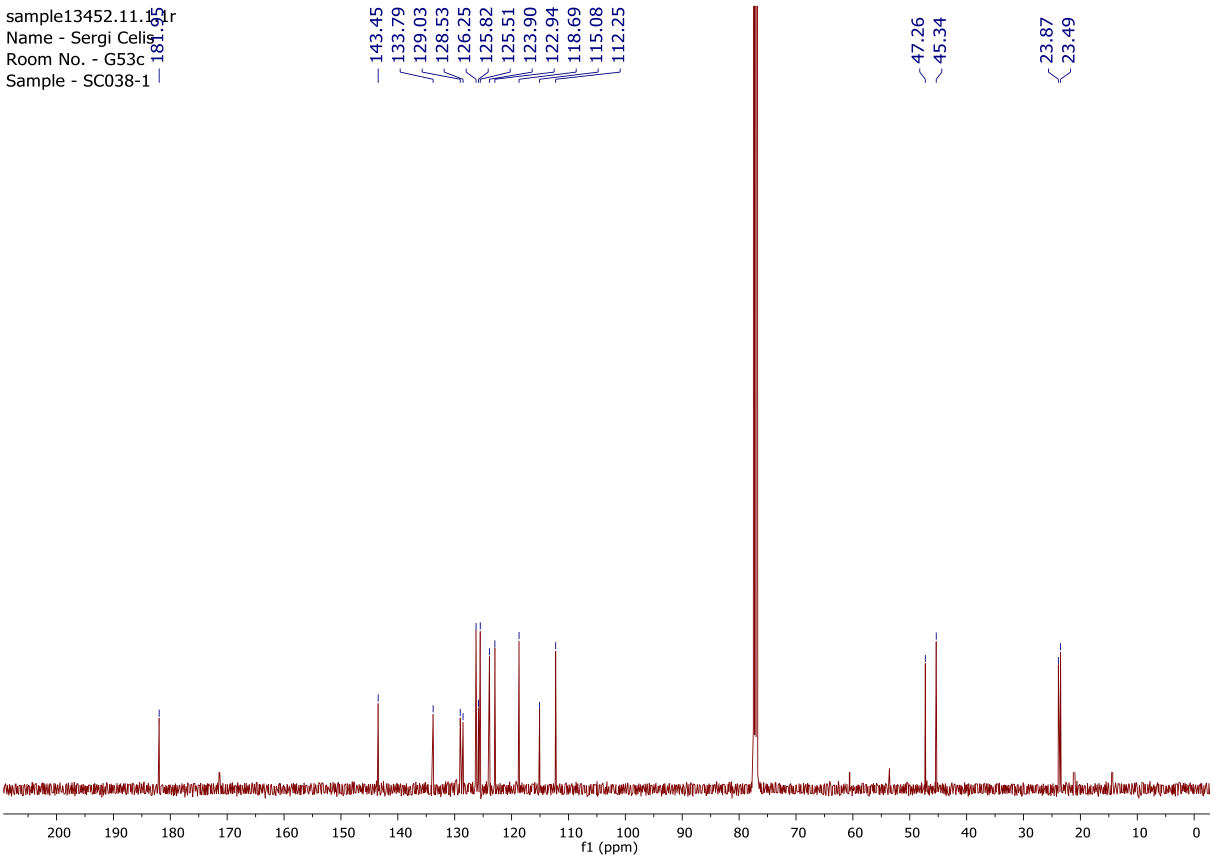

3-(5-Chloro-1H-indol-3-yl)-3-(5-chlorothiophen-2-yl)-2,2-dimethyl-*N*-[2-(pyridin-2-yl)ethyl]propanamide

sample13527.10.1.1r
Name - Sergi Celis
Room No. - G53c
Sample - SC043-1

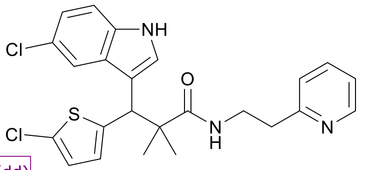

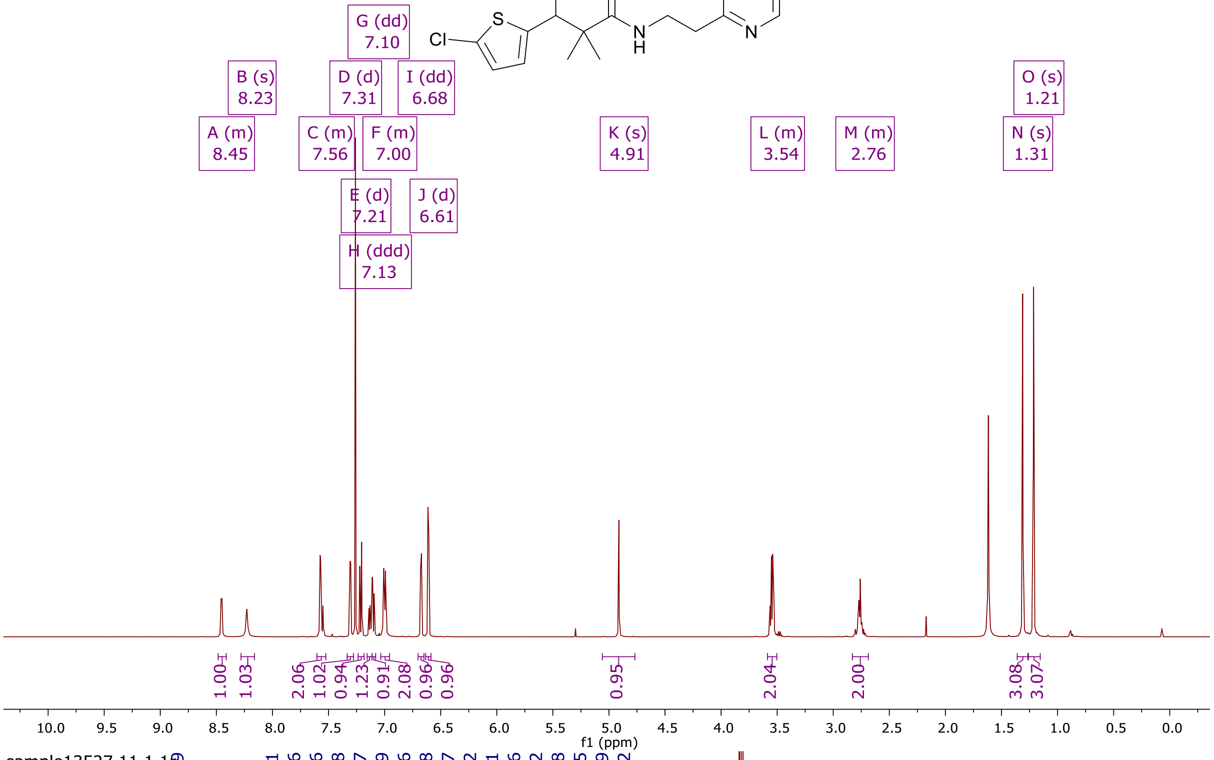

sample13527.11.1.1r
Name - Sergi Celis
Room No. - G53c
Sample - SC043-1

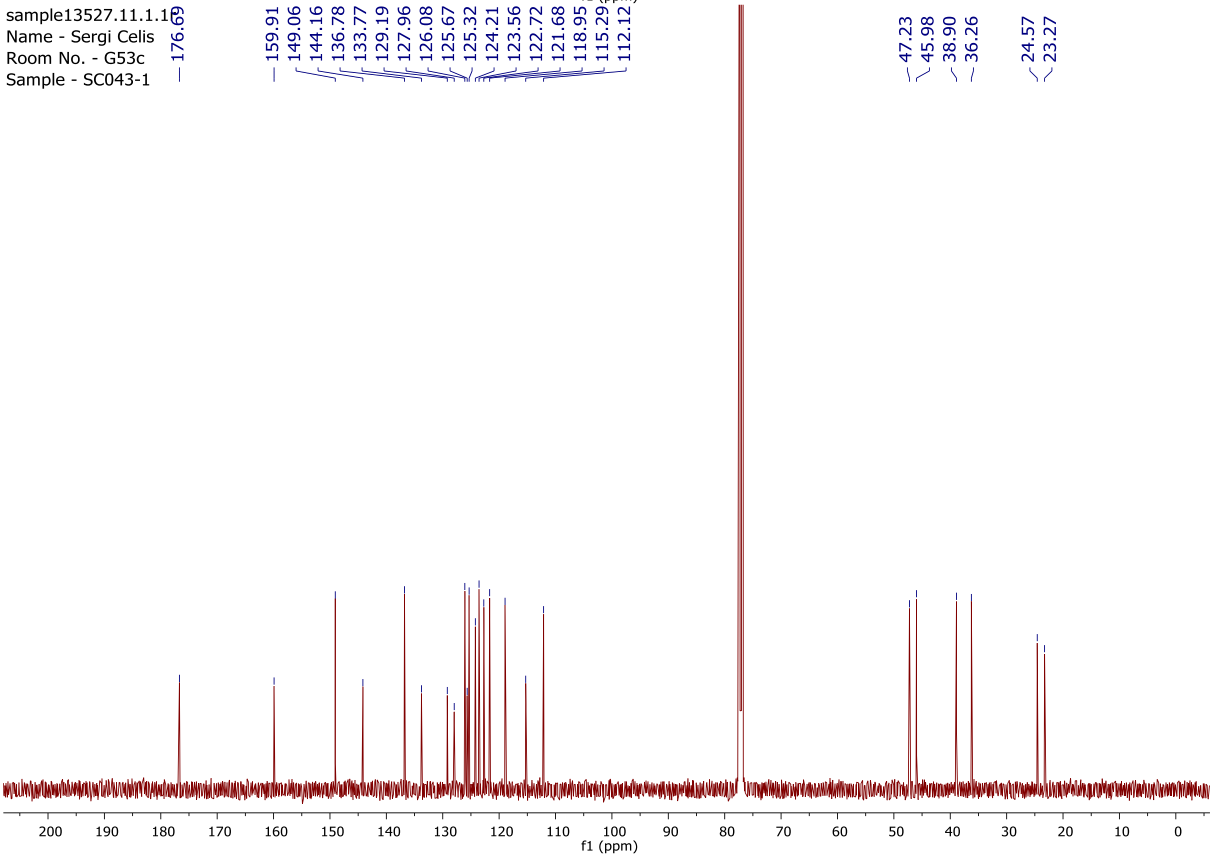

rac-syn- and *rac-anti*-Methyl 3-(5-chloro-1H-indol-3-yl)-3-(5-chlorothiophen-2-yl)-2-methylpropanoate

sample13448.10.1.1r
Name - Sergio Celis
Room No. - G53c
Sample - SC040-1

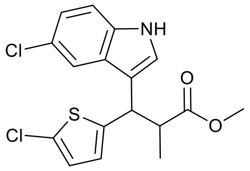

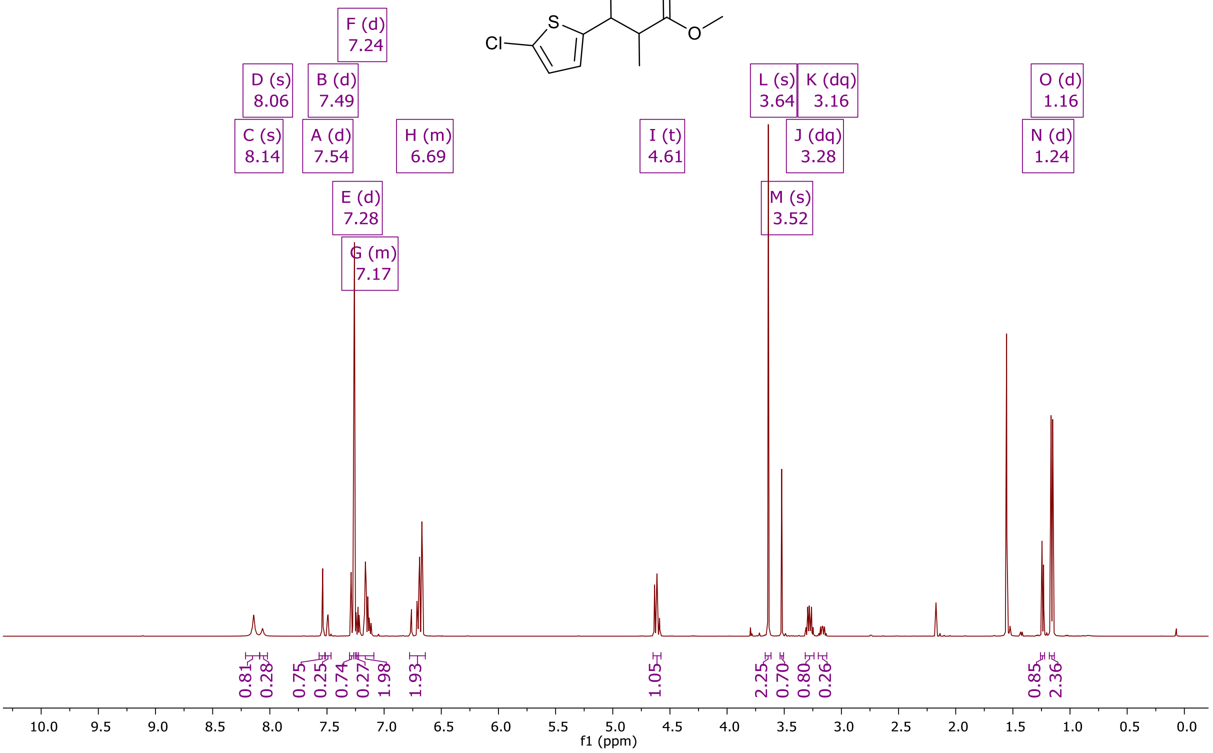

sample13448.11.1.1r
Name - Sergio Celis
Room No. - G53c
Sample - SC040-1

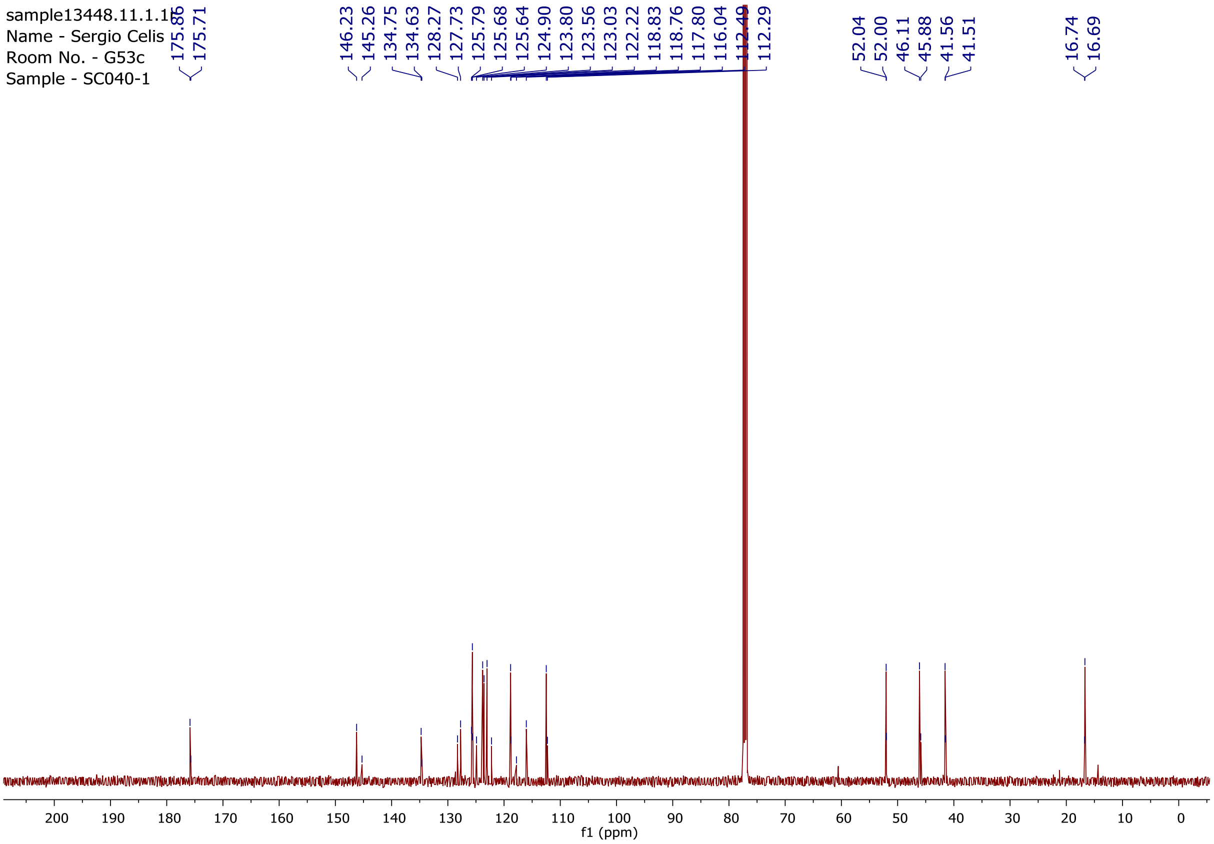

rac-syn- and *rac-anti*-3-(5-Chloro-1H-indol-3-yl)-3-(5-chlorothiophen-2-yl)-2-methylpropanoic acid

sample13476.10.1.1r
Name - Sergio celis
Room No. - G53c
Sample - SC042-1

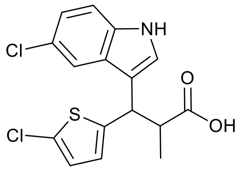

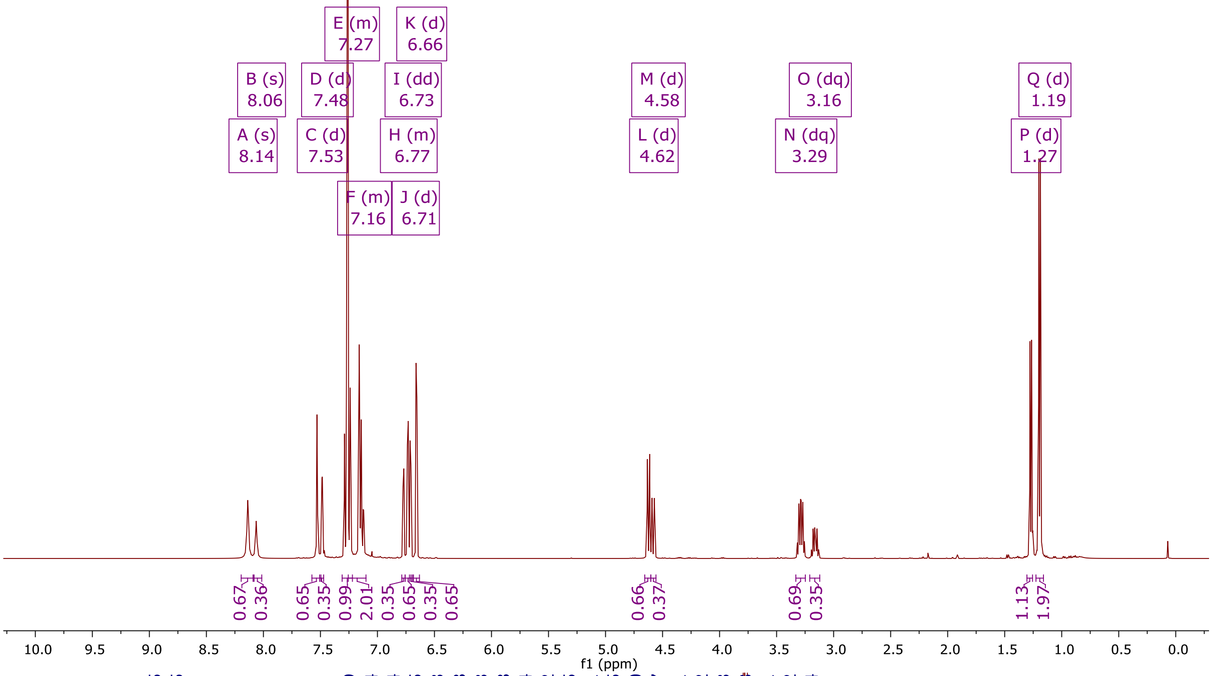

sample13476.11.1.1r
Name - Sergio celis
Room No. - G53c
Sample - SC042-1

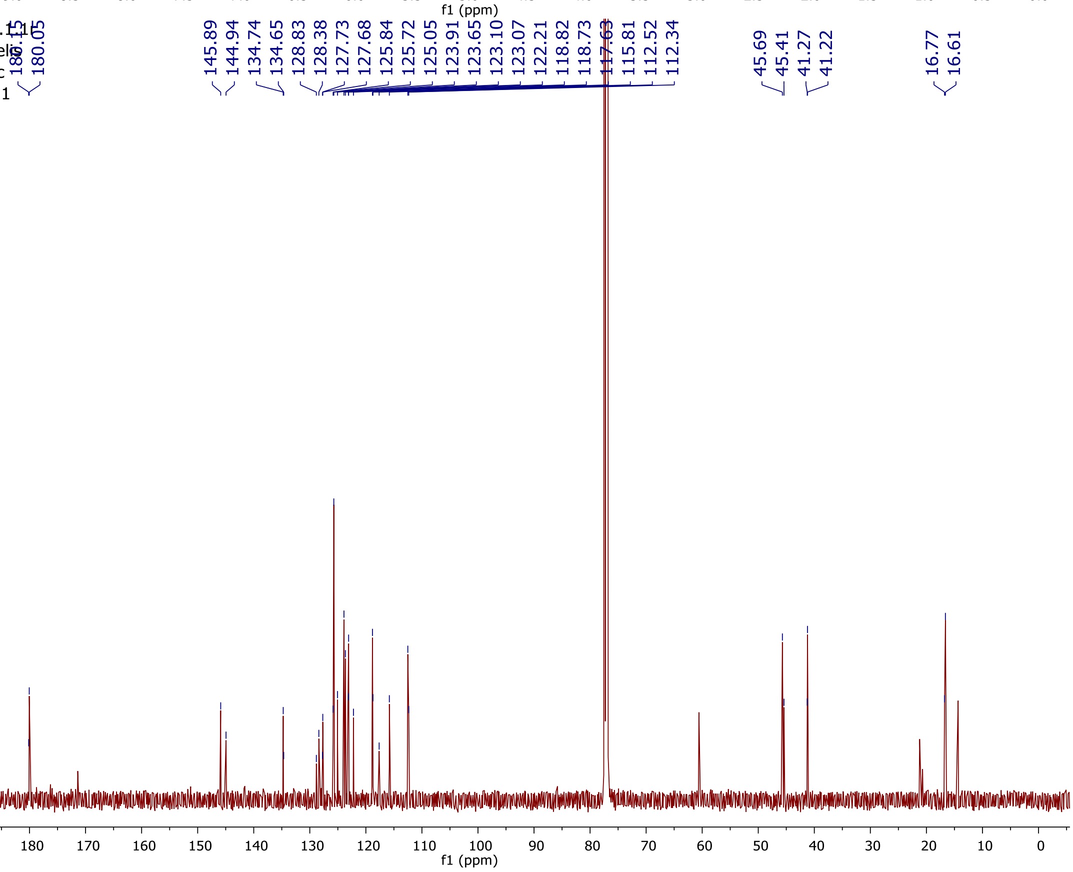

rac-syn-3-(5-Chloro-1H-indol-3-yl)-3-(5-chlorothiophen-2-yl)-2-methyl-*N*-[2-(pyridin-2-yl)ethyl]propanamide

sample13707.10.1.1r
Name - Sergi Celis
Room No. - G53c
Sample - SC044-1

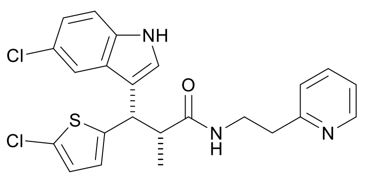

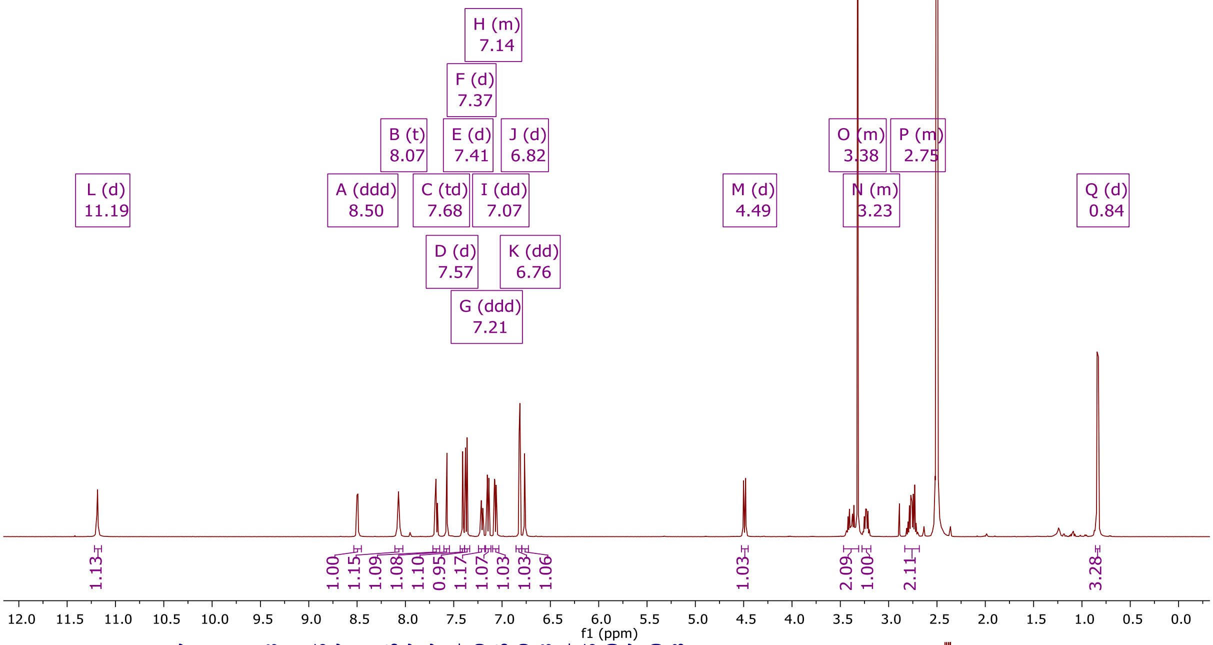

sample13707.11.1.1r
Name - Sergi Celis
Room No. - G53c
Sample - SC044-1

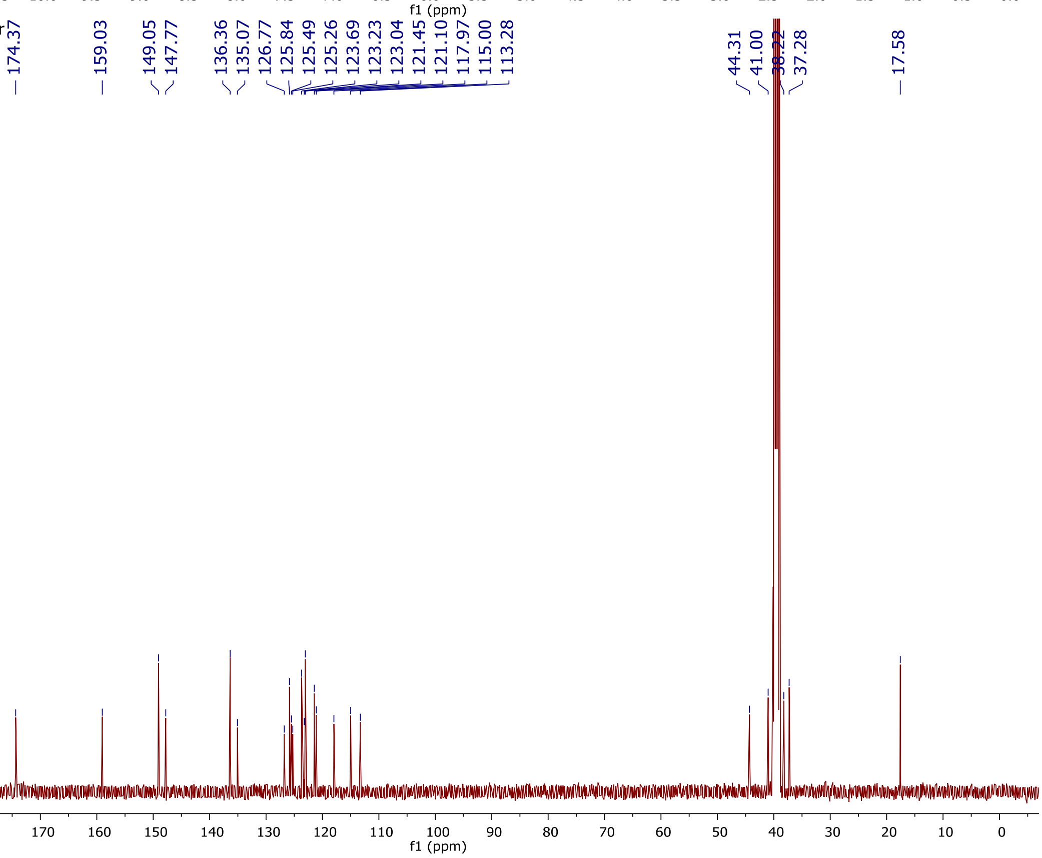

***rac-anti*-3-(5-Chloro-1H-indol-3-yl)-3-(5-chlorothiophen-2-yl)-2-methyl-*N*-[2-(pyridin-2-yl)ethyl]propanamide**

sample13708.10.1.1r
Name - Sergi Celis
Room No. - G53c
Sample - SC044-2

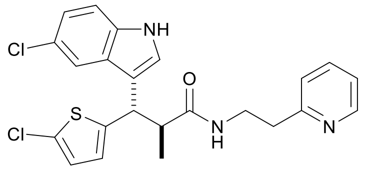

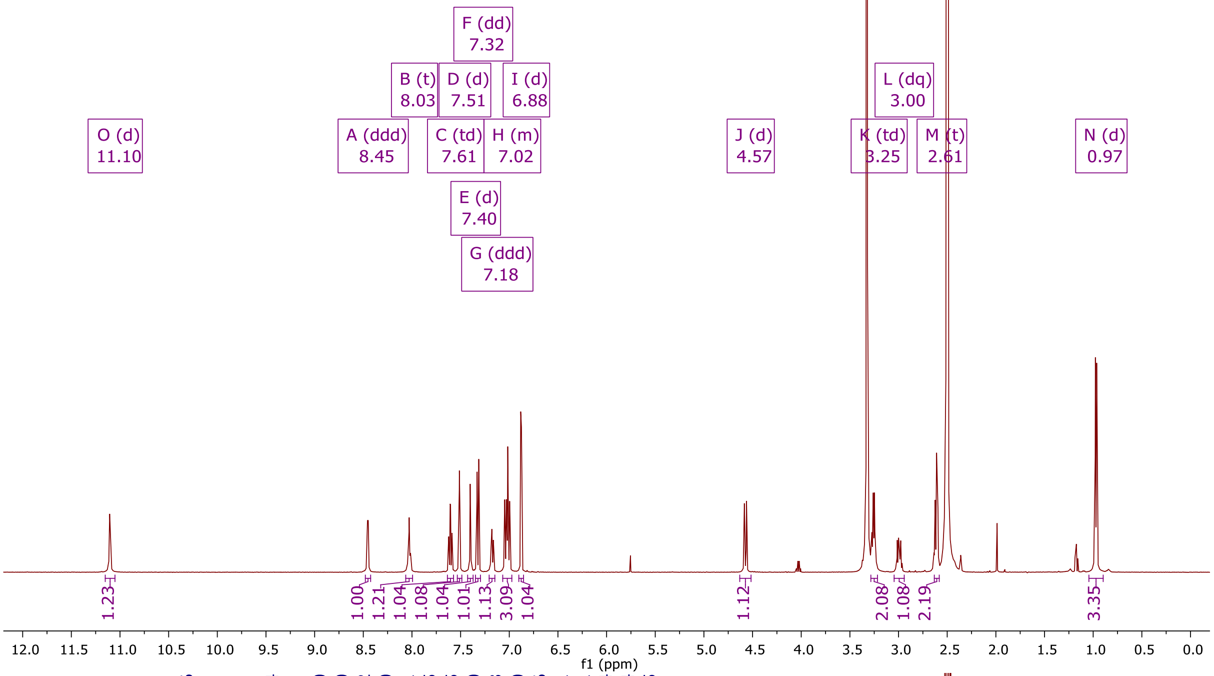

sample13708.11.1.1r
Name - Sergi Celis
Room No. - G53c
Sample - SC044-2

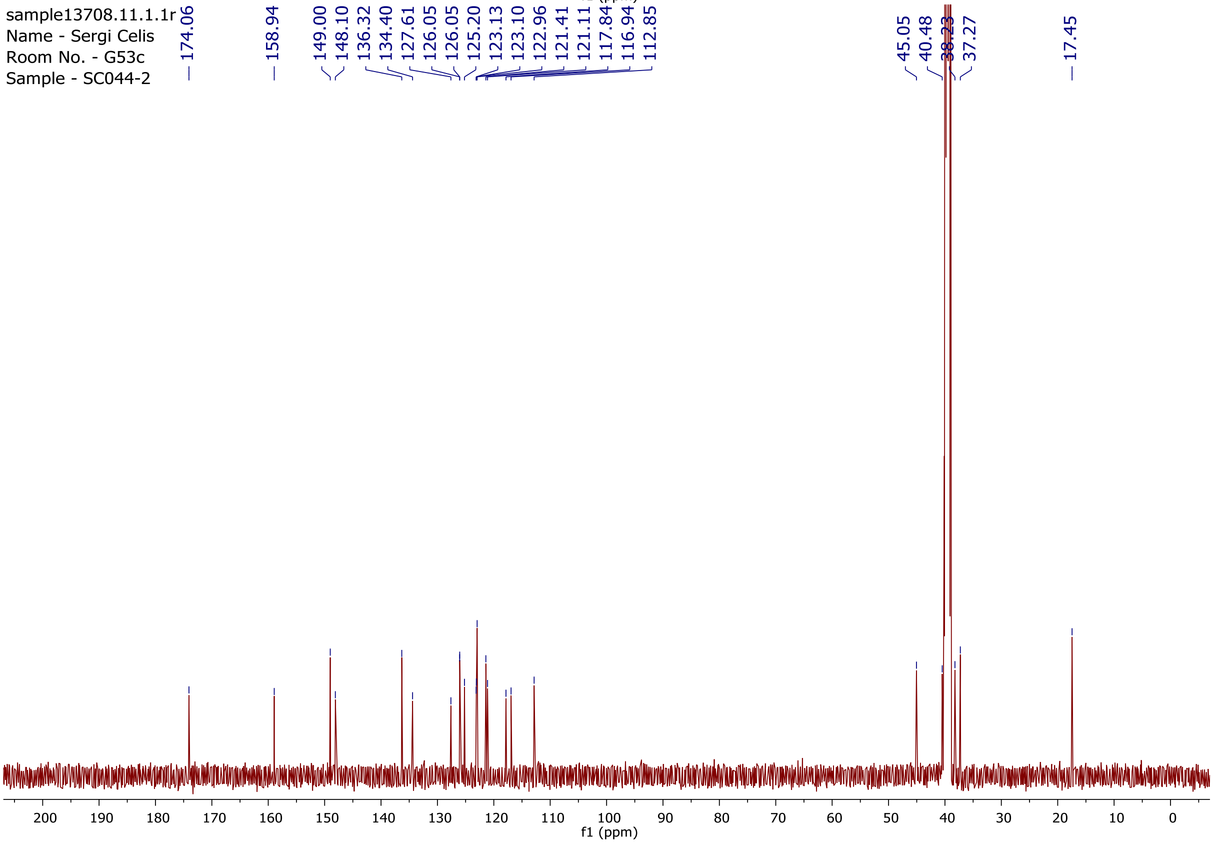

Methyl 3-(2-bromo-5-chloro-1H-indol-3-yl)-3-(5-chlorothiophen-2-yl)propanoate

sample13572.10.1.1r
Name - Sergi celis
Room No. - G53c
Sample - SC049-1

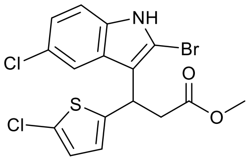

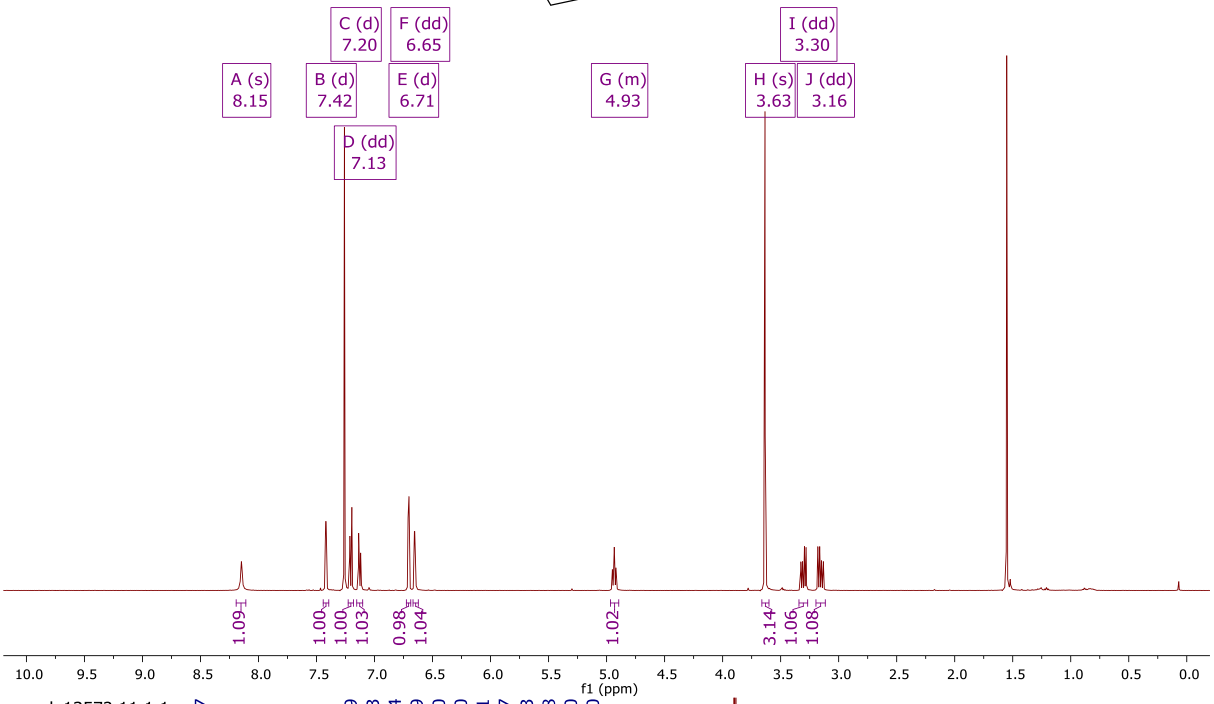

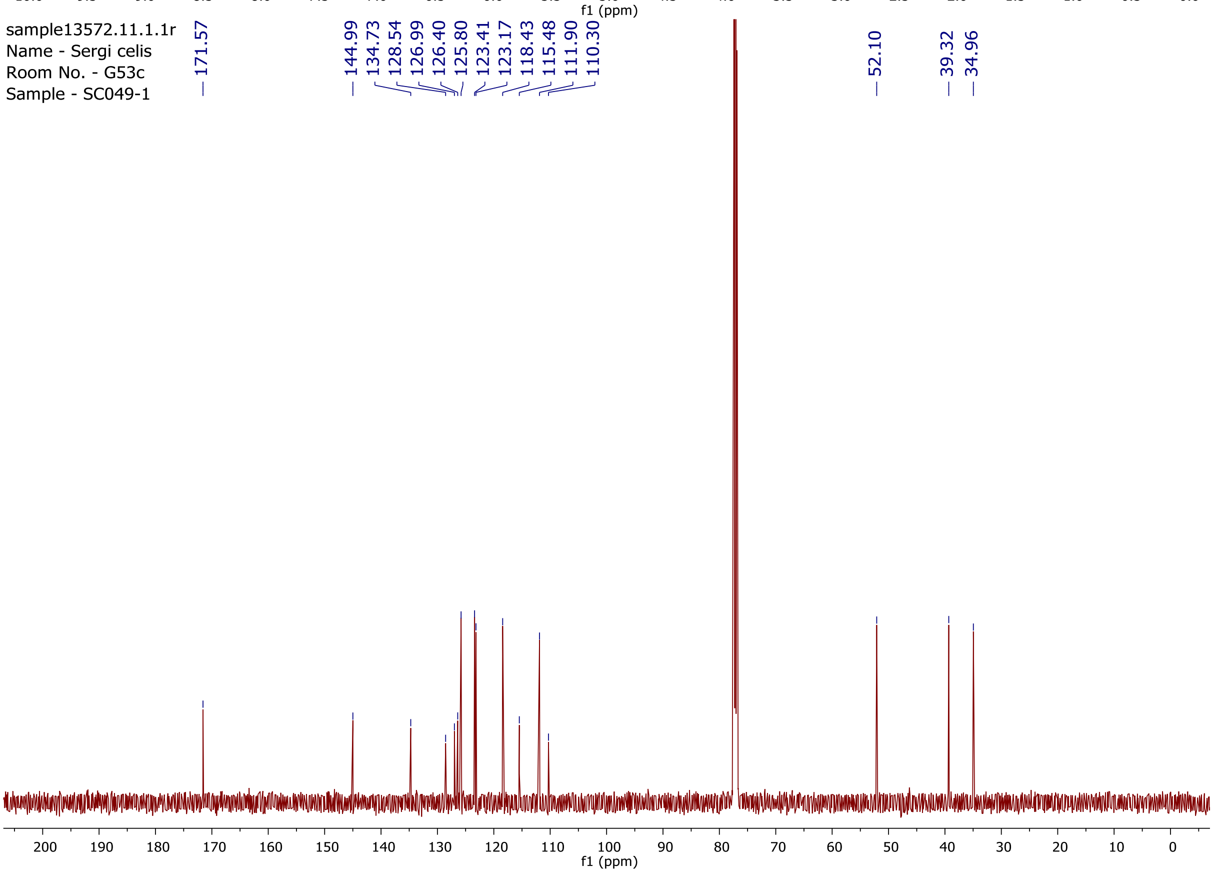

3-(2-Bromo-5-chloro-1H-indol-3-yl)-3-(5-chlorothiophen-2-yl)propanoic acid

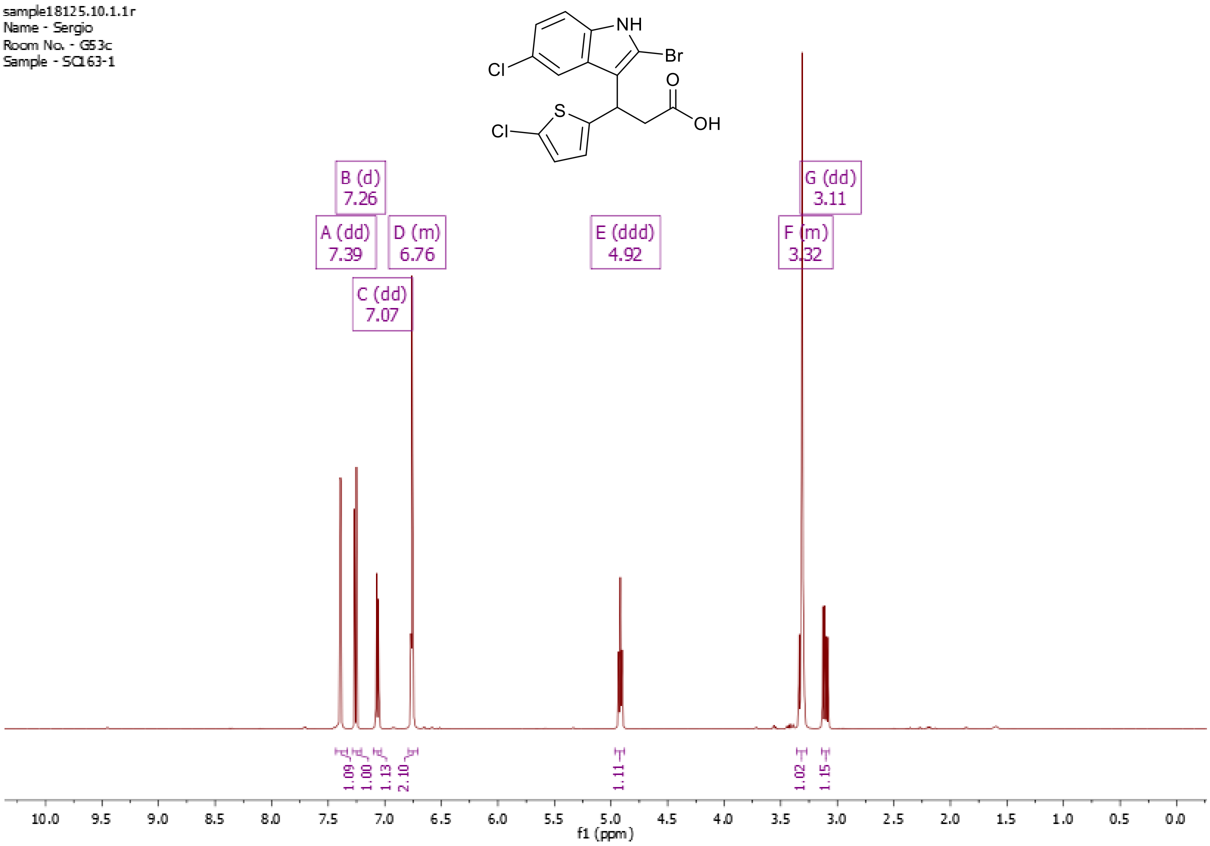

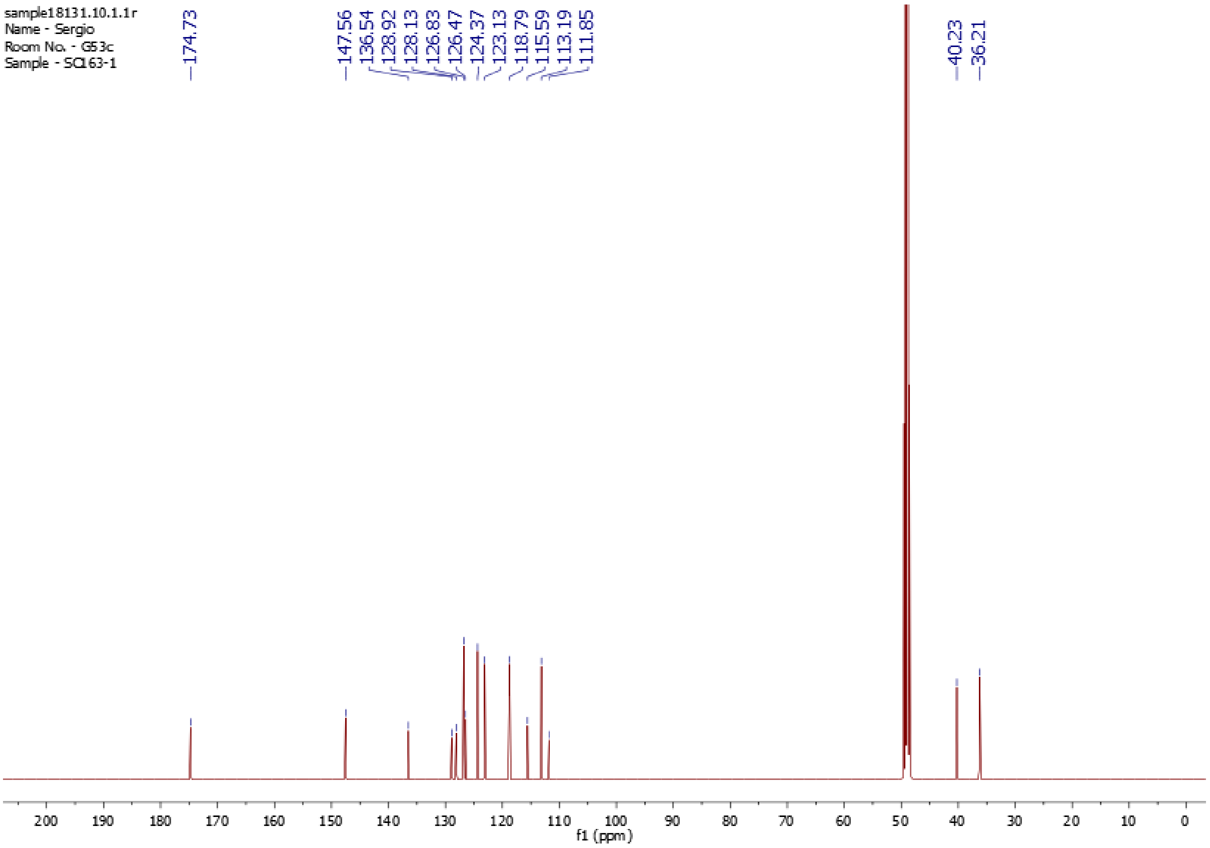

3-(2-Bromo-5-chloro-1H-indol-3-yl)-3-(5-chlorothiophen-2-yl)-*N*-[2-(pyridin-2-yl)ethyl]propanamide

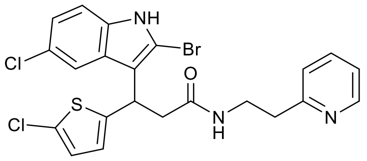

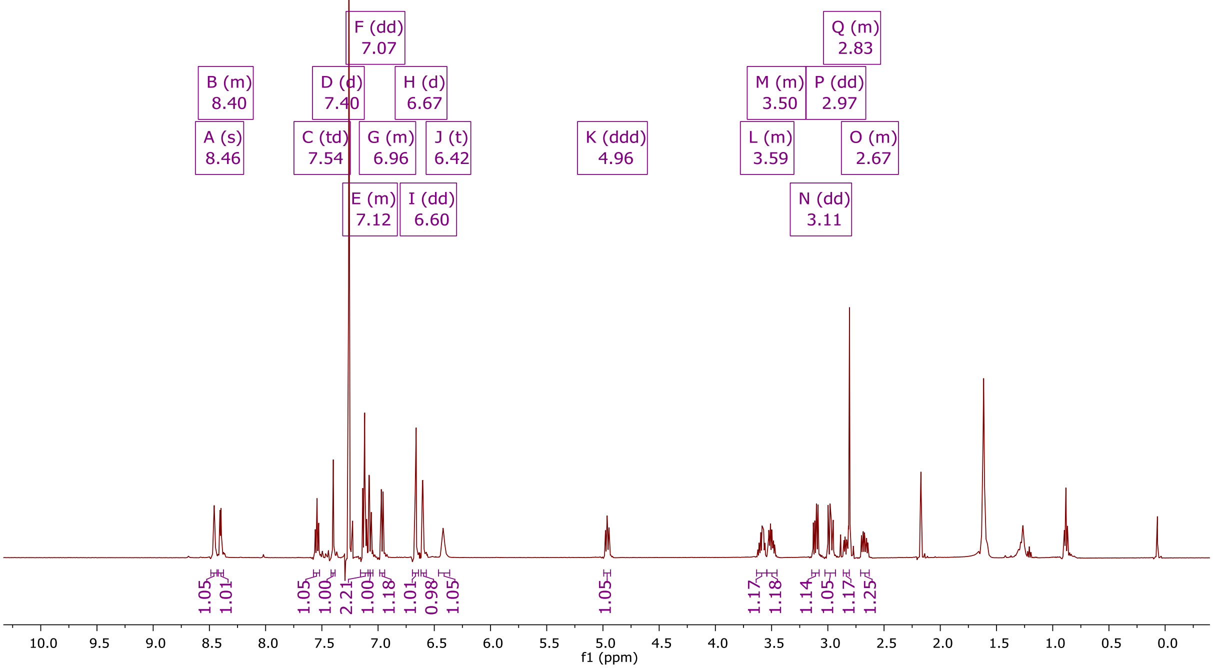

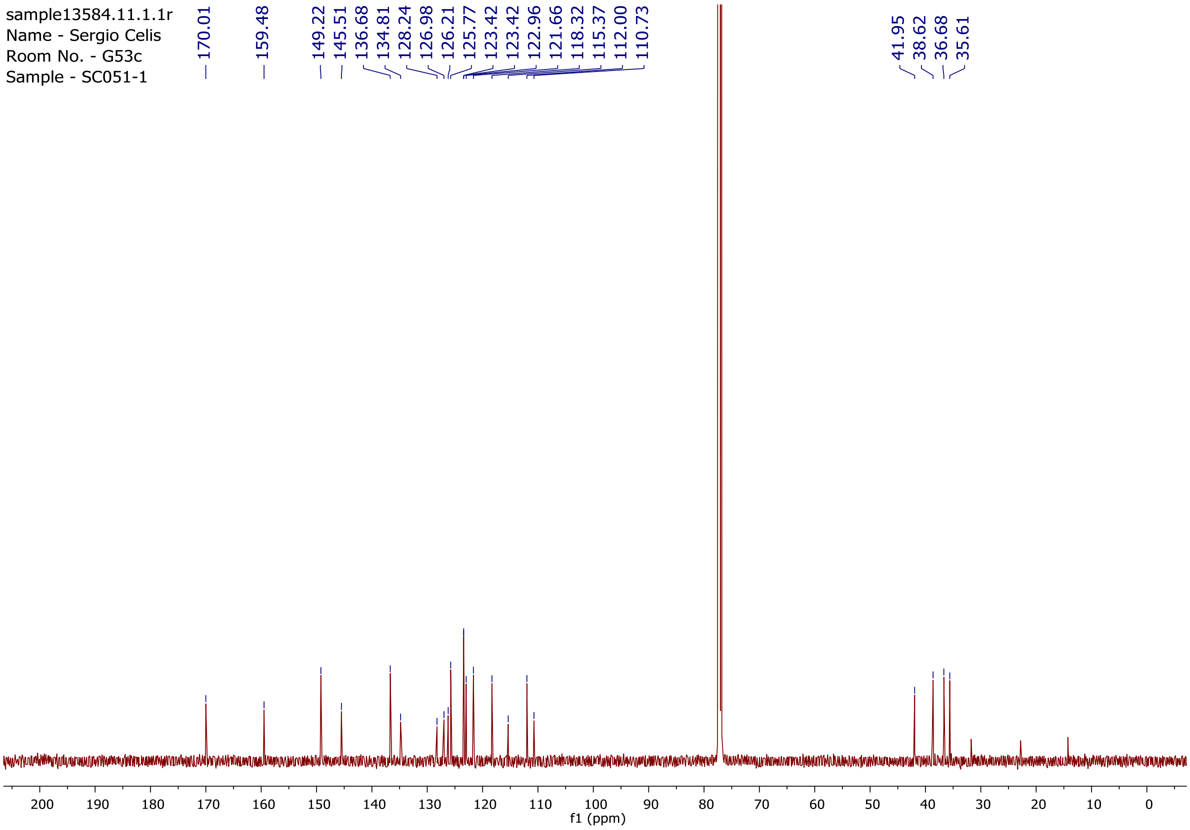

N-Benzyl-5-chloro-3-[(5-chlorothiophene-2-yl)(4-methylbenzenesulfonyl) methyl]-1H-indole-2-carboxamide

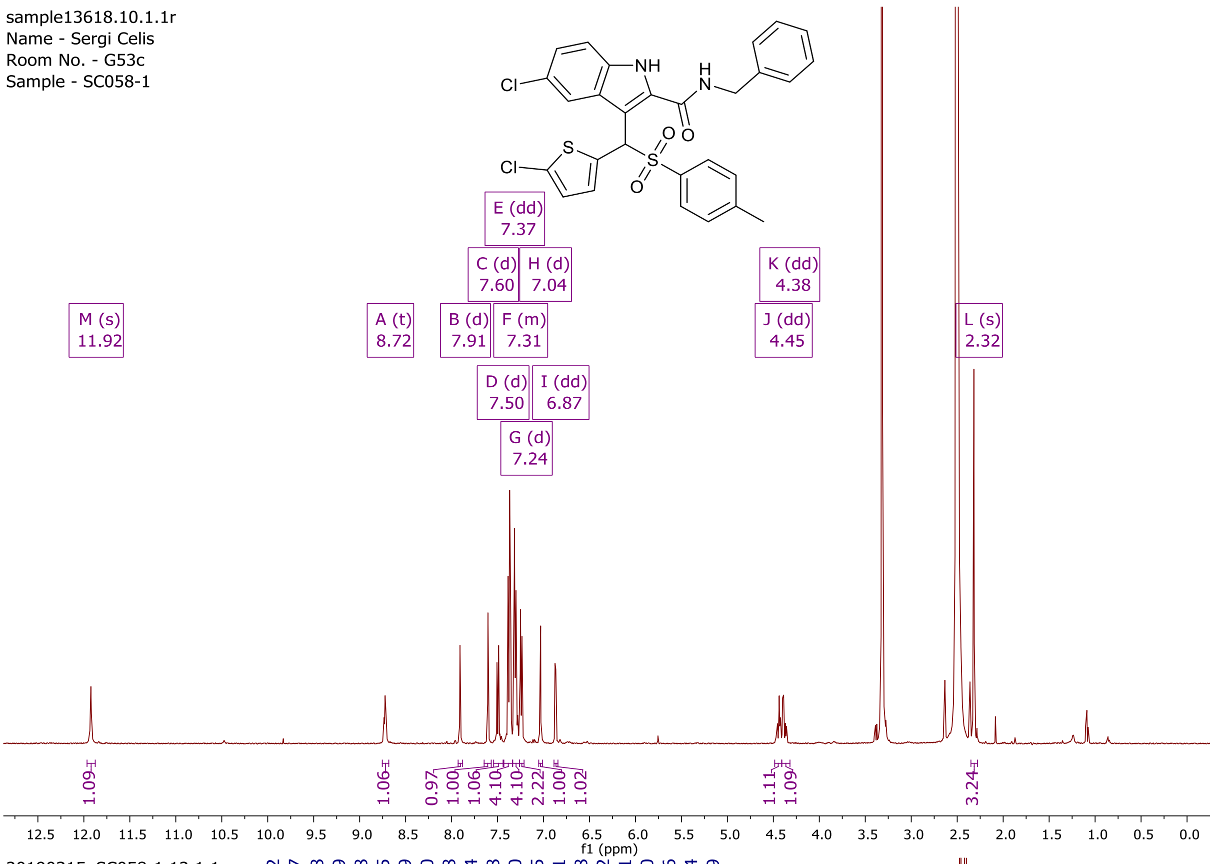

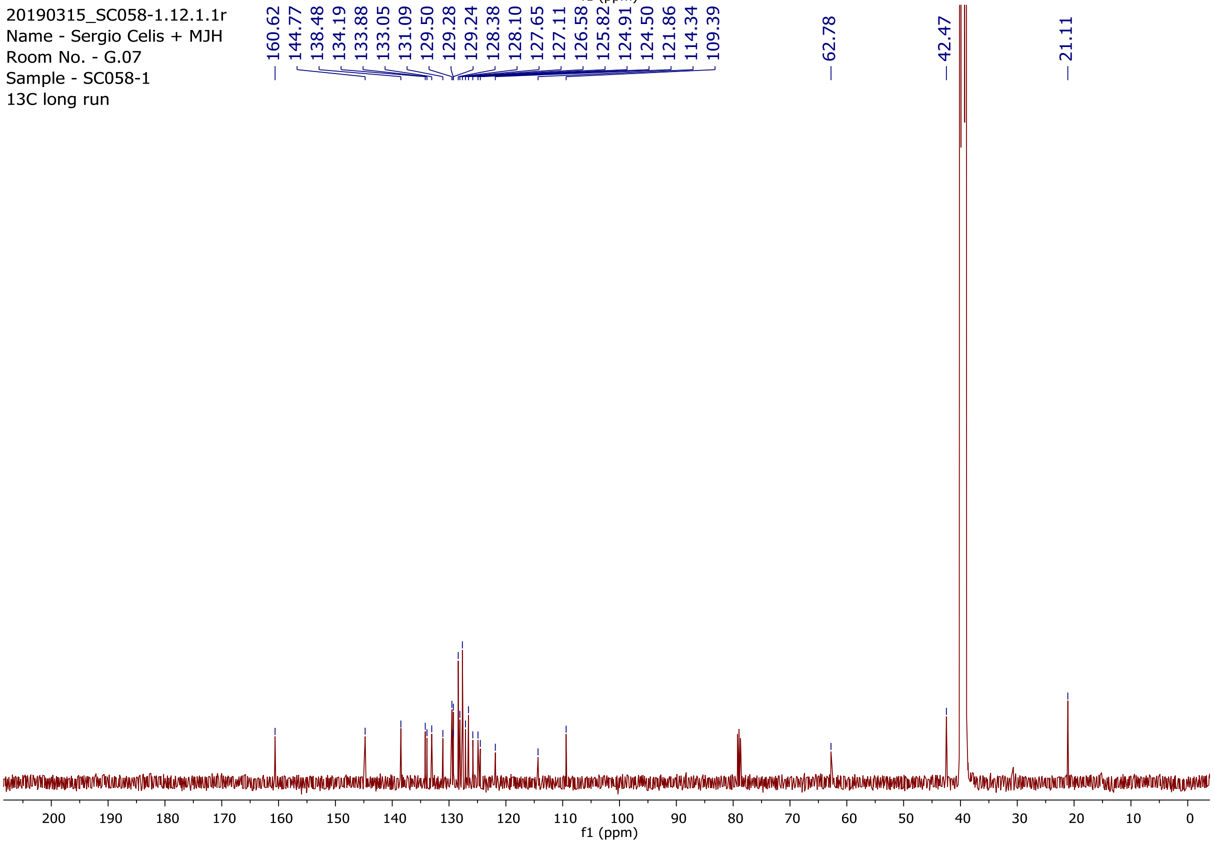

Methyl 3-[2-(benzylcarbamoyl)-5-chloro-1H-indol-3-yl]-3-(5-chlorothiophen-2-yl) propanoate

Name - Sergio
Room No. - G53c
Sample - SC061-3

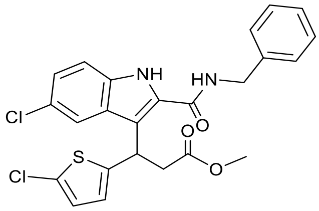

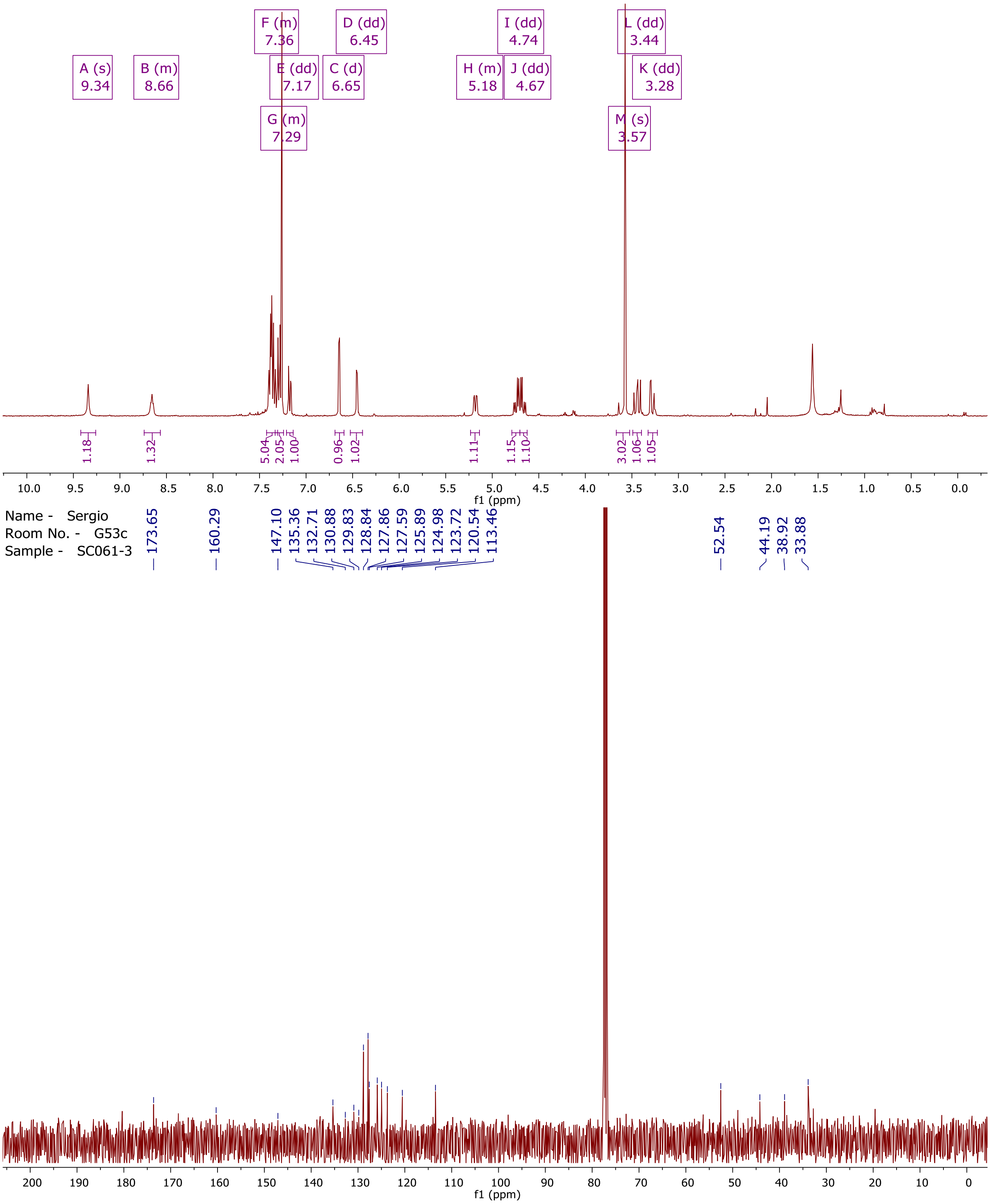

3-[2-(Benzylcarbamoyl)-5-chloro-1H-indol-3-yl]-3-(5-chlorothiophen-2-yl) propanoic acid

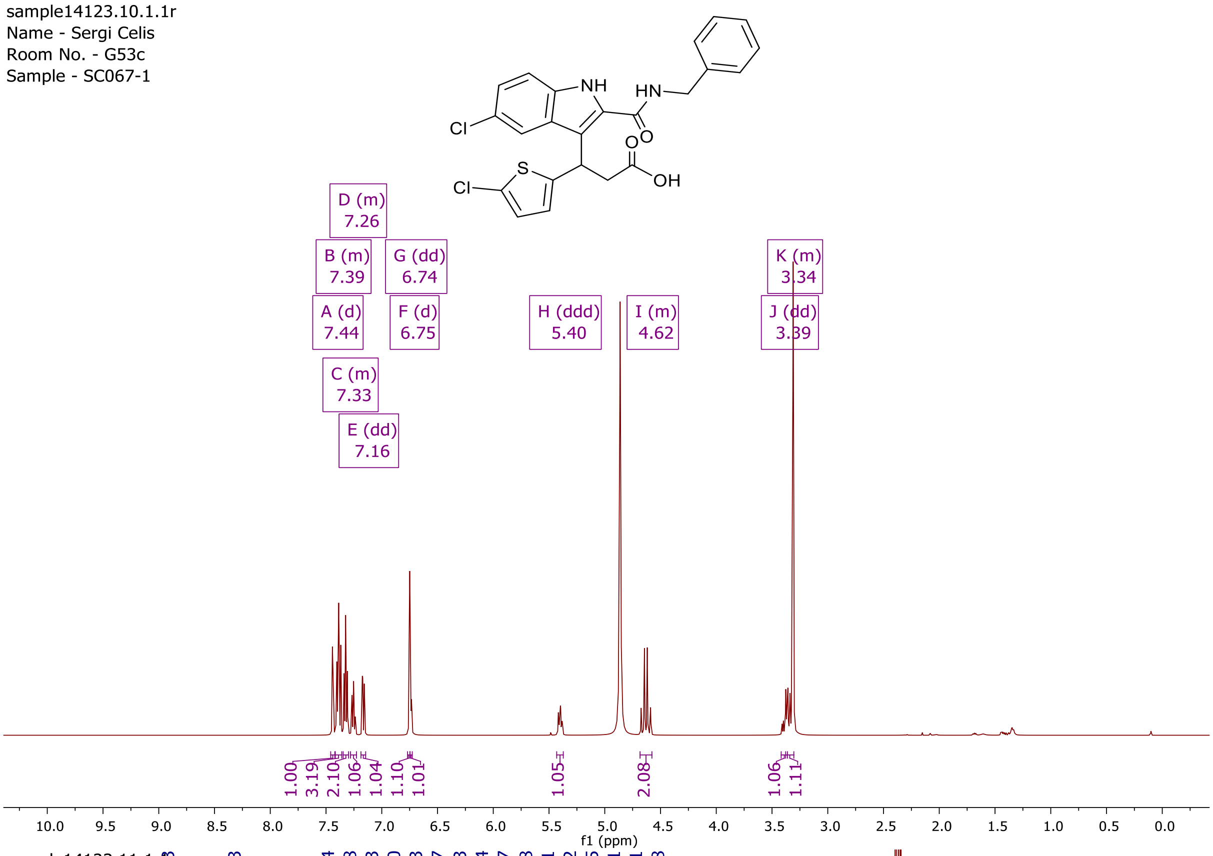

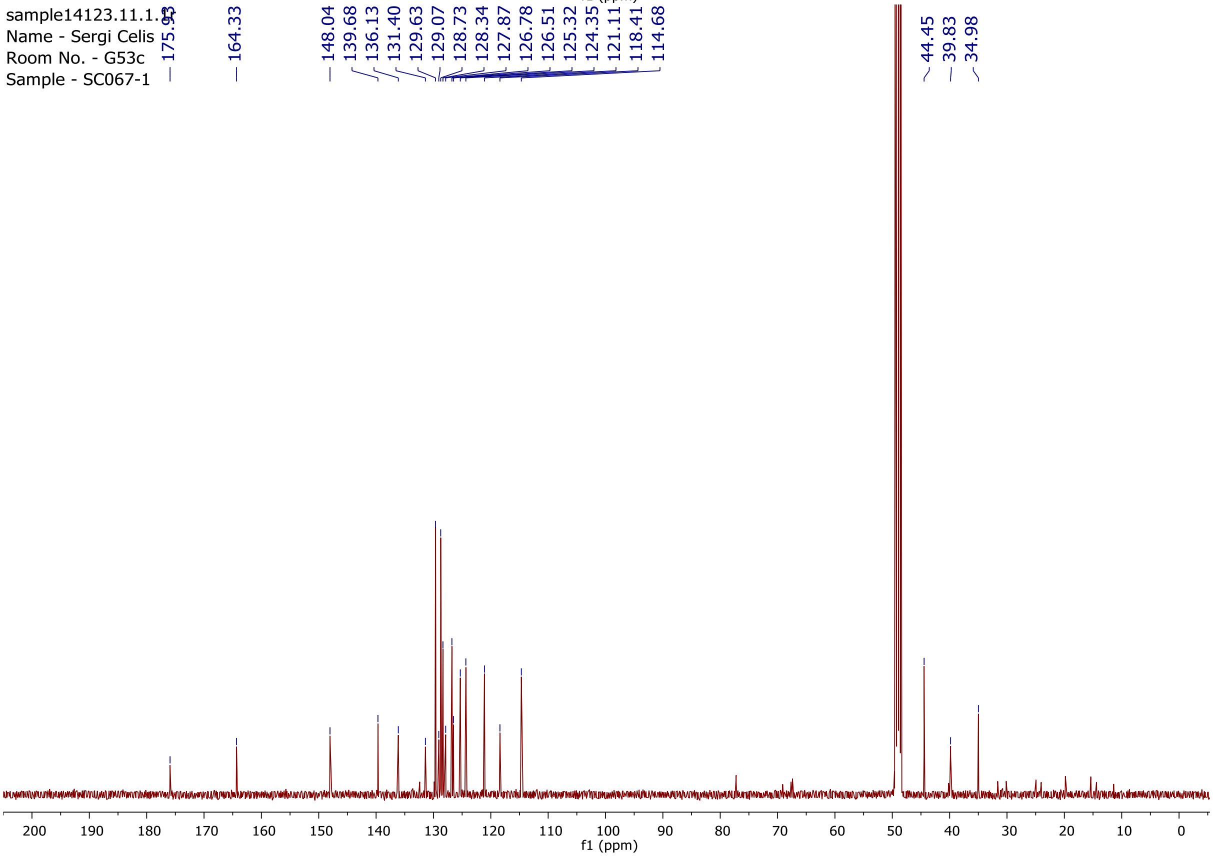

N-Benzyl-5-chloro-3-[1-(5-chlorothiophene-2-yl)-2-{[2-(pyridin-2-yl)ethyl] carbamoyl}ethyl]-1H-indole-2-carboxamide

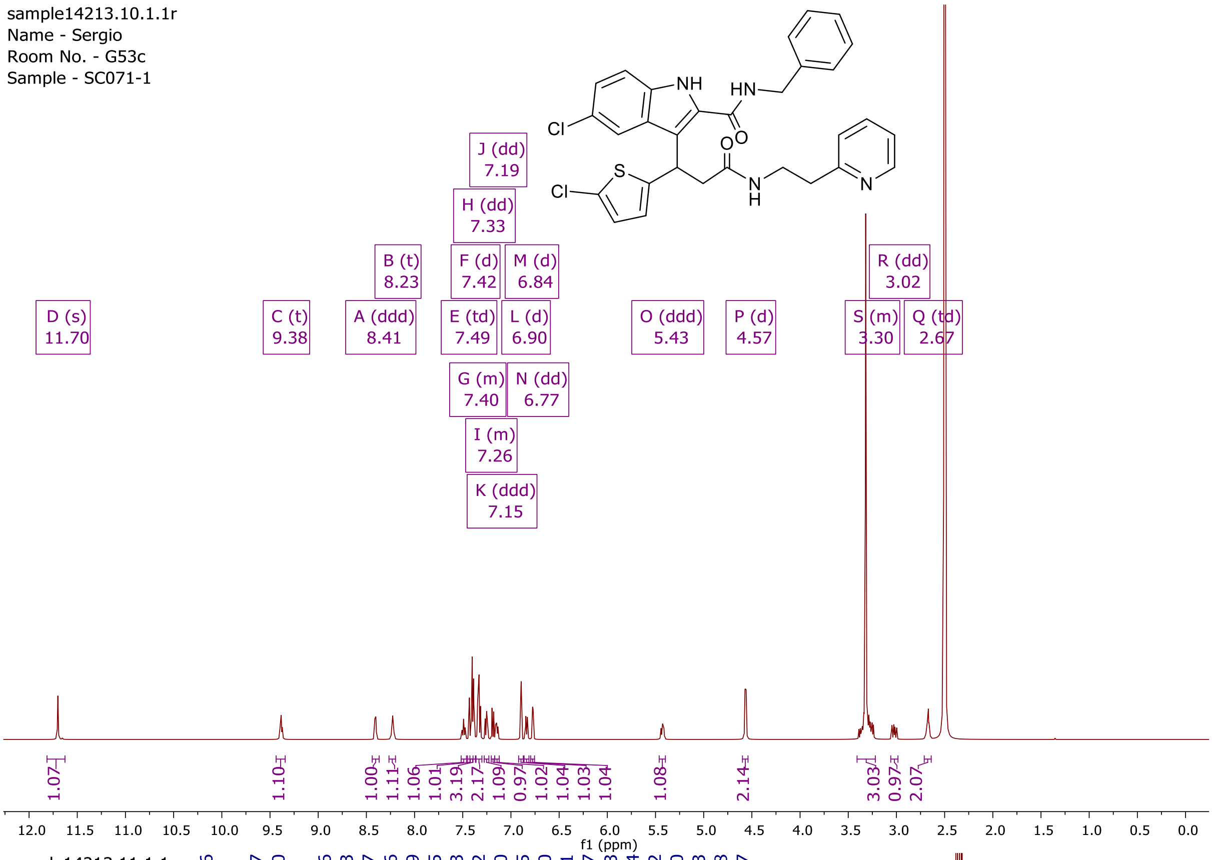

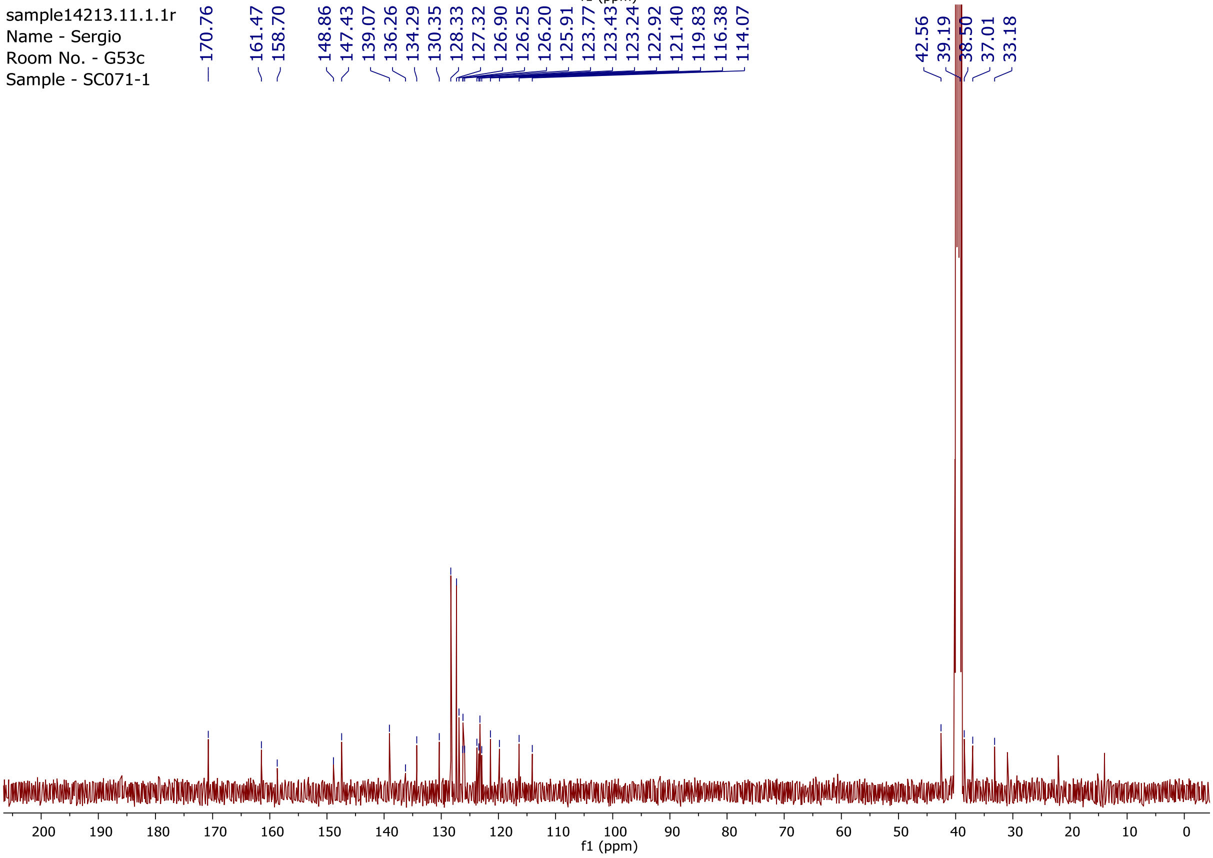

5-Chloro-3-[(5-chlorothiophene-2-yl)(4-methylbenzenesulfonyl)methyl]-*N*-(2-methylpropyl)-1H-indole-2-carboxamide

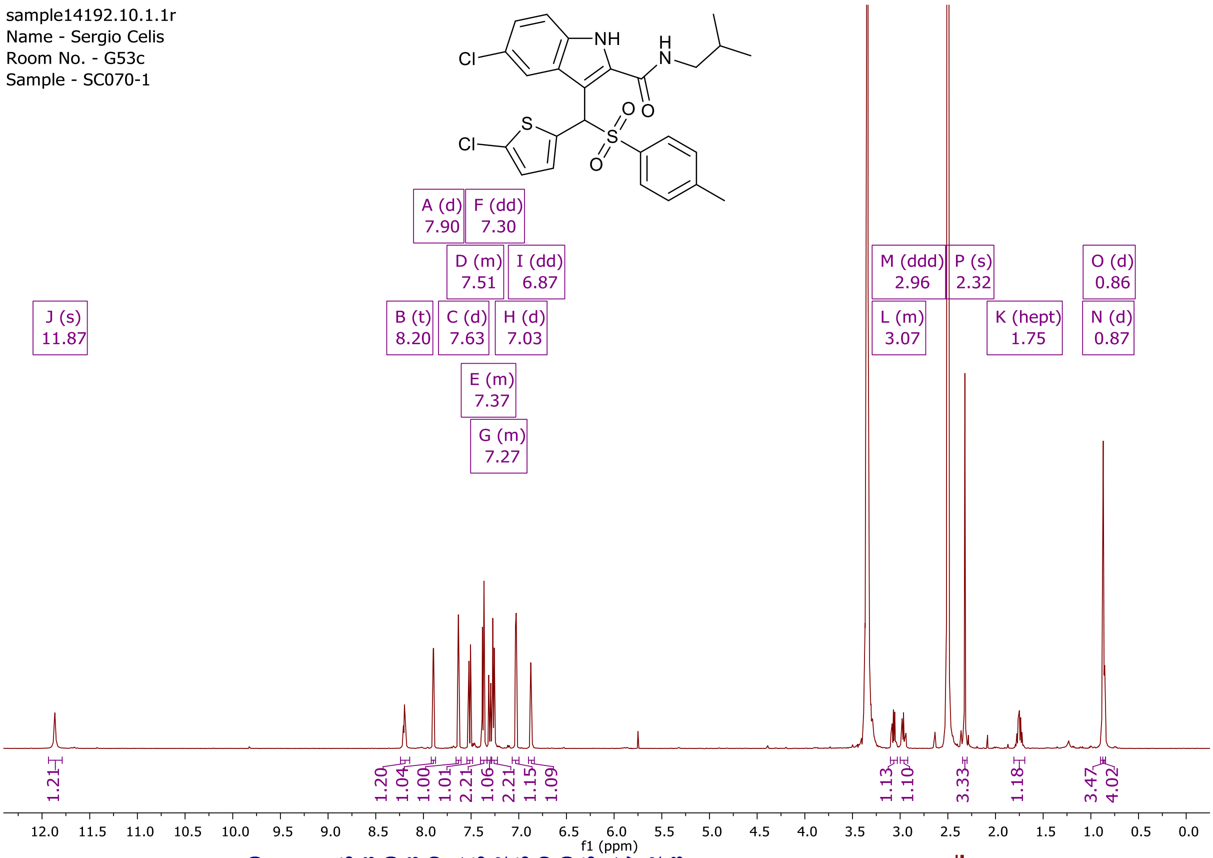

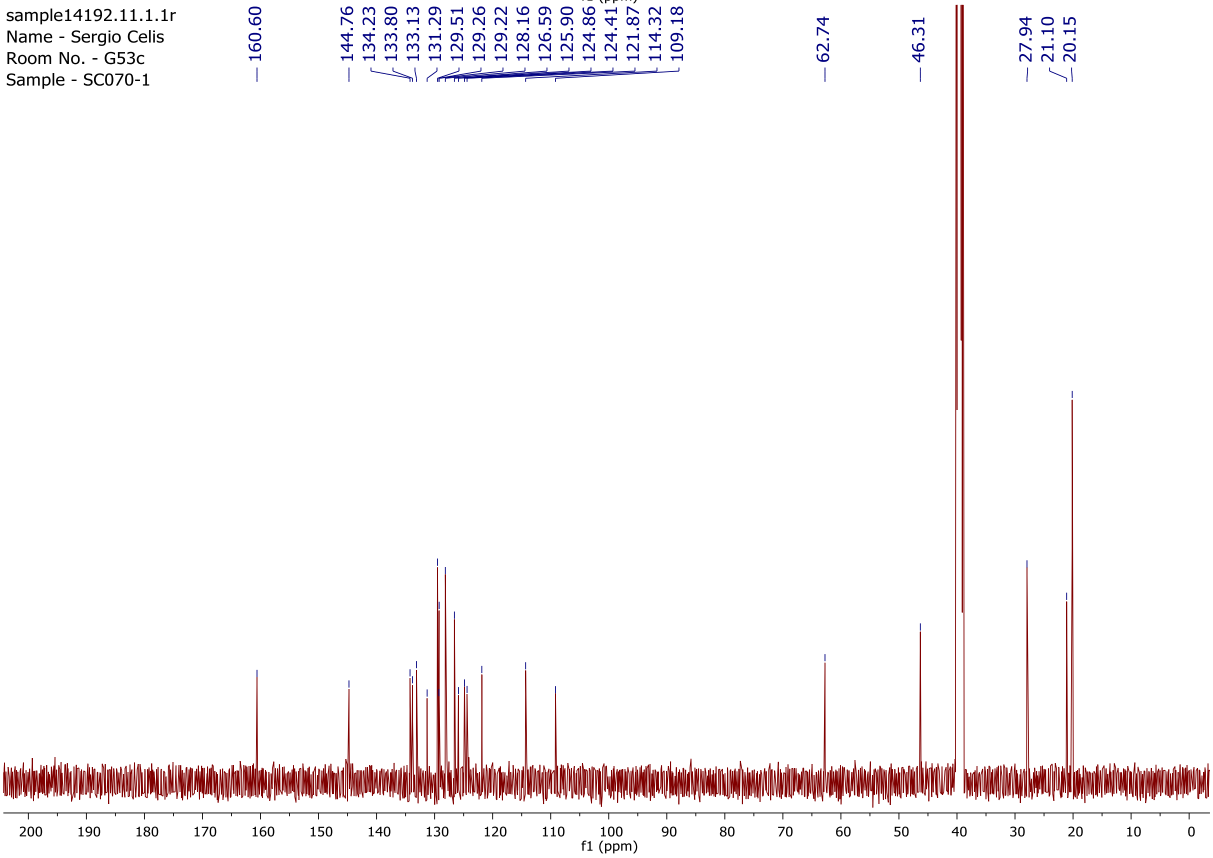

Methyl 3-{5-chloro-2-[(2-methylpropyl)carbamoyl]-1H-indol-3-yl}-3-(5-chlorothiophen-2-yl)propanoate

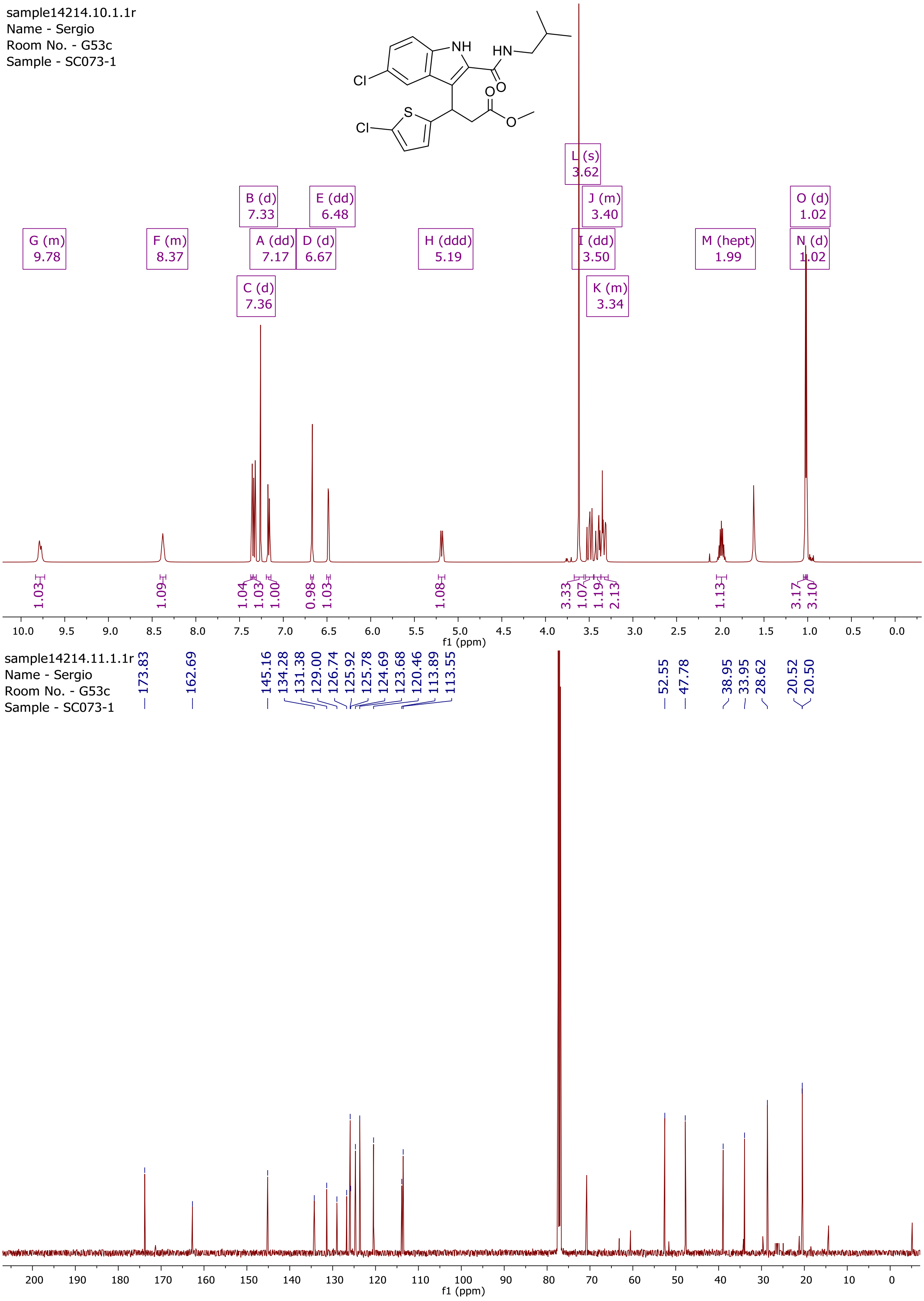

3-{5-Chloro-2-[(2-methylpropyl)carbamoyl]-1H-indol-3-yl}-3-(5-chlorothiophen-2-yl)propanoic acid

sample14336.10.1.1r
Name - Sergio
Room No. - G53c
Sample - SC074-1

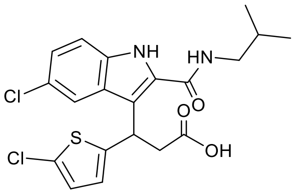

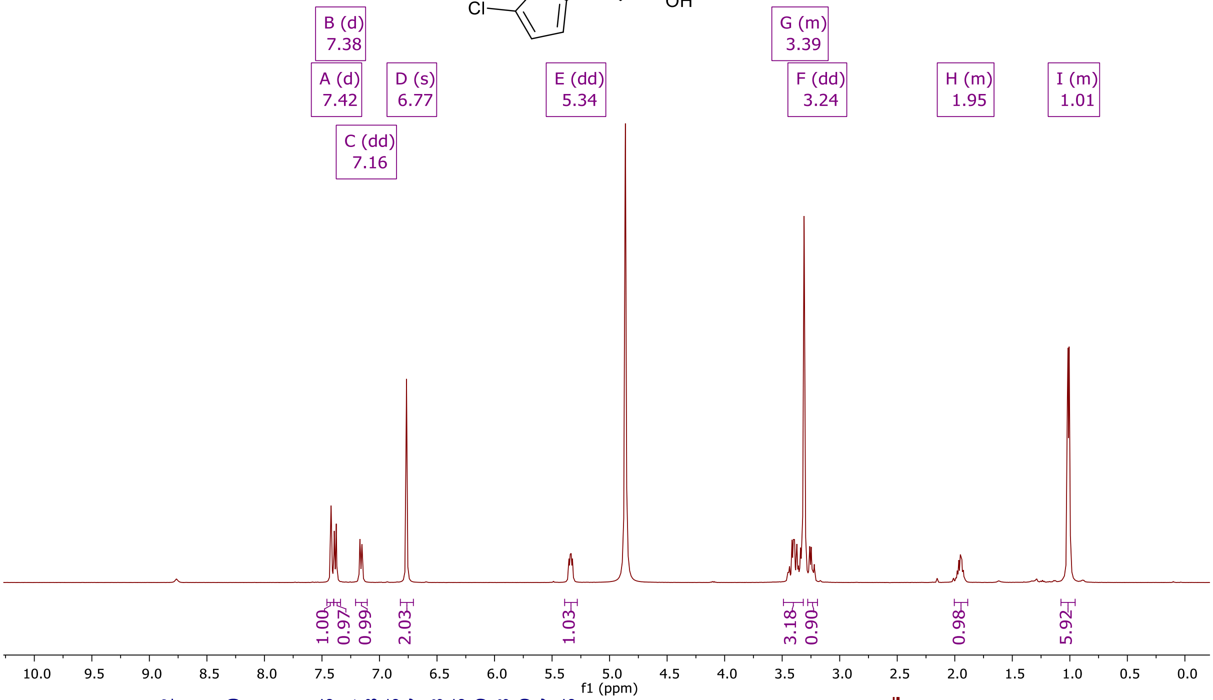

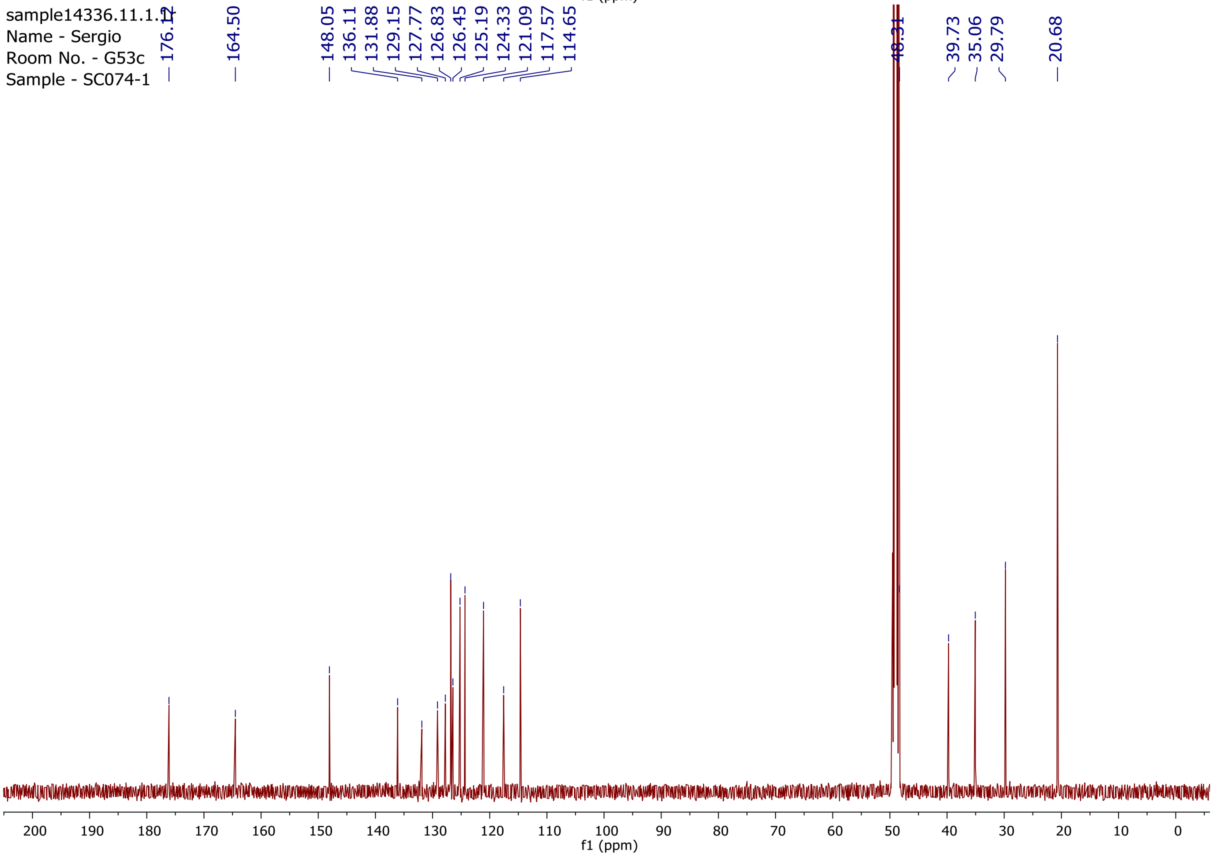

5-Chloro-3-[1-(5-chlorothiophene-2-yl)-2-{[2-pyridin-2-yl)ethyl]carbamoyl}ethyl]-*N*-(2-methylpropyl)-1H-indole-2-carboxamide

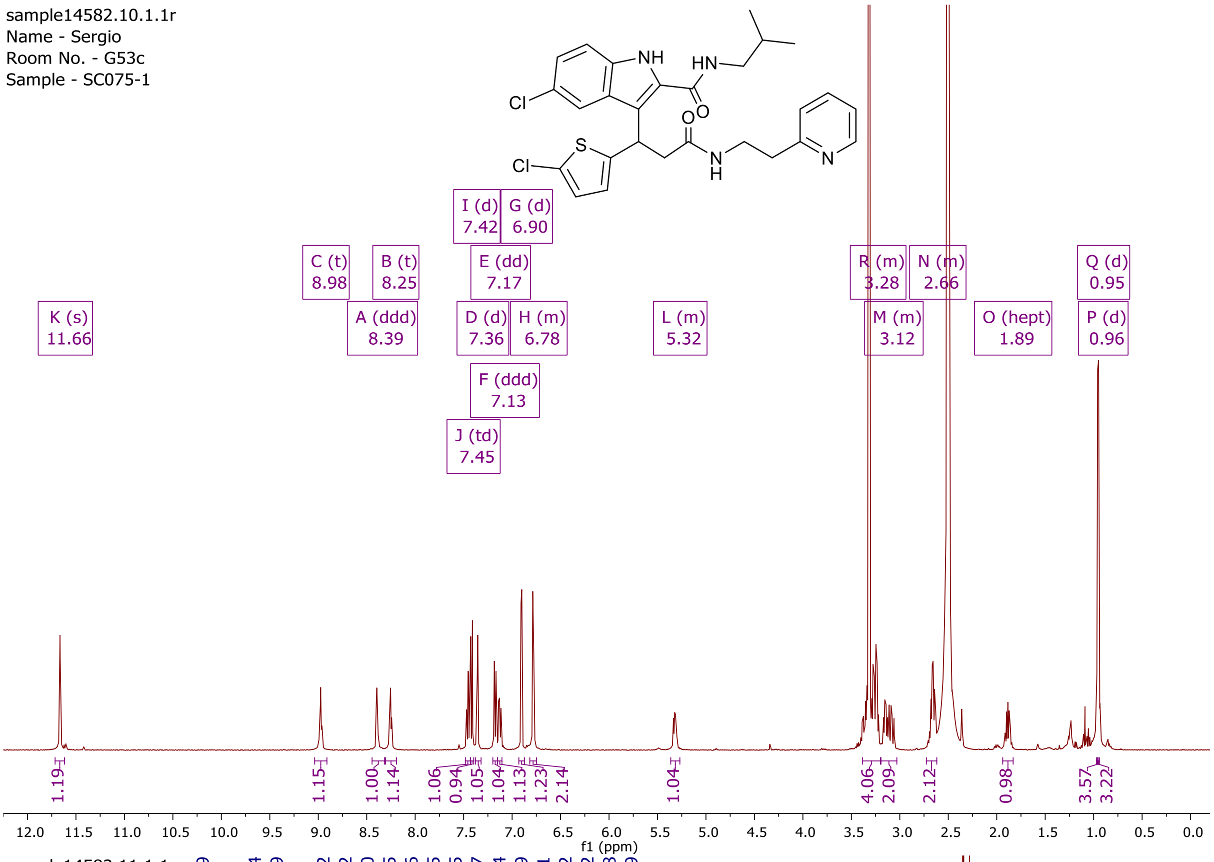

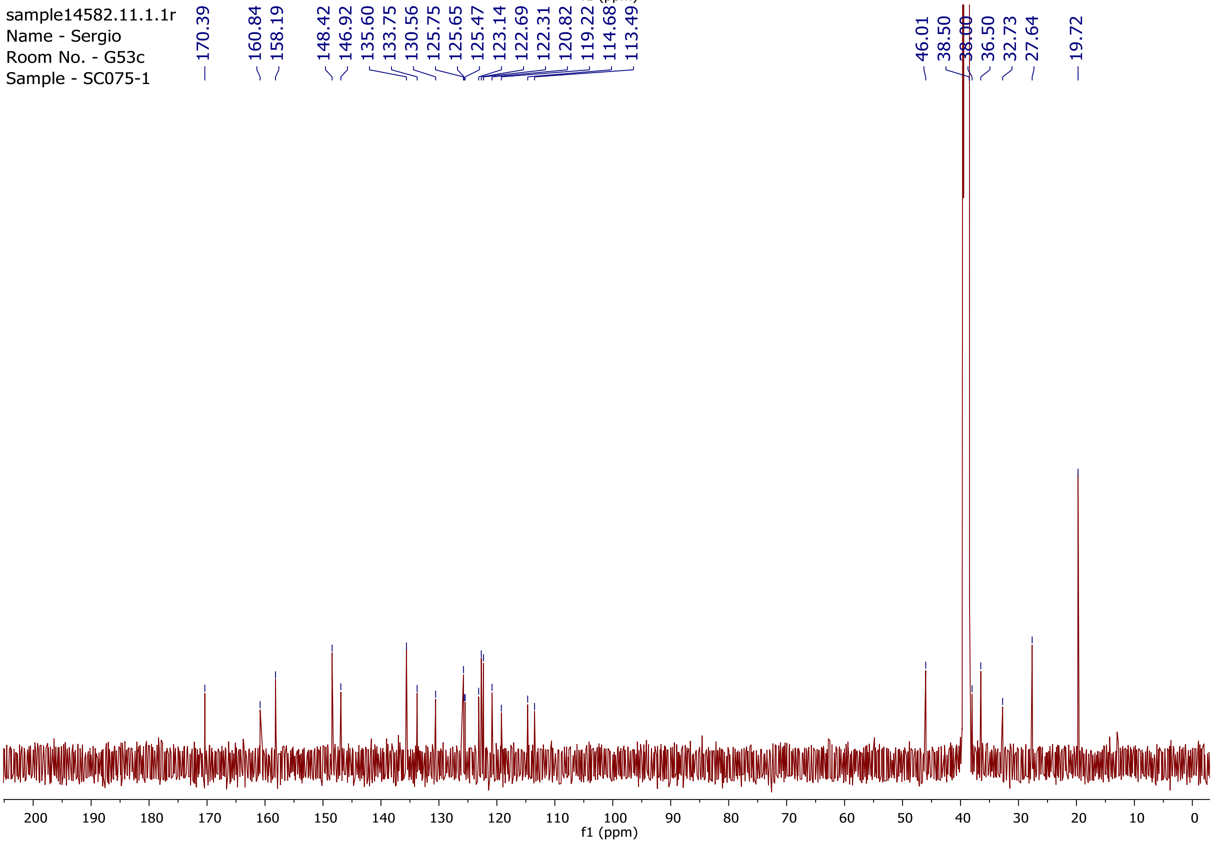

***tert*-Butyl *N*-{2-[(5-chloro-1H-indol-2-yl)formamido]ethyl}carbamate**

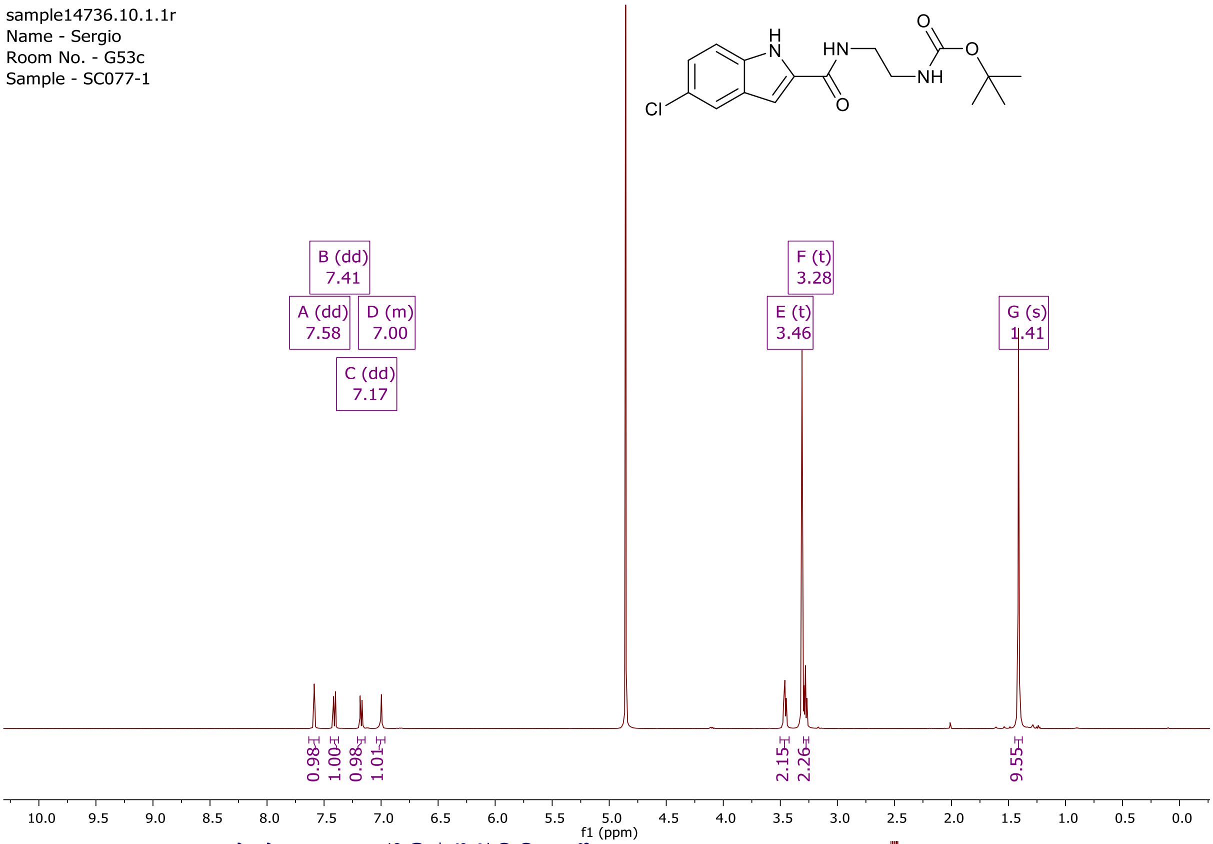

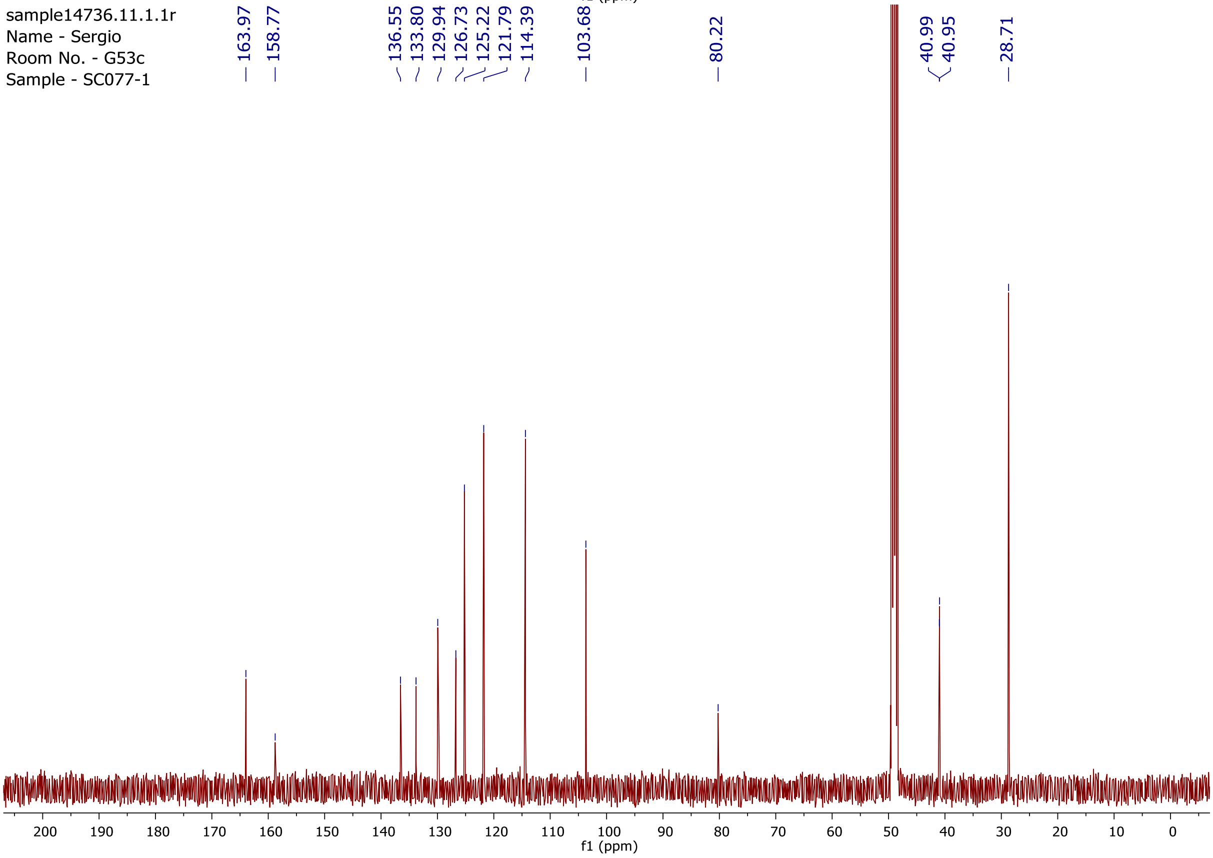

5-Chloro-3-[(5-chlorothiophene-2-yl)(4-methylbenzenesulfonyl)methyl]-1H-indole-2-carboxylic acid

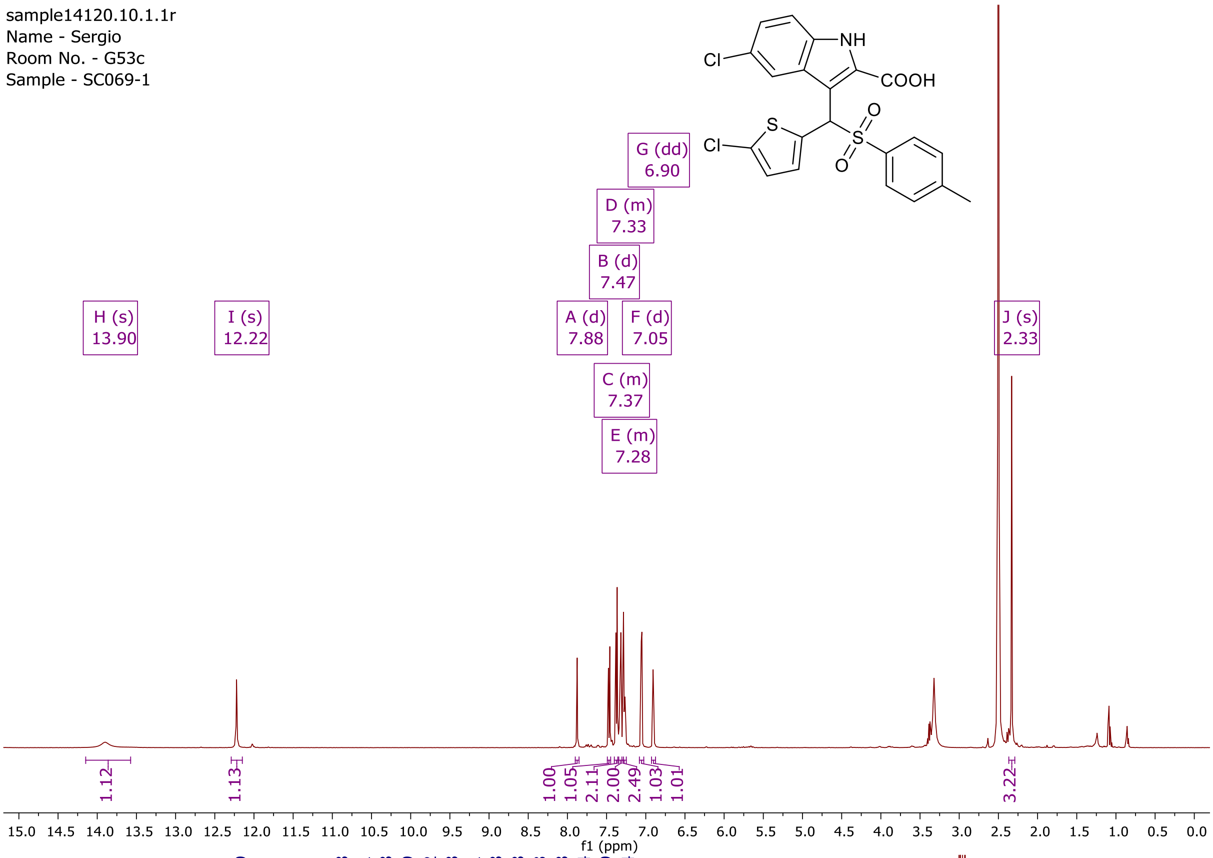

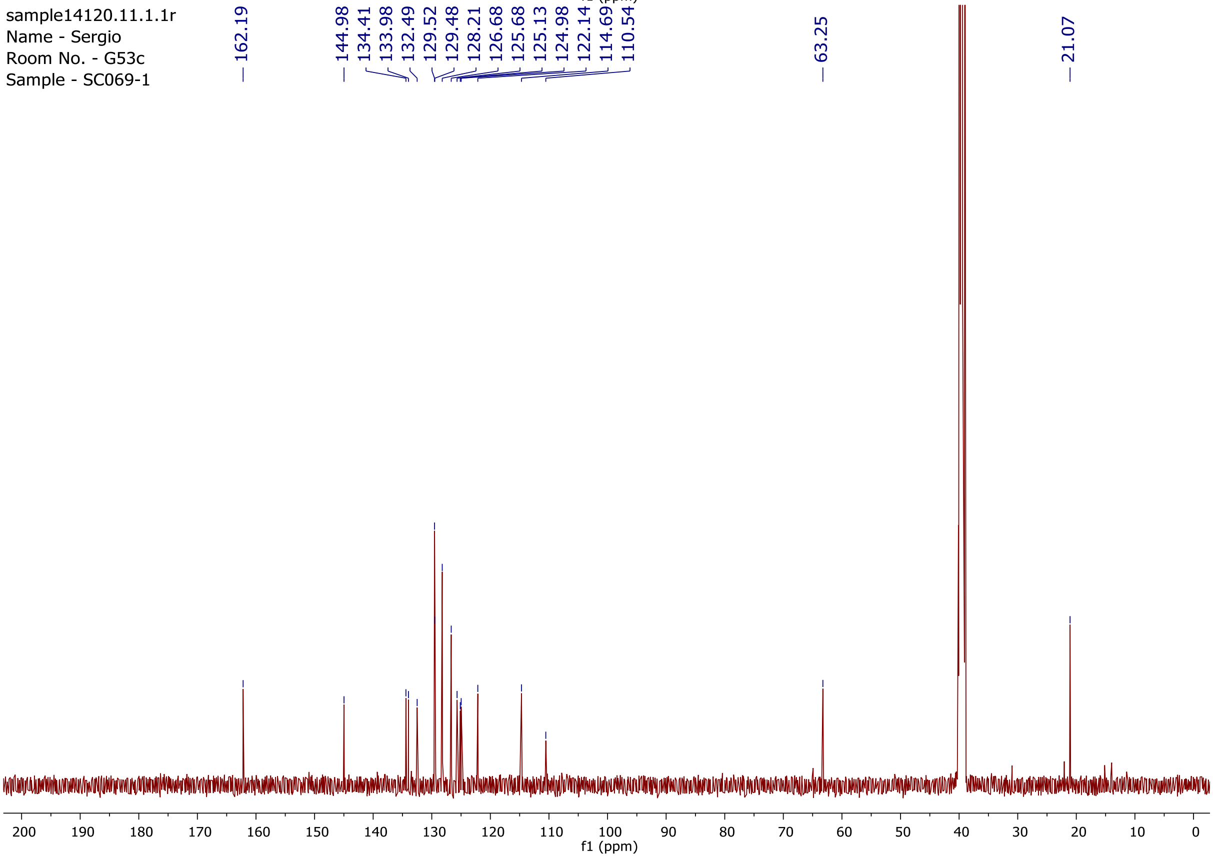

5-Chloro-3-[1-(5-chlorothiophene-2-yl)-3-methoxy-3-oxopropyl]-1H-indole-2-carboxylic acid

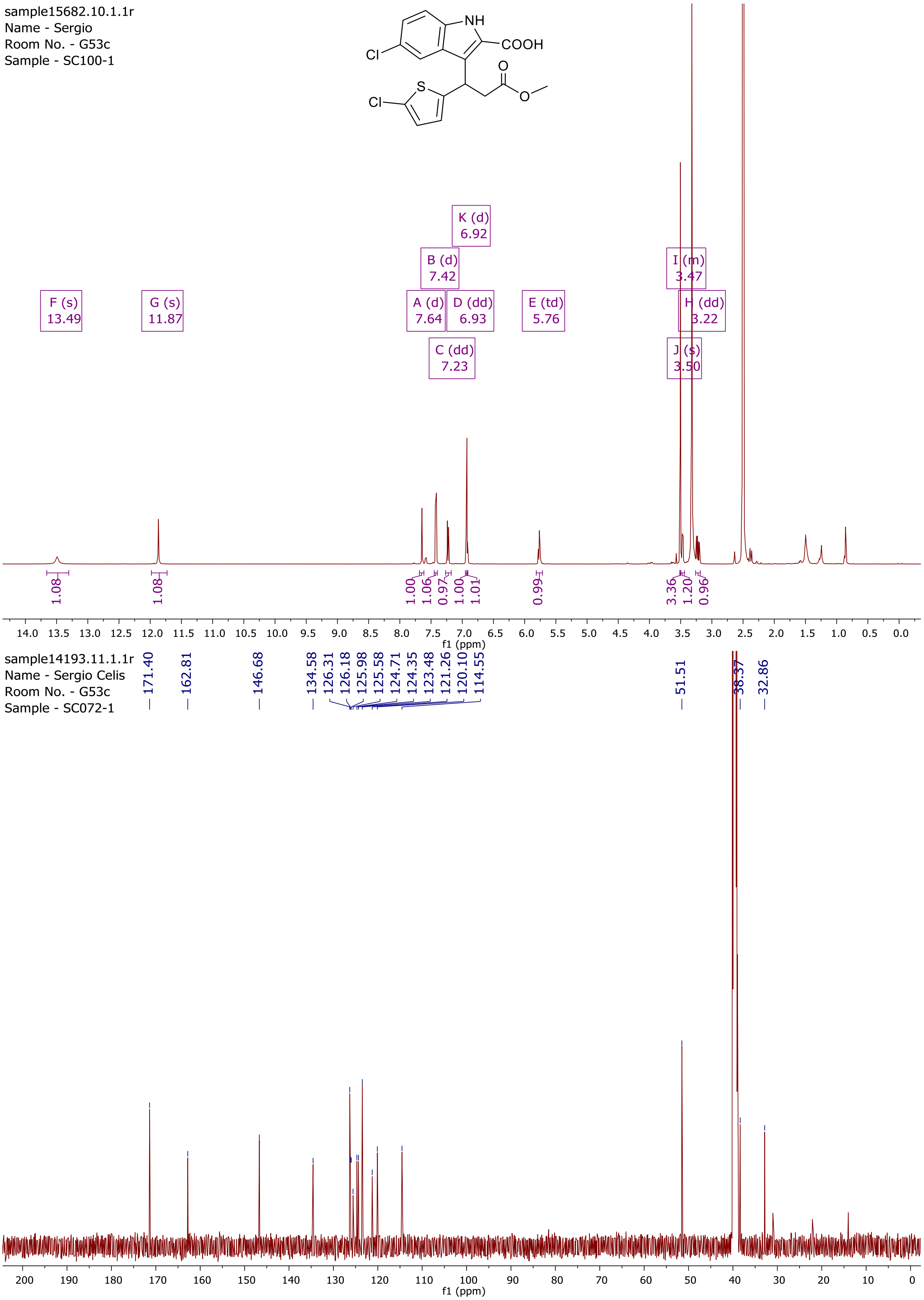

3-[2-Carboxy-1-(5-chlorothiophen-2-yl)ethyl]-5-chloro-1H-indole-2-carboxylic acid

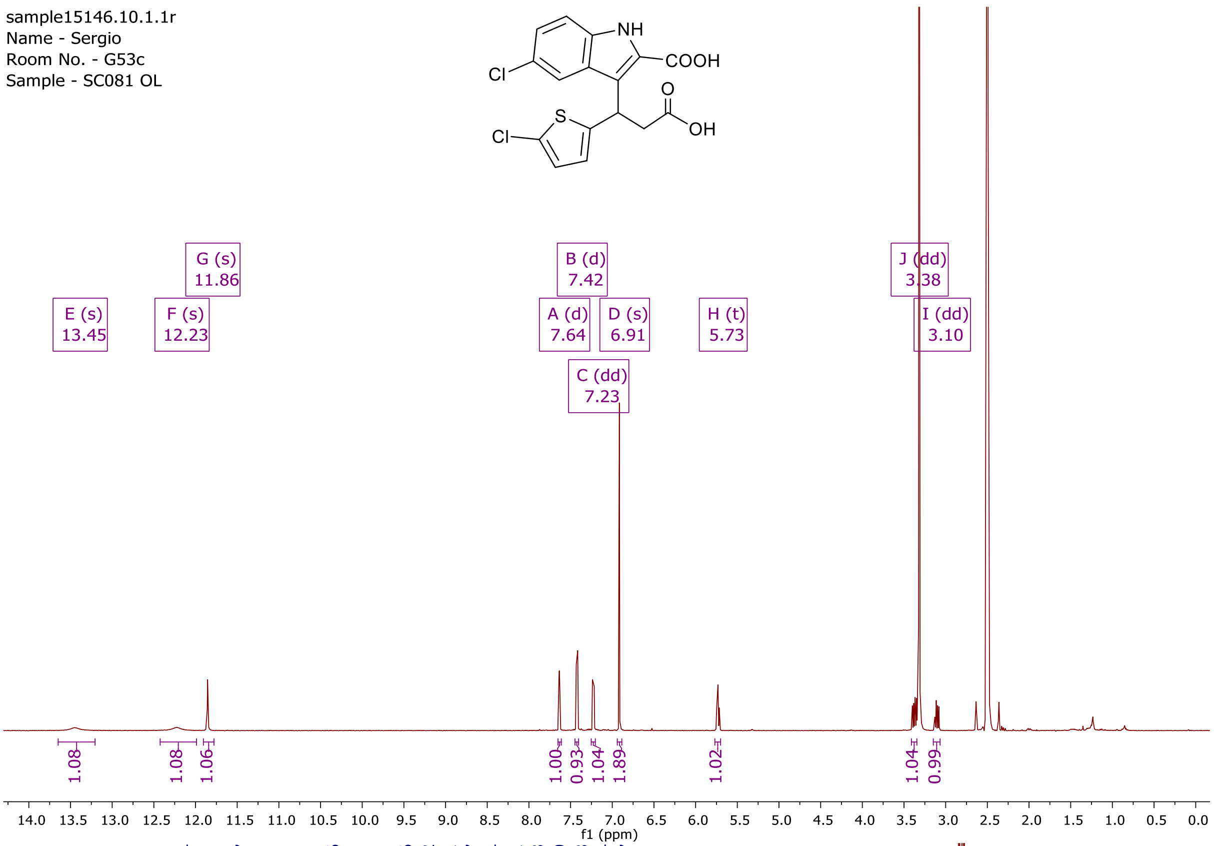

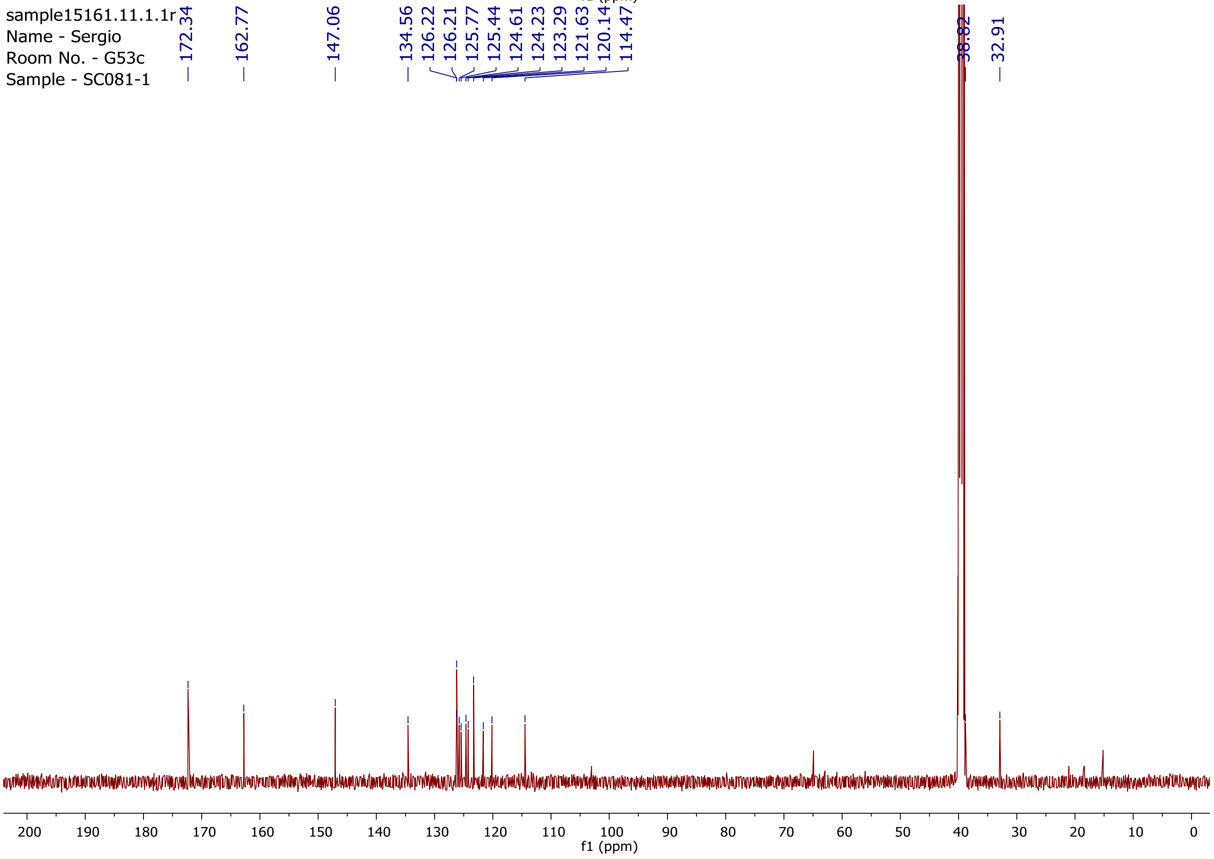

5-Chloro-3-[1-(5-chlorothiophen-2-yl)-2-{[2-(pyridin-2-yl)ethyl]carbamoyl}ethyl]-1H-indole-2-carboxylic acid

sample15999.10.1.1r
Name - Sergio
Room No. - G53c
Sample - SC104-3

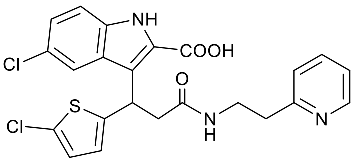

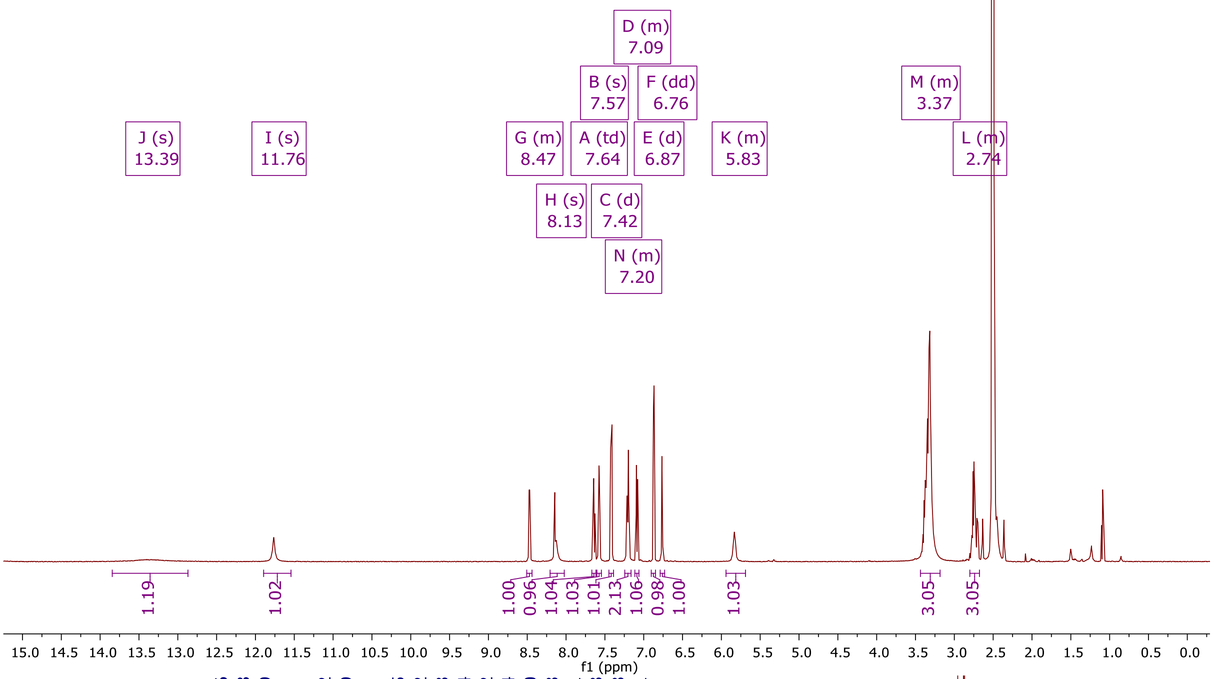

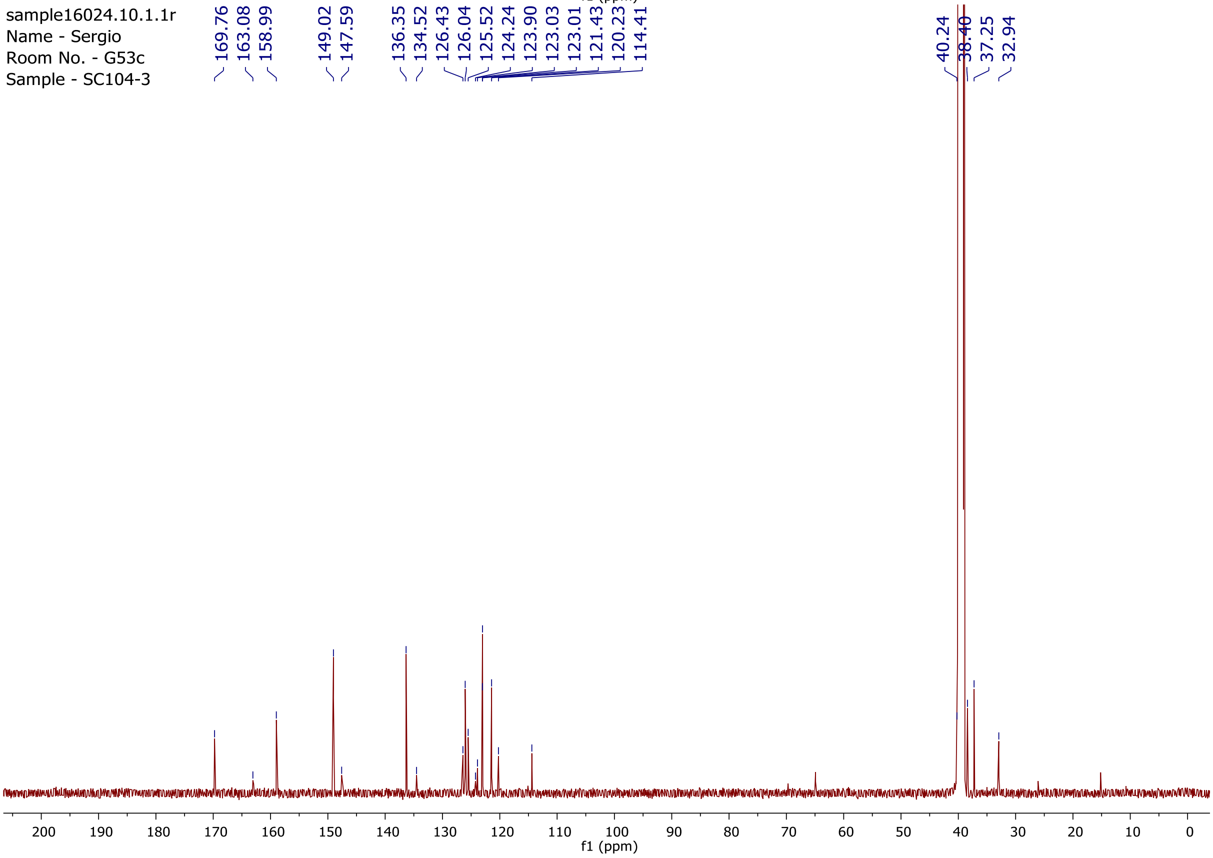

Methyl 5-chloro-3-[1-(5-chlorothiophen-2-yl)-2-{[2-(pyridin-2-yl)ethyl] carbamoyl}ethyl]-1H-indole-2-carboxylate

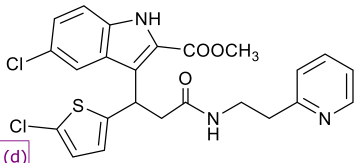

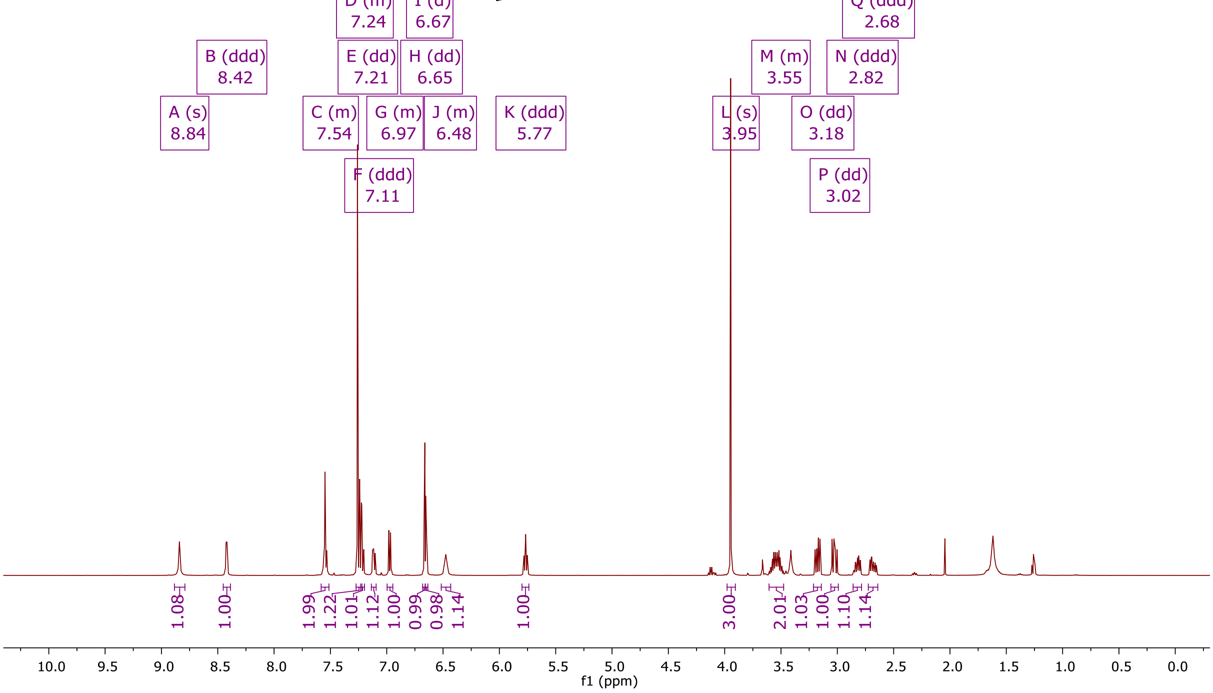

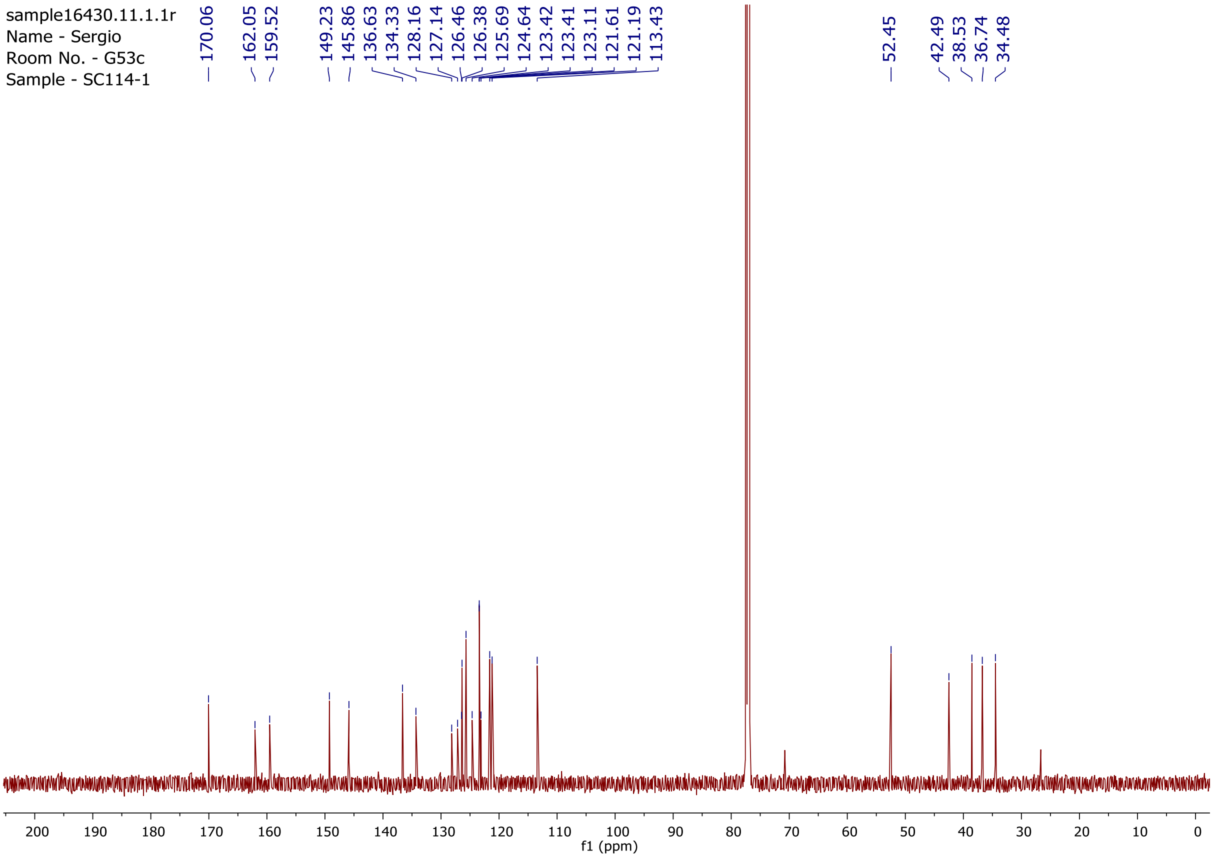

***tert*-Butyl *N*-[2-({5-chloro-3-[1-(5-chlorothiophen-2-yl)-2-{[2-(pyridin-2-yl) ethyl]carbamoyl}ethyl]-1H-indol-2-yl}formamido)ethyl]carbamate**

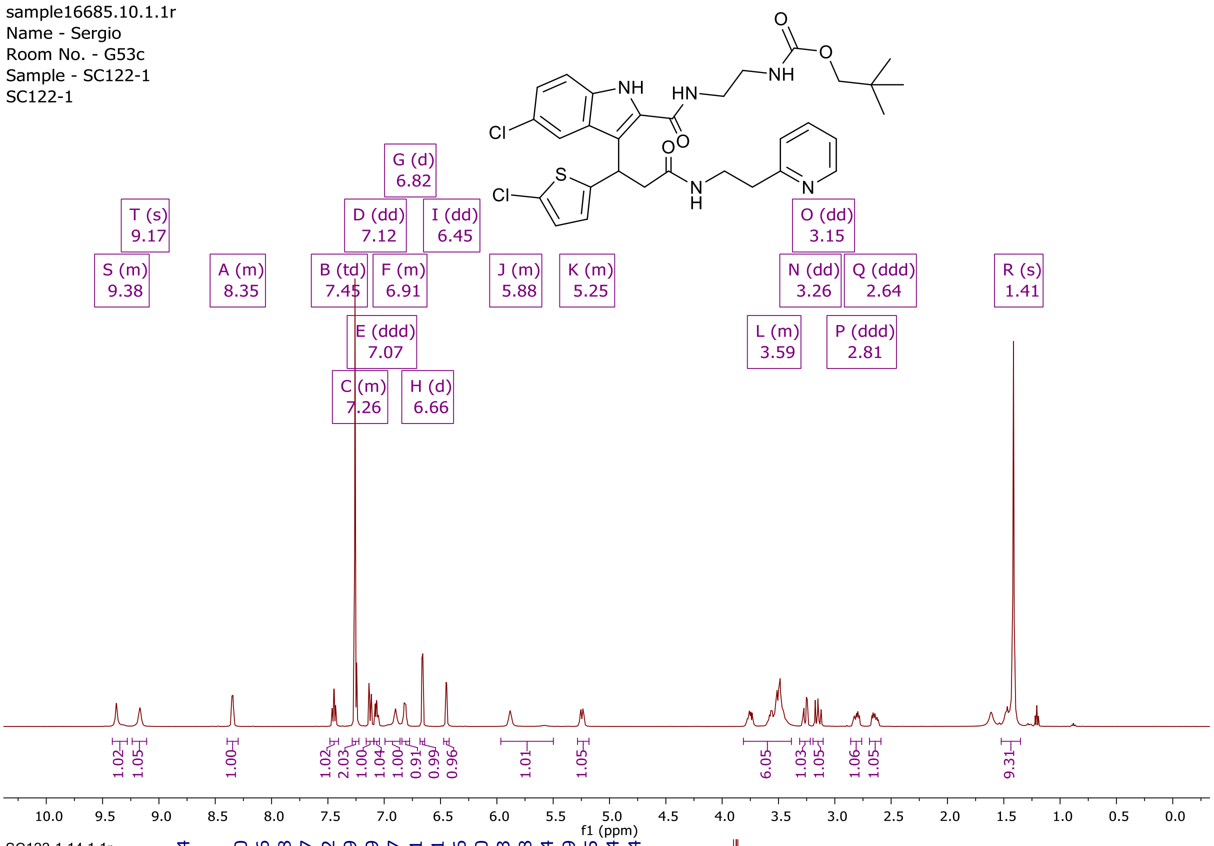

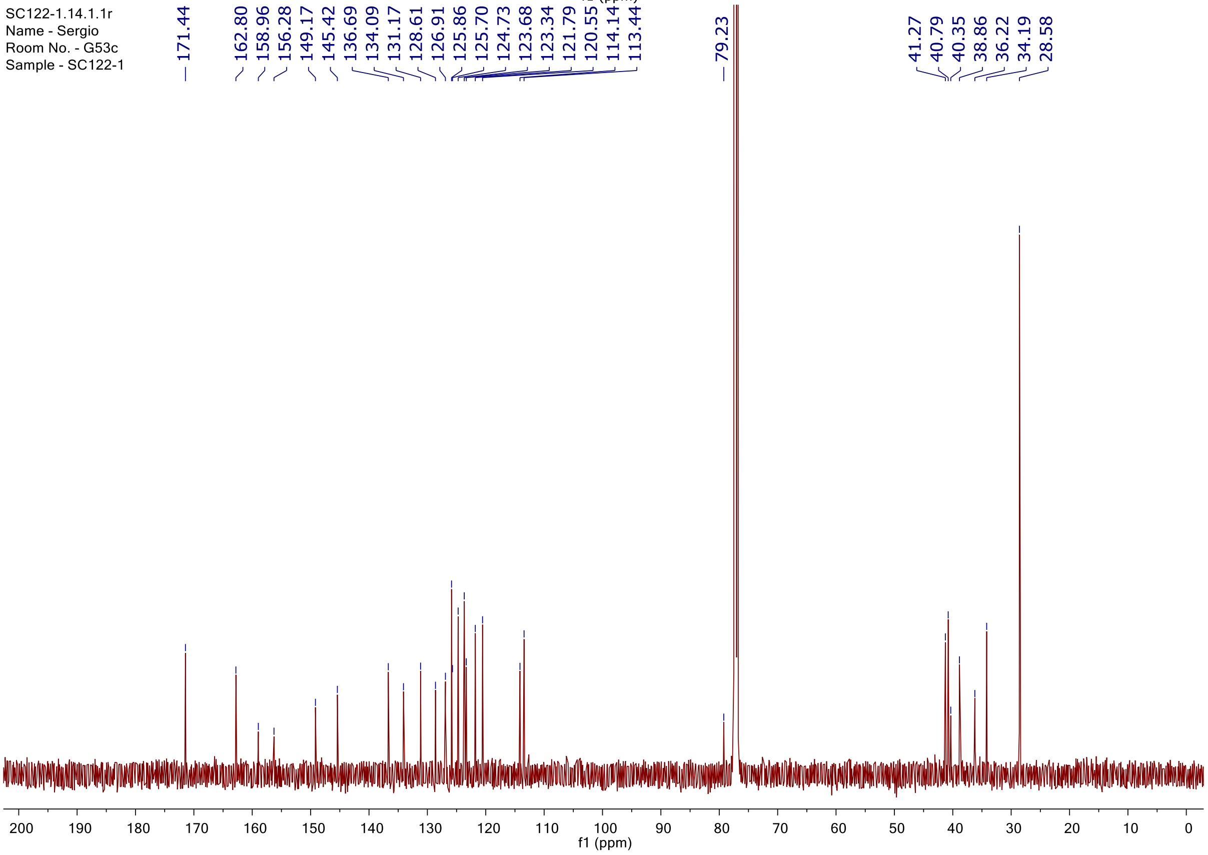

_N_-(2-Aminoethyl)-5-chloro-3-[1-(5-chlorothiophen-2-yl)-2-{[2-(pyridin-2-yl)ethyl]carbamoyl}ethyl]-1H-indole-2-carboxamide hydrochloride

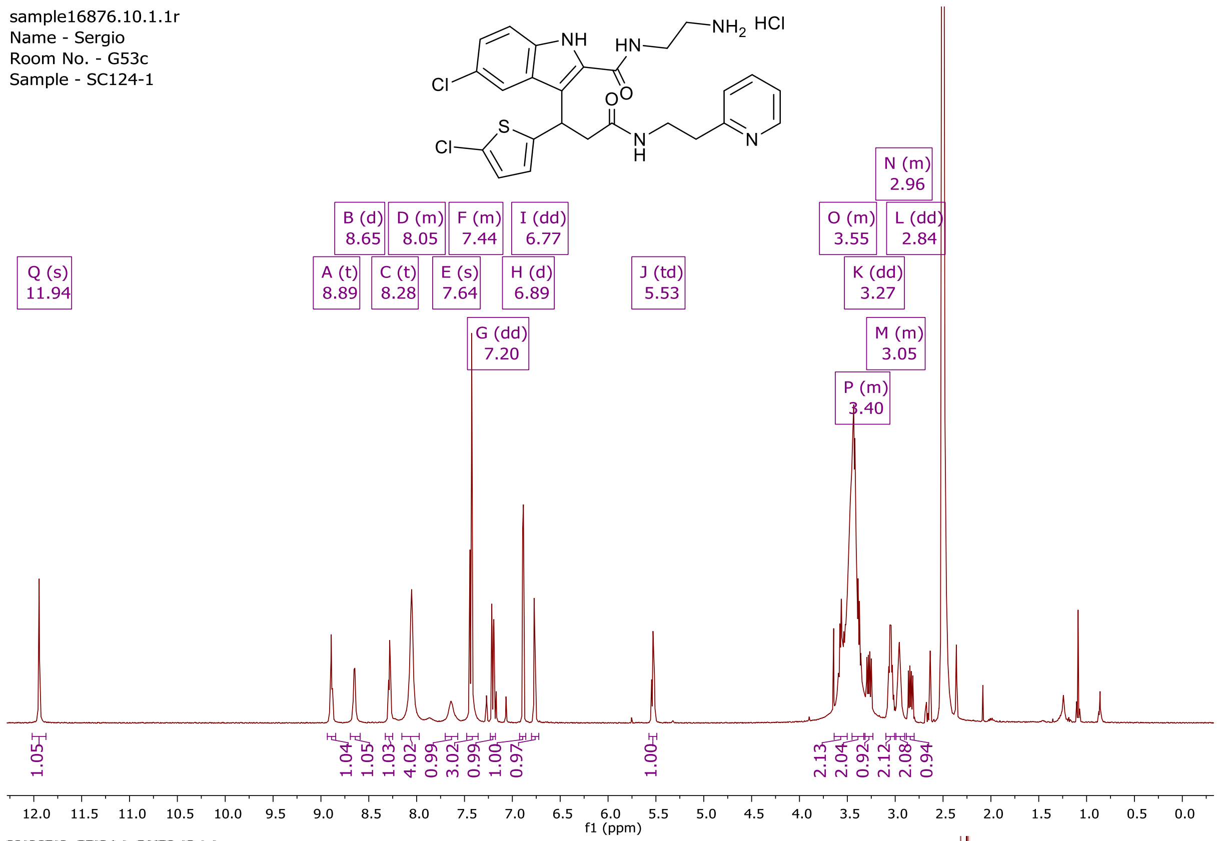

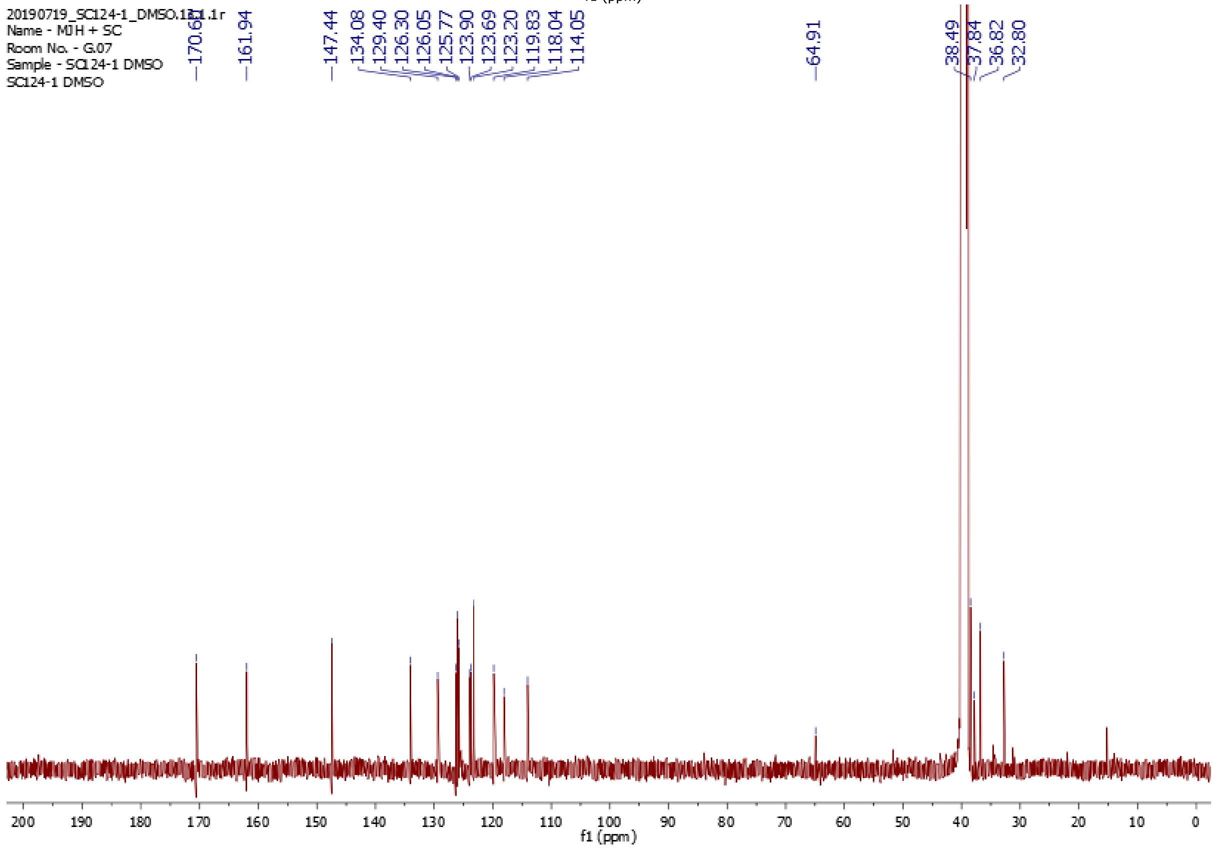

Ethyl 3-({5-chloro-3-[1-(5-chlorothiophen-2-yl)-2-{[2-(pyridin-2-yl)ethyl] carbamoyl}ethyl]-1H-indol-2-yl}formamido)propanoate

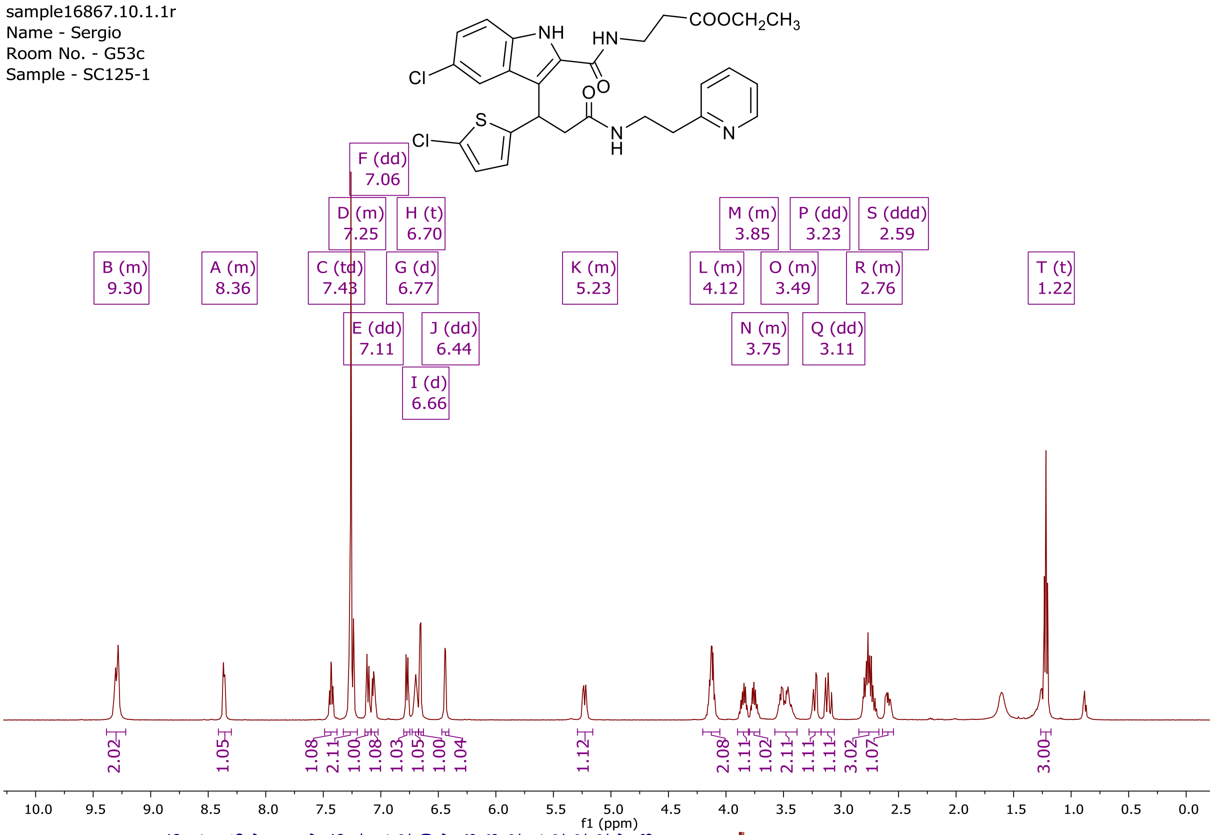

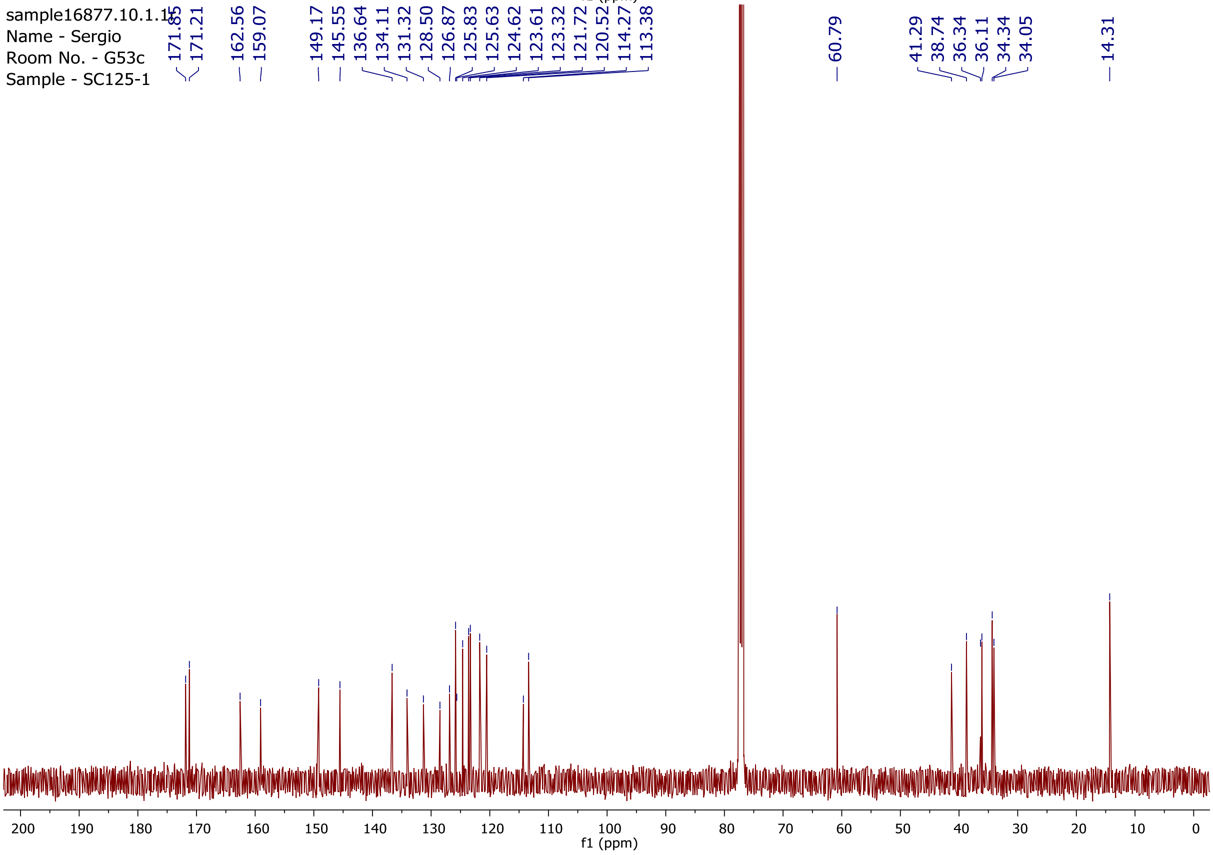

3-({5-Chloro-3-[1-(5-chlorothiophen-2-yl)-2-{[2-(pyridin-2-yl)ethyl]carbamoyl}ethyl]-1H-indol-2-yl}formamido)propanoic acid

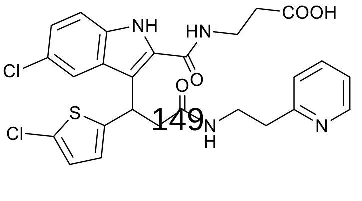

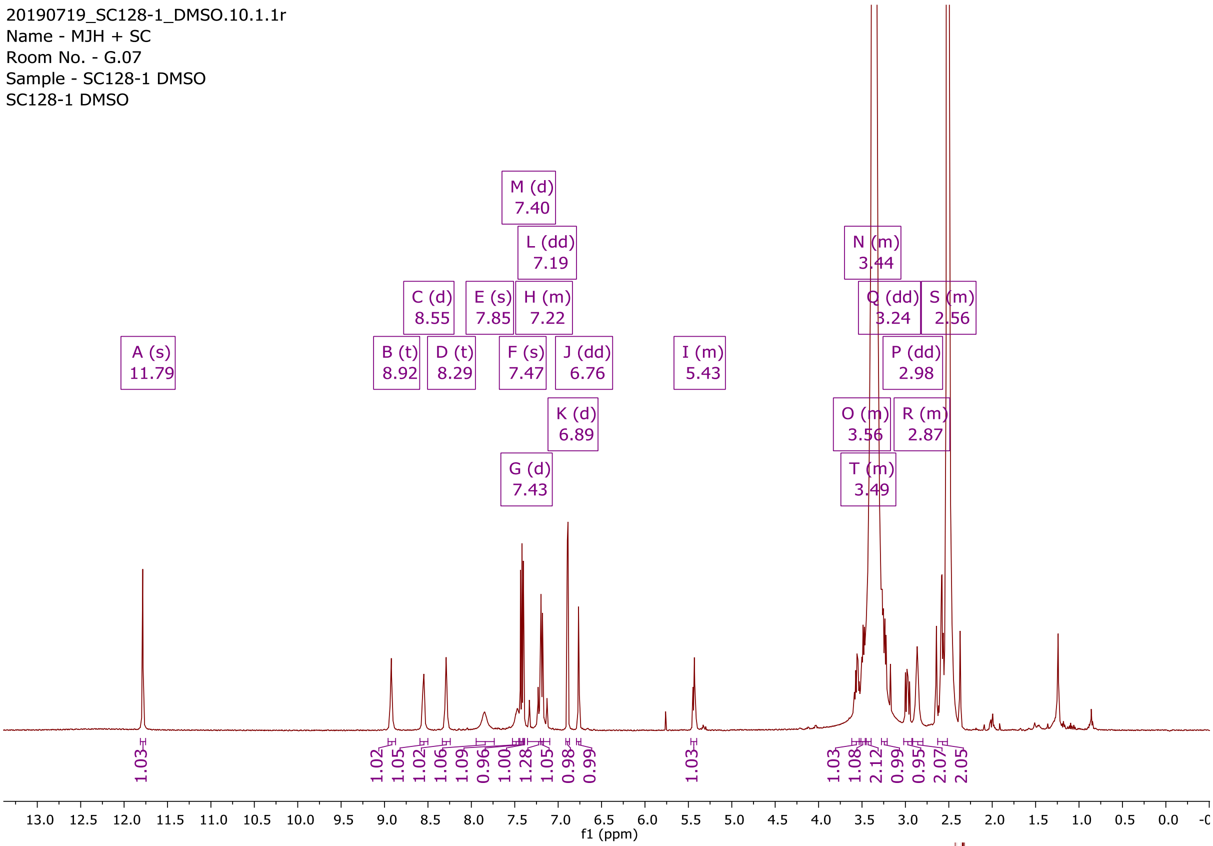

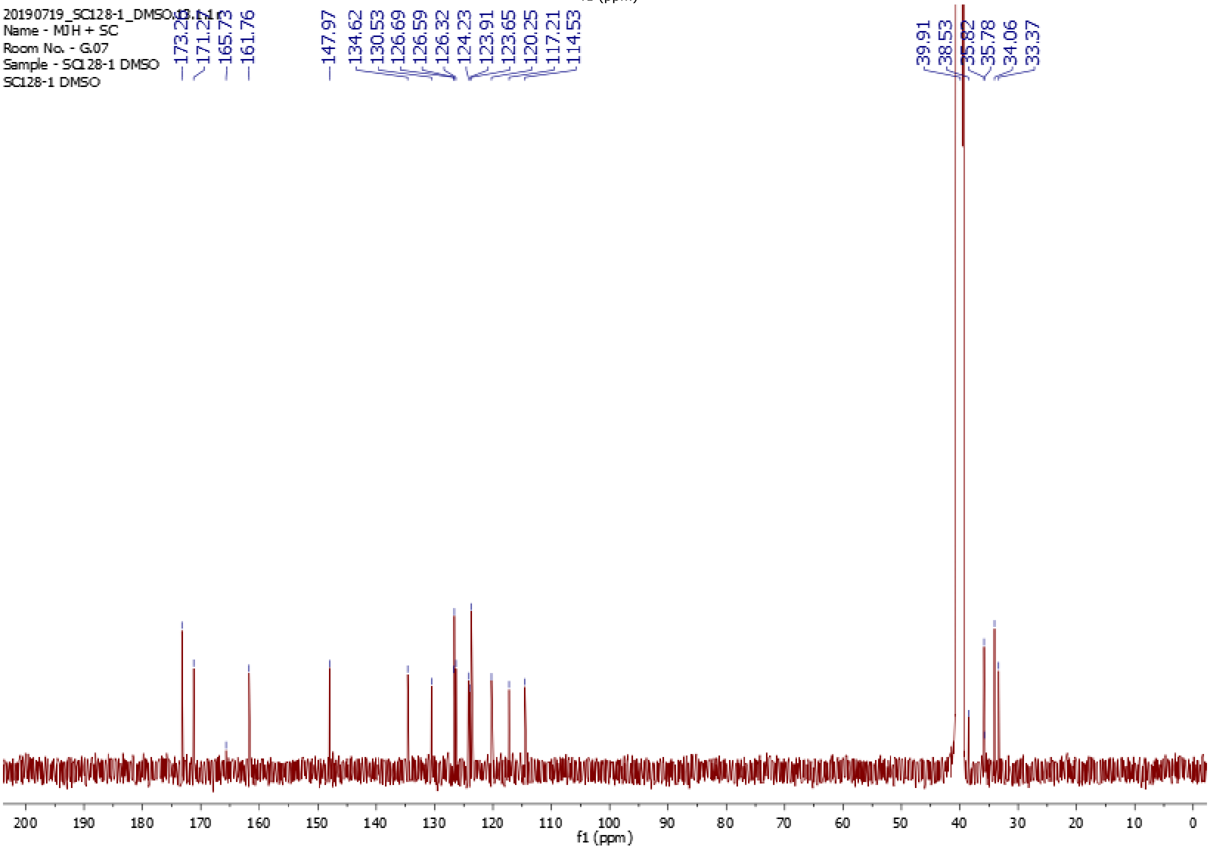

***rac*-*syn*- and *rac*-*anti*-Methyl 3-(5-chloro-1H-indol-3-yl)-3-(5-chlorothiophen-2-yl)-2-methylpropanoate**

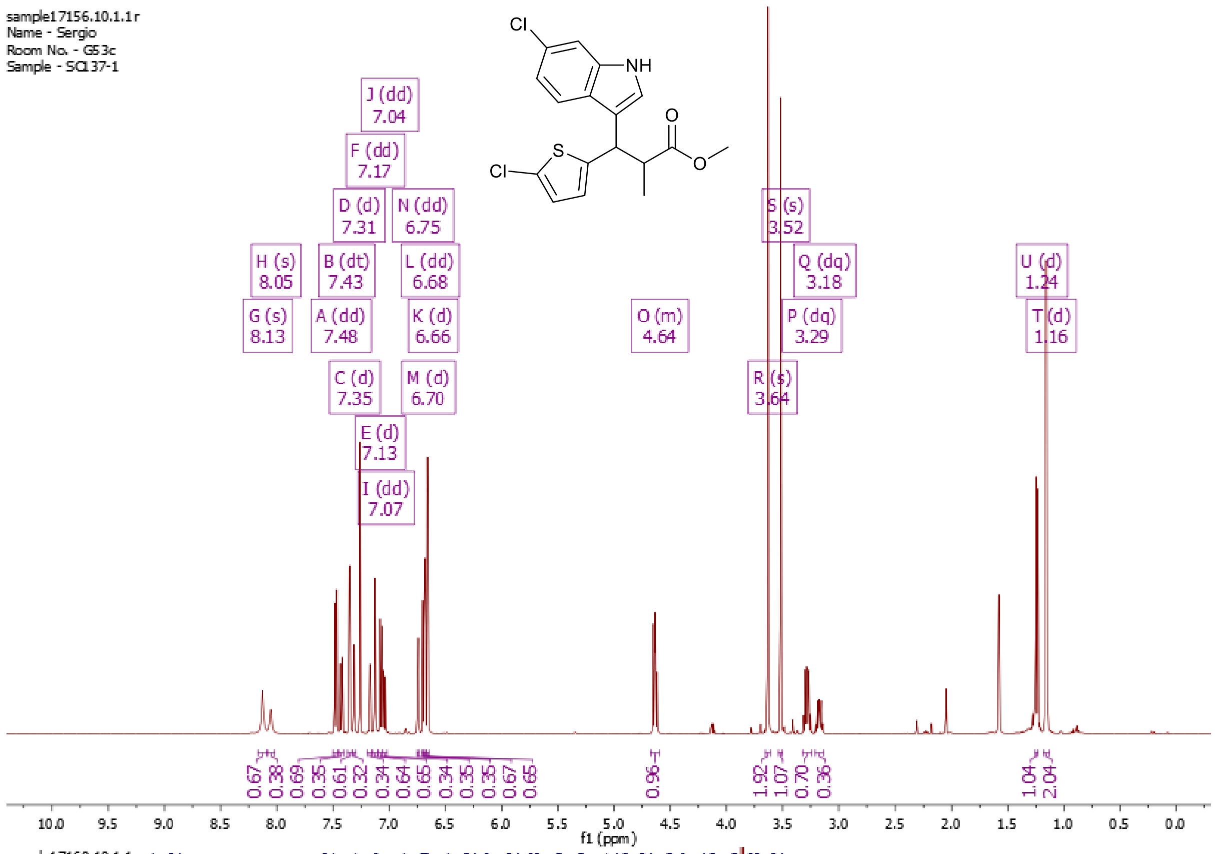

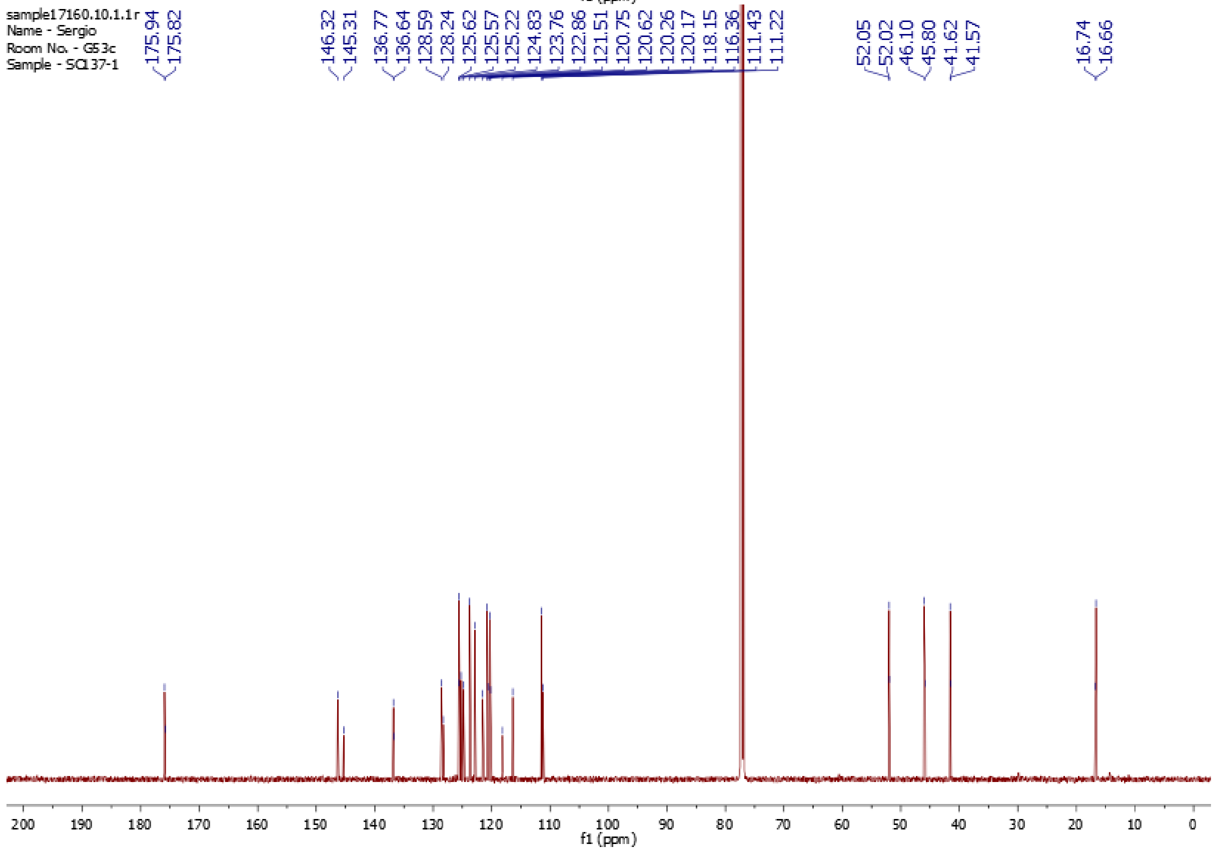

***rac-syn*- and *rac-anti*-Methyl 3-(2-bromo-5-chloro-1H-indol-3-yl)-3-(5-chlorothiophen-2-yl)-2-methyl propanoate**

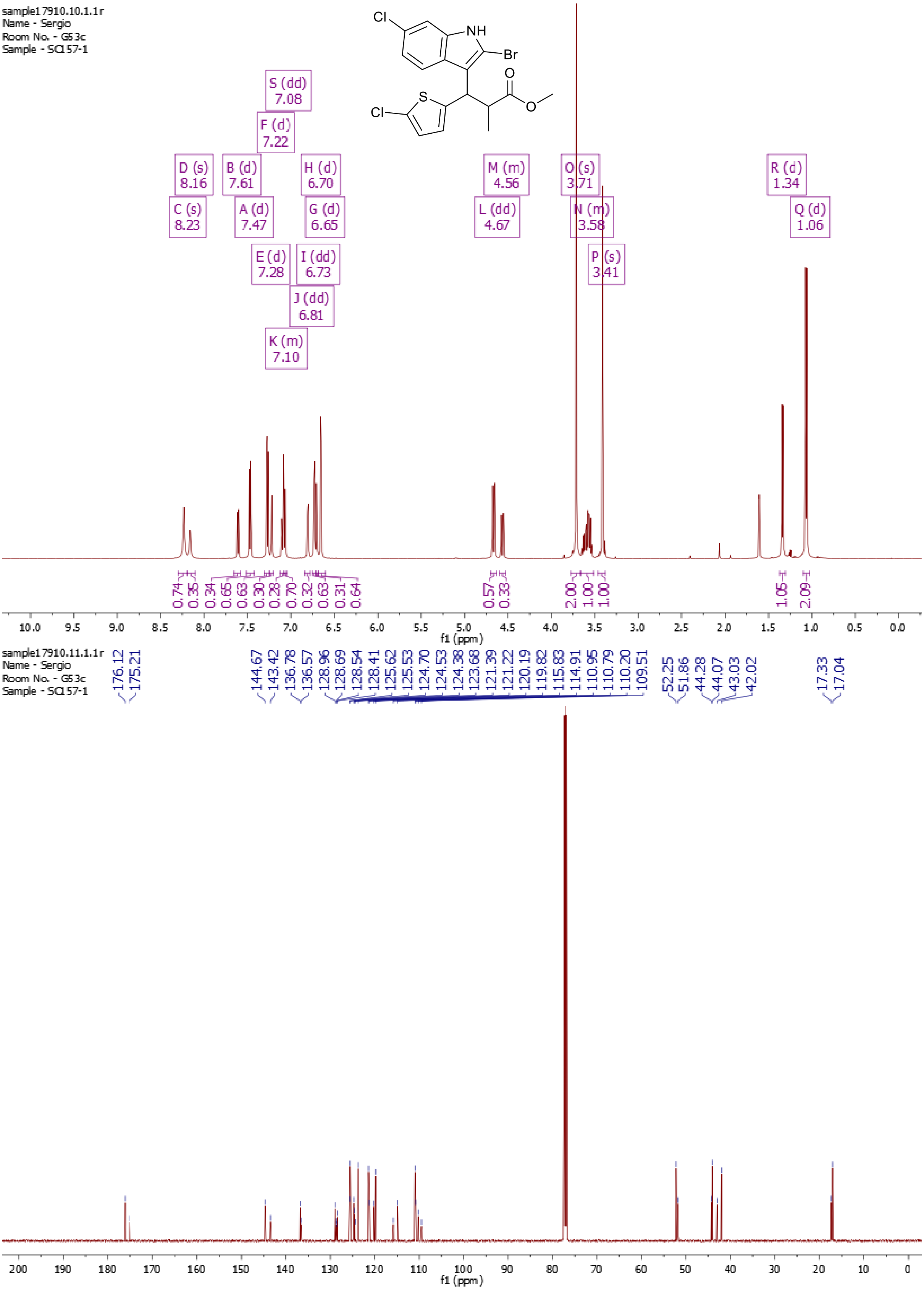

***rac-syn*- and *rac-anti*-3-(2-Bromo-6-chloro-1H-indol-3-yl)-3-(5-chlorothiophen-2-yl)-2-methyl propanoic acid**

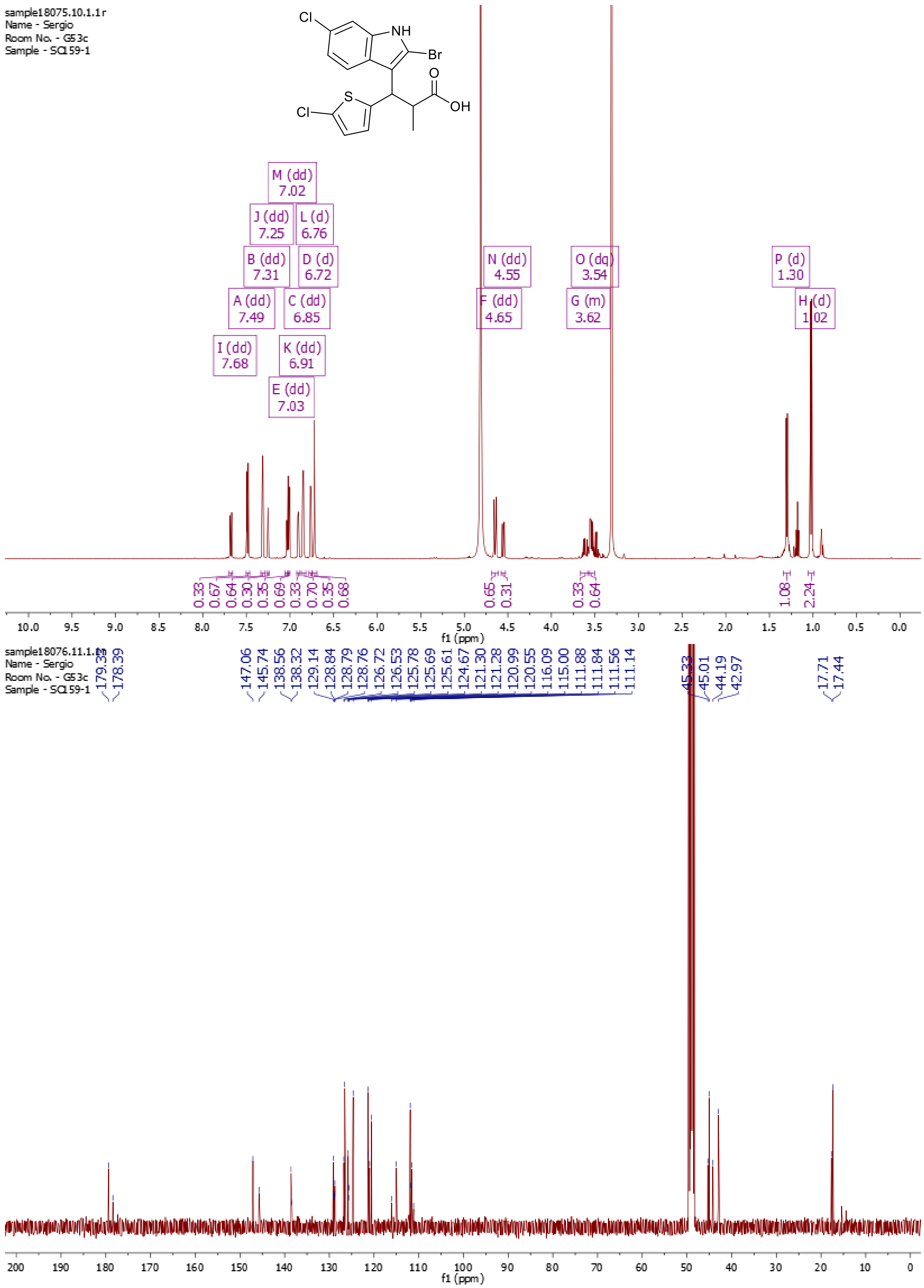

***rac-syn*-3-(2-Bromo-6-chloro-1H-indol-3-yl)-3-(5-chlorothiophen-2-yl)-2-methyl-*N*-[2-(pyridin-2-yl)ethyl]propanamide**

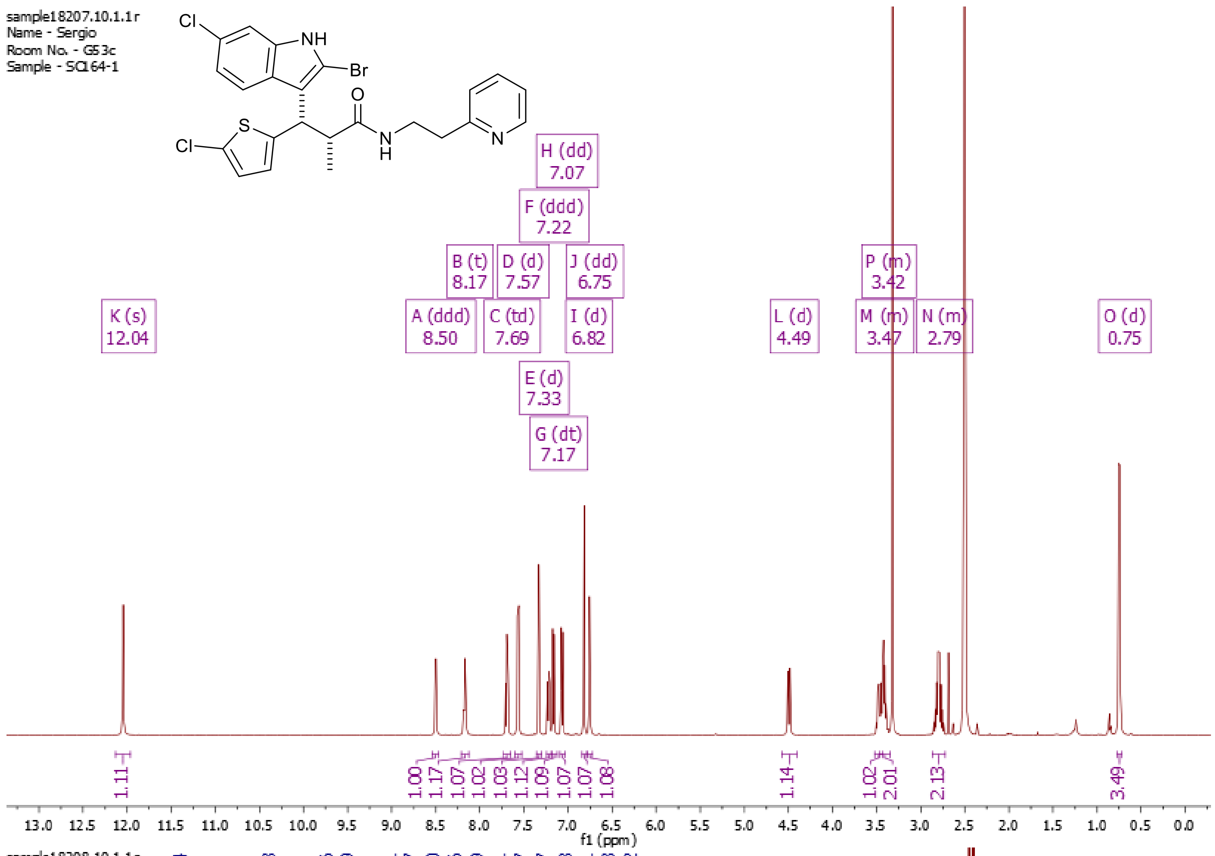

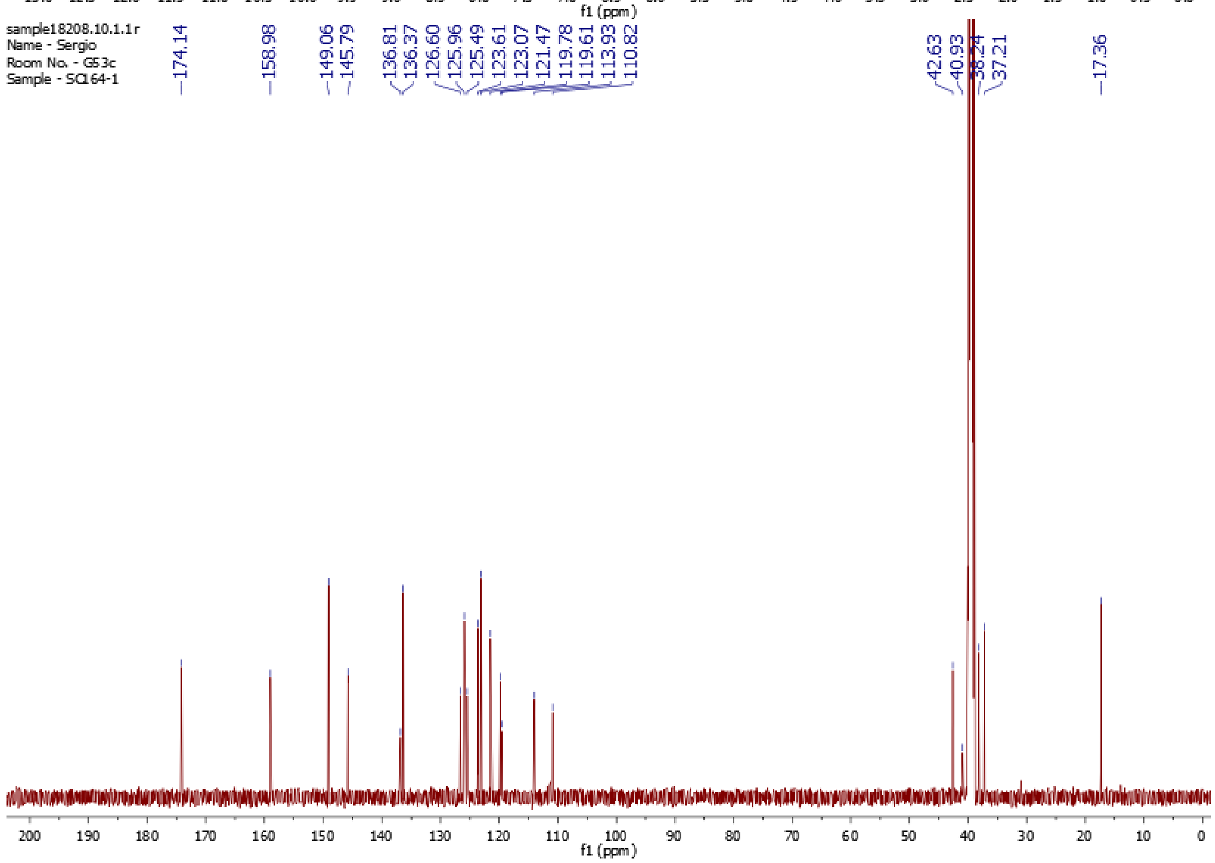

***rac-anti*-3-(2-Bromo-6-chloro-1H-indol-3-yl)-3-(5-chlorothiophen-2-yl)-2-methyl-*N*-[2-(pyridin-2-yl)ethyl]propanamide**

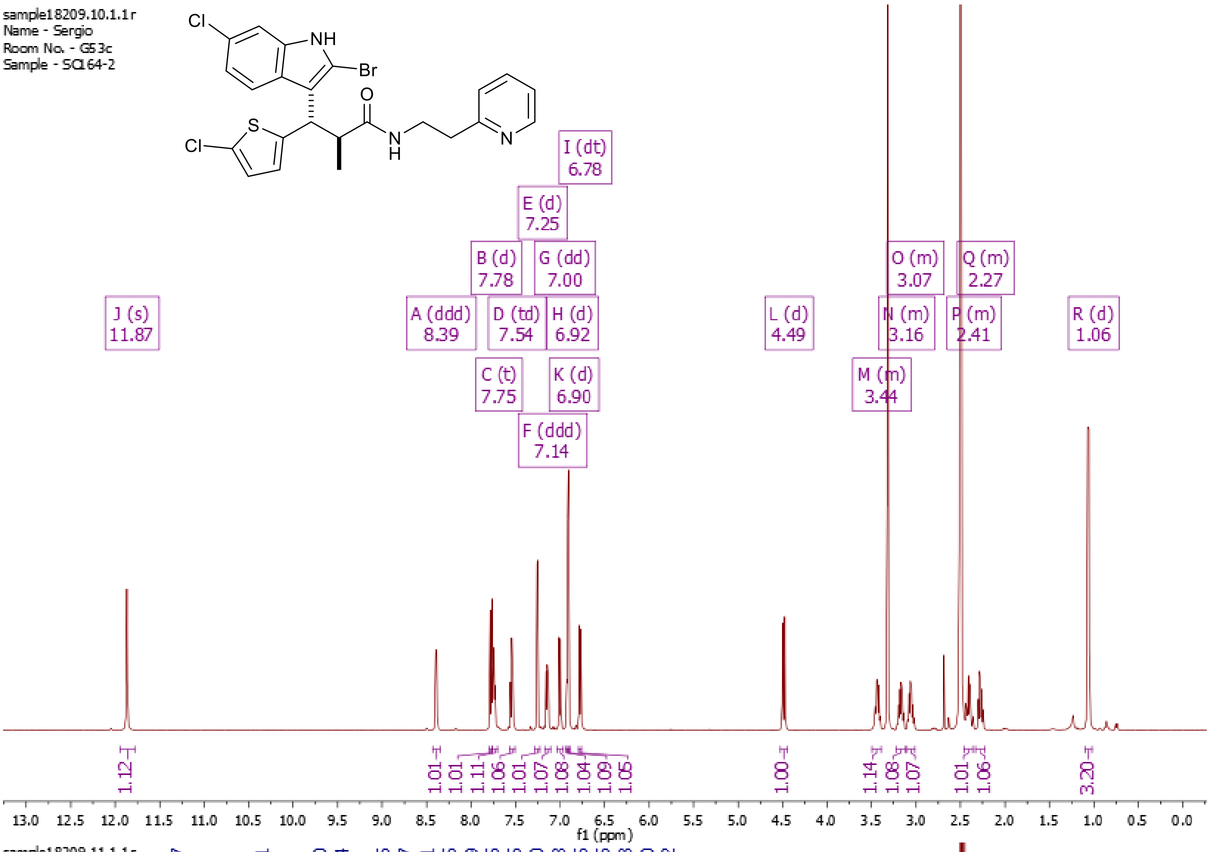

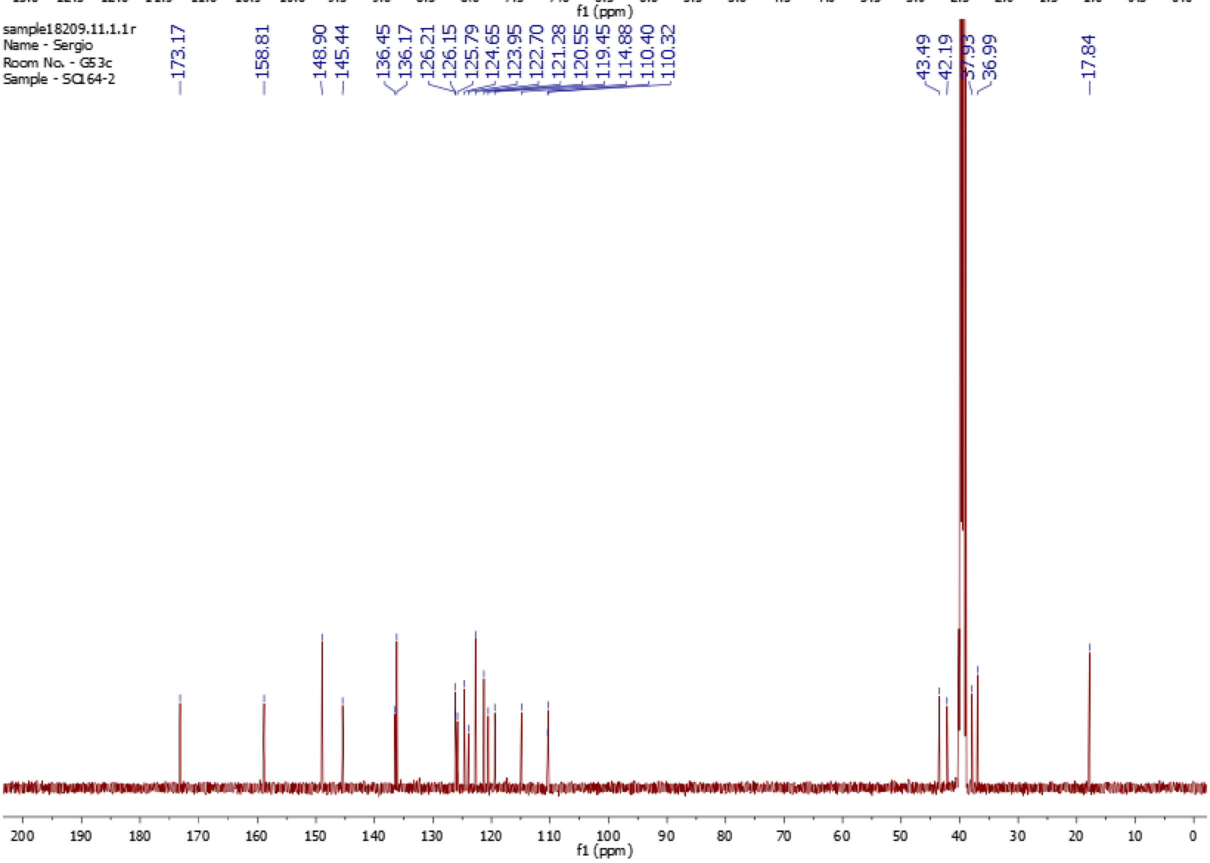

***rac-syn*- and *rac-anti*-3-(6-Chloro-1H-indol-3-yl)-3-(5-chlorothiophen-2-yl)-2-methylpropanoic acid**

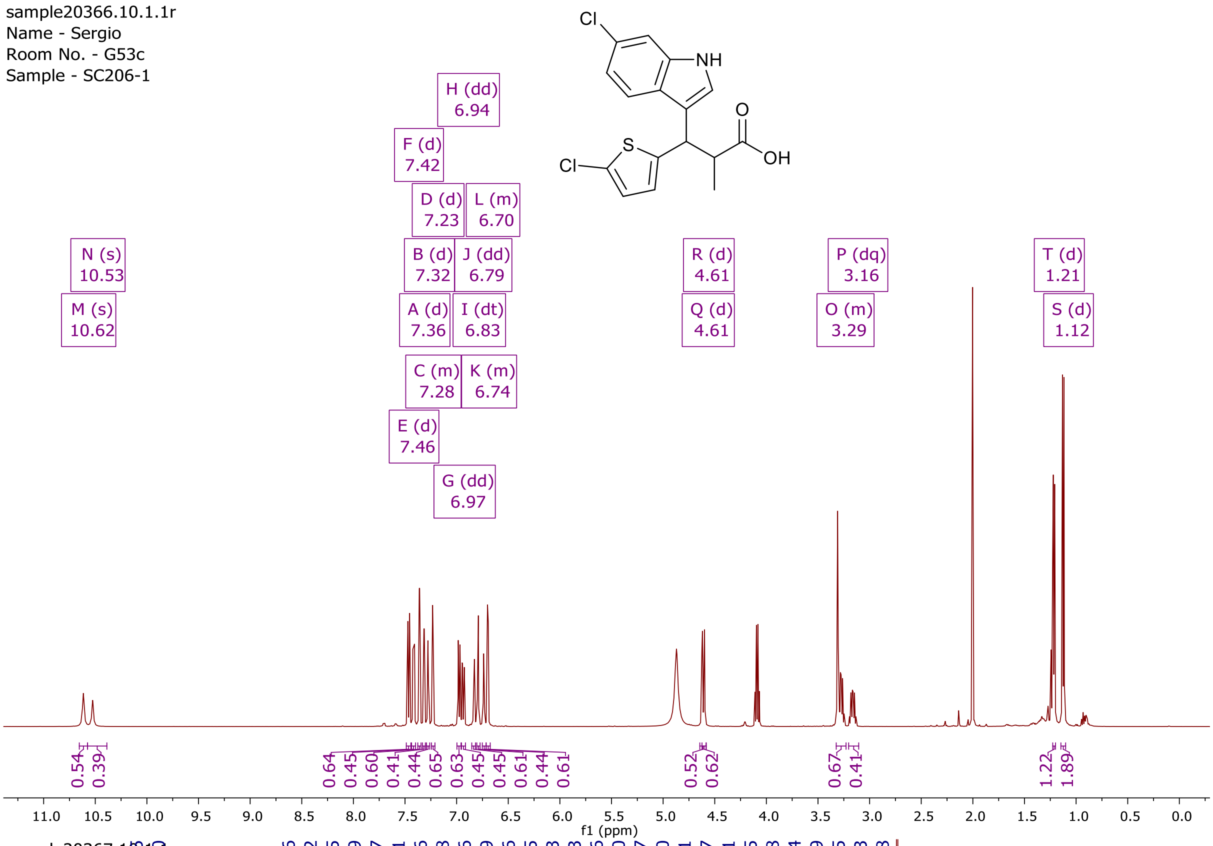

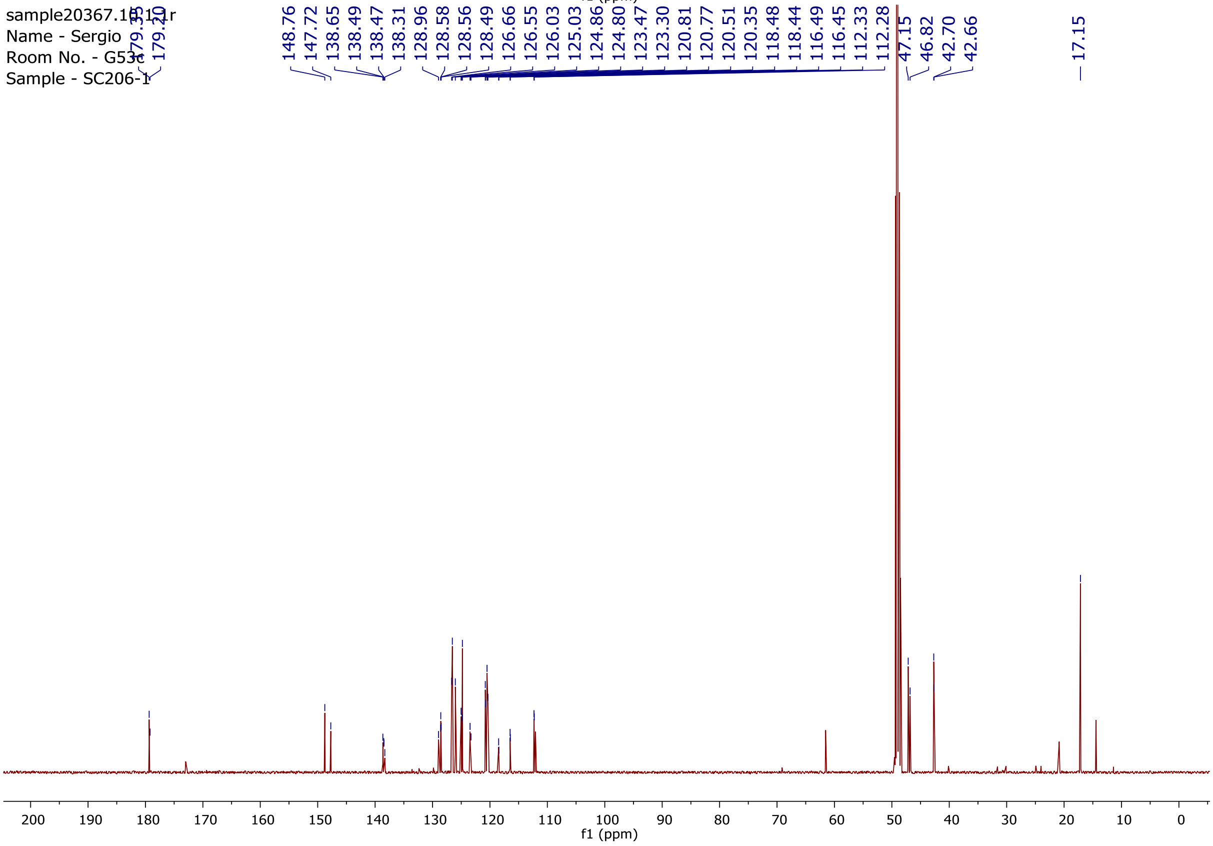

***rac-syn-* and *rac-anti*-3-(6-Chloro-1H-indol-3-yl)-3-(5-chlorothiophen-2-yl)-2-methyl-1-[3-(pyridin-2-yl)pyrrolidin-1-yl]propan-1-one**

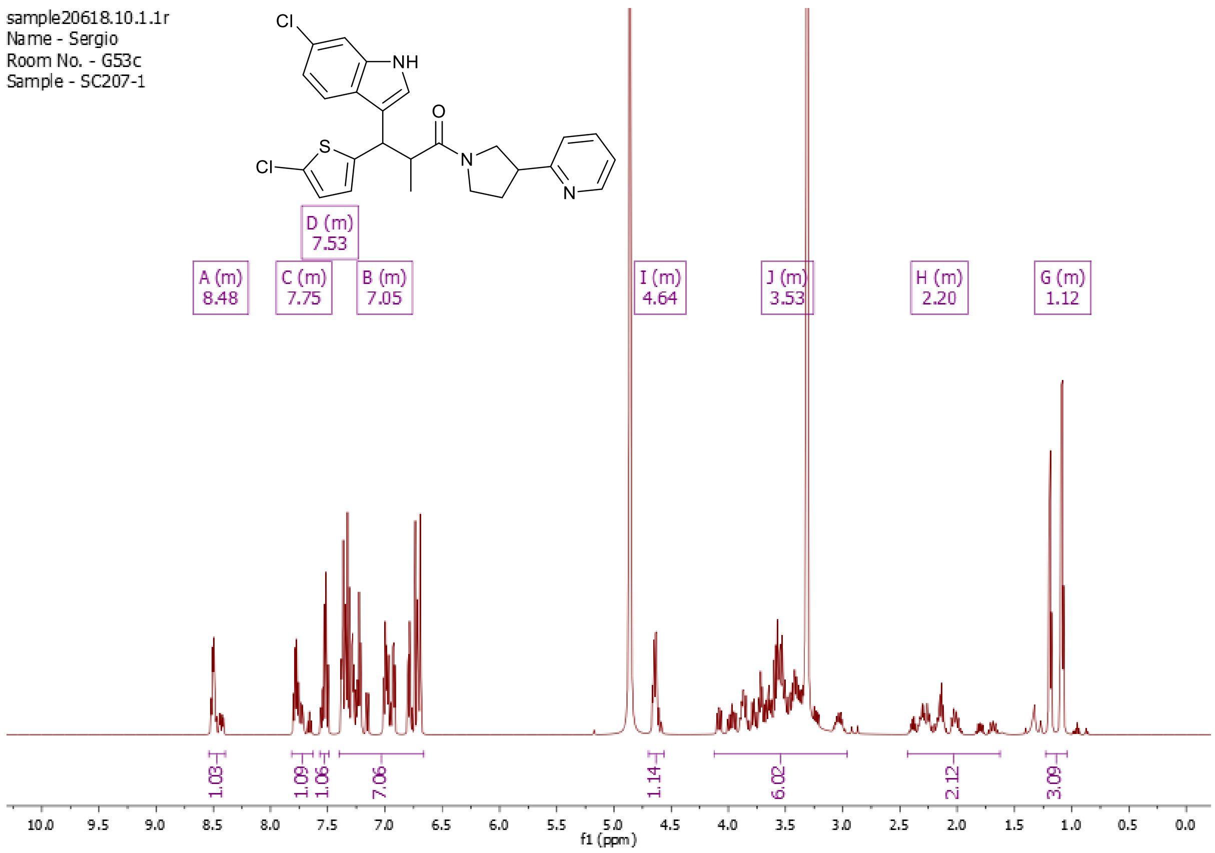

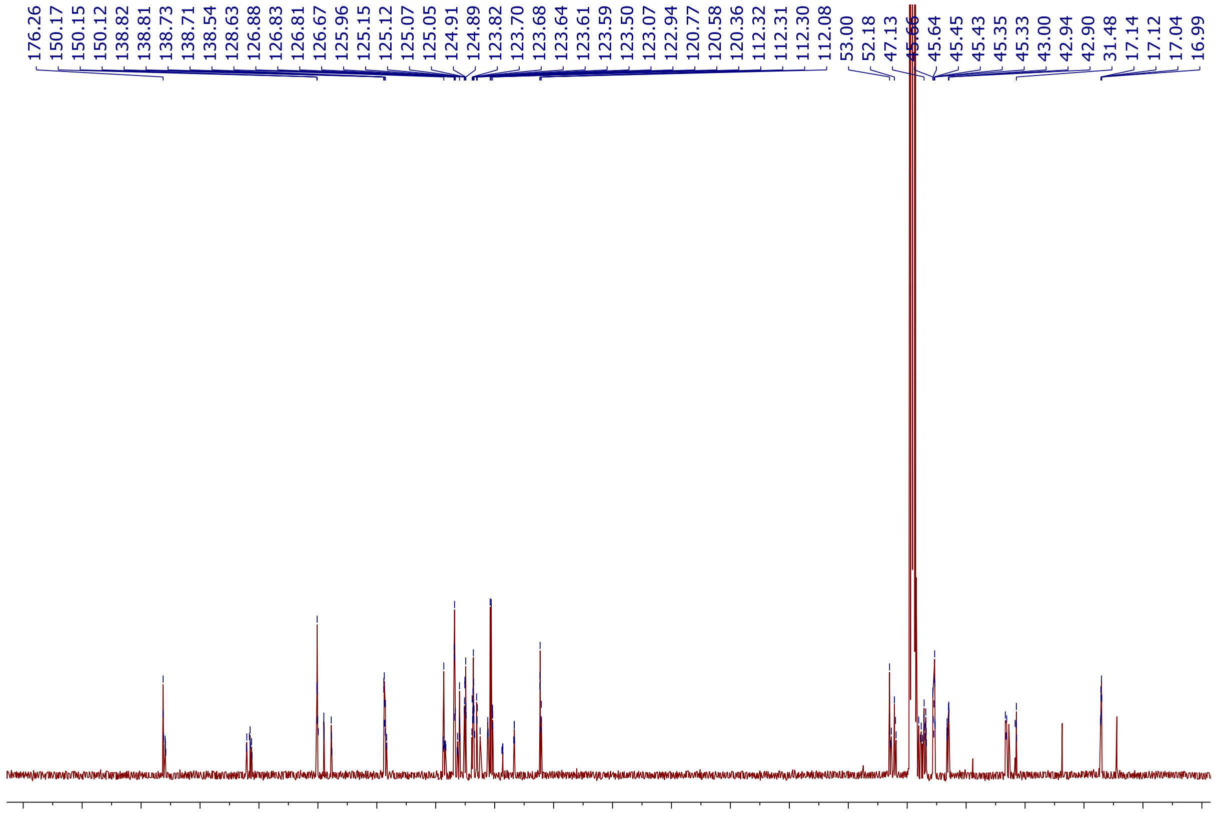

***rac-syn-* and *rac-anti*-3-(6-Chloro-1H-indol-3-yl)-3-(5-chloro thiophen-2-yl)-2-methyl-*N*-[2-phenylcyclopropyl]propenamide**

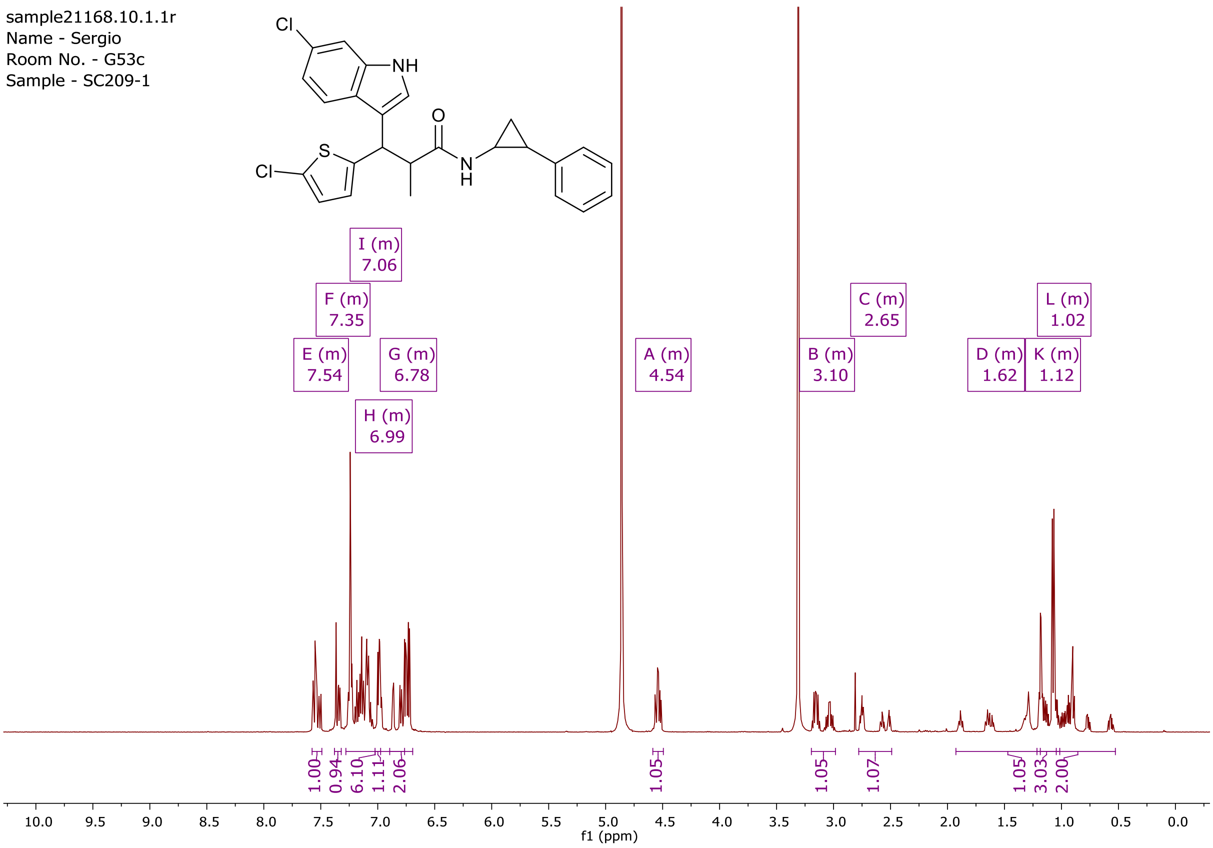

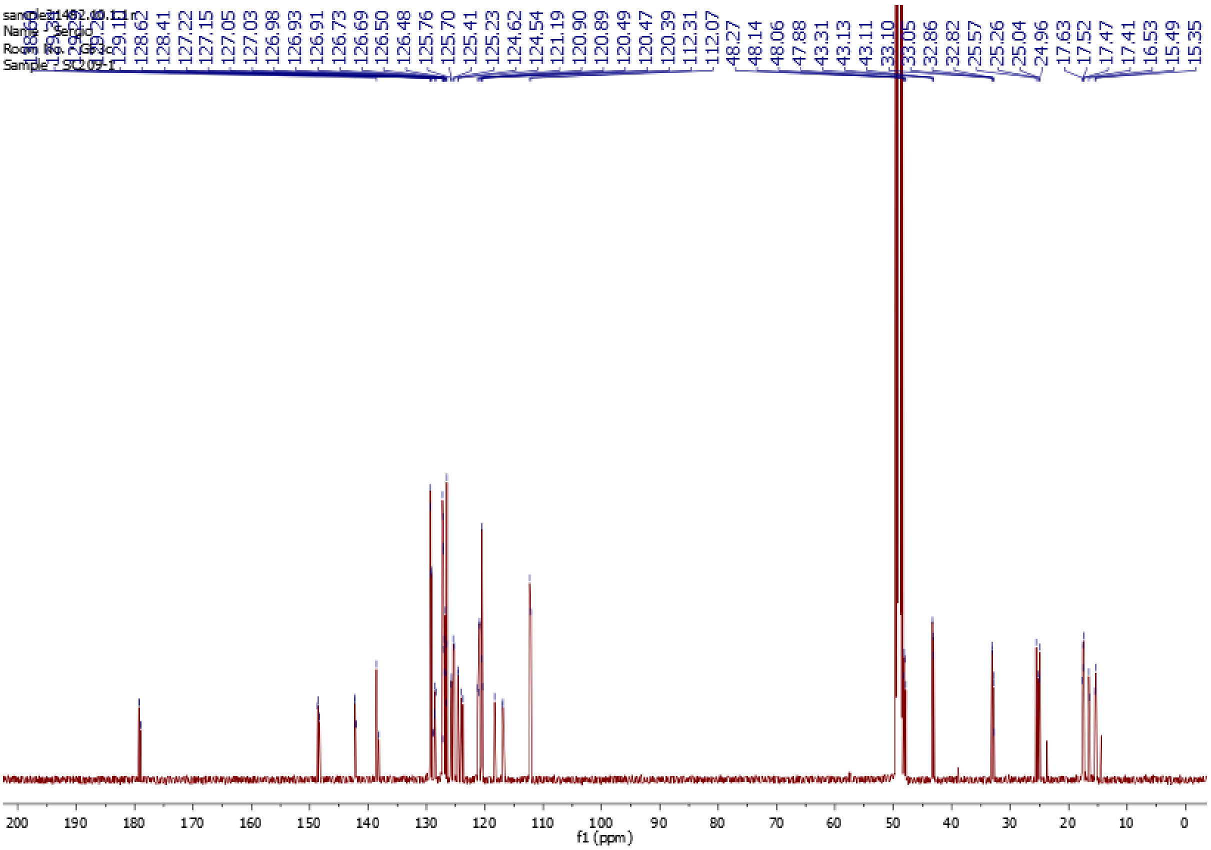

rac-3-(5-Chloro-1H-indol-3-yl)-3-(5-chlorothiophen-2-yl)-1-[3-(pyridin-2-yl)pyrrolidin-1-yl]propan-1-one

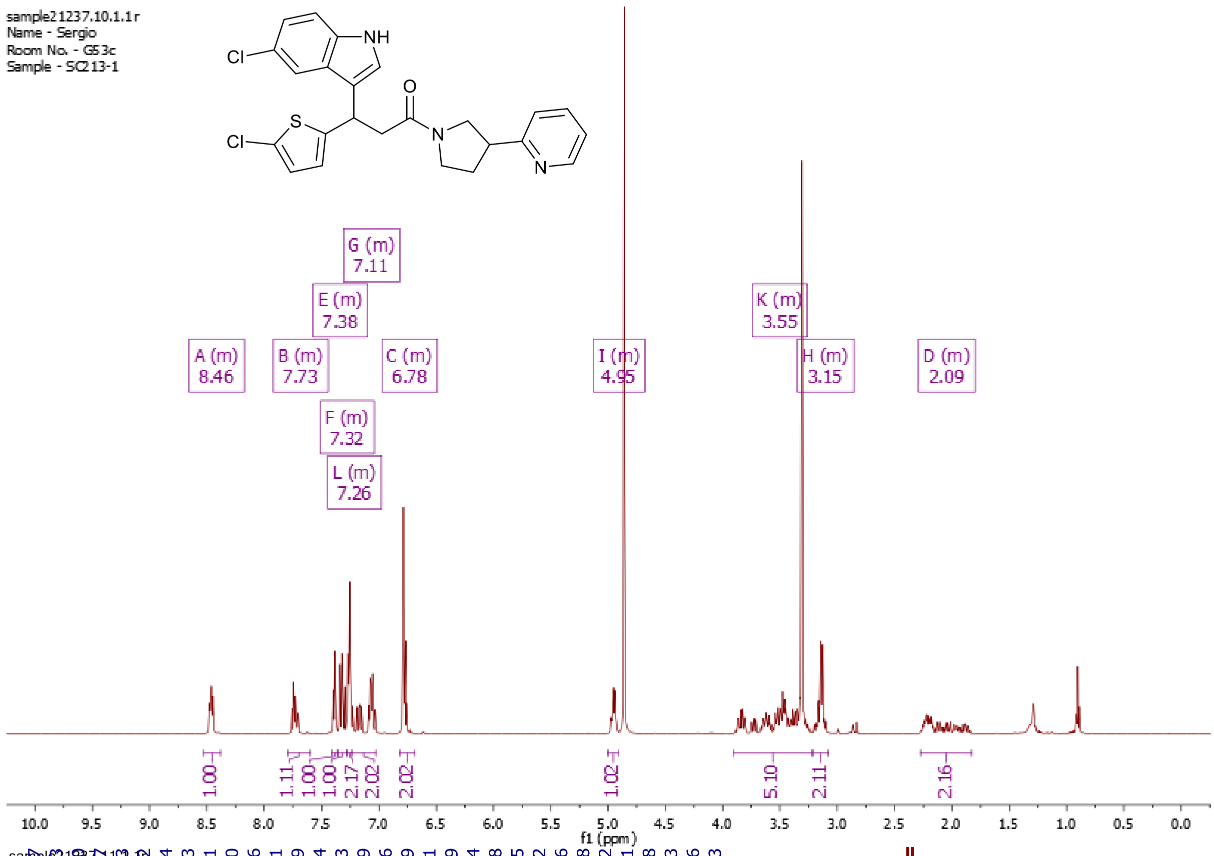

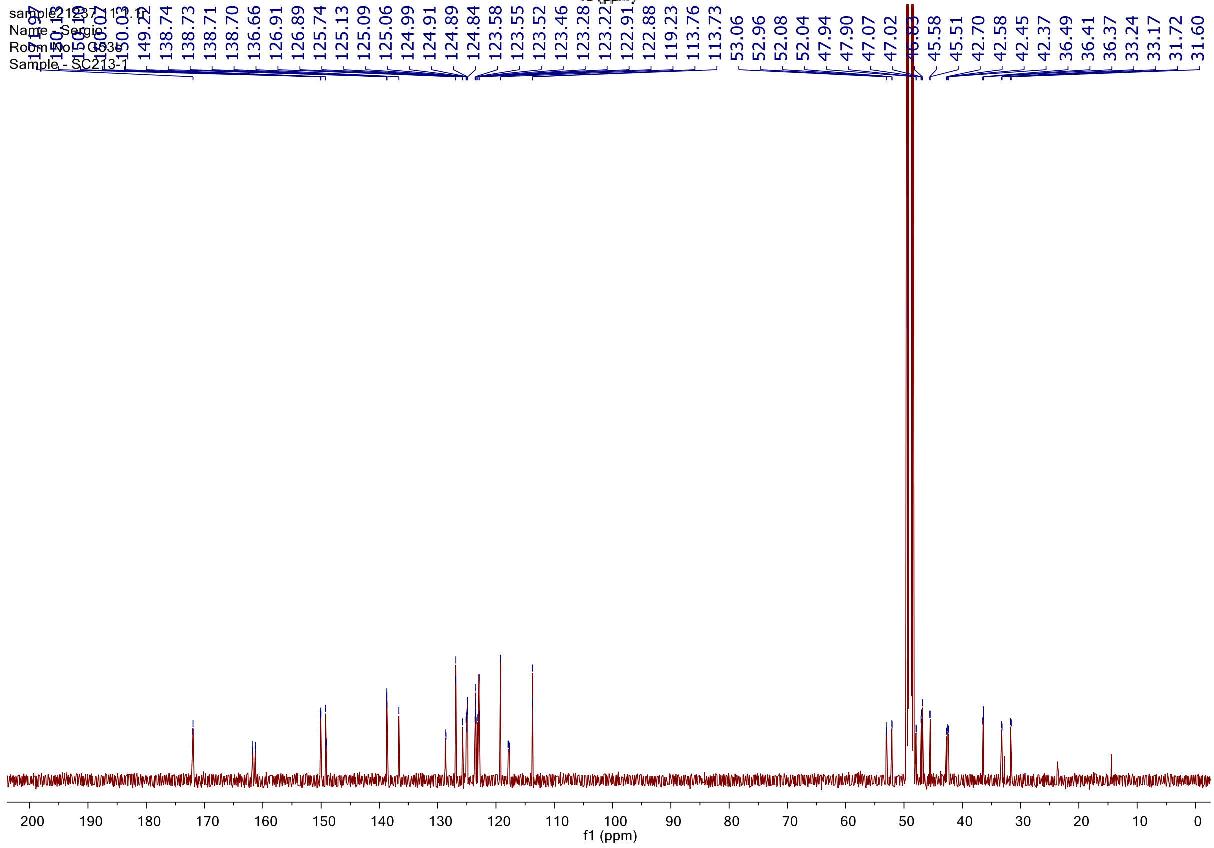

***rac*-3-(5-Chloro-1H-indol-3-yl)-3-(5-chlorothiophen-2-yl)-*N*-[2-phenyl cyclopropyl]propanamide**

sample21236.10.1.1r
Name - Sergio
Room No. - G53c
Sample - SC212-1

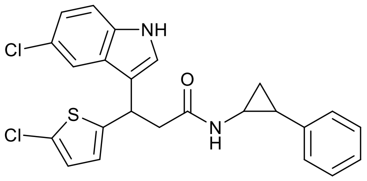

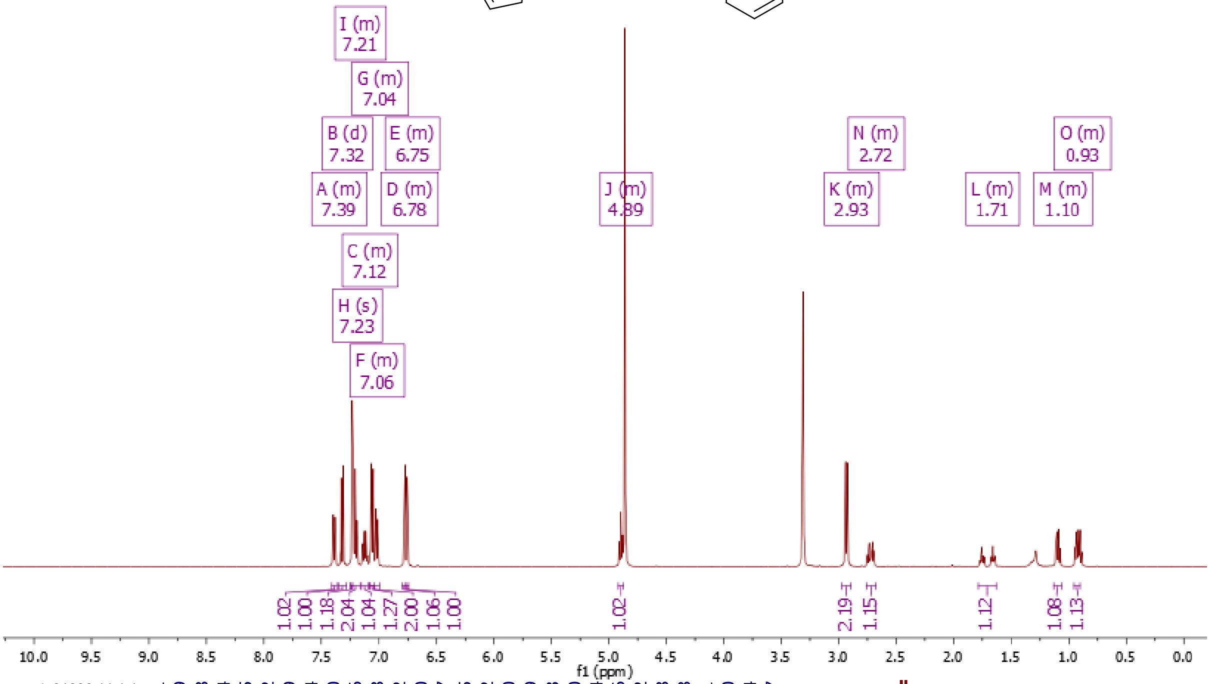

sample21236.11.1.1r
Name - Sergio
Room No. - G53c
Sample - SC212-1

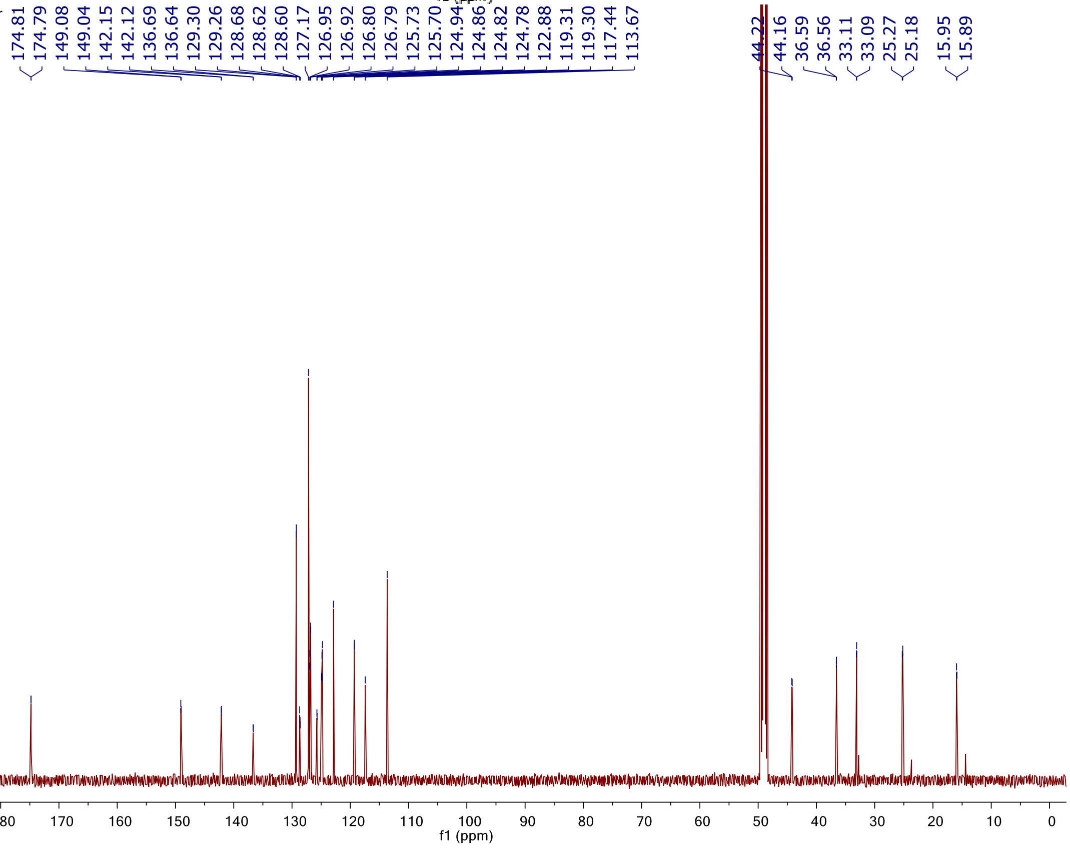

5-chloro-3-[1-(5-chlorothiophen-2-yl)-2-{[2-phenylcyclopropyl]carbamoyl}ethyl]-1H-indole-2-carboxylic acid

sample23315.10.1.1r
Name - Sergi Celis
Room No. - G53d
Sample - SC236-1

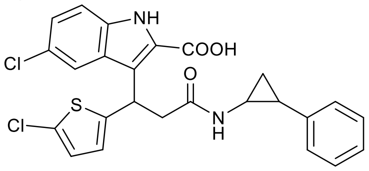

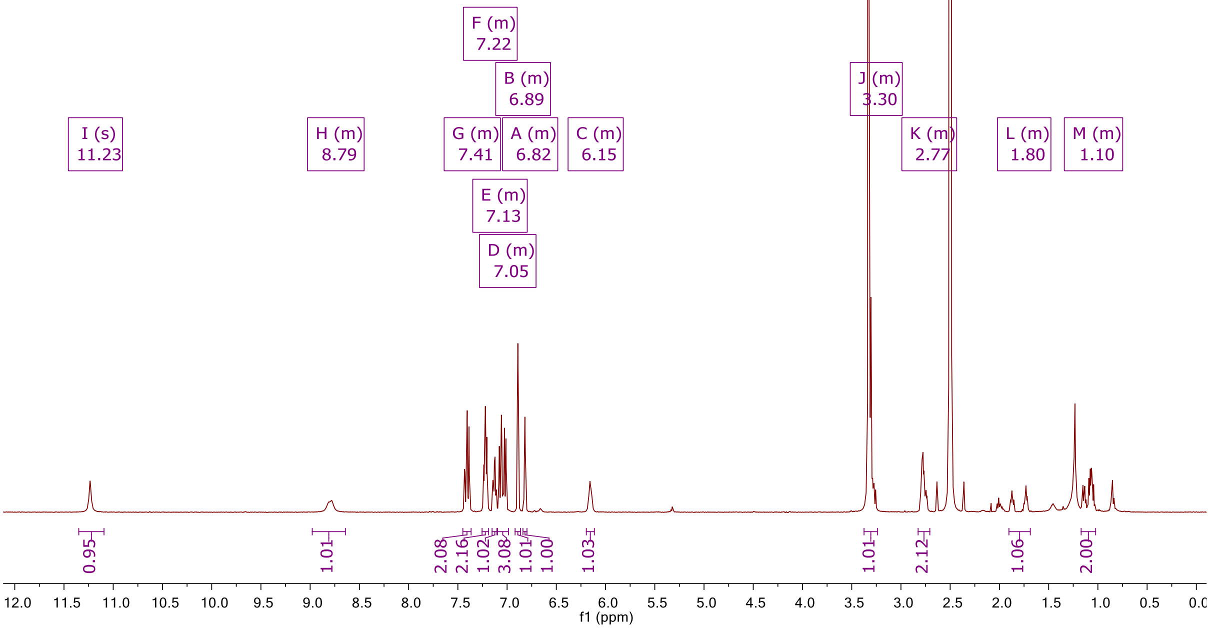

sample23315.11.1.1r
Name - Sergi Celis
Room No. - G53d
Sample - SC236-1

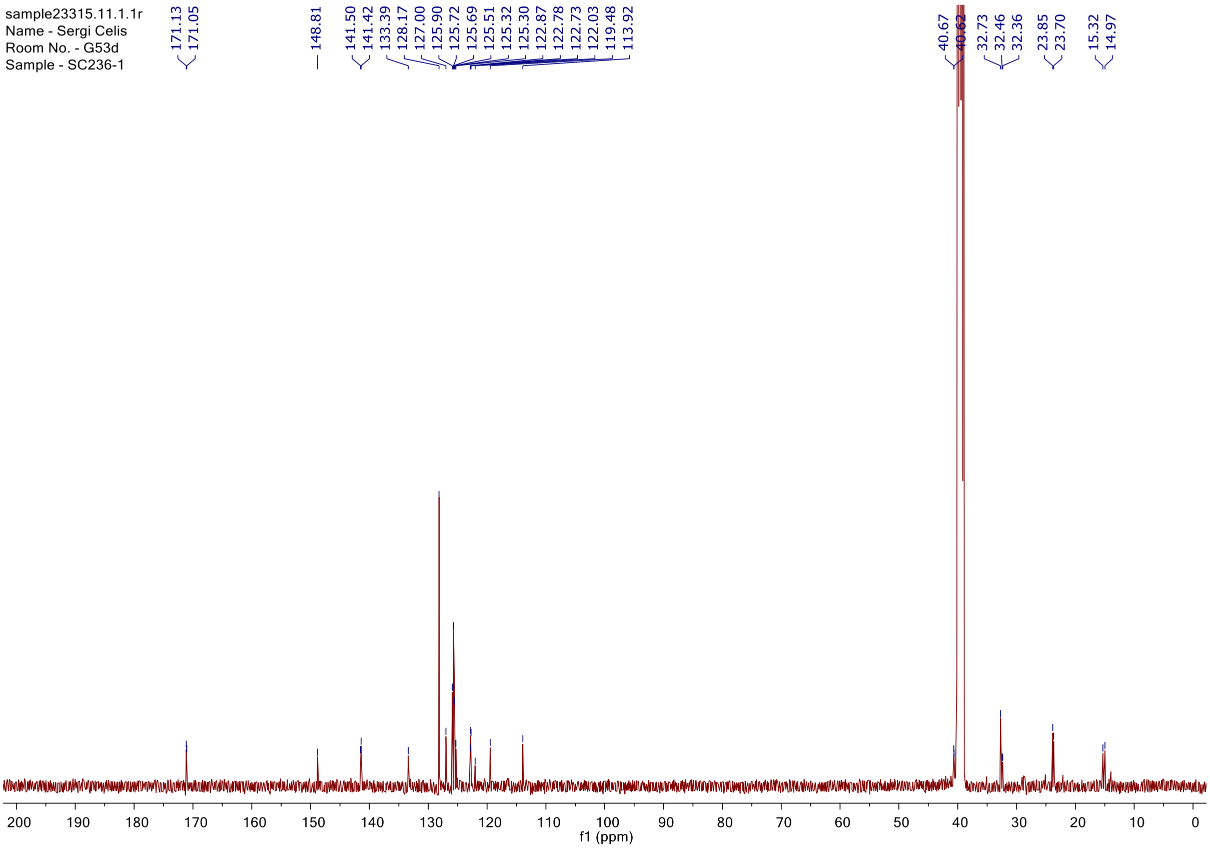

10. Bibliography

1. A. I. Green, F. Hobor, C. P. Tinworth, S. Warriner, A. J. Wilson and A. Nelson, *Chem. Eur. J.*, 2020, **n/a**, 10.1002/chem.202002153.
2. A. A. Ibarra, G. J. Bartlett, Z. Hegedüs, S. Dutt, F. Hobor, K. A. Horner, K. Hetherington, K. Spence, A. Nelson, T. A. Edwards, D. N. Woolfson, R. B. Sessions and A. J. Wilson, *ACS. Chem. Biol.*, 2019, **14**, 2252-2263.
3. V. Azzarito, P. Prabhakaran, A. I. Bartlett, N. S. Murphy, M. J. Hardie, C. A. Kilner, T. A. Edwards, S. L. Warriner and A. J. Wilson, *Organic & Biomolecular Chemistry*, 2012, **10**, 6469-6472.
4. J. P. Plante, T. Burnley, B. Malkova, M. E. Webb, S. L. Warriner, T. A. Edwards and A. J. Wilson, *Chem. Commun.*, 2009, DOI: 10.1039/B908207G, 5091-5093.
5. W. Lee, M. Tonelli and J. L. Markley, *Bioinformatics*, 2014, **31**, 1325-1327.
6. T. D. Goddard and D. G. Kneller, *University of California, San Fransisco, USA.*, 2006.
7. G. Winter, *J. Appl. Crystallogr.*, 2010, **43**, 186-190.
8. W. Kabsch, *Acta Crystallographica Section D*, 2010, **66**, 125-132.
9. P. Evans, *Acta Crystallographica Section D*, 2006, **62**, 72-82.
10. P. R. Evans and G. N. Murshudov, *Acta Crystallographica Section D*, 2013, **69**, 1204-1214.
11. A. J. McCoy, R. W. Grosse-Kunstleve, P. D. Adams, M. D. Winn, L. C. Storoni and R. J. Read, *J. Appl. Crystallogr.*, 2007, **40**, 658-674.
12. A. A. Vagin, R. A. Steiner, A. A. Lebedev, L. Potterton, S. McNicholas, F. Long and G. N. Murshudov, *Acta Crystallographica Section D*, 2004, **60**, 2184-2195.
13. P. Emsley, B. Lohkamp, W. G. Scott and K. Cowtan, *Acta Crystallographica Section D*, 2010, **66**, 486-501.
14. M. D. Winn, C. C. Ballard, K. D. Cowtan, E. J. Dodson, P. Emsley, P. R. Evans, R. M. Keegan, E. B. Krissinel, A. G. W. Leslie, A. McCoy, S. J. McNicholas, G. N. Murshudov, N. S. Pannu, E. A. Potterton, H. R. Powell, R. J. Read, A. Vagin and K. S. Wilson, *Acta Crystallographica Section D*, 2011, **67**, 235-242.
15. P. V. Afonine, R. W. Grosse-Kunstleve, N. Echols, J. J. Headd, N. W. Moriarty, M. Mustyakimov, T. C. Terwilliger, A. Urzhumtsev, P. H. Zwart and P. D. Adams, *Acta Crystallographica Section D*, 2012, **68**, 352-367.
16. V. B. Chen, W. B. Arendall, III, J. J. Headd, D. A. Keedy, R. M. Immormino, G. J. Kapral, L. W. Murray, J. S. Richardson and D. C. Richardson, *Acta Crystallographica Section D*, 2010, **66**, 12-21.
17. C. W. Wood, A. A. Ibarra, G. J. Bartlett, A. J. Wilson, D. N. Woolfson and R. B. Sessions, *Bioinformatics*, 2020, DOI: 10.1093/bioinformatics/btaa026.
18. J. A. Grant, M. A. Gallardo and B. T. Pickup, *J. Comput. Chem.*, 1996, **17**, 1653-1666.
19. J. J. Sutherland, R. K. Nandigam, J. A. Erickson and M. Vieth, *Journal of Chemical Information and Modeling*, 2007, **47**, 2293-2302.
20. T. Tuccinardi, G. Ortore, M. A. Santos, S. M. Marques, E. Nuti, A. Rossello and A. Martinelli, *Journal of Chemical Information and Modeling*, 2009, **49**, 1715-1724.
21. T. S. Rush, J. A. Grant, L. Mosyak and A. Nicholls, *J. Med. Chem.*, 2005, **48**, 1489-1495.

22. R. P. Sheridan, G. B. McGaughey and W. D. Cornell, *J. Comput. Aided Mol. Des.*, 2008, **22**, 257-265.
23. J. Venhorst, S. Núñez, J. W. Terpstra and C. G. Kruse, *J. Med. Chem.*, 2008, **51**, 3222-3229.
24. P. C. D. Hawkins, A. G. Skillman and A. Nicholls, *J. Med. Chem.*, 2007, **50**, 74-82.
25. S. McIntosh-Smith, T. Wilson, A. Á. Ibarra, J. Crisp and R. B. Sessions, *The Computer Journal*, 2012, **55**, 192-205.
26. S. McIntosh-Smith, J. Price, R. B. Sessions and A. A. Ibarra, *International Journal of High Performance Computing Applications*, 2015, **29**, 119–134.
27. J. W. M. Nissink and S. Blackburn, *Future Medicinal Chemistry*, 2014, **6**, 1113-1126.
28. K. Lindorff-Larsen, S. Piana, K. Palmo, P. Maragakis, J. L. Klepeis, R. O. Dror and D. E. Shaw, *Proteins*, 2010, **78**, 1950-1958.
29. J. Wang, R. M. Wolf, J. W. Caldwell, P. A. Kollman and D. A. Case, *J. Comput. Chem.*, 2004, **25**, 1157–1174.
30. H. J. C. Berendsen, D. van der Spoel and R. van Drunen, *Comput. Phys. Commun.*, 1995, **91**, 43-56.
31. W. Humphrey, A. Dalke and K. Schulten, *J. Mol. Graphics*, 1996, **14**, 33-38.
32. E. F. Pettersen, T. D. Goddard, C. C. Huang, G. S. Couch, D. M. Greenblatt, E. C. Meng and T. E. Ferrin, *J. Comput. Chem.*, 2004, **25**, 1605-1612.
33. A. Barnard, K. Long, H. L. Martin, J. A. Miles, T. A. Edwards, D. C. Tomlinson, A. Macdonald and A. J. Wilson, *Angewandte Chemie International Edition*, 2015, **54**, 2960-2965.
34. V. Azzarito, J. A. Miles, J. Fisher, T. A. Edwards, S. Warriner and A. Wilson, *Chem. Sci.*, 2015, **6**, 2434-2443.
35. J. Saupe, Y. Roske, C. Schillinger, N. Kamdem, S. Radetzki, A. Diehl, H. Oschkinat, G. Krause, U. Heinemann and J. Rademann, *ChemMedChem*, 2011, **6**, 1411-1422.
